# Integrated analysis of bulk RNA-seq and single-cell RNA-seq reveals the function of pyrocytosis in the pathogenesis of abdominal aortic aneurysm

**DOI:** 10.18632/aging.205350

**Published:** 2023-12-18

**Authors:** Shiqi Zhao, Hai Cang, Ying Liu, Yanjie Huang, Song Zhang

**Affiliations:** 1 Department of Intensive Care Unit, The First Affiliated Hospital, School of Medicine, Zhejiang University, Hangzhou 310003, Zhejiang, China; 2 Department of Cardiology, The Fourth Affiliated Hospital of Harbin Medical University, Harbin 150001, Heilongjiang, China; 3 Department of Anesthesiology, Heilongjiang Provincial Hospital, Harbin 150036, Heilongjiang, China; 4 Department of Cardiology, The First Affiliated Hospital of Harbin Medical University, Harbin 150001, Heilongjiang, China

**Keywords:** abdominal aortic aneurysm, pyroptosis, diagnosis biomarkers, single-cell sequencing, immune cells

## Abstract

Pyrocytosis is involved in the development of abdominal aortic aneurysm (AAA), we explored the pyrocytosis-related hub genes in AAA and conducted a diagnostic model based on the pyrocytosis-related genes score (PRGs). A total of 2 bulk RNA-seq (GSE57691 and GSE47472) datasets and pyrocytosis-related genes were integrated to obtain 24 pyrocytosis-related different expression genes (DEGs). The LASSO Cox regression analysis was conducted to filter out 7 genes and further establish the nomogram signature based on the PRGs that exhibited a good diagnosis value. Weighted gene co-expression network analysis (WGCNA) established 14 gene modules and further identified 6 hub genes which were involved in the regulatory process of pyrocytosis in AAA. At the single cell level, we further identified 3 immune cells were highly associated with the pyrocytosis process in AAA. Finally, the cell-cell communication demonstrated that fibroblasts and endothelial cells and myeloid cells maintained close communications. Here, we identified the dysfunctional expressed pyrocytosis-related genes and immune cells in AAA, which provide a comprehensive understanding of the pathogenesis of AAA.

## INTRODUCTION

Abdominal aortic aneurysm (AAA) is a fatal vascular disease with a focal dilation in the abdominal aorta, which might lead to vascular rupture associated with a mortality rate of 90 to 100% [1–3]. The abdominal ultrasound imaging is the gold standard for AAA diagnosis which presents with dilated aorta diameter over 50% than the normal [4], however, unruptured aneurysms are usually asymptomatic in most AAA patients, and it is very difficult to detect the presence of AAA at an early stage. Although some common risk factors of AAA have been identified, such as smoking, hypertension, family history, male gender and age [5], these are also closely associated with other cardiovascular diseases [6]. Therefore, it is urgent to find some specific diagnosis biomarkers for the early stage of AAA.

Pyroptosis, a recently discovered pro-inflammatory mode of programmed cell death, is mediated by the caspase family and might play a pivotal role in the dysfunction of vessel smooth muscle cells (VSMCs) [7]. Suppression of pyroptosis by Gasdermin D pathways could prevent AAA formation due to inhibiting the pro-inflammatory phenotype in VSMCs [8]. Mounting evidence shows the pyroptosis-related genes share an overlapping pathogenic effect which leads to the chronic inflammation and VSMCs remodeling in AAA, such as the Nod-like receptor family pyrin (NLRP) 3 [9, 10], the interleukin (IL)-1β [11] and caspase family [12]. Recently, inhibiting the activation of NLRP3 inflammasome by the potential inhibitor MCC950 which could prevent the aortic destruction and aneurysm in mice [13]. However, the role of these pyroptosis molecules in AAA is not fully understood. Therefore, it is necessary to explore the application value of pyroptosis in AAA.

Single-cell (sc) RNA sequencing is an experimental approach to investigate the differences in gene expression and cell progression by generating transcriptomic profiles of individual cells [14, 15]. Some researchers performed scRNA sequencing that identified the major cell types, such as VSMCs and fibroblasts and macrophages, might contribute differently to AAA pathogenesis [16]. As these studies were conducted based on small samples and lacked the ability to further explore the potential molecular function. Moreover, the transcription factors (TFs) and downstream signaling pathways which play important roles in AAA were urgently needed to clarify.

Here, we identify the dysfunctional expressed pyrocytosis-related genes and immune cells in AAA, which provide a comprehensively understanding about the pathogenesis of AAA.

## MATERIALS AND METHODS

### Data processing

Two public abdominal aortic aneurysm (AAA) RNA chip datasets, GSE57691 [17] and GSE47472 [18], were obtained from the Gene Expression Omnibus (GEO) database (http://www.ncbi.nlm.nih.gov/geo). Following the standardization, 59 *Homo Sapiens* samples in GSE57691 (10 normal, 49 AAA samples) and 22 *Homo Sapiens* samples in GSE47472 (8 normal and 14 AAA samples) were obtained. Individual genes in the GSE57691 and GSE47472 datasets were annotated by the platform of GPL10558 Illumina HumanHT-12 V4.0 expression beadchip. The gene information of these samples was provided in the Supplementary Table 1.

### Downloading single cell RNA-seq data

Single-cell RNA-Seq data of vascular wall of 2 transplant donors and 4 AAA patients were downloaded from the GEO database (GSE166676). Low-quality data were excluded using Seurat as described above. Relevant gene information of these samples was provided in Supplementary Table 1.

### Downloading pyroptosis-related genes data

The key genes which may involve in the process of pyroptosis were obtained from the Molecular Signatures Database (MSigDB) [19] and the previous pyroptosis-related research [20]. After removing overlapping genes, 52 pyroptosis genes were included (Supplementary Table 2).

### Identification analysis of differentially expressed genes

To identify potential mechanisms of action and related biological characteristics and pathways in AAA, we used “sva” R Package [21] for batch correction to prevent the batch effect from disrupting downstream analyses in GSE57691 and GSE47472. Then the combined data set was standardized by “limma” R Package, and the AAA combined datasets was obtained. Principal component analysis (PCA) was performed on the expression matrix of the datasets in order to remove the batch effect from the AAA combined datasets.

Next, screened with log-fold change (| logFC |) >0.5 and adjusted *P* < 0.05, the differentially expressed genes (DEGs) between the AAA group and the normal group were identified by the “limma” R Package. The | logFC | >0.5 and adjusted *P* < 0.05 were up-regulated genes and the | logFC |<−0.5 and adjusted *P* < 0.05 were down-regulated genes. In order to clarify the expression of pyrogenic-related genes in AAA patients, the “RCircos” R Package [21] was used to demonstrate the localization of these genes in chromosomes. All pyroptosis-related genes expression levels in the samples were presented as a heat map by “pheatmap” R Package. Statistical significance of DEGs in AAA and normal groups was determined by Wilcoxon-test.

### Gene function and pathway enrichment analysis

The gene ontology (GO) and Kyoto Encyclopedia of Genes and Genomes (KEGG) pathway enrichment analysis were performed by the “clusterProfiler” R Package (v4.0) [22] to demonstrate the function and molecular pathways of DEGs [23]. We used the *P*-value < 0.05 and false discovery rate (FDR) <0.05 to determine statistically significant enrichment, and the statistical analyses were conducted by Benjamini-Hochberg procedure (B-H).

### Correlation analysis

In order to further explore the correlation among pyroptosis-related genes, we performed a Spearman correlation analysis at each gene expression level and created a heatmap to visualize the correlation by the “corrplot” R Package. The correlation coefficient and *P*-values were illustrated using “ggplot2” R Package shown as a scatter plot. A *P* value < 0.05 and |correlation coefficient| > 0.3 were considered significantly correlative. The |correlation coefficient| between 0.3 with 0.5 means weak correlation, between 0.5 with 0.8 means moderate correlation, and greater than 0.8 means strong correlation.

### Diagnosis model construction

To obtain diagnostic models for pyroptosis-related genes in AAA combined datasets, the univariate Cox regression was performed with parameter family = “binomial” by using the “glm” R Package. By building a penalty function, which simultaneously sets certain coefficients to zero and compresses some coefficients, the least absolute shrinkage and selection operator (Lasso) technique creates a more refined model. Next, the Lasso regression analysis was performed to screen for novel pyroptosis-related AAA biomarkers. The risk score of pyroptosis was calculated according to the level of gene expression and its related multivariate regression coefficient, the formula is as follows:


$$\text{riskScore}=\sum_{i}Coefficient\;(hub\;gene_{i})\times mRNA\;Expression\;(hub\;gene_{i})$$

The AAA patients were further divided into the high pyroptosis-related gene score (PRGs) group and low PRGs group according to the median value of risk score of pyroptosis. The Nomogram is a graph that shows the functional connection between several independent variables in a rectangular coordinate system using a collection of discontinuous line segments. The “rms” R Package (https://cran.r-project.org/web/packages/rms/) was used to construct the nomogram model [24]. A graphical analytic tool called the Receiver Operating Characteristic (ROC) Curve may be used to choose the best model, remove the second-best model, or establish the optimum threshold within the same model. The composition technique reflects the correlation between sensitivity and specificity, and the ROC curve is a complete indication of continuous variables that represent both sensitivity and specificity [25]. The area under the ROC curve (AUC) of 0.9 to 1.0 is considered excellent, 0.8 to 0.9 is very good, 0.7 to 0.8 is good, 0.6 to 0.7 is sufficient, 0.5 to 0.6 is bad, and less than 0.5 is considered not useful. The “pROC” R Package was used to plot the ROC curves of both pyroptosis-related genes and PRGs with AAA. Then, the calibration curves were used to evaluate the predictive value of the nomogram. Decision Curve Analysis (DCA) is an evaluative clinical prediction model for diagnostic tests and molecular markers [26]. Finally, the “ggDCA” R Package was used to a draw DCA image to evaluate the accuracy and discrimination of the logistic regression model.

### Gene set enrichment analysis

Gene Set Enrichment Analysis (GSEA) is a method utilized to evaluate the distribution pattern of genes within a pre-defined gene set that is rated by their relationship to phenotype in order to ascertain how much each gene contributes to the phenotype of a disease [27]. Accordingly, the AAA Combined Datasets were stratified into either the low-risk (low PRGs score) group or the high-risk (high PRGs score) group, and the DEGs from the low-risk group and the high-risk group were calculated by “limma” R Package, then ranked according to their | logFC | value. The potential biological mechanisms of the DEGs were determined using the “clusterProfiler” R Packages. The number of calculations was 1000 and the number of genes in each gene set was 10 to 500. The *P*-value correction method was Benjamini-Hochberg (BH). The “c2.cp.v7.2.symbols.gmt” gene datasets using GSEA that were downloaded from the Molecular Signatures database (MSigDB; https://www.gsea-msigdb.org/gsea/msigdb/index.jsp) [28], and a *P*-value < 0.05 and FDR <0.05 of screening criteria was considered for significant enrichment.

### Gene set variation analysis

Gene set variation analysis (GSVA) was used to calculate the relative enrichment of gene signatures of matrix of expression from the AAA Combined Datasets based on the reference gene sets of “c2.all.v7.4.symbols.gmt” that were downloaded from the Molecular Signatures database (MSigDB; https://www.gsea-msigdb.org/gsea/msigdb/index.jsp), and the functional differences of enriched pathways in GSVA enrichment analysis results between different risk groups were calculated. A *P* < 0.05 was considered significant.

### Weighted co-expression network analysis

We conducted the weighted co-expression network analysis (WGCNA) by “WGCNA” R Package on AAA patients from the integrated GEO datasets with a standard deviation of expression > 0 for further analysis, excluding outlier data. Different branches of the cluster tree represent different gene modules, different colors represent different modules, and then the module significance is calculated.

Here, WGCNA was used as the input of 5000 absolute median difference genes, the minimum number of module genes was set as 30, softpower was set as the best soft threshold of 8, module combined shear height was set as 0.3, and the minimum distance was set as 0.2 to measure the correlation between high PRGs group and low PRGs group with different modules. The genes in each module were also recorded, and the genes in each module were regarded as module feature genes. After we selected the module of interest based on the correlation value, all genes within the module were identified as expressed genes highly associated with pyroptosis.

### Protein-protein interaction network

Protein-protein interaction network (PPI network) is the interaction of individual proteins, which is involved in biological signal transmission and gene expression regulation in energy metabolism and cell cycle regulation. We used the NetworkAnalyst database (https://www.networkanalyst.ca/), a database searching for interactions between known transcription factors and target genes and proteins and predicted proteins, to construct a transcription factor-target gene interaction network of pyroptosis hub genes, which was visualized by Cytoscape (version 3.8.1) [29].

### Tissue-infiltrating immune cells analysis

CIBERSORTx is a web tool that evaluates the relative proportions of immune cells in tissue using a deconvolution algorithm [30]. The high and low risk group matrix data of the AAA integrated dataset were uploaded to the CIBERSORTx website. Combined with the patient's immune cell characteristic gene matrix, the data with immune cell enrichment scores greater than zero were screened, and the specific results of immune cell infiltration abundance matrix were finally obtained and displayed [31]. The contents of different immune cells in samples from the high and low risk groups of the AAA integrated dataset are presented by heatmaps and boxplots. The correlation between immune cells and pyroptosis in different risk groups was calculated by Spearman correlation analysis, and the “pheatmap” R Package was used to create a correlation heat map.

### Construction of molecular subtypes based on pyroptosis-related genes

Consensus clustering is a consensus clustering algorithm based on resampling, which determines the number of members and subgroups and verifies the rationality of clustering [32]. Unsupervised clustering analysis was performed using the “ConsensusClusterPlus” R Package based on the AAA Combined datasets [33]. Consensus clustering method was used to identify different disease subtypes of AAA patients based on pyroptosis-related genes. In addition, the analysis included 1000 iterations to ensure the stability of the classification.

### ESTIMATE immunoactivity analysis

In this study, we used the “ESTIMATE” R Package to evaluate the different sample immune activities based on the AAA integrated dataset [34]. ESTIMATE (Estimation of STromal and Immune cells in MAlignant Tumour tissues using Expression data) calculates the proportion or abundance of immune cells, stromal cells and tumor cells related to the tumor microenvironment. We calculated the immune and stromal scores of all patients based on the ESTIMATE algorithm, and then compared the differences in the immune scores of AAA patients between the high and low risk groups.

### Single cell RNA-seq data processing

The single-cell dataset GSE166676 used the count data of original unique molecular identifier (UMI). Data preprocessing, quality control, normalization, dimensionality reduction and clustering were processed using the “Seurat” R package (https://cran.r-project.org/web/packages/Seurat/index.html). The quality control (QC) process of scRNA-seq data was performed using “Seurat”. The gene expressed in <3 cells, the cell expressing <200 genes and the sample having <6000 nFeature RNA would be removed. Also, cells expressing >25% of genes derived from the mitochondrial genome had been eliminated due to a risk of low quality. The “harmony” R Package is used to integrate data across samples. Single cell group nomenclature was performed using signature genes from published literature and manual annotation.

### Cell–cell interactions

CellphoneDB was employed to explore the cell–cell interactions between AAA patients and normal of single cell dataset GSE166676 [35]. The expression matrix and annotation files were constructed and imported into CellphoneDB software. Finally, the communication molecules and the communication strength and significance of the communication molecules were obtained.

### Single-cell regulatory network inference and clustering analysis

Single-cell regulatory network inference and clustering (SCENIC) analysis was performed by cell subtype-specific transcription factors using the “SCENIC” R Package [36]. By constructing the expression matrix and annotation files, SCENIC software was imported to construct the co-expression network and the transcription factor-target gene network, and the activity of transcription factor Regulons was calculated.

### Statistical analysis

All data processing and analysis were performed using R software (version 3.6.1). When comparing continuous variables between two groups, the independent Student’s *t*-test for normally distributed variables, and the Mann-Whitney *U* test for non-normally distributed variables. Chi-square test or Fisher exact test was used to compare and analyze the statistical significance between the two groups of categorical variables. The drawing of the nomogram is based on the rms R Package. *P* < 0.05 was considered statistically significant.

### Availability of data and materials

All data generated or analyzed in this study are included in this article. Other data that are relevant to this article are available from the corresponding author upon reasonable request.

## RESULTS

The flow chart of our study is shown in Supplementary Figure 1.

### To remove the batch effect of AAA combined datasets

First, the “sva” R package was used to normalize and remove batch effects from the abdominal aortic aneurysm (AAA) Datasets GSE5296 and GSE47681 to obtain AAA Combined Datasets. Distribution boxplots were used to compare the total gene expression values before (Supplementary Figure 2A) and after (Supplementary Figure 2B) data processing, which confirmed that gene expression values were normalized between samples of the dataset. Next, principal component analysis (PCA) was used to compare global gene expression signatures between before (Supplementary Figure 2C) and after removal of batch effects (Supplementary Figure 2D), which suggests that the batch effect of samples in AAA Combined Datasets was eliminated.

### Differentially expressed genes and enrichment analysis of AAA

**Figure 1:**
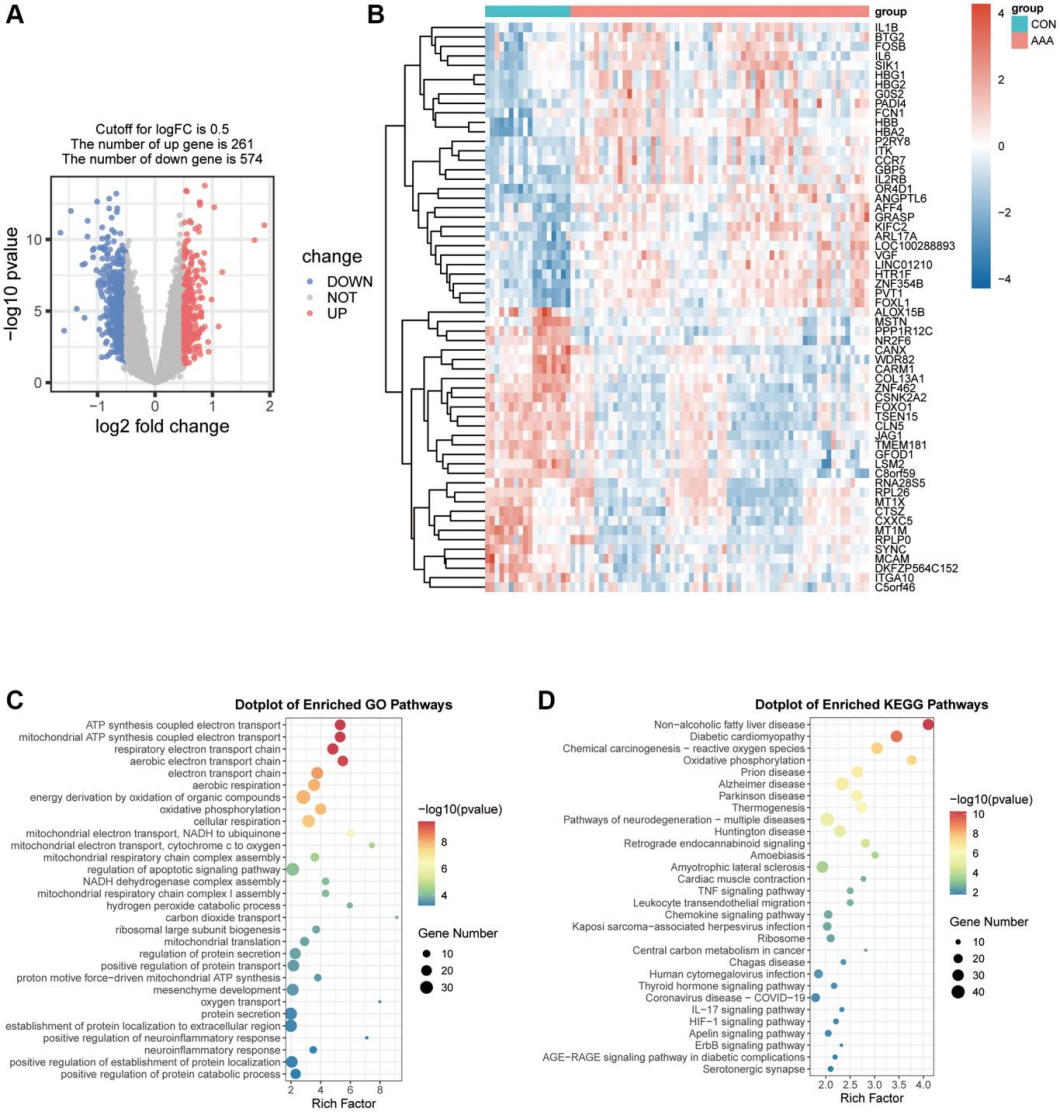
**Visualization of the DEGs in AAA Combined Datasets.** (**A**) Volcano map of differential expression gene analysis of the difference between the AAA group and the CON group. Red represents significantly up-regulated genes and blue represents significantly down-regulated genes. (**B**) Thermogram of the expression values of 30 up-regulated and 30 down-regulated genes in the AAA group compared with the CON group. Red means high gene expression, blue means low gene expression. (**C**, **D**) Bubble map of differential gene GO functional enrichment analysis (**C**) and KEGG pathway enrichment (**D**). Bubbles represent the number of enriched genes, the larger the enrichment genes, the darker the color, the more significant the *P*-value, and the darker the color, the more significant the difference. Abbreviations: AAA: abdominal aortic aneurysm; CON: control; GO: Gene Ontology; KEGG: Kyoto Encyclopedia of Genes and Genomes.

Total 835 genes were obtained according to the filtering criteria (| logFC | > 0.5 and adjusted *p* < 0.05), and 261 genes were significantly upregulated and 574 genes were significantly downregulated (Supplementary Table 3), and these genes were displayed by a volcano graph (Figure 1A). The most significant 30 up-regulated genes and 30 down-regulated genes were selected and used “pheatmap” R package to display as heatmap (Figure 1B).

We further analyzed the relationship between DEGs and AAA from the aspects of biological processes, molecular functions, cell components and biological pathways. The results of GO analysis showed DEGs were mainly enriched in the energy production process, such as the ATP synthesis coupled electron transport, mitochondrial ATP synthesis coupled electron transport and positive regulation of defense response respiratory electron transport chain (Figure 1C). The KEGG pathway was enriched for non-alcoholic fatty liver disease, oxidative phosphorylation pathway and chemical carcinogenesis-reactive oxygen species (Figure 1D).

### Differential expression and correlation analysis of pyroptosis-related genes in AAA patients

**Figure 2:**
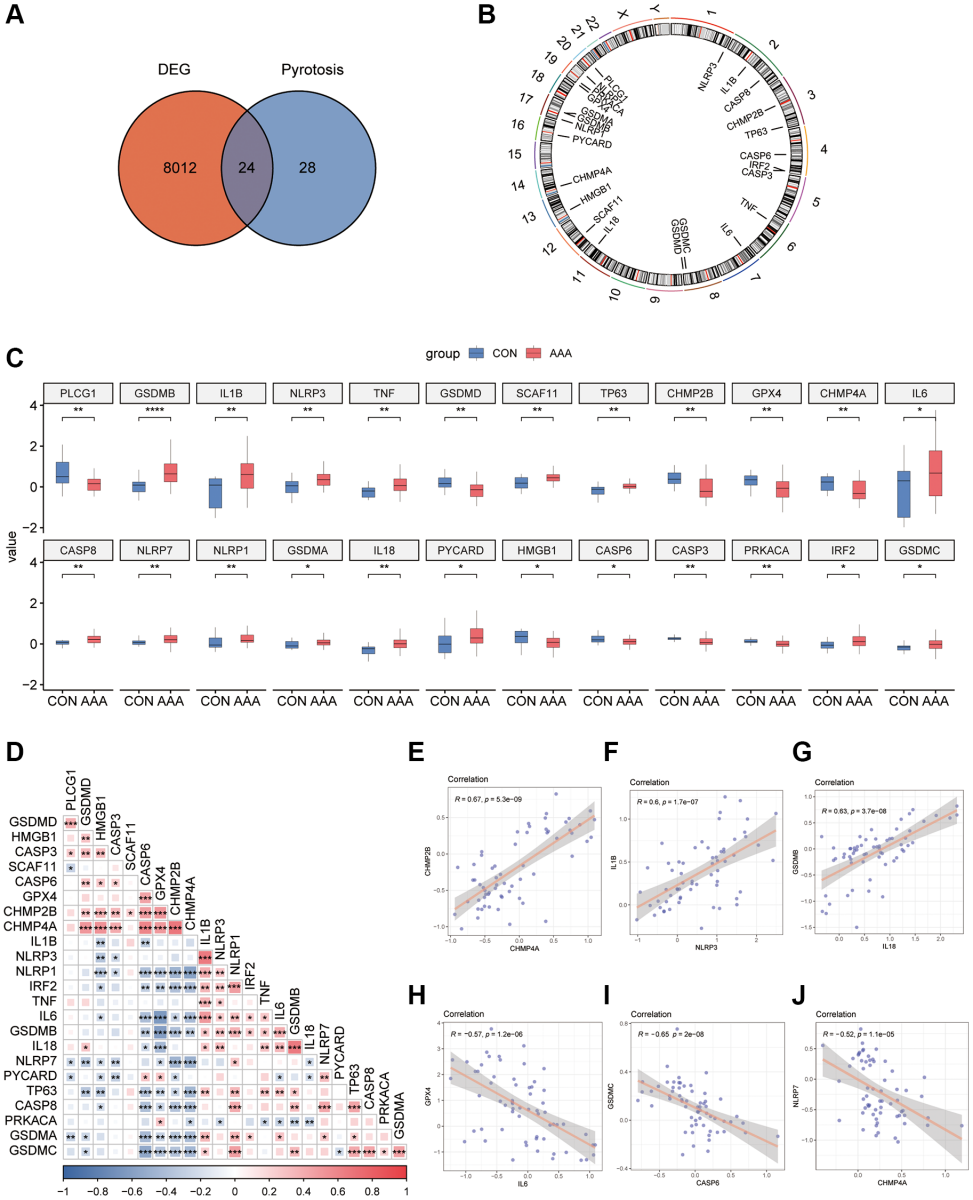
**Pyroptosis-related gene expression in AAA Combined Datasets.** (**A**) Venn diagram of pyroptosis-related genes and DEGs. (**B**) Chromosomal mapping of pyroptosis-related genes from overlapping genes. (**C**) Box plot of pyroptosis-related genes expression in the AAA and Con groups. (**D**) Heatmap of pyroptosis-related in combined GEO Datasets. ^*^P < 0.05, *P* < 0.01, ^***^P < 0.001, the red represents positive correlation, blue represents the negative correlation. Scatter plot of the correlation between CHMP2B and CHMP4A (**E**), IL-1β and NLRP3 (**F**), IL-18 and GSDMB (**G**), IL-6 and GPX4 (**H**), CASP6 and GSDMC (**I**), and CHMP4A and NLRP7 (**J**) in AAA Combined Datasets. Abbreviations: AAA: Abdominal aortic aneurysm; CON: Control; DEG: differential gene expression.

To demonstrate the role of pyroptosis in the pathogenesis of AAA, we took the intersection of DEGs from the AAA combined database and pyroptosis-related genes to obtain 24 overlapping genes (Figure 2A), and the location was further annotated using the “RCircos” R package. The classic pyroptosis regulatory genes, such as the NLRP3 is located on chromosome 1, IL-1β and CASP8 on chromosome 2, and TNF on chromosome 6 (Figure 2B).

The results of the specific differences of 24 overlapping genes were shown by box plots, among them 15 genes, such as GSDMB and IL-1β were significantly up-regulated, and 9 genes such as PLCG1 and GSDMD were significantly down-regulated in AAA group (Figure 2C).

To explore the expression relationships between pyroptosis-related genes, the expression matrices from AAA Combined Datasets were used to make correlation analysis and the results were shown by heatmaps (Figure 2D). The results showed that there were positive correlations between a variety of pyroptosis-related genes, such as CASP6, GPX4, CHMP2B and TNF, IL-6, IL-18 (*P* < 0.05, r > 0). In contrast, the NLRP1 was negatively correlated with CASP6, GPX4, and CHMP2B (*P* < 0.05, r < 0) and the GSDMC was negatively correlated with CASP6, GPX4, CHMP2B and CHMP4A (*P* < 0.05, r < 0). We also observed a positive correlation between expression of CHMP2B and CHMP4A (r = 0.67, *P* < 0.05), IL-1β and NLRP3 (r = 0.6, *P* < 0.05), IL-18 and GSDMB (r = 0.63, *P* < 0.05) in the AAA Combined Datasets (Figure 2E–2G); IL-6 and GPX4 (r = −0.57, *P* < 0.05), CASP6 and GSDMC (r = −0.65, *P* < 0.05), CHMP4A and NLRP7 (r = −0.52, *P* < 0.05) were negatively correlated in the AAA Combined Datasets (Figure 2H–2J).

### Construction of a nomogram of pyroptosis-related genes to predict AAA

To determine the diagnostic value of pyroptosis-related genes in the AAA Combined Datasets, the LASSO logistic regression was performed based on the 24 overlapping DEGs for gene screening. Then cross-validation was performed (Supplementary Figure 3A), and the goal was to select model genes corresponding to λ with as few variable characteristics and as little error as possible. We identified 7 critical genes as important biomarkers of AAA based on the 24 overlapping DEGs (Supplementary Figure 3A, 3B). These genes including GSDMB, GSDMD, NLRP3, PLCG1, TNF, IL-1β and SCAF11 were significantly associated with the incidence of AAA, and the AUC according to the ROC curve was from 0.0.729 to 0.818 (Supplementary Figure 3C–3I), which suggests these genes had a highly predictive value.

The pyroptosis-related genes score (PRGs) was calculated according to the scores corresponding to the gene expression value, and we combined the PRGs to construct a nomogram model to diagnosis for AAA (Supplementary Figure 3J). The nomogram model has the highest diagnostic accuracy with an AUC value 0.994 (Supplementary Figure 3K). A calibration plot was used to visualize the performance of the nomogram, which confirmed the performance of our model (Supplementary Figure 3L). Then, both the decision curve analysis (DCA) and the clinical impact curve (CIC) were used to confirm the findings (Supplementary Figure 3M, 3N).

### Identification of key pyroptosis-related gene modules via WGCNA

**Figure 3:**
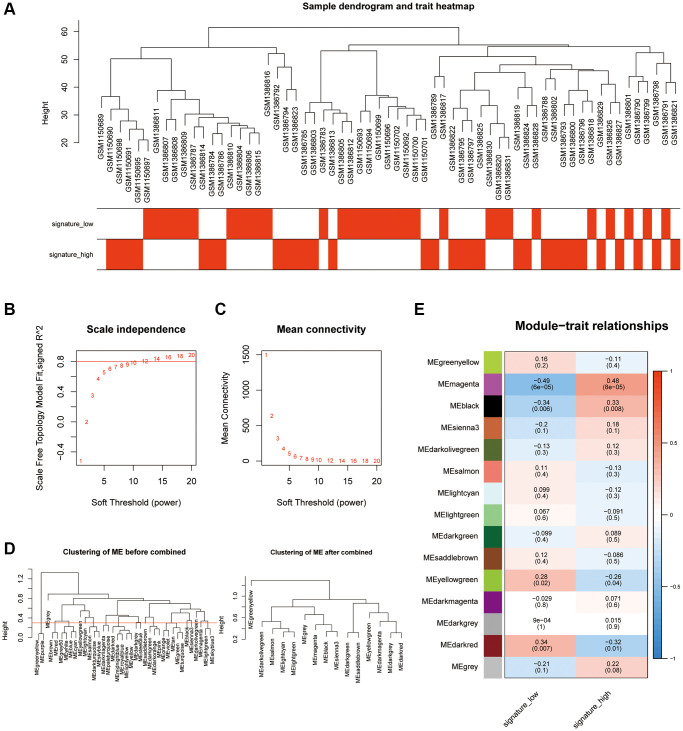
**Analysis of the weighted co-expression network in high and low PRGs.** (**A**) Presentation of sample grouping results in high and low PRGs from the AAA combined datasets. (**B**, **C**) Sample module screening threshold scale-free networks in high and low PRGs from the AAA combined datasets displaying scale-free topological fit index (**B**) and average connectivity degree (**C**). (**D**) Presentation of module aggregation in high and low PRGs from the AAA combined datasets. (**E**) Presentation of correlation analysis between cluster modules and different groups in high and low PRGs from the AAA combined datasets. Abbreviations: WGCNA: weighted correlation network analysis; AAA: abdominal aortic aneurysm.

To identify the key pyroptosis-related genes associated with the AAA patients, co-expression network analysis was performed via WGCNA using the AAA combined database. We divided AAA patients into a high PRGs group and a low PRGs group based on the previously constructed prediction model. First, we clustered and labeled the high PRGs group and low PRGs group, and determined the optimal number of modules by setting a screening criterion of 0.8 based on the scale-free topology fitting index and the average connectivity degree (Figure 3A–3C). Then, we set the module merging shear height to 0.25, and merge the modules whose module merging shear height is lower than 0.25 (Figure 3D). Finally, based on the expression patterns of module genes and the grouping information of different groups, we obtained correlations between 14 modules and groups with high or low risk of PRGs (Figure 3E).

### Functional enrichment analysis of pyroptosis module and identification of the hub pyroptosis genes

Next, a total of 14 modules (excluding useless modules: MEgrey) which met differential expression criteria (*P* < 0.05, | COR | ≥ 0.3) including the black (| COR | = 0.33, *P* < 0.01), magenta (| COR | = 0.48, *P* < 0.01) and dark red (| COR | = 0.34, *P* < 0.01) were selected for subsequent analysis (Supplementary Figure 4A). The functional analysis of hub genes was also performed and found that the Magenta module genes were mainly enriched in biological processes such as neutrophil migration (Supplementary Figure 4B), the black module genes were mainly enriched in biological processes such as DNA binding and MiRNA metabolism (Supplementary Figure 4C) and the dark red module genes were mainly enriched in biological processes such as mitochondrial respiration (Supplementary Figure 4D).

Next, total 6 overlapping genes between the differential expression modulars and pyroptosis-related genes were obtained as hub genes, including CHMP4B, CYCS, GSDMB, GZMB, PLCG1 and IL-6 (Supplementary Figure 4E). The transcriptional factors (TF)-gene interaction network was constructed using the NetworkAnalyst website [37], and the results showed these hub genes were mainly regulated by some target TFs, such as the TEAD1, MAFK, and FOXA3, etc. (Supplementary Figure 4F).

### Functional and enrichment analysis in DEGs from high PRGs group and low PRGs group

The GSVA was performed to determine the difference of biological function between the high PRGs group and low PRGs group. After obtaining the pathway activity matrix, the “limma” R package was used for differential analysis (Supplementary Table 4), and the top 30 pathways in each group were selected and displayed as a heatmap (Supplementary Figure 5A) and a bar graph (Supplementary Figure 5B). In addition, the results of GSEA showed that intestinal immune network for IgA production, cell adhesion molecules cams, complement and coagulation cascades, cytokine-cytokine receptor interaction, oxidative phosphorylation and spliceosome pathways were enriched in the high PRGs group compared with the low PRGs group (Supplementary Table 5 and Supplementary Figure 5C–5H).

### The differential characteristics in immune infiltration between the high PRGs group and low PRGs group

**Figure 4:**
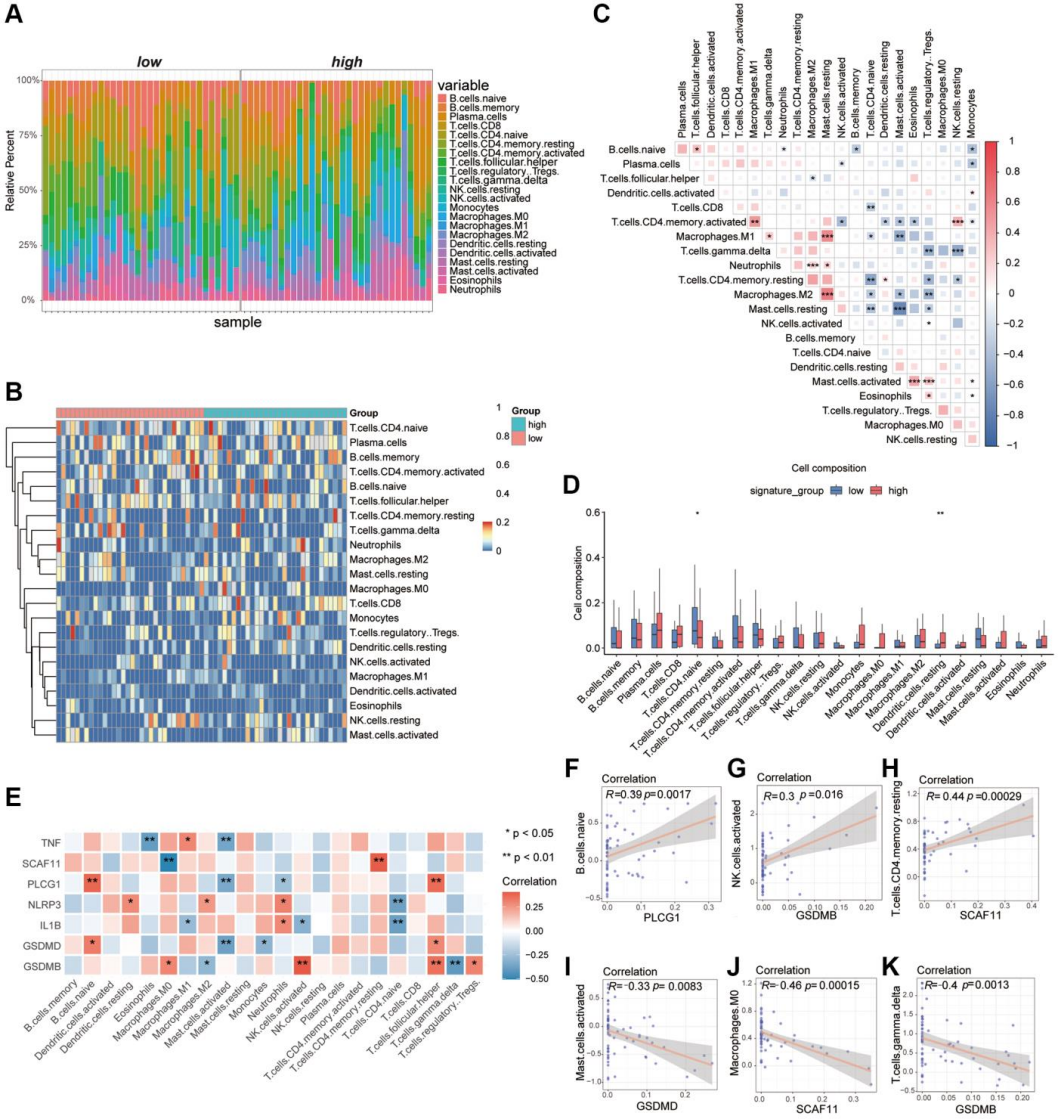
**Analysis of differences in immune characteristics between high and low PRGs groups based on the AAA combined datasets.** (**A**) Bar graph of abundance of immune cell infiltration between high and low PRGs groups. (**B**) Heatmap of the abundance of immune cell infiltration between the high and low PRGs groups. Red representing high cell abundance and blue representing low cell abundance. (**C**) Correlation heatmap of infiltration abundance of different immune cells. Red represents positive correlation and blue represents negative correlation. (**D**) Box plot of immune cell infiltration abundance between the high and low PRGs groups. Red represents the high PRGs group and blue represents the low PRGs group. (**E**) Heatmap of correlation between pyroptosis biomarker and infiltration abundance of different immune cells. Red represents positive correlation and blue represents negative correlation. (**F**–**K**) Scatter plot of correlation between pyroptosis biomarker and abundance of different immune cell infiltration. PLCG1 was significantly correlated with B.c.naive (**F**). There was a significant positive correlation between GSDMB and NK.cells.activated (**G**). The SCAF11 was significantly positively related to T.cell.CD4.Memory. Resting (**H**). The GSDMD was negatively correlated with Mast.cells.activaetd (**I**). The SCAF11 was negatively correlated with Macrophage.M0 (**J**). There was a significant negative correlation between GSDMB and T.cal.GMMA.delta (**K**). ^*^P < 0.05, ^**^P < 0.01, ^***^P < 0.001. Abbreviation: PCA: Principal Component Analysis.

Recent researches showed the excess immune cells infiltration was involved in the pathogenesis of AAA [38, 39]. In order to demonstrate the difference of immune-mediated infiltration between the high PRGs group and low PRGs group, the CIBERSORTx algorithm was used to calculate the infiltration abundance of immune cells in two group samples (Supplementary Table 6). Here, a total of 22 types of immune cells in the samples were identified and the results of immune infiltration abundance were displayed by bar graphs (Figure 4A) and heatmaps (Figure 4B). In addition, there is also a certain correlation between different immune cells (Figure 4C). Besides, the abundance of DC.resting was significantly up-regulated in the high PRGs group, and the T.cell.CD4.naive was significantly down-regulated (Figure 4D).

We had previously identified 7 critical genes as important biomarkers of AAA from the 24 overlapping pyroptosis-related DEGs. Here, we also found a certain correlation between the abundance of immune-mediated infiltration with these AAA biomarkers which were displayed as heatmaps (Figure 4E), such as the PLCG1 was a positive correlation with the B.cell.naive (r > 0, *P* < 0.05), the GSDMB was positive correlation with the NK.cells.activated (r > 0, *P* < 0.05) and the SCAF11 was positive correlation with the T.cell.CD4.memory.resting (r > 0, *P* < 0.05). In contrast, the GSDMD was negative correlation with the Mast.cells.activaetd (r < 0, *P* < 0.05), the SCAF11 was negative correlation with the Macrophage.M0 (r < 0, *P* < 0.05) and the GSDMB was negative correlation with the T.cell.gmma.delta (r < 0, *P* < 0.05) (Figure 4F–4K).

### Construction of the specific pyroptosis-associated AAA subtypes

We used the “ConsensusClusterPlus” R package to identify the different AAA subtypes based on the 24 overlapping pyroptosis-related DEGs. Results of consensus cluster analysis were displayed by the cumulative distribution function (CDF) plot and the AUC (Supplementary Figure 6A, 6B), and we found the optimal k value is 2 as the number of clusters for unsupervised clustering (Supplementary Figure 6C–6E). Then, 2 AAA disease subtypes (cluster1 and cluster2) were performed for further analysis, which had 37 samples and 26 samples, respectively. The principal component analysis (PCA) of the expression matrices from the 2 AAA disease subtypes was conducted and found the 2 disease subtypes were significantly separate (Supplementary Figure 6F).

### The differences of immune infiltration in pyroptosis-related AAA subtypes

The expression of 24 overlapping pyroptosis-related DEGs from 2 AAA subtypes based on the AAA Combined database was displayed by “pheatmap” R Package, and found that 11 genes had remarkable difference in expression (Supplementary Figure 7A). The GSDMB, IL-1β, NLRP3, IL-6, NLRP1, IRF2 and GSDMC were upregulated in the cluster2, and SCAF11, GPX4, PYCARD and CASP6 were downregulated in the cluster2. The results of immunocompetence analysis showed that the cluster2 had a higher immune score, stronger interferon response, more costimulatory molecule expression and HLA compared with the cluster1, which suggests that the AAA disease subtypes had different characteristics of immune infiltration (Supplementary Figure 7B).

### Single-cell quality control and identification of cell subsets

To further explore which cells were involved in the development of AAA based on the pyroptosis-related genes, the single-cell dataset GSE166676 was downloaded. After filtration based on the gene features, gene counts and fraction of mitochondrial genes, a total of 13716 single cells were analyzed (Supplementary Figure 8A–8C). Thereafter, we identified 2000 genes with high variability for PCA dimensions reduction (Supplementary Figure 8D). The uniform manifold approximation and projection (UMAP) was conducted following gene expression normalization to reduce the dimensionality (Supplementary Figure 8E–8G).

**Figure 5:**
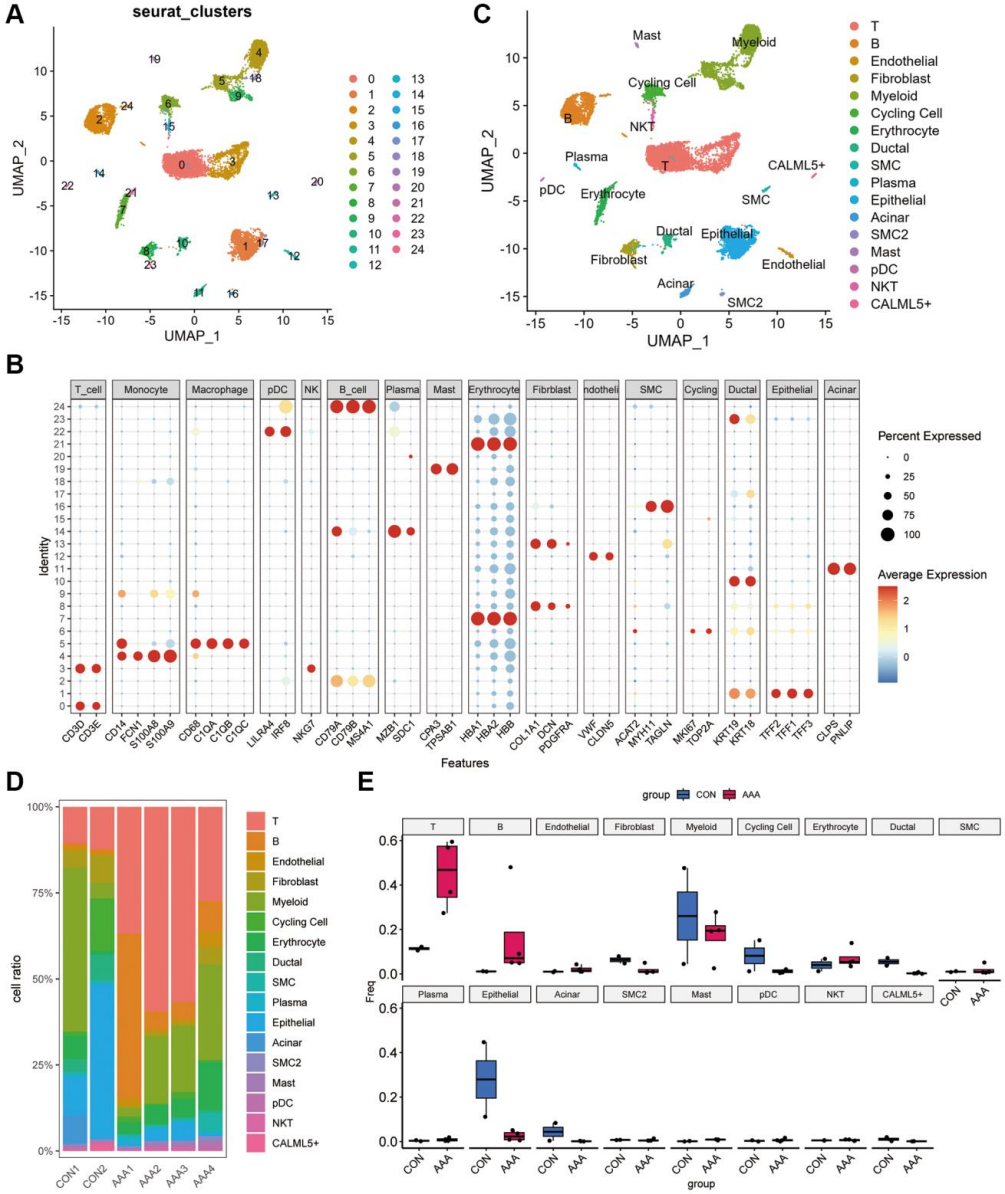
**Dimension reduction clustering in GSE166676 databases.** (**A**) When resolution = 0.5, the GSE166676 dataset was divided into 24 cell populations. (**B**) The expression of specific markers of different cell types in 24 cell clusters. The color depth represents the gene expression level, and the size of the dot represents the positive proportion of the gene in the cell. (**C**) The 24 cell populations were divided into 15 cell types based on cell type-specific markers. (**D**) Bar graph of cell proportions in AAA group and control group. (**E**) Box plots of cell proportions in AAA group and control group. Abbreviation: AAA: Thoracic aortic aneurysm.

As shown in Figure 5A, we clustered the cells into 24 clusters based on the “FindNeighbors” and “FindClusters” function in “Seurat” R Package. According to the marker gene, we identified 16 cell types including the T cells, B cells, endothelial cells, fibroblasts, myeloid cells, plasmacytoid dendritic cells cells (pDC), cycling cells, erythrocyte, ductal cells, smooth muscle cells (SMC), plasma cells, epithelial cells, acinar cells, mast cells, natural killer T cells and CALML5+ cells (Figure 5B, 5C). The proportion of cell subclusters in each patient revealed that the AAA group had an increased proportion of T and B cells and a decreased proportion of epithelial cells compared with the control group, which suggests that AAA had excess immune cell infiltrations (Figure 5D, 5E).

### Identification and enrichment analysis of T lymphocyte subclusters in AAA

Because the proportion of T lymphocyte, myeloid cells and B lymphocyte was significantly different between the AAA and CON groups, the T lymphocytes, myeloid cells and B lymphocyte subclusters were further identified based on the GSE166676 dataset. A total of 6 T lymphocyte subclusters were identified by cell-specific markers (Supplementary Figure 9A, 9B), and the hallmark genes of different subtypes had different gene expression characteristics (Supplementary Figure 9C). The results are shown as bar graphs and box graphs (Supplementary Figure 9D, 9E). We found that the proportion of CD8+T lymphocytes was significantly increased in AAA group than that in CON group but the proportion of CD4_JUN and NK cells were significantly reduced, and the CD8+T lymphocytes had the highest pyroptosis score among 6 T lymphocyte subclusters (Supplementary Figure 9F, 9G).

To further explore the functional characteristics of T lymphocyte subclusters in AAA, the GSVA analysis was performed based on the GSE166676 dataset. Here, we found that the NK_NKG7 and NK_GNLY cells were enriched in a variety of metabolic pathways, such as histidine and tryptophan metabolism, the CD4_JUN cells were enriched in protein trafficking and autophagy pathways, the CD8+T cells were enriched in glutathione metabolism and mucopolysaccharide synthesis pathways (Supplementary Figure 10A). The results of functional analysis showed that AAA group had increased activity in mucopolysaccharide degradation and decreased activity of tyrosine metabolism compared with the CON group (Supplementary Figure 10B).

Single-cell regulatory network inference and clustering (SCENIC) was used to predict transcription factors (TFs) and corresponding target genes [40]. The SCENIC analysis (Supplementary Figure 10C) point TCF7 and LEF1 acted as the key TF to regulate CD4_CCR7, TGIF1 and FOSB was key to CD4_JUN, LEF1 and RAD2 showed high gene regulatory activity in CD4_SESN3, NFIL3 and MYBL1 was key to CD8+T, TBX21 was key to NK_GNLY and ZNF143 was key to NK_GNG7 (Supplementary Figure 10D–10I).

### Identification and enrichment analysis of myeloid cells subclusters in AAA

A total of 4 myeloid cell subclusters were identified by cell-specific markers (Supplementary Figure 11A, 11B). The results of the abundance of the most significant hallmark genes of different subtypes were shown in Supplementary Figure 11C. Here, we found that the proportion of monocytes, Macro_ZNF331, was significantly increased in the AAA group than in Con group (Supplementary Figure 11D, 11E). In addition, the expression level of pyroptosis-related genes was also elevated in the myeloid cell subclusters, such as Macro_ZNF331 showed higher expression of CHMP2B and HMGB1, Macro_CXCL3 highly expressed IL1B and NLRP3, Macro_APOE highly expressed GPX4 and CHMP4A and PYCARD was highly expressed in monocytes. The Macro_CXCL3 was found to have a higher pyroptosis score than the others (Supplementary Figure 11F, 11G).

The GSVA showed that Macro_CXCL3 was enriched in extracellular matrix interaction pathways, the Monocytes, Macro_CXCL3 and Macro_APOE were enriched in linoleic acid metabolism and taurine metabolism pathways (Supplementary Figure 12A). Compared with CON group, the activities of cytochrome P450 and DNA sensing pathways were increased, and the activities of sphingosine-lipid metabolism and nitrogen metabolism pathways were decreased in AAA group (Supplementary Figure 12B).

Our SCENIC analysis found that Macro_APOE had highly activity of MAF and ATF5, Macro_ZNF331 had highly activity of STAT4 and JUNB, Monocytes showed highly activity of RARA and STAT2 (Supplementary Figure 12C). We also found that the specific differences in TF between each cell subcluster, such as Monocytes specifically expressed STAT2, Macro_APOE specifically expressed MAF, Macro_CXCL3 specifically expressed NFKB1 and NFKB2 and Macro_ZNF331 specifically expresses STAT3 (Supplementary Figure 12D–12G).

### Identification and enrichment analysis of B lymphocyte subclusters in AAA

The proportion of B lymphocytes was also increased in AAA, and we identified 5 subclusters by cell-specific markers (Supplementary Figure 13A). As shown in Supplementary Figure 13B, MZB1 was expressed in plasma cells, MS4A1 in B cells, IL-4R in naive B cells, RNU12 in Memeory_B_TNFRSF13B, TNFRSF13B in Memeory_B_TNFRSF13B, FOS in B_FOS. The results of the most prominent signature genes in each subcluster were shown as a bubble diagram (Supplementary Figure 13C). In addition, we found that the proportion of plasma cells were decreased and the Naïve B cells were increased in AAA group compared with CON group (Supplementary Figure 13D, 13E). For the expression level of pyroptosis-related genes, we found that Memeory_B_RNU12 highly expressed CASP8, Naïve B highly expressed HMGB1 and CHMP4A, and plasma cells highly expressed GPX4, CASP3 and CHMP2B (Supplementary Figure 13F). The Naïve B had the higher PRGs than the others (Supplementary Figure 13G).

The GSVA showed that the plasma cells were enriched in protein transport and polysaccharide synthesis pathways, while Memory_B_RNU12 and Memory_B_TNFRSF13B cells were enriched in ribosome and leukocyte migration pathways (Supplementary Figure 14A). Compared with CON group, the activity of base excision repair and N-polysaccharide synthesis was increased in AAA group, and the activity of O-polysaccharide metabolism and drug metabolism was decreased (Supplementary Figure 14B).

The activity of different TFs was analyzed by SCENIC, such as CREB3 and CREB3L2 were increased in plasma cells, and the JUNB and STAT3 were increased in B_FOS FOSB cells (Supplementary Figure 14C). The most specific TF for each cell subclusters were shown in Supplementary Figure 14D–14H.

### Intercellular communication analysis in AAA

**Figure 6:**
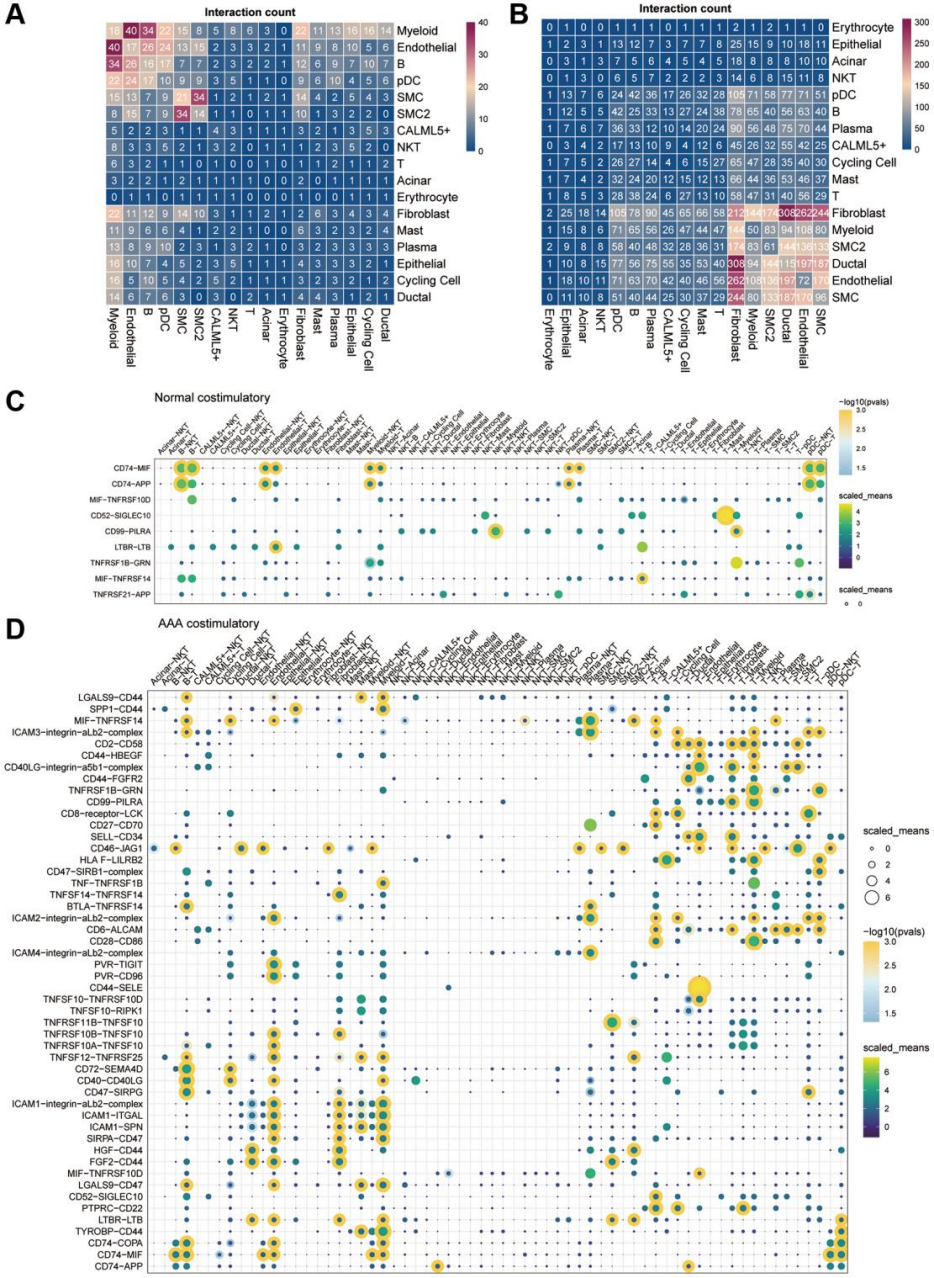
**Cell-cell communication in the GSE166676 single-cell dataset.** (**A**, **B**) Heatmap of cell-cell communication between different cell types in the control group (**A**) and the AAA group (**B**) in the GSE166676 single-cell dataset. (**C**, **D**) Interaction of costimulatory molecules between different cells in the control group (**C**) and AAA group (**D**). The color of the dot represents the communication strength, and the size of the circle represents the significance of the strong *p*-value. Abbreviations: AAA: abdominal aortic aneurysm; CON: Control.

To determine the potential mechanism of intercellular communication between different cells in AAA, the total number of interactions between the AAA group and CON group of the datasets was constructed by CellphoneDB v2.0 (Figure 6A, 6B). Compared with the CON group, intercellular communication between fibroblasts, endothelial cells and myeloid cells was significantly enhanced in the AAA group.

**Figure 7:**
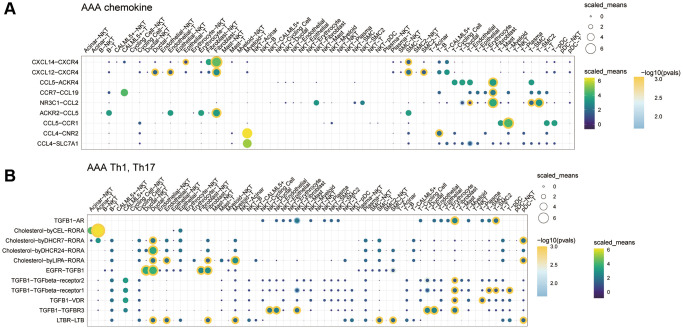
**The role of cytokine communication between different cell types in the GSE166676 single-cell dataset.** (**A**) The role of chemokines between different cells in the AAA group. (**B**) The role of Th cytokines between different cells in the AAA. Abbreviations: AAA: abdominal aortic aneurysm; CON: Control.

We further analyzed the communication molecules between different cells. Among the costimulatory family molecules, communication molecules such as CD74-MIF and CD74-APP were predominated in the CON group, while CD40LG-integin and CD28-CD86 were increased in the AAA group (Figure 6C, 6D). In addition, the chemokines CXCL14-CXCR4, CXCL12-CXCR4, T cell factors TGFB1-AR, EGFR-TGFB1 and TGFB1-TGFBR3 were increased in the AAA group than that in the CON group (Figure 7A, 7B).

## DISCUSSION

Abdominal aortic aneurysms (AAA), also called the “silent killer” as they grow without symptoms until the final rupture. Once an aneurysm ruptures, the mortality rate could almost reach 100%. Until now, no pharmacological treatment has been implemented to prevent the formation of AAA or to cure the disease [41–43]. As unruptured aneurysms are usually asymptomatic in most AAA patients which results in it being almost impossible to detect the presence of AAA at early stage.

Pyroptosis was defined as a gasdermin-mediated pro-inflammatory programmed cell death process, which is different from apoptosis (a noninflammatory program of cell death) [44–46]. Some studies recently demonstrated that the pyroptosis in the aortic wall contributes to the development of AAA through promoting the chronic inflammatory manner [47, 48]. However, the molecular mechanisms and their diagnostic value of pyroptosis-related genes in AAA remain unclear, and it is urgent to identify the pyroptosis-related hub genes.

In the present study, we obtained the DEGs between AAA and normal samples from the GEO database. After combining with pyrocytosis-related genes from the MSigDB, we identified 24 differential expressed pyrocytosis-related genes and calculated the PRGs in AAA. The WGCNA were performed to identify 6 hub genes including the CHMP4B, CYCS, GSDMB, GZMB, PLCG1 and IL-6, which related pyrocytosis process in AAA. Meanwhile, we found the immune cells infiltration was significantly increased in AAA than normal group by the CIBERSORTx evaluation. Finally, the publicly available scRNA-Seq datasets derived from AAA patients were collected and performed the GSVA to elucidate the underlying molecular pathways in AAA.

Then, we identified 24 pyroptosis-related genes that were strongly related to AAA based on two existing AAA datasets and a pyroptosis database. In addition, we firstly constructed a diagnostic model based on the pyroptosis-related gene score (PRGs) and identified 6 hub genes of pyroptosis regulatory in AAA. In addition, the PRGs was calculated according to the scores corresponding to the gene expression value, and we combined the PRGs to construct a nomogram model to diagnosis for AAA. Depends on the specific database, the nomogram model had a relatively high diagnostic accuracy (AUC = 0.994). However, as the specific model of single data set would cause overfitting and led to a false accuracy, this diagnostic nomogram model should be also confirmed by the other database in the future. Finally, the subsets of T lymphocytes, myeloid cells and B lymphocytes, and their characteristics of immunity and potential signaling pathways in AAA were also analyzed, respectively.

Here, we screened out the hub genes by the WGCNA from the high PRGs and low PRGs groups. Total 6 hub genes were obtained from the module, including the CHMP4B, CYCS, GSDMB, GZMB, PLCG1 and IL-6. GSDMB, a unique member of the gasdermin family, which has the inability to form the pores in the cell membrane, and recently confirmed to be associated with immune diseases in humans. Rana et al., [49] identified the expression of GSDMB is significantly increased in the inflammatory bowel disease patients, and further demonstrated the GSDMB-dependent pyroptosis in activated epithelial cells is the key mechanism of pathogenesis. According to these results, we speculated that the GSDMB would have an injury effect in VSMCs which could promote the development of AAA. However, the exact molecular mechanism of GSDMB remains to be confirmed by future experiments. Another hub gene, GZMB, a protease which exocytosed from T cells and endocytosed through a receptor-dependent mechanism into the target cells, had a close relationship with vascular cell death [50, 51]. As GZMB could induced SMC death was through apoptosis, but its effect on AAA never been explored. So, our results provide a novel possible treatment target for AAA.

Strong evidence has been accumulated suggesting that AAA is an autoimmune specific-antigen driven disease and that autoimmunity might be responsible for the pathogenesis of AAA. Next, we identified 16 cell clusters from a public sc-RNA sequence database, including the T cells, B cells, endothelial cells, fibroblast, myeloid cells, pDC, cycling cells, erythrocyte, ductal cells, SMC, plasma cells, epithelial cells, acinar cells, mast cells, natural killer cells and CALML5+ cells. Although some researchers had revealed the genetic characteristics at the single-cell level in AAA, the PRGs in different immune cells had never been clarified. Here, we found the CD8^+^ T cells, Macro_CXCL3 and Naïve B cells had the higher PRGs than others, which suggests these immune cells subsets might mainly depend on regulatory of pyroptosis to promote the development of AAA.

Meanwhile, the potential signaling pathways and TFs were also identified by the GSVA and SCENIC analysis, respectively. Recent studies have established the mechanisms of action of T cells, myeloid cells and B cells in tumor microenvironment [5, 52–54]. Although the pathogenic molecular mechanism in AAA is still lacking, we identified the potential signaling pathways by GSVA, such as T lymphocytes were involved in the glycosaminoglycan degradation and aldosterone regulated pathways, myeloid cells were involved in the metabolism of xenobiotics and drugs by CYP450 pathways and B cells were involved in the base-excision-repair and non-homologous end joining pathways, which would affording the comprehensive understanding of downstream function of different immune cells in AAA.

Not only the immune cell infiltration, and the switch phenotype of VSMCs, apoptosis of endothelial cells and proliferation of fibroblast were also involved in the development of AAA [55, 56], which suggests the imbalance of cell-cell communication could produce some causative agents and to affect the homeostasis of target organs. Depending on the “CellphoneDB” R package, we found the cell-cell communication network among the fibroblasts, endothelial cells and myeloid cells was significantly increased by calculating communication probability. More importantly, we identified the communication effects of chemokines, such as CXCL14-CXCR4 and CXCL12-CXCR4, and T cell cytokine, such as TGFβ1-AR, EGFR-TGFβ1 and TGFβ1-TGFβR3 were increased in AAA. Although the pathogenic role of the CXCL12 and its ligands in AAA had been explored [57, 58], our results might provide a novel approach based on the between different cell types to prevent the AAA.

However, our research has some limitations. Firstly, our data analysis comes from the public databases and lacks the original sample analysis, which makes it completely dependent on present studies and leads to the quality of samples being uncontrollable. Second, we used tissue samples to reveal the association between pyroptosis-related genes and AAA. This approach could provide more high-quality and specific and sensitive information for the study, and the measurement of biomarkers levels would more reliably. However, the acquisition of tissue-specific genes requires invasive operations that directly reduce its clinical operability. Besides, the acquisition and analysis of tissue-specific genes require special laboratory equipment and specialized technologies. In addition, the collected tissue samples may be affected by certain factors, such as the patient’s treatment history, the way of sample collection and processing, etc., which may lead to errors in the test results. Therefore, in order to solve the limitations of tissue-specific gene acquisition, we believe that blood markers as a method for early diagnosis and screening of AAA may be more convenient and safer. So, although tissue-specific genes can be used as an ideal method to reveal the mechanism of AAA, its disadvantages also limit its wide applicability in clinical application. The blood markers may provide a feasible method for early diagnosis and screening, and we suggest that the combination of tissue marker genes and blood markers in the future will improve the accuracy and wide applicability of AAA diagnosis. Third, although a large number of gene functional analyses have been performed, the downstream molecular mechanisms are still needing *in vivo* and *in vitro* validation. Next, our model had a good value of the diagnostic accuracy for the AAA patients, but it lacks validation in a large clinical population.

## CONCLUSION

In summary, we constructed a diagnostic model based on the pyroptosis-related genes for AAA, which might be an effective and noninvasive method for AAA in the early stage of the disease. Besides, we identified the hub pyrocytosis-related genes in AAA and demonstrated the potential pathogenic mechanism might be contributed from excess immune infiltration. Moreover, the various of cell subsets at single-cell level and the imbalance cell-cell communication network in AAA were also identified. Our results will contribute to the comprehensive understanding of the pathogenesis of AAA and discover some novel molecular targets for prevention AAA at early stage.

## Supplementary Materials

Supplementary Figures

Supplementary Tables 1 and 3-6

Supplementary Table 2
