# Supplementary material for: Integrated analysis of bulk RNA-seq and single-cell RNA-seq reveals the function of pyrocytosis in the pathogenesis of abdominal aortic aneurysm
Source: Aging (Albany NY). 2023 Dec 18;15(24):15287–323. doi: 10.18632/aging.205350 (PMC10781497; doi:10.18632/aging.205350)
Supplement: Supplementary Figures [file aging-15-205350-s001.pdf]

SUPPLEMENTARY FIGURES

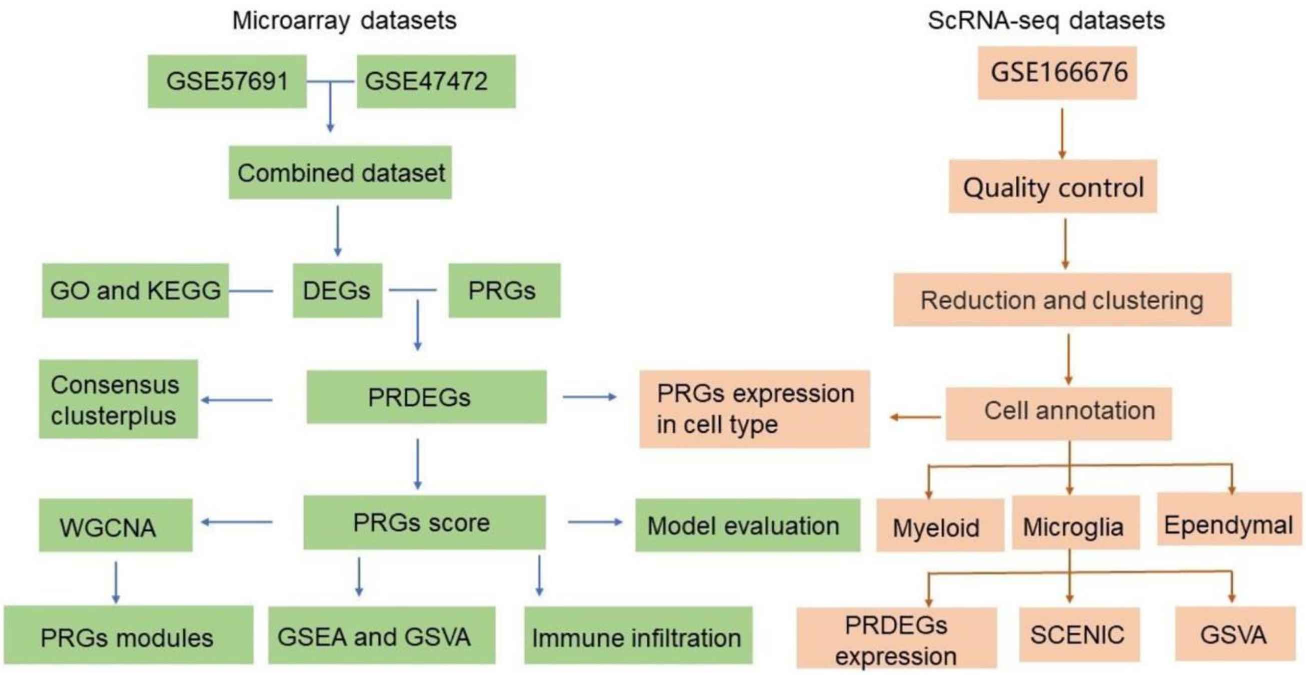

Supplementary Figure 1. Flow chart of the research process. Abbreviations: GO: Gene Ontology; KEGG: Kyoto Encyclopedia of Genes and Genomes; DEGs: Differentially expressed genes; PRGs: Pyroptosis Related genes; PRDEGs: Pyroptosis-related differentially expressed genes; PRGs score: Pyroptosis-related genes score; WGCNA: Weighted gene co-expression network analysis; GSEA: Gene Set Enrichment Analysis; GSVA: Gene Set Variation Analysis; SCENIC: Single-Cell regulatory Network Inference and Clustering.

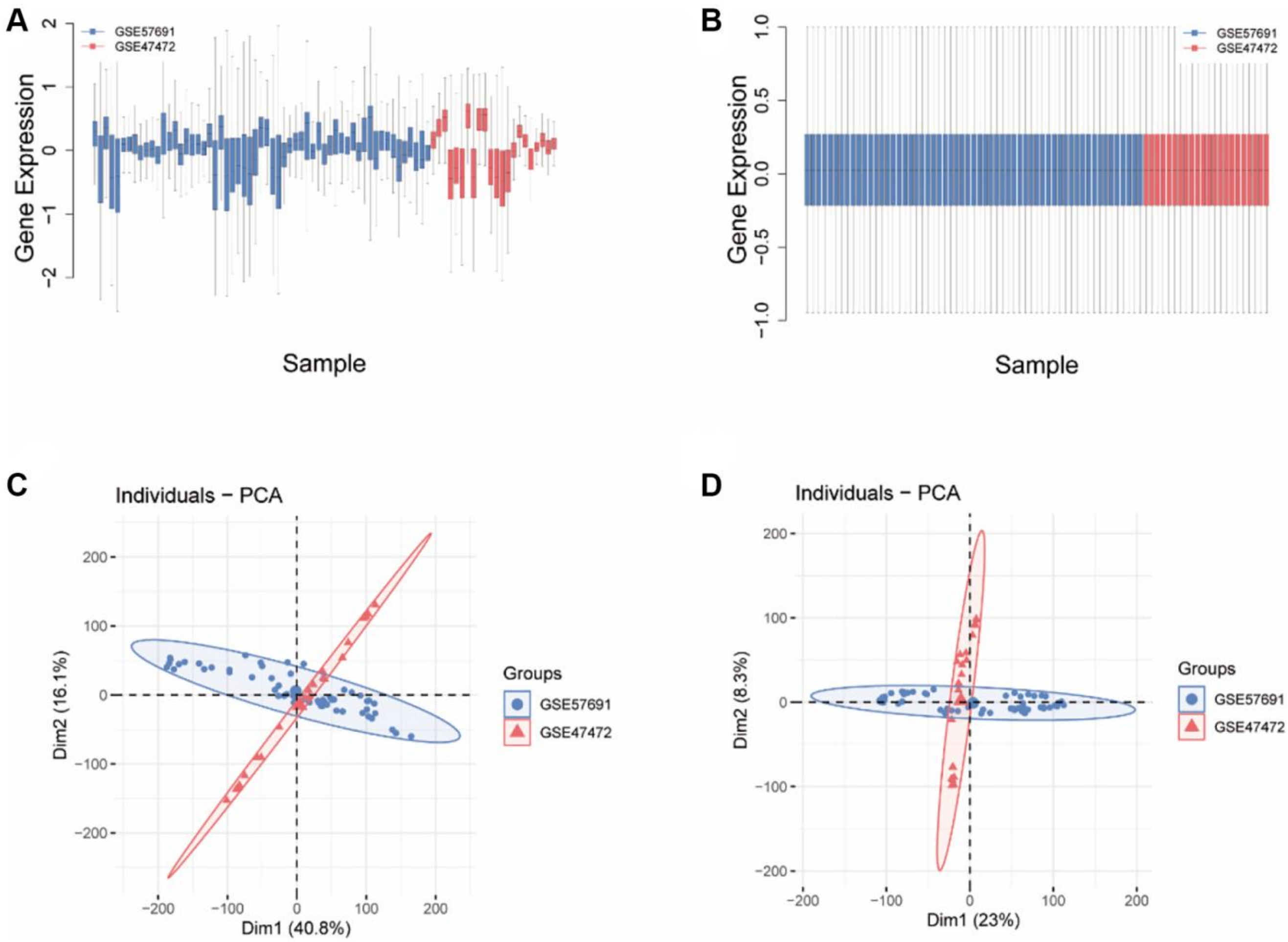

Supplementary Figure 2. Data normalization and remove batch effects of the AAA combined datasets. (**A**, **B**) Boxplot of the abdominal aortic aneurysm dataset before (**A**) and after (**B**) removal of batch effect processing. (**C**, **D**) PCA plots of the abdominal aortic aneurysm dataset before (**C**) and after (**D**) removal of batch effect processing. Abbreviation: PCA: Principal Component Analysis.

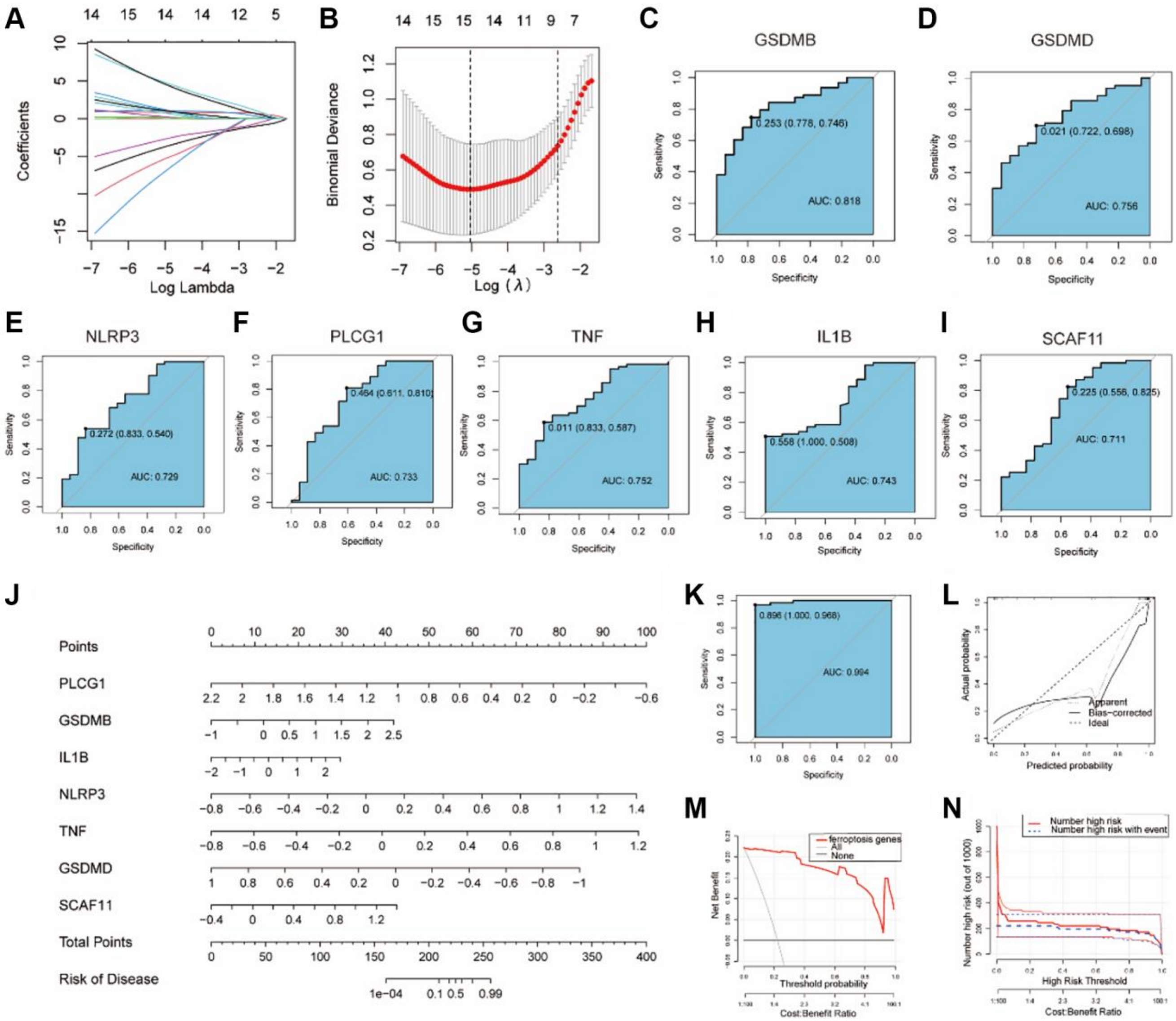

Supplementary Figure 3. Establishment of pyroptosis-related gene score model for AAA prediction. (**A**) LASSO to screen the pyroptosis biomarkers. (**B**) The biomarkers were cross-validated by LASSO regression analysis. ROC curves of GSDMB (**C**), GSDMD (**D**), NLRP3 (**E**), PLCG1 (**F**), TNF (**G**), IL-1β (**H**), and SCAF11 (**I**) in the combined GEO Datasets. (**J**) Nomogram model of pyroptosis genes in the logistic diagnostic model. (**K**) ROC curves of PRGs in combined GEO Datasets. (**L**) Calibration plot of PRGs in the logistic regression model. (**M**) DCA plot of pyroptosis scores in logistic regression models. The ordinate is the net benefit and the abscissa is the threshold or threshold probability. (**N**) Clinical impact curves of PRGs in logistic regression models. The abscissa is the probability threshold, and the ordinate is the number of people. The red line represents the number of people judged as high risk by the model at different probability thresholds. The blue lines represent the number of people who were judged by the model to be at high risk and who actually experienced an outcome event at various probability thresholds. At the bottom is the benefit ratio, which represents the proportion of losses and benefits at different probability thresholds. Abbreviations: ROC: receiver operating characteristic curve; AUC: Area Under Curve; OR: odds ratio; LASSO: Least absolute shrinkage and selection operator; PRGs: Pyroptosis-related gene score; DCA: decision curve analysis.

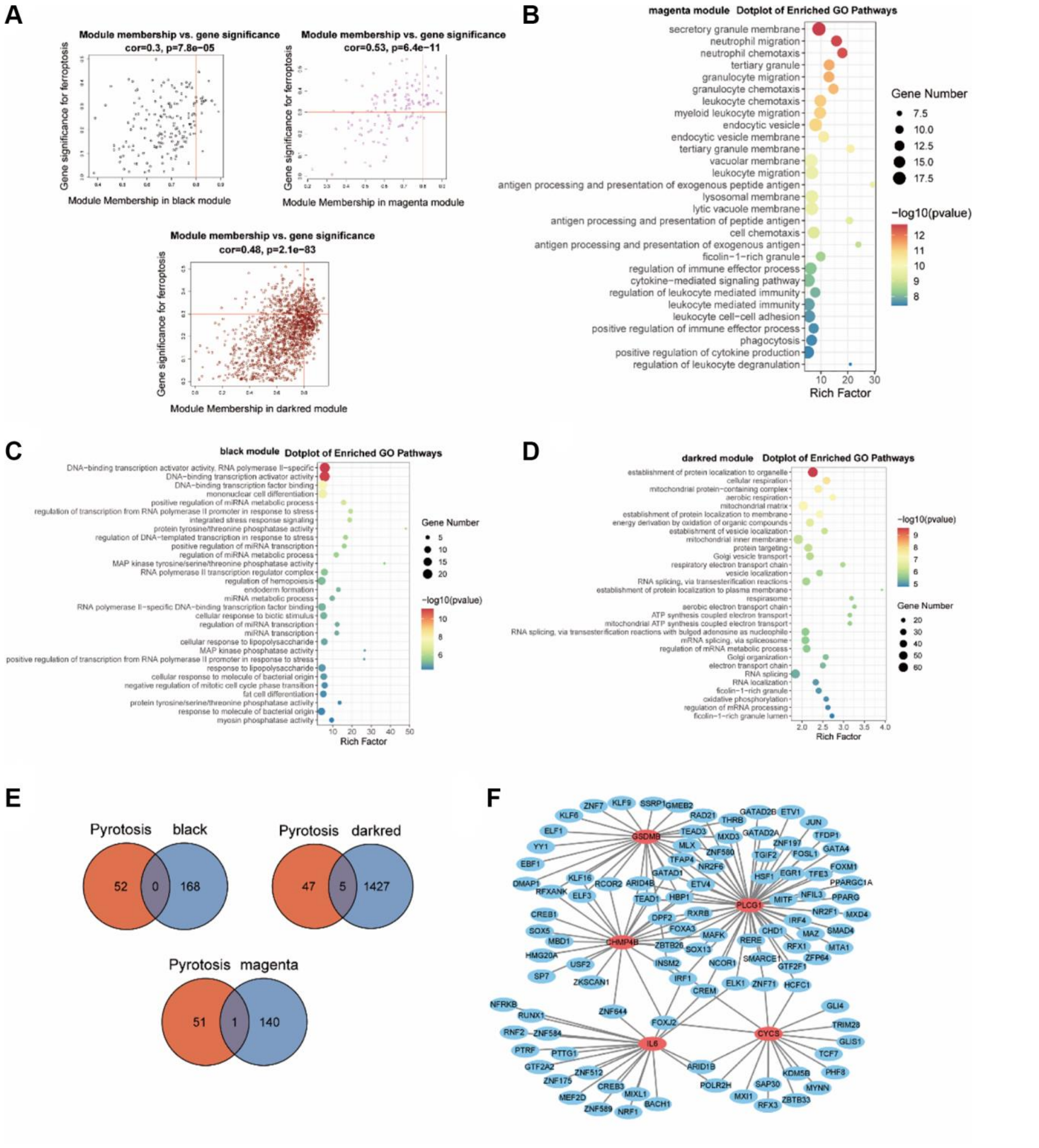

Supplementary Figure 4. Functional enrichment analysis of pyroptosis-related gene modules based on the AAA combined datasets. (**A**) Scatter plots of associations between genes in the black, magenta, and darkred modules and high and low PRGs. (**B**–**D**) Bubble plots of GO enrichment analysis of genes from magenta (**B**) and black (**C**) and darkred (**D**) modules. The bubble size represents the number of enriched genes, the larger the number of enriched genes, the darker the color represents the significance of the *P*-value, and the darker the color represents the more significant the difference. (**E**) Venn diagram of black, magenta, and darkred module genes and pyroptotic genes. (**F**) Network diagram showing the interaction between transcription factors and pyroptosis Hub genes. Abbreviations: GO: Gene Ontology; KEGG: Kyoto Encyclopedia of Genes and Genomes.

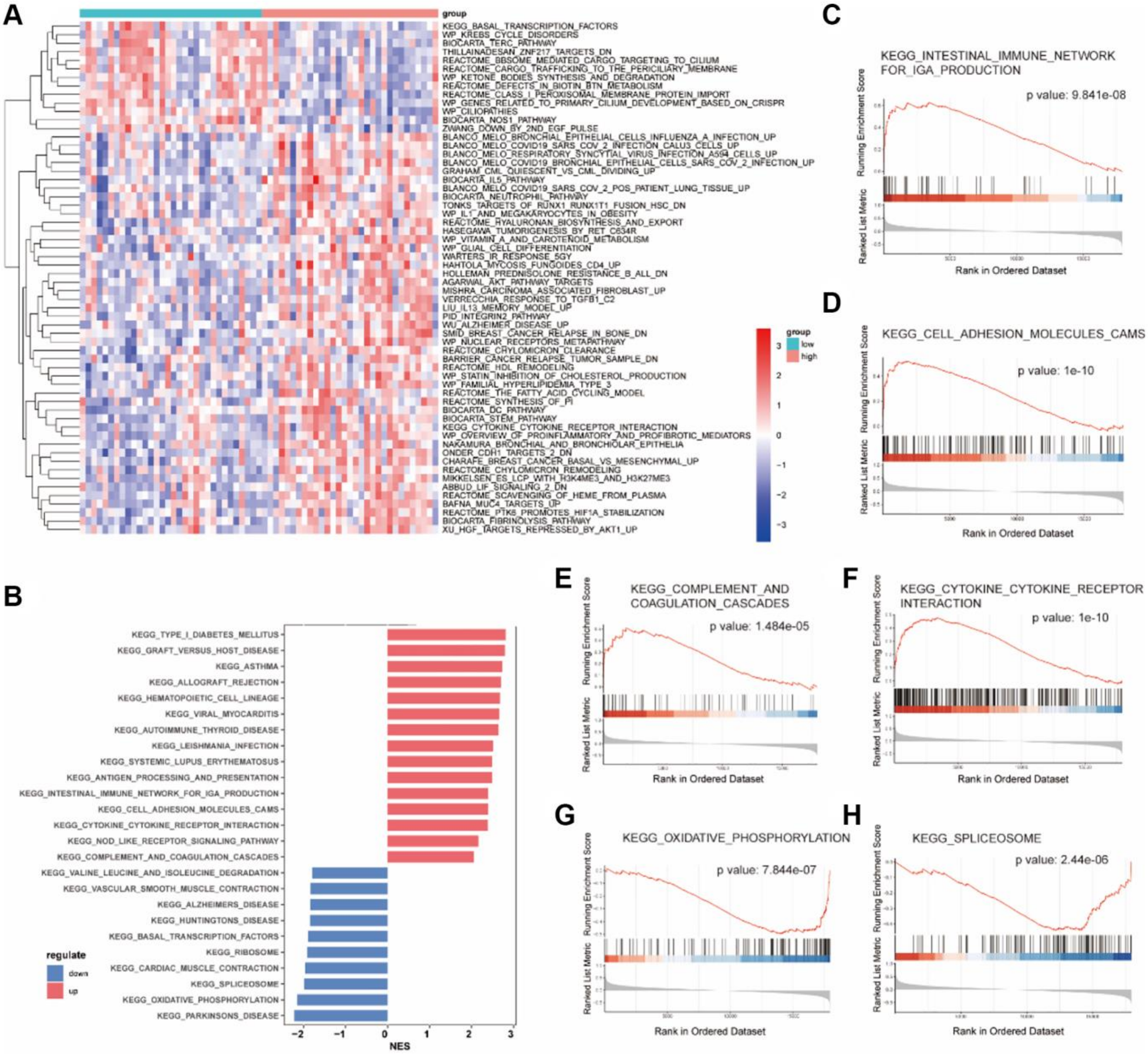

Supplementary Figure 5. Enrichment analysis of GSVA and GSEA of high and low PRGs groups based on the AAA combined datasets. (**A**) Heatmap of GSVA enrichment analysis results between high and low PRGs groups. Red represents a high GSVA score and blue represents a low GSVA score. (**B**) GSEA enrichment analysis bar graph between high and low PRGs groups. Red represents the upregulation of AAA group and blue represents the downregulation of AAA group. (**C**–**H**) GSEA enrichment maps of KEGG_INTESTINAL_IMMUNE_NETWORK_FOR_IGA_PRODUCTION (**C**), KEGG_CELL_ADHESION_MOLECULES_CAMS (**D**), KEGG_COMPLEMENT_AND_COAGULATION_CASCADES (**E**), KEGG_CYTOKINE_CYTOKINE_RECEPTOR_INTERACTION (**F**), KEGG_OXIDATIVE_PHOSPHORYLATION (**G**) and KEGG_SPLICEOSOME (**H**). Abbreviations: GSEA: Gene Set Enrichment Analysis; GSVA: Gene Set Variation Analysis; AAA: abdominal aortic aneurysm.

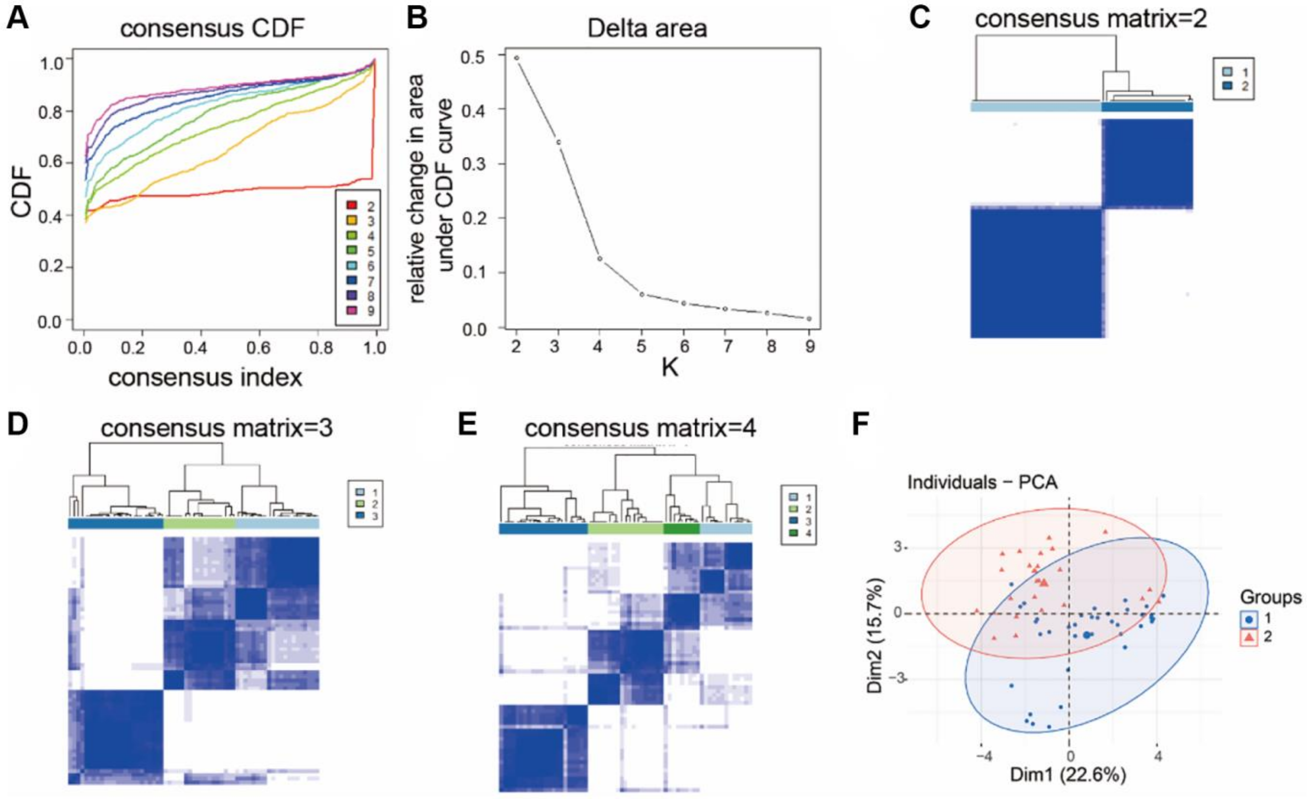

Supplementary Figure 6. Molecular typing of pyroptosis-related genes in AAA patients based on the AAA combined datasets. (**A**) Results of consensus cluster analysis, A function plot of the cumulative distribution of consensus clusters with k values of 2 to 9. (**B**) Relative change in the area under the curve of the cumulative distribution function plot. (**C**–**E**) Cluster plots of samples with k = 2 (**C**), k3 (**D**), and k4 (**E**). The tracking curve showed that the optimal grouping was when k = 3. (**F**) PCA analysis of three subgroups; Blue represents Cluster1 patients and red represents Cluster1 patients.

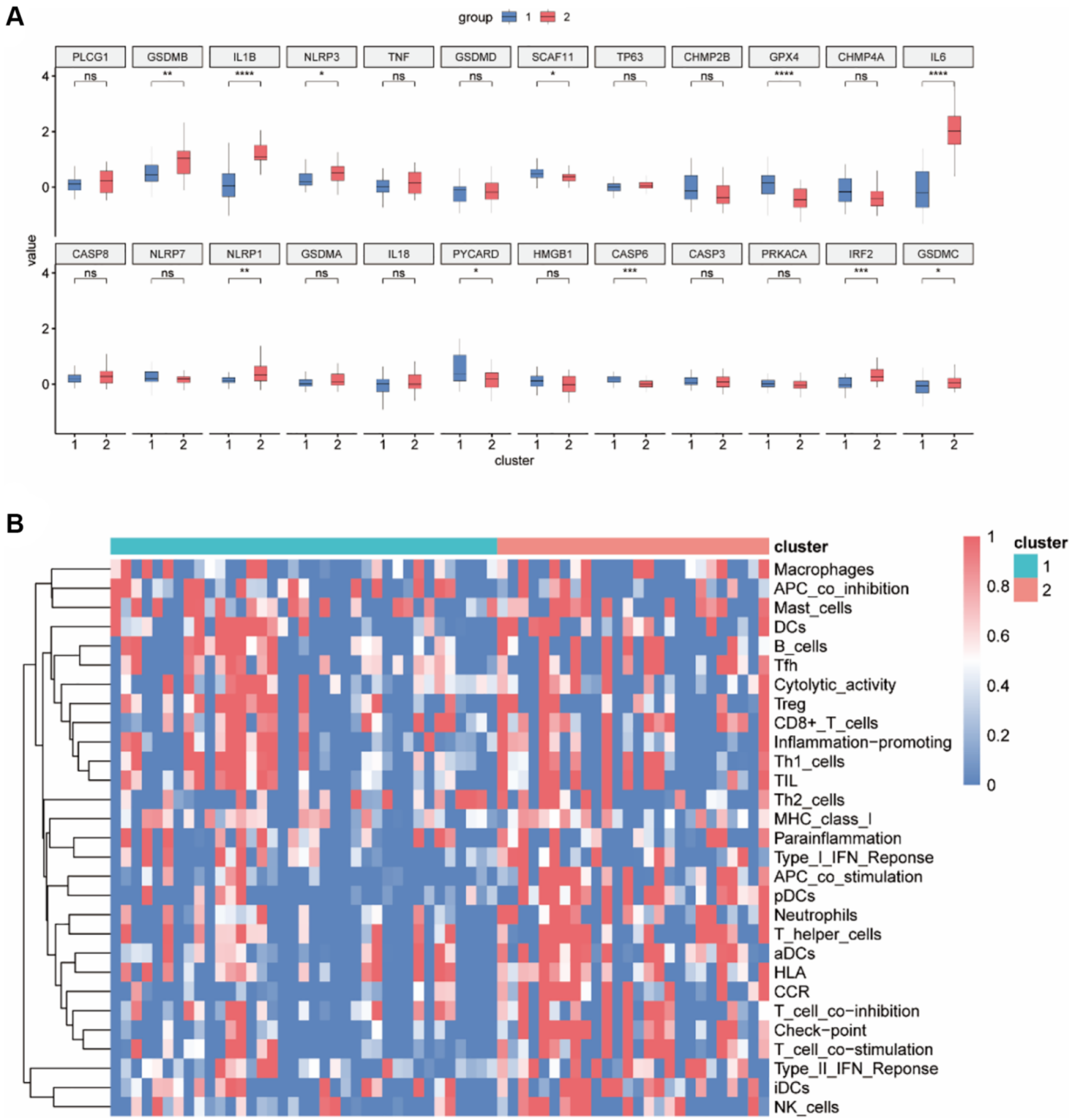

Supplementary Figure 7. Analysis of immune characteristics of pyroptosis subtypes in AAA patients based on the AAA combined datasets. (**A**) Boxplot of pyroptotic gene expression in 2 subgroups, blue for Cluster1 patients, orange for Cluster2 patients, and red for Cluster3 patients. (**B**) The estimate method was used to detect immune cell scores in the two subgroups. Red represents a high score and blue represents a low score. $^{*}P < 0.05$, $^{**}P < 0.01$, $^{***}P < 0.001$. Abbreviation: AAA: Abdominal aortic aneurysm.

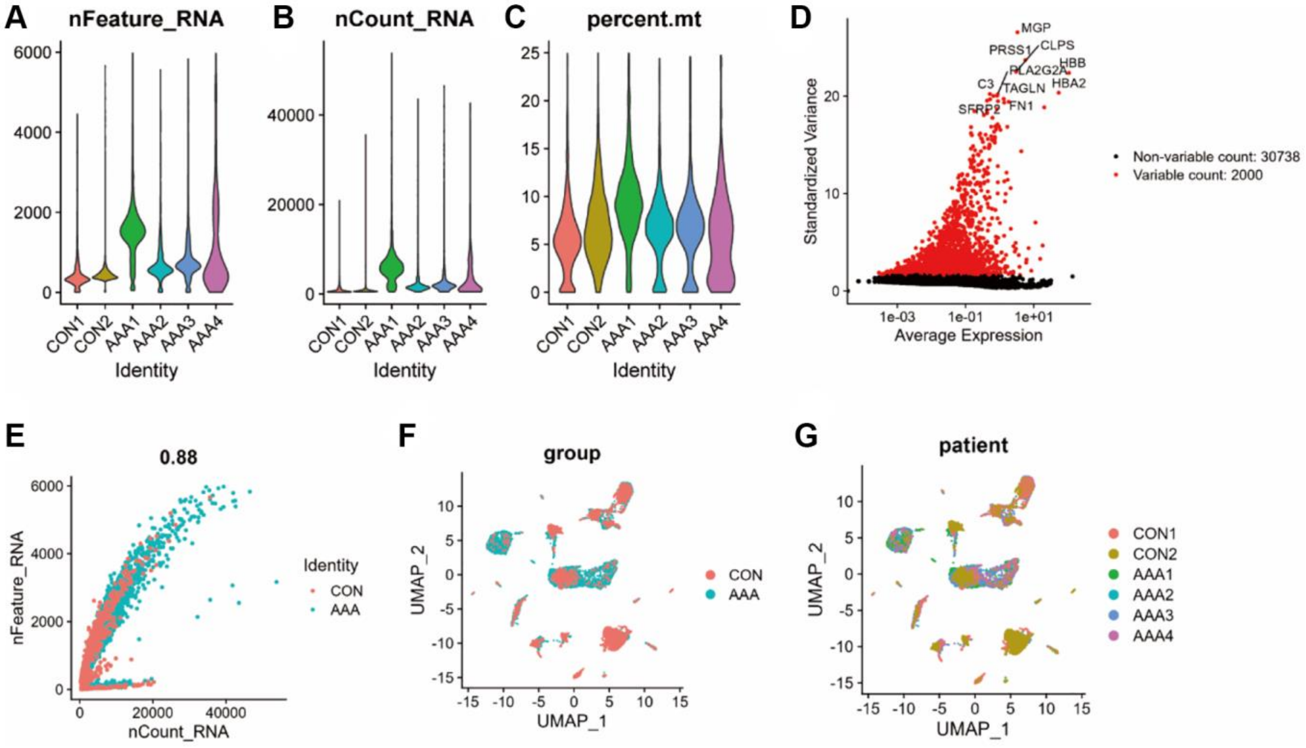

Supplementary Figure 8. Single-cell quality control in GSE166676 databases. (**A**–**C**) Violin plot of number of genes and sequenced counts and proportion of mitochondria in the GSE166676 databases. (**D**) Scatter plot of hypervariable genes. (**E**) Scatter plot of the correlation between nCount and nFeature in all cells. (**F**) UMAP plot demonstrating the dimensionality reduction of single-cell sequencing in AAA group and control group. (**G**) UMAP plot showing the dimensionality reduction effect of single-cell sequencing in AAA group and control group at different times.

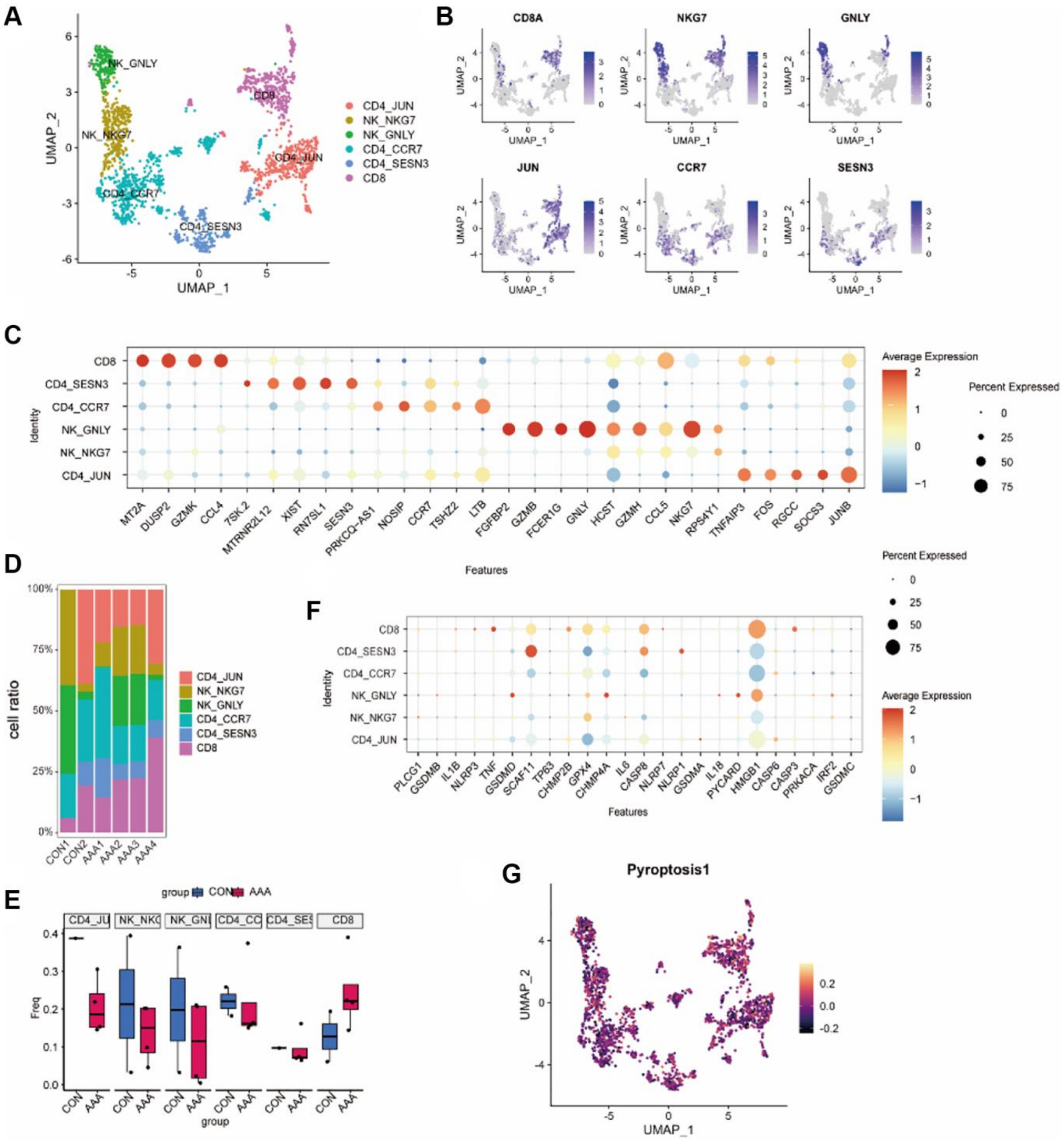

Supplementary Figure 9. Identification of T cell subsets in the GSE166676 dataset. (**A**) UMAP diagram showing the subtypes of T cells. (**B**) Heatmap of hallmark genes expression of T cell subsets. Gray representing low gene expression and blue representing high gene expression. (**C**) The bubble plot shows the expression level of the signature gene in different cell subsets, the color depth represents the expression level of the gene, and the size of the dot represents the positive proportion of the gene in the cell. (**D**) Bar graph of the proportions of T cell subsets in AAA group and CON group. (**E**) Box plot of the proportions of T cell subsets in AAA group and CON group. (**F**) Bubble plot shows the expression levels of pyroptosis-related genes in different cell subsets, the color depth represents the expression level of the gene, and the size of the dot represents the positive proportion of the gene in the cell. (**G**) Heatmap shows pyroptosis scores for T cell subsets, with black representing low pyroptosis scores and orange representing high pyroptosis scores.

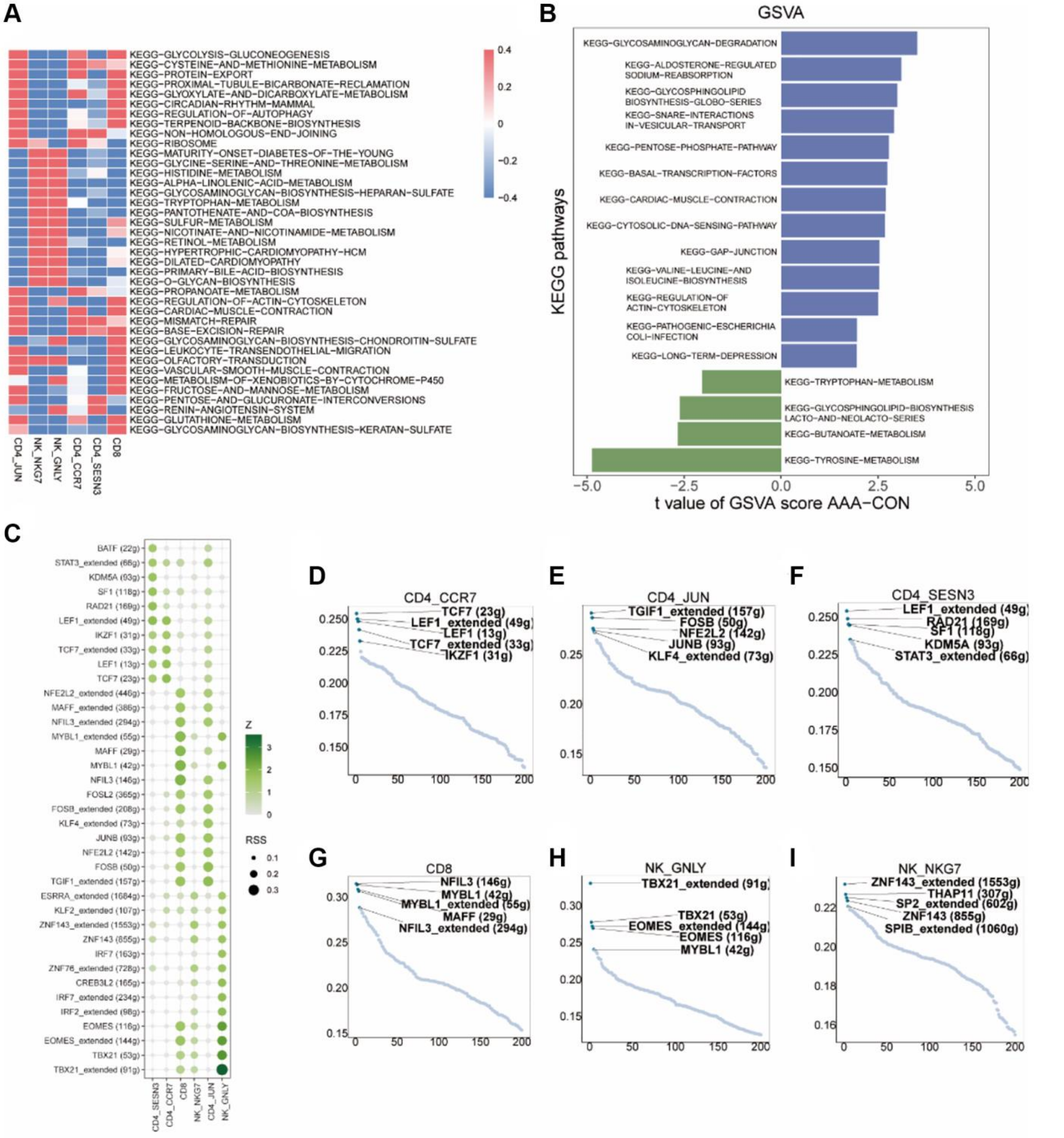

Supplementary Figure 10. GSVA analysis and transcription factor analysis of T cell subsets in the GSE166676 datasets. (**A**) Heatmap of the results of GSVA enrichment analysis among different T cell subsets, with low expression in blue and high expression in red. (**B**) Bar graph of the enrichment analysis results of T cell subsets in the AAA and CON groups. Blue represents the upregulated pathway in AAA group and green represents the downregulated pathway in AAA group. (**C**) Bubble plot of SCENIC analysis of transcription factor activity between different T cell subsets, color depth represents transcription factor activity, circle size represents transcription factor specificity score. (D-I) Scatter plots showing specific transcription factors for CD4_CCR7 (**D**), CD4_JUN (**E**), CD4_SESN3 (**F**), CD8+ T (**G**), NK_GNLY (**H**) and NK_NKG7 (**I**). The horizontal axis shows sequencing, and vertical axis shows specificity, the five most specific TFS are labeled in white. Abbreviations: GSVA: Gene Set Variation Analysis; AAA: abdominal aortic aneurysm; CON: Control; RSS: regulon specificity score.

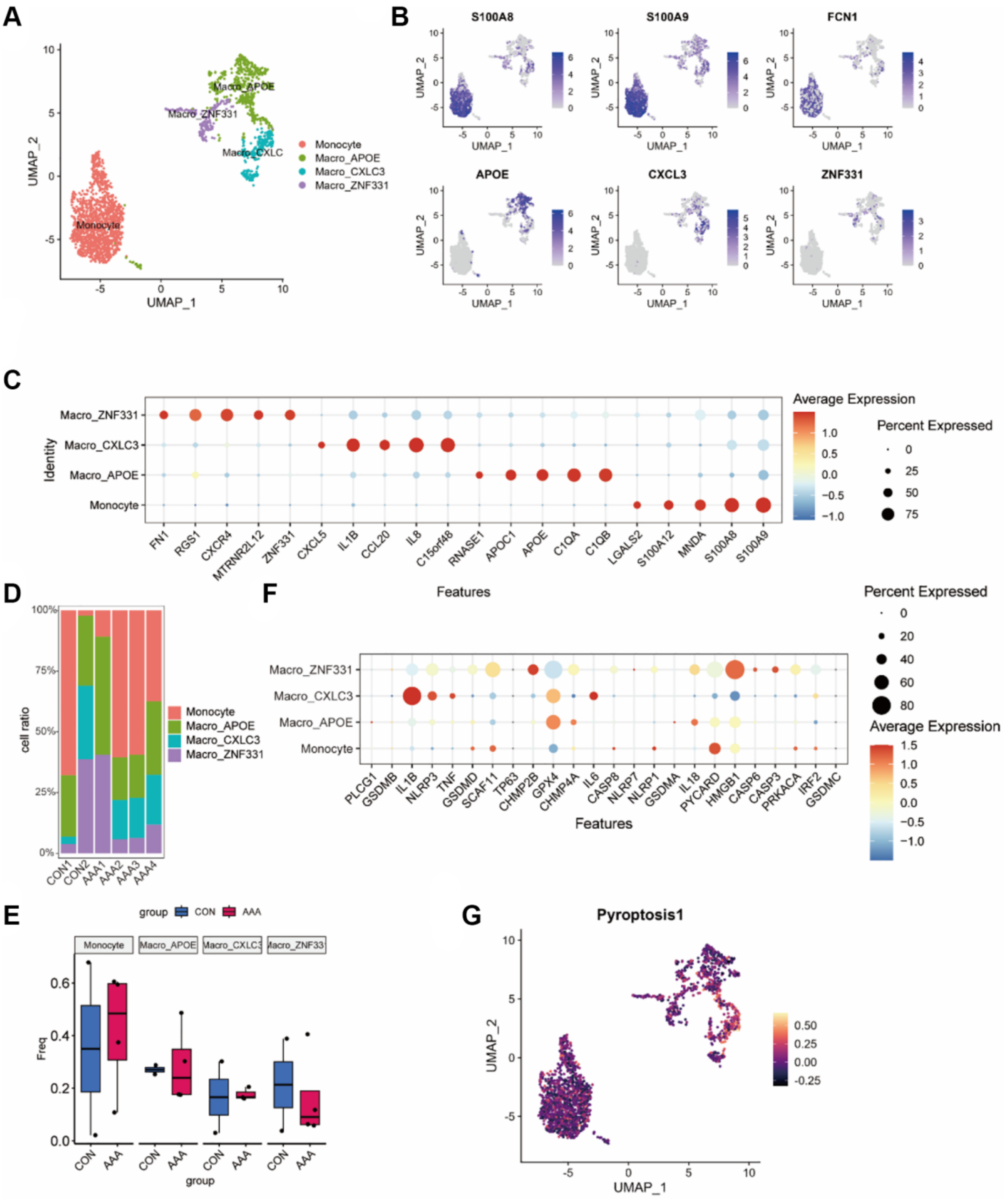

Supplementary Figure 11. Identification of myeloid cell subsets in the GSE166676 dataset. (**A**) UMAP diagram demonstrating myeloid cell subsets. (**B**) Heatmap of myeloid cell subsets marker gene expression, gray represents low gene expression and blue represents high gene expression. (**C**) The bubble plot shows the expression level of the signature gene in different cell subsets, the color depth represents the expression level of the gene, and the size of the dot represents the positive proportion of the gene in the cell. (**D**, **E**) Proportion of myeloid cell subsets in AAA group and CON group that displayed by bar (**D**) and box (**E**) plots. (**F**) Bubble plot shows the expression levels of pyroptosis-related genes in myeloid cell subsets, the color depth represents the expression level of the gene, and the size of the dot represents the positive proportion of the gene in the cell. (**G**) Heatmap shows the pyroptosis score of myeloid cell subsets, with black representing low pyroptosis score and orange representing high pyroptosis score. Abbreviations: UMAP: Uniform Manifold Approximation and Projection; AAA: abdominal aortic aneurysm; CON: Control.

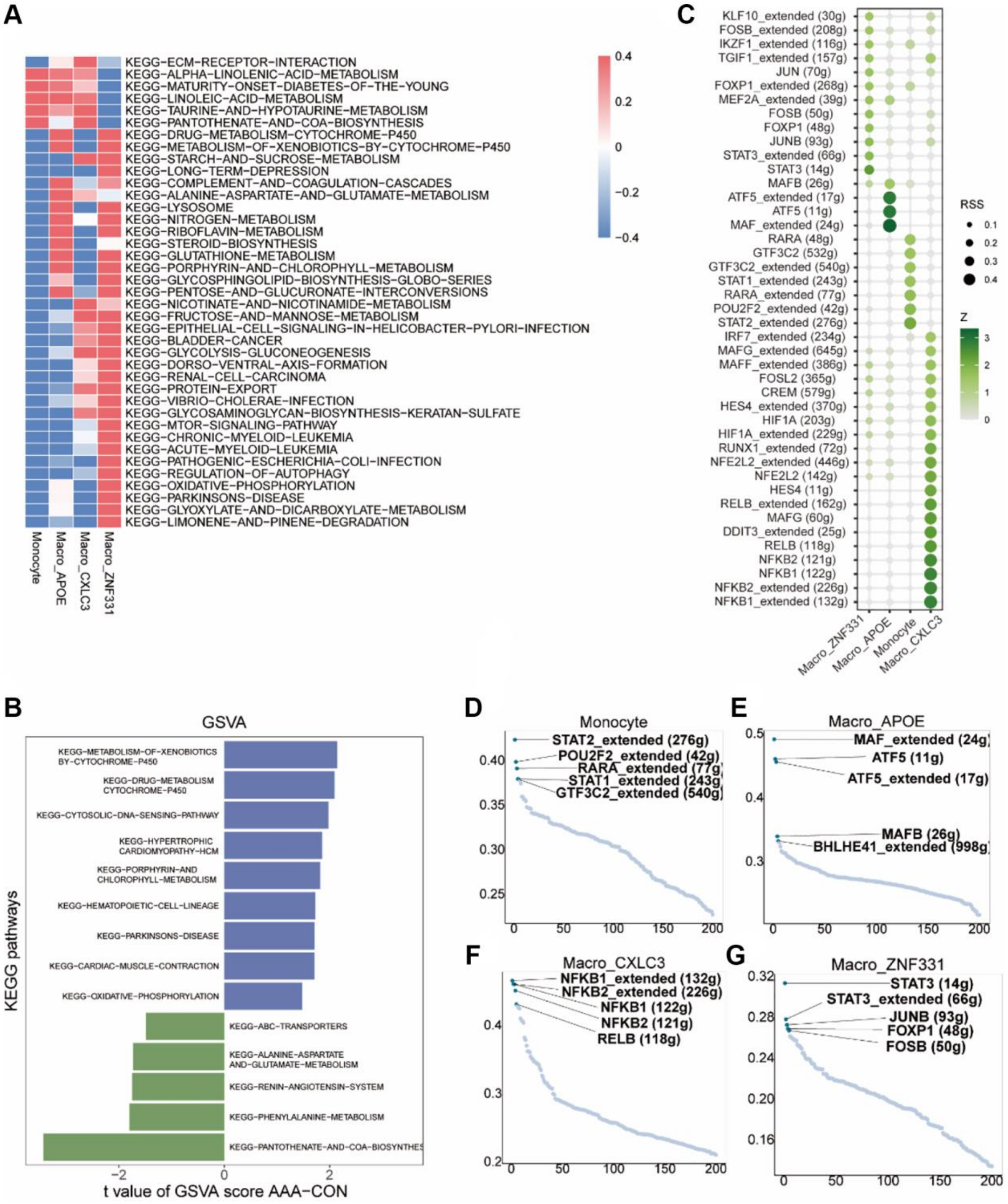

Supplementary Figure 12. GSVA analysis and transcription factor analysis of myeloid cell subsets in the GSE166676 datasets. (**A**) Heatmap of GSVA enrichment analysis results between different subsets of myeloid cells, blue for low expression and red for high expression. (**B**) Bar graph of enrichment analysis results of myeloid cell AAA group compared with CON group. Blue represents the upregulated pathway in AAA group and green represents the downregulated pathway in AAA group. (**C**) Bubble plot of SCENIC analysis of transcription factor activity between different subsets of myeloid cells, color depth represents transcription factor activity, circle size represents transcription factor specificity score. (**D**–**G**) Scatter plot showing the specific transcription factors of monocyte (**D**), Macro_APOE (**E**), Macro_CXCL13 (**F**) and Macro_ZFN331 (**G**). The horizontal axis shows rank and vertical axis shows specificity, and the five most specific transcription factors are labeled in white. Abbreviations: GSVA: Gene Set Variation Analysis; AAA: abdominal aortic aneurysm; CON: Control; RSS: regulon specificity score.

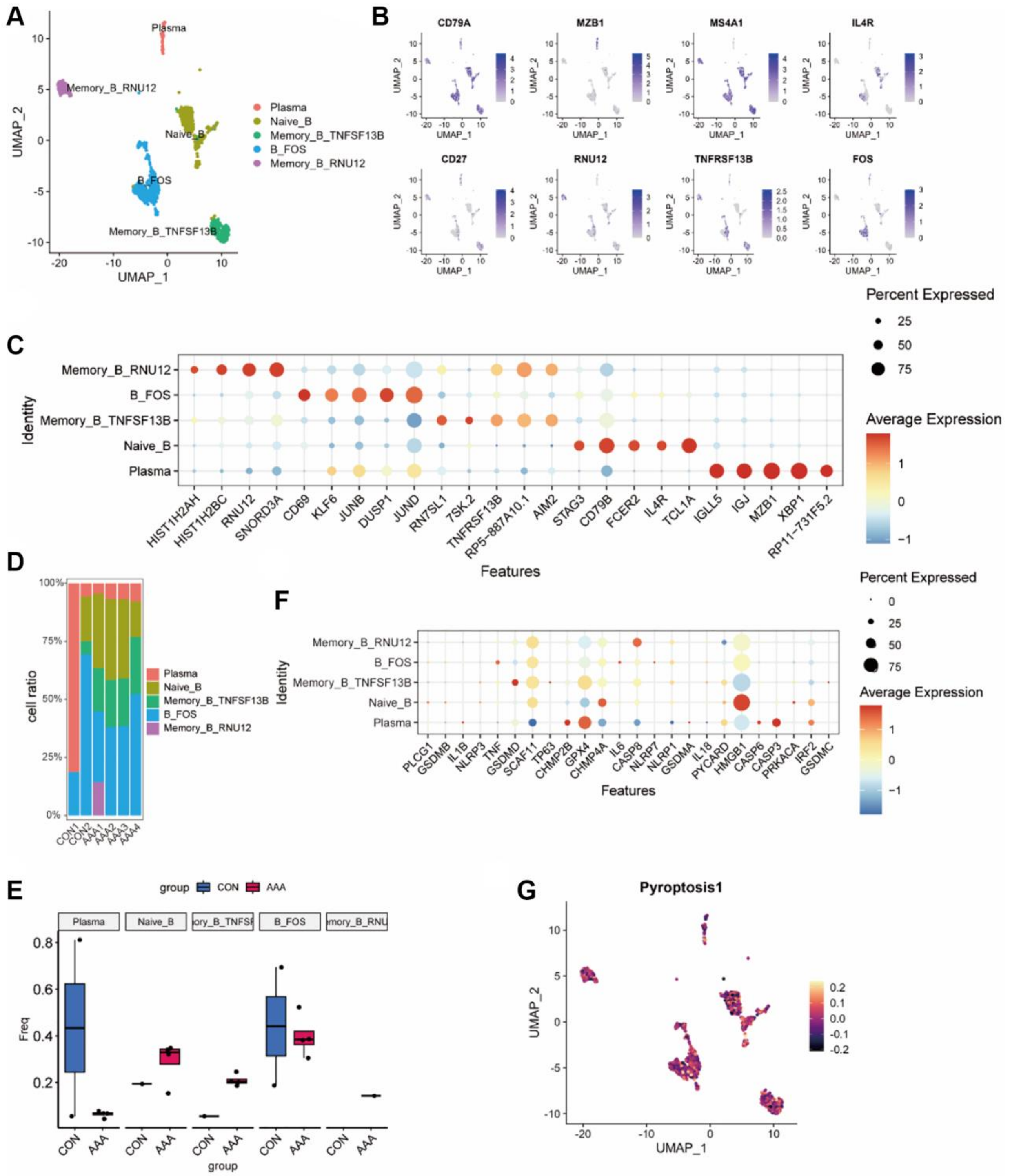

Supplementary Figure 13. Identification of B cell subsets in the GSE166676 dataset. (**A**) UMAP map showing B cell subsets. (**B**) Heatmap of signature genes expression in B-cell subsets. Gray represents low gene expression and blue represents high gene expression. (**C**) The bubble plot shows the expression level of the signature gene in different cell subsets, the color depth represents the expression level of the gene, and the size of the dot represents the positive proportion of the gene in the cell. Bar (**D**) and box (**E**) plots of B-cell subsets AAA group and CON group. *P* < 0.05 was considered statistically significant. (**F**) Bubble plot shows the expression levels of pyroptosis-related genes in B cell subsets, the color depth represents the expression level of the gene, and the size of the dot represents the positive proportion of the gene in the cell. (**G**) Heatmap shows pyroptosis scores for B-cell subsets, with black representing low pyroptosis scores and orange representing high pyroptosis scores. Abbreviations: UMAP: Uniform Manifold Approximation and Projection; AAA: abdominal aortic aneurysm; CON: Control.

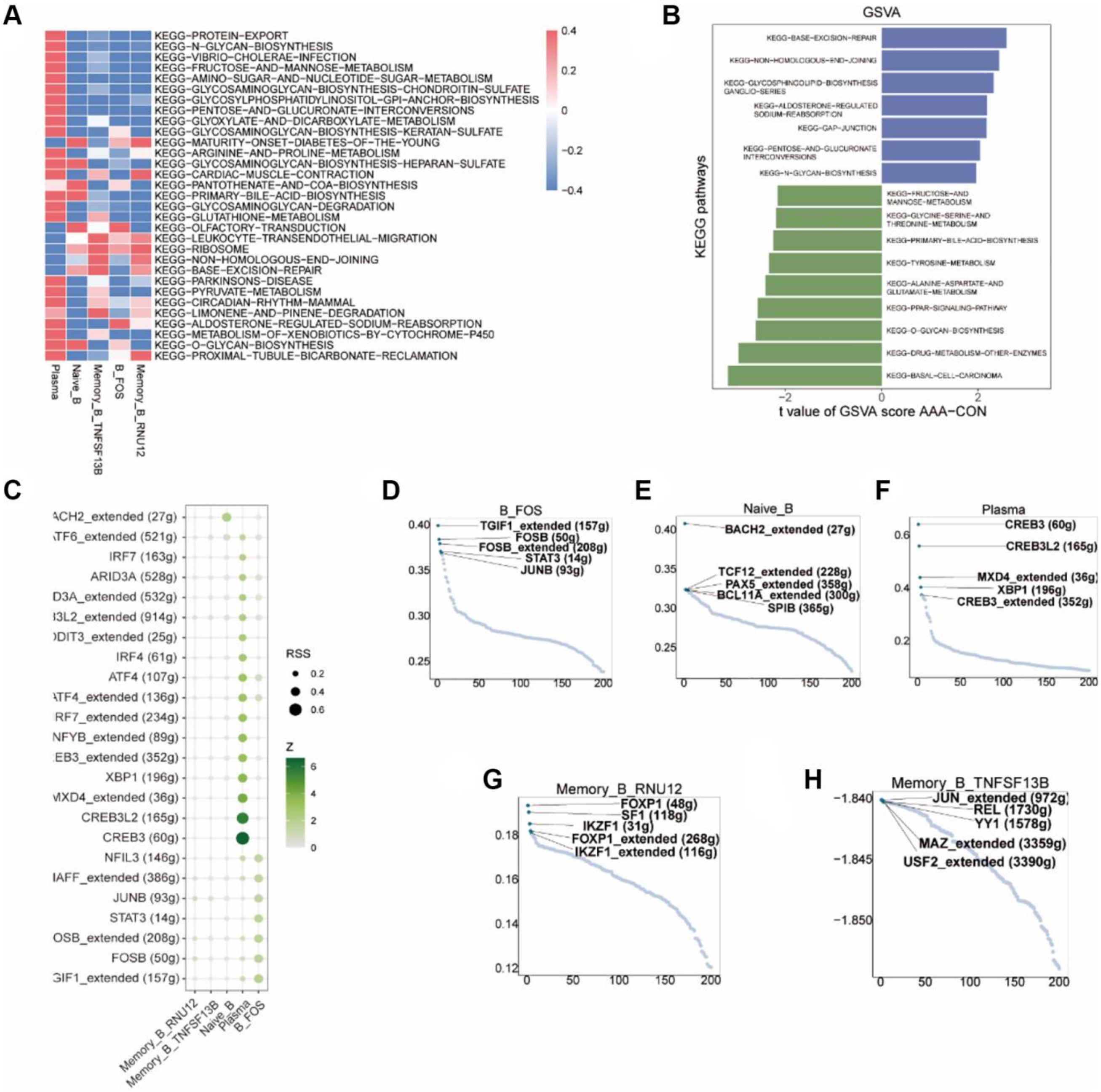

Supplementary Figure 14. GSVA analysis and transcription factor analysis of B cell subsets in the GSE166676 datasets. (**A**) Heatmap of GSVA enrichment analysis results between different subsets of B cell, blue for low expression and red for high expression. (**B**) Bar graph of enrichment analysis results of the B cell in AAA group and CON group. Blue represents the upregulated pathway in AAA group and green represents the downregulated pathway in AAA group. (**C**) Bubble plot of SCENIC analysis of transcription factor activity between different subsets of B cells, color depth represents transcription factor activity, circle size represents transcription factor specificity score. Scatter plot showing the specific transcription factors in B_FOS (**D**), Naive_B (**E**), Plasma (**F**), Memory_B_RNU12 (**G**) and Memory_B_TNFRSF13B (**H**). The horizontal axis shows ranking, the vertical axis shows specificity. The five most specific TFS are labeled in white.
