# Supplementary material for: Integrated analysis of bulk RNA-seq and single-cell RNA-seq reveals the function of pyrocytosis in the pathogenesis of abdominal aortic aneurysm
Source: Aging (Albany NY). 2023 Dec 18;15(24):15287–323. doi: 10.18632/aging.205350 (PMC10781497; doi:10.18632/aging.205350)
Supplement: Supplementary Tables 1 and 3-6 [file aging-15-205350-s002.docx]

**Supplementary Table 1. The gene information of bulk RNA-seq and single-cell RNA-seq from the GSE57691 and GSE47472 databases.**

| **GSE57691** | **title** | **geo_accession** | **characteristics_ch1** | **characteristics_ch1.1** | **GEO** |
| --- | --- | --- | --- | --- | --- |
| GSM1386783 | small AAA_Sample 1 | GSM1386783 | disease state: small AAA | subjects: patients with AAA undergoing open surgery to treat AAA | GSE57691 |
| GSM1386784 | small AAA_Sample 2 | GSM1386784 | disease state: small AAA | subjects: patients with AAA undergoing open surgery to treat AAA | GSE57691 |
| GSM1386785 | small AAA_Sample 3 | GSM1386785 | disease state: small AAA | subjects: patients with AAA undergoing open surgery to treat AAA | GSE57691 |
| GSM1386786 | small AAA_Sample 4 | GSM1386786 | disease state: small AAA | subjects: patients with AAA undergoing open surgery to treat AAA | GSE57691 |
| GSM1386787 | small AAA_Sample 5 | GSM1386787 | disease state: small AAA | subjects: patients with AAA undergoing open surgery to treat AAA | GSE57691 |
| GSM1386788 | small AAA_Sample 6 | GSM1386788 | disease state: small AAA | subjects: patients with AAA undergoing open surgery to treat AAA | GSE57691 |
| GSM1386789 | small AAA_Sample 7 | GSM1386789 | disease state: small AAA | subjects: patients with AAA undergoing open surgery to treat AAA | GSE57691 |
| GSM1386790 | small AAA_Sample 8 | GSM1386790 | disease state: small AAA | subjects: patients with AAA undergoing open surgery to treat AAA | GSE57691 |
| GSM1386791 | small AAA_Sample 9 | GSM1386791 | disease state: small AAA | subjects: patients with AAA undergoing open surgery to treat AAA | GSE57691 |
| GSM1386792 | small AAA_Sample 10 | GSM1386792 | disease state: small AAA | subjects: patients with AAA undergoing open surgery to treat AAA | GSE57691 |
| GSM1386793 | small AAA_Sample 11 | GSM1386793 | disease state: small AAA | subjects: patients with AAA undergoing open surgery to treat AAA | GSE57691 |
| GSM1386794 | small AAA_Sample 12 | GSM1386794 | disease state: small AAA | subjects: patients with AAA undergoing open surgery to treat AAA | GSE57691 |
| GSM1386795 | small AAA_Sample 13 | GSM1386795 | disease state: small AAA | subjects: patients with AAA undergoing open surgery to treat AAA | GSE57691 |
| GSM1386796 | small AAA_Sample 14 | GSM1386796 | disease state: small AAA | subjects: patients with AAA undergoing open surgery to treat AAA | GSE57691 |
| GSM1386797 | small AAA_Sample 15 | GSM1386797 | disease state: small AAA | subjects: patients with AAA undergoing open surgery to treat AAA | GSE57691 |
| GSM1386798 | small AAA_Sample 16 | GSM1386798 | disease state: small AAA | subjects: patients with AAA undergoing open surgery to treat AAA | GSE57691 |
| GSM1386799 | small AAA_Sample 17 | GSM1386799 | disease state: small AAA | subjects: patients with AAA undergoing open surgery to treat AAA | GSE57691 |
| GSM1386800 | small AAA_Sample 18 | GSM1386800 | disease state: small AAA | subjects: patients with AAA undergoing open surgery to treat AAA | GSE57691 |
| GSM1386801 | small AAA_Sample 19 | GSM1386801 | disease state: small AAA | subjects: patients with AAA undergoing open surgery to treat AAA | GSE57691 |
| GSM1386802 | small AAA_Sample 20 | GSM1386802 | disease state: small AAA | subjects: patients with AAA undergoing open surgery to treat AAA | GSE57691 |
| GSM1386803 | large AAA_Sample 21 | GSM1386803 | disease state: large AAA | subjects: patients with AAA undergoing open surgery to treat AAA | GSE57691 |
| GSM1386804 | large AAA_Sample 22 | GSM1386804 | disease state: large AAA | subjects: patients with AAA undergoing open surgery to treat AAA | GSE57691 |
| GSM1386805 | large AAA_Sample 23 | GSM1386805 | disease state: large AAA | subjects: patients with AAA undergoing open surgery to treat AAA | GSE57691 |
| GSM1386806 | large AAA_Sample 24 | GSM1386806 | disease state: large AAA | subjects: patients with AAA undergoing open surgery to treat AAA | GSE57691 |
| GSM1386807 | large AAA_Sample 25 | GSM1386807 | disease state: large AAA | subjects: patients with AAA undergoing open surgery to treat AAA | GSE57691 |
| GSM1386808 | large AAA_Sample 26 | GSM1386808 | disease state: large AAA | subjects: patients with AAA undergoing open surgery to treat AAA | GSE57691 |
| GSM1386809 | large AAA_Sample 27 | GSM1386809 | disease state: large AAA | subjects: patients with AAA undergoing open surgery to treat AAA | GSE57691 |
| GSM1386810 | large AAA_Sample 28 | GSM1386810 | disease state: large AAA | subjects: patients with AAA undergoing open surgery to treat AAA | GSE57691 |
| GSM1386811 | large AAA_Sample 29 | GSM1386811 | disease state: large AAA | subjects: patients with AAA undergoing open surgery to treat AAA | GSE57691 |
| GSM1386812 | large AAA_Sample 30 | GSM1386812 | disease state: large AAA | subjects: patients with AAA undergoing open surgery to treat AAA | GSE57691 |
| GSM1386813 | large AAA_Sample 31 | GSM1386813 | disease state: large AAA | subjects: patients with AAA undergoing open surgery to treat AAA | GSE57691 |
| GSM1386814 | large AAA_Sample 32 | GSM1386814 | disease state: large AAA | subjects: patients with AAA undergoing open surgery to treat AAA | GSE57691 |
| GSM1386815 | large AAA_Sample 33 | GSM1386815 | disease state: large AAA | subjects: patients with AAA undergoing open surgery to treat AAA | GSE57691 |
| GSM1386816 | large AAA_Sample 34 | GSM1386816 | disease state: large AAA | subjects: patients with AAA undergoing open surgery to treat AAA | GSE57691 |
| GSM1386817 | large AAA_Sample 35 | GSM1386817 | disease state: large AAA | subjects: patients with AAA undergoing open surgery to treat AAA | GSE57691 |
| GSM1386818 | large AAA_Sample 36 | GSM1386818 | disease state: large AAA | subjects: patients with AAA undergoing open surgery to treat AAA | GSE57691 |
| GSM1386819 | large AAA_Sample 37 | GSM1386819 | disease state: large AAA | subjects: patients with AAA undergoing open surgery to treat AAA | GSE57691 |
| GSM1386820 | large AAA_Sample 38 | GSM1386820 | disease state: large AAA | subjects: patients with AAA undergoing open surgery to treat AAA | GSE57691 |
| GSM1386821 | large AAA_Sample 39 | GSM1386821 | disease state: large AAA | subjects: patients with AAA undergoing open surgery to treat AAA | GSE57691 |
| GSM1386822 | large AAA_Sample 40 | GSM1386822 | disease state: large AAA | subjects: patients with AAA undergoing open surgery to treat AAA | GSE57691 |
| GSM1386823 | large AAA_Sample 41 | GSM1386823 | disease state: large AAA | subjects: patients with AAA undergoing open surgery to treat AAA | GSE57691 |
| GSM1386824 | large AAA_Sample 42 | GSM1386824 | disease state: large AAA | subjects: patients with AAA undergoing open surgery to treat AAA | GSE57691 |
| GSM1386825 | large AAA_Sample 43 | GSM1386825 | disease state: large AAA | subjects: patients with AAA undergoing open surgery to treat AAA | GSE57691 |
| GSM1386826 | large AAA_Sample 44 | GSM1386826 | disease state: large AAA | subjects: patients with AAA undergoing open surgery to treat AAA | GSE57691 |
| GSM1386827 | large AAA_Sample 45 | GSM1386827 | disease state: large AAA | subjects: patients with AAA undergoing open surgery to treat AAA | GSE57691 |
| GSM1386828 | large AAA_Sample 46 | GSM1386828 | disease state: large AAA | subjects: patients with AAA undergoing open surgery to treat AAA | GSE57691 |
| GSM1386829 | large AAA_Sample 47 | GSM1386829 | disease state: large AAA | subjects: patients with AAA undergoing open surgery to treat AAA | GSE57691 |
| GSM1386830 | large AAA_Sample 48 | GSM1386830 | disease state: large AAA | subjects: patients with AAA undergoing open surgery to treat AAA | GSE57691 |
| GSM1386831 | large AAA_Sample 49 | GSM1386831 | disease state: large AAA | subjects: patients with AAA undergoing open surgery to treat AAA | GSE57691 |
| GSM1386841 | Donor_Sample 59 | GSM1386841 | disease state: control | subjects: heart-beating, brain-dead donors | GSE57691 |
| GSM1386842 | Donor_Sample 60 | GSM1386842 | disease state: control | subjects: heart-beating, brain-dead donors | GSE57691 |
| GSM1386843 | Donor_Sample 61 | GSM1386843 | disease state: control | subjects: heart-beating, brain-dead donors | GSE57691 |
| GSM1386844 | Donor_Sample 62 | GSM1386844 | disease state: control | subjects: heart-beating, brain-dead donors | GSE57691 |
| GSM1386845 | Donor_Sample 63 | GSM1386845 | disease state: control | subjects: heart-beating, brain-dead donors | GSE57691 |
| GSM1386846 | Donor_Sample 64 | GSM1386846 | disease state: control | subjects: heart-beating, brain-dead donors | GSE57691 |
| GSM1386847 | Donor_Sample 65 | GSM1386847 | disease state: control | subjects: heart-beating, brain-dead donors | GSE57691 |
| GSM1386848 | Donor_Sample 66 | GSM1386848 | disease state: control | subjects: heart-beating, brain-dead donors | GSE57691 |
| GSM1386849 | Donor_Sample 67 | GSM1386849 | disease state: control | subjects: heart-beating, brain-dead donors | GSE57691 |
| GSM1386850 | Donor_Sample 68 | GSM1386850 | disease state: control | subjects: heart-beating, brain-dead donors | GSE57691 |
| **GSE47472** | **title** | **geo_accession** | **characteristics_ch1** | **characteristics_ch1.1** | **GEO** |
| GSM1150689 | AAA neck (Sample 1) | GSM1150689 | subjects: patient with AAA undergoing open surgery to treat AAA | tissue type: non-aneurysmal AAA neck tissue | GSE47472 |
| GSM1150690 | AAA neck (Sample 2) | GSM1150690 | subjects: patient with AAA undergoing open surgery to treat AAA | tissue type: non-aneurysmal AAA neck tissue | GSE47472 |
| GSM1150691 | AAA neck (Sample 3) | GSM1150691 | subjects: patient with AAA undergoing open surgery to treat AAA | tissue type: non-aneurysmal AAA neck tissue | GSE47472 |
| GSM1150692 | AAA neck (Sample 4) | GSM1150692 | subjects: patient with AAA undergoing open surgery to treat AAA | tissue type: non-aneurysmal AAA neck tissue | GSE47472 |
| GSM1150693 | AAA neck (Sample 5) | GSM1150693 | subjects: patient with AAA undergoing open surgery to treat AAA | tissue type: non-aneurysmal AAA neck tissue | GSE47472 |
| GSM1150694 | AAA neck (Sample 6) | GSM1150694 | subjects: patient with AAA undergoing open surgery to treat AAA | tissue type: non-aneurysmal AAA neck tissue | GSE47472 |
| GSM1150695 | AAA neck (Sample 7) | GSM1150695 | subjects: patient with AAA undergoing open surgery to treat AAA | tissue type: non-aneurysmal AAA neck tissue | GSE47472 |
| GSM1150696 | AAA neck (Sample 8) | GSM1150696 | subjects: patient with AAA undergoing open surgery to treat AAA | tissue type: non-aneurysmal AAA neck tissue | GSE47472 |
| GSM1150697 | AAA neck (Sample 9) | GSM1150697 | subjects: patient with AAA undergoing open surgery to treat AAA | tissue type: non-aneurysmal AAA neck tissue | GSE47472 |
| GSM1150698 | AAA neck (Sample 10) | GSM1150698 | subjects: patient with AAA undergoing open surgery to treat AAA | tissue type: non-aneurysmal AAA neck tissue | GSE47472 |
| GSM1150699 | AAA neck (Sample 11) | GSM1150699 | subjects: patient with AAA undergoing open surgery to treat AAA | tissue type: non-aneurysmal AAA neck tissue | GSE47472 |
| GSM1150700 | AAA neck (Sample 12) | GSM1150700 | subjects: patient with AAA undergoing open surgery to treat AAA | tissue type: non-aneurysmal AAA neck tissue | GSE47472 |
| GSM1150701 | AAA neck (Sample 13) | GSM1150701 | subjects: patient with AAA undergoing open surgery to treat AAA | tissue type: non-aneurysmal AAA neck tissue | GSE47472 |
| GSM1150702 | AAA neck (Sample 14) | GSM1150702 | subjects: patient with AAA undergoing open surgery to treat AAA | tissue type: non-aneurysmal AAA neck tissue | GSE47472 |
| GSM1150703 | Donor (Sample 15) | GSM1150703 | subjects: heart-beating, brain-dead donor | tissue type: normal aortic tissue | GSE47472 |
| GSM1150704 | Donor (Sample 16) | GSM1150704 | subjects: heart-beating, brain-dead donor | tissue type: normal aortic tissue | GSE47472 |
| GSM1150705 | Donor (Sample 17) | GSM1150705 | subjects: heart-beating, brain-dead donor | tissue type: normal aortic tissue | GSE47472 |
| GSM1150706 | Donor (Sample 18) | GSM1150706 | subjects: heart-beating, brain-dead donor | tissue type: normal aortic tissue | GSE47472 |
| GSM1150707 | Donor (Sample 19) | GSM1150707 | subjects: heart-beating, brain-dead donor | tissue type: normal aortic tissue | GSE47472 |
| GSM1150708 | Donor (Sample 20) | GSM1150708 | subjects: heart-beating, brain-dead donor | tissue type: normal aortic tissue | GSE47472 |
| GSM1150709 | Donor (Sample 21) | GSM1150709 | subjects: heart-beating, brain-dead donor | tissue type: normal aortic tissue | GSE47472 |
| GSM1150710 | Donor (Sample 22) | GSM1150710 | subjects: heart-beating, brain-dead donor | tissue type: normal aortic tissue | GSE47472 |

**Supplementary Table 3. The differential expression genes between AAA and normal patients from the AAA Combined Datasets.**

|  | **logFC** | **Average expression** | **T value** | **P. Value** | **Adjust P. Value** | **Bold** |
| --- | --- | --- | --- | --- | --- | --- |
| ANGPTL6 | 0.867251 | -0.02187 | 9.240762 | 1.74E-14 | 2.73E-10 | 22.45267 |
| ADAMTS6 | 0.539117 | -0.04396 | 9.063432 | 3.98E-14 | 2.73E-10 | 21.66192 |
| PLXDC1 | 0.55632 | -0.02797 | 9.045036 | 4.33E-14 | 2.73E-10 | 21.57986 |
| R3HCC1 | -0.67499 | -0.07151 | -8.96438 | 6.31E-14 | 2.98E-10 | 21.22004 |
| HES6 | -0.79719 | 0.020628 | -8.79029 | 1.42E-13 | 5.38E-10 | 20.44325 |
| CSNK2A2 | -1.00844 | -0.05456 | -8.69043 | 2.27E-13 | 6.12E-10 | 19.99777 |
| ZNF354B | 0.776326 | -0.07601 | 8.665738 | 2.54E-13 | 6.12E-10 | 19.88759 |
| PVT1 | 0.786531 | -0.12826 | 8.661647 | 2.59E-13 | 6.12E-10 | 19.86934 |
| OR4D1 | 0.780202 | 0.020294 | 8.567942 | 4.01E-13 | 8.42E-10 | 19.4514 |
| LINC01210 | 1.031805 | -0.05193 | 8.496757 | 5.59E-13 | 1.06E-09 | 19.13401 |
| TBXA2R | -0.65615 | 0.299248 | -8.45777 | 6.70E-13 | 1.07E-09 | 18.96022 |
| KLF15 | -0.79723 | -0.11929 | -8.45504 | 6.78E-13 | 1.07E-09 | 18.94808 |
| ZNF462 | -1.46481 | -0.0982 | -8.36674 | 1.02E-12 | 1.49E-09 | 18.55462 |
| UBL5 | -0.66551 | 0.209138 | -8.32595 | 1.24E-12 | 1.67E-09 | 18.37296 |
| BTC | -0.75567 | -0.14906 | -8.13593 | 2.99E-12 | 3.54E-09 | 17.52755 |
| PPP1R12C | -1.17363 | 0.055226 | -8.02619 | 4.98E-12 | 5.29E-09 | 17.04005 |
| GUCY2F | 0.605424 | -0.09531 | 8.023871 | 5.03E-12 | 5.29E-09 | 17.02976 |
| FOXP4 | 0.520862 | 0.119129 | 7.969832 | 6.46E-12 | 6.36E-09 | 16.78996 |
| ZNF324B | 0.628616 | -0.08788 | 7.961009 | 6.73E-12 | 6.36E-09 | 16.75083 |
| HBA2 | 1.903505 | 0.271811 | 7.872515 | 1.01E-11 | 8.72E-09 | 16.3586 |
| EFCC1 | 0.765363 | -0.13263 | 7.823302 | 1.27E-11 | 1.05E-08 | 16.1407 |
| DKFZP564C152 | -0.91924 | -0.02439 | -7.79919 | 1.42E-11 | 1.12E-08 | 16.03401 |
| MELK | 0.7024 | 0.151969 | 7.752761 | 1.76E-11 | 1.33E-08 | 15.8287 |
| MRGBP | -0.74682 | -0.08106 | -7.70212 | 2.23E-11 | 1.62E-08 | 15.60496 |
| TRIM58 | 0.764203 | -0.02813 | 7.651266 | 2.82E-11 | 1.97E-08 | 15.38048 |
| HSBP1 | -0.70179 | -0.00622 | -7.61756 | 3.29E-11 | 2.20E-08 | 15.23184 |
| WDR82 | -1.64667 | -0.01773 | -7.612 | 3.37E-11 | 2.20E-08 | 15.20733 |
| METTL22 | -0.59952 | 0.018665 | -7.58464 | 3.83E-11 | 2.41E-08 | 15.08675 |
| CYP4F3 | 0.552942 | 0.138303 | 7.566301 | 4.16E-11 | 2.54E-08 | 15.00597 |
| C8orf59 | -1.17642 | -0.20017 | -7.47678 | 6.28E-11 | 3.60E-08 | 14.61213 |
| NDUFS3 | -0.81641 | -0.075 | -7.46948 | 6.50E-11 | 3.61E-08 | 14.58002 |
| NXPH3 | -0.83682 | -0.13054 | -7.42844 | 7.84E-11 | 4.24E-08 | 14.39978 |
| CLN5 | -0.92083 | -0.02056 | -7.40408 | 8.77E-11 | 4.60E-08 | 14.2929 |
| FGF9 | 0.578332 | 0.219723 | 7.351578 | 1.11E-10 | 5.57E-08 | 14.06271 |
| HBB | 1.731428 | -0.00287 | 7.350636 | 1.12E-10 | 5.57E-08 | 14.05858 |
| RSL1D1 | -0.71705 | -0.07672 | -7.30721 | 1.37E-10 | 6.45E-08 | 13.86843 |
| HDAC7 | 0.535361 | 0.010517 | 7.29927 | 1.42E-10 | 6.53E-08 | 13.8337 |
| MFHAS1 | -0.81993 | -0.02374 | -7.25872 | 1.70E-10 | 7.67E-08 | 13.6564 |
| EPB41L1 | 0.515388 | 0.150089 | 7.176766 | 2.48E-10 | 1.06E-07 | 13.29869 |
| ARG2 | -0.56981 | -0.08093 | -7.16428 | 2.62E-10 | 1.10E-07 | 13.24429 |
| TRPC1 | -0.86858 | -0.08145 | -7.12931 | 3.07E-10 | 1.24E-07 | 13.09195 |
| GFOD1 | -0.91518 | -0.14882 | -7.09052 | 3.66E-10 | 1.43E-07 | 12.92323 |
| COL13A1 | -0.96107 | -0.09875 | -7.08502 | 3.76E-10 | 1.43E-07 | 12.89928 |
| FOXO1 | -0.92497 | -0.01253 | -7.08297 | 3.79E-10 | 1.43E-07 | 12.89041 |
| NR2F6 | -1.06438 | -0.05617 | -7.05425 | 4.32E-10 | 1.60E-07 | 12.76561 |
| LOC101928030 | 0.513543 | -0.0152 | 7.042171 | 4.56E-10 | 1.63E-07 | 12.71318 |
| CDK19 | -0.88003 | -0.01552 | -7.0289 | 4.85E-10 | 1.70E-07 | 12.65558 |
| CTBP1 | -0.55041 | 0.215761 | -7.01786 | 5.10E-10 | 1.71E-07 | 12.60771 |
| ITGB1BP1 | -0.78374 | 0.218694 | -7.01701 | 5.12E-10 | 1.71E-07 | 12.60401 |
| GGNBP2 | -0.88894 | -0.04026 | -7.0158 | 5.14E-10 | 1.71E-07 | 12.59878 |
| GRK5 | -0.56777 | 0.121399 | -7.00727 | 5.35E-10 | 1.74E-07 | 12.56179 |
| MRPL24 | -0.61858 | 0.080137 | -6.99791 | 5.58E-10 | 1.76E-07 | 12.52119 |
| CXXC5 | -1.08325 | 0.010488 | -6.99768 | 5.58E-10 | 1.76E-07 | 12.52021 |
| COL9A3 | 0.536197 | -0.09515 | 6.981399 | 6.01E-10 | 1.86E-07 | 12.44966 |
| FXR2 | -0.50838 | -0.03461 | -6.93688 | 7.35E-10 | 2.21E-07 | 12.25699 |
| TMEM229B | 0.613096 | -0.08108 | 6.915926 | 8.08E-10 | 2.38E-07 | 12.16639 |
| ETFB | -0.53046 | 0.29403 | -6.91329 | 8.18E-10 | 2.38E-07 | 12.15501 |
| PDZK1IP1 | 0.520239 | -0.03611 | 6.90496 | 8.49E-10 | 2.39E-07 | 12.11902 |
| PAIP1 | 0.537551 | 0.19412 | 6.904498 | 8.51E-10 | 2.39E-07 | 12.11702 |
| VGF | 0.858927 | -0.02554 | 6.901778 | 8.61E-10 | 2.39E-07 | 12.10527 |
| SSTR3 | 0.554966 | 0.101844 | 6.897882 | 8.77E-10 | 2.40E-07 | 12.08844 |
| KDELR1 | -0.64815 | -0.09524 | -6.89319 | 8.95E-10 | 2.42E-07 | 12.06817 |
| MKRN2 | -0.82319 | -0.13206 | -6.85819 | 1.05E-09 | 2.75E-07 | 11.91717 |
| ACOT2 | -0.83476 | 0.312321 | -6.84728 | 1.10E-09 | 2.85E-07 | 11.87014 |
| CCDC155 | 0.528321 | -0.08671 | 6.842831 | 1.12E-09 | 2.87E-07 | 11.85095 |
| ZHX3 | -0.65083 | -0.04823 | -6.82792 | 1.20E-09 | 2.99E-07 | 11.7867 |
| KLLN | 0.730331 | -0.08082 | 6.809145 | 1.31E-09 | 3.17E-07 | 11.70588 |
| LGR5 | 0.708021 | -0.15805 | 6.805841 | 1.33E-09 | 3.18E-07 | 11.69166 |
| HMGB3 | 0.510212 | -0.04443 | 6.788276 | 1.44E-09 | 3.39E-07 | 11.61611 |
| LRRC8A | -0.61311 | -0.06659 | -6.75953 | 1.63E-09 | 3.77E-07 | 11.49257 |
| CARM1 | -1.07736 | 0.06846 | -6.74411 | 1.75E-09 | 3.98E-07 | 11.42633 |
| OR8B12 | 0.646404 | -0.07203 | 6.741684 | 1.77E-09 | 3.98E-07 | 11.41594 |
| ELOVL3 | 0.637541 | -0.13771 | 6.720278 | 1.95E-09 | 4.33E-07 | 11.32411 |
| FADS6 | 0.524481 | 0.013918 | 6.715451 | 1.99E-09 | 4.37E-07 | 11.30341 |
| CHCHD3 | -0.53545 | 0.016396 | -6.71085 | 2.03E-09 | 4.37E-07 | 11.28367 |
| NACC2 | -0.73641 | 0.273141 | -6.71057 | 2.03E-09 | 4.37E-07 | 11.28249 |
| NCK2 | -0.64804 | 0.353593 | -6.69882 | 2.14E-09 | 4.54E-07 | 11.23213 |
| SYNC | -0.91602 | 0.059444 | -6.69713 | 2.16E-09 | 4.54E-07 | 11.22488 |
| MPC2 | -0.8234 | -0.02027 | -6.67781 | 2.36E-09 | 4.84E-07 | 11.14217 |
| ZSWIM1 | -0.6017 | -0.0658 | -6.66664 | 2.48E-09 | 4.98E-07 | 11.09438 |
| ARHGEF12 | -0.63194 | -0.06495 | -6.62631 | 2.97E-09 | 5.78E-07 | 10.92196 |
| LINC00116 | -0.54341 | -0.05746 | -6.62333 | 3.01E-09 | 5.80E-07 | 10.90926 |
| HTR1F | 0.789823 | -0.14515 | 6.579391 | 3.66E-09 | 6.78E-07 | 10.7218 |
| CPNE8 | -0.63135 | 0.020022 | -6.56744 | 3.86E-09 | 7.08E-07 | 10.67088 |
| NCF1B | 0.638721 | -0.10522 | 6.563431 | 3.93E-09 | 7.14E-07 | 10.65381 |
| GPRC5C | -0.72628 | 0.406598 | -6.54786 | 4.21E-09 | 7.58E-07 | 10.58751 |
| RCN1 | -0.68668 | 0.34404 | -6.53445 | 4.47E-09 | 7.89E-07 | 10.53048 |
| ZC3H11A | 0.624865 | -0.14056 | 6.518548 | 4.79E-09 | 8.39E-07 | 10.46288 |
| LSM2 | -1.21993 | -0.03806 | -6.50922 | 5.00E-09 | 8.59E-07 | 10.42327 |
| FOXL1 | 0.831915 | -0.16022 | 6.498101 | 5.25E-09 | 8.94E-07 | 10.37603 |
| PAFAH2 | -0.56523 | -0.13723 | -6.49282 | 5.37E-09 | 9.03E-07 | 10.35363 |
| QARS | -0.64138 | 0.011711 | -6.49181 | 5.40E-09 | 9.03E-07 | 10.34931 |
| ECI1 | -0.56588 | -0.0402 | -6.47417 | 5.84E-09 | 9.63E-07 | 10.27448 |
| MT1M | -1.26228 | -0.00297 | -6.47342 | 5.86E-09 | 9.63E-07 | 10.27133 |
| C2CD2 | -0.55277 | 0.192088 | -6.46728 | 6.02E-09 | 9.81E-07 | 10.24529 |
| PRAMEF9 | 0.627477 | -0.1673 | 6.444698 | 6.65E-09 | 1.07E-06 | 10.14962 |
| RBM23 | -0.78784 | 0.086498 | -6.43655 | 6.90E-09 | 1.09E-06 | 10.11512 |
| ZBTB16 | -0.85056 | 0.331618 | -6.4223 | 7.35E-09 | 1.12E-06 | 10.05484 |
| OR2AG2 | 0.500375 | -0.08065 | 6.398941 | 8.14E-09 | 1.23E-06 | 9.956093 |
| LLGL1 | -0.79051 | -0.17235 | -6.33699 | 1.07E-08 | 1.56E-06 | 9.694817 |
| ATP6V1C1 | -0.5309 | 0.239663 | -6.31948 | 1.16E-08 | 1.67E-06 | 9.621117 |
| ITGB3BP | -0.52716 | 0.016372 | -6.30981 | 1.21E-08 | 1.73E-06 | 9.580453 |
| KLF9 | -0.89661 | -0.12781 | -6.30813 | 1.22E-08 | 1.73E-06 | 9.573376 |
| GBP5 | 0.840954 | 0.055912 | 6.296087 | 1.28E-08 | 1.81E-06 | 9.522768 |
| SMCO3 | 0.578479 | -0.07742 | 6.293659 | 1.30E-08 | 1.81E-06 | 9.512564 |
| ITGA10 | -1.02083 | -0.04068 | -6.27762 | 1.39E-08 | 1.89E-06 | 9.445216 |
| ATF4 | -0.50511 | 0.250577 | -6.2771 | 1.39E-08 | 1.89E-06 | 9.443038 |
| GABRR3 | 0.737396 | -0.07882 | 6.26686 | 1.46E-08 | 1.97E-06 | 9.40007 |
| LINC01144 | 0.691096 | -0.11465 | 6.24367 | 1.61E-08 | 2.15E-06 | 9.302854 |
| PMM1 | -0.59371 | -0.03416 | -6.22928 | 1.72E-08 | 2.27E-06 | 9.242616 |
| ENKD1 | -0.67813 | -0.03453 | -6.21684 | 1.81E-08 | 2.37E-06 | 9.190524 |
| BDKRB2 | 0.574207 | -0.06711 | 6.211284 | 1.86E-08 | 2.41E-06 | 9.167307 |
| LRFN3 | -0.73403 | -0.07448 | -6.20853 | 1.88E-08 | 2.41E-06 | 9.155784 |
| ARL17A | 1.170403 | 0.059691 | 6.206863 | 1.90E-08 | 2.41E-06 | 9.148822 |
| BBX | -0.85099 | -0.15071 | -6.20367 | 1.92E-08 | 2.42E-06 | 9.135459 |
| TSEN15 | -0.95107 | -0.02589 | -6.18559 | 2.08E-08 | 2.56E-06 | 9.05996 |
| TUFM | -0.61096 | -0.01601 | -6.17506 | 2.18E-08 | 2.66E-06 | 9.015995 |
| SPATA6L | 0.595492 | -0.05874 | 6.1706 | 2.22E-08 | 2.69E-06 | 8.99738 |
| PFDN1 | -0.72112 | -0.07263 | -6.1661 | 2.27E-08 | 2.73E-06 | 8.978605 |
| PRR7 | 0.561711 | -0.01582 | 6.162184 | 2.30E-08 | 2.74E-06 | 8.962281 |
| PCMT1 | -0.79509 | -0.07643 | -6.11306 | 2.85E-08 | 3.37E-06 | 8.757765 |
| OSBPL5 | -0.63925 | 0.092696 | -6.07325 | 3.39E-08 | 3.89E-06 | 8.592423 |
| GPR27 | 0.551535 | -0.08353 | 6.071606 | 3.42E-08 | 3.89E-06 | 8.58562 |
| SNU13 | -0.57253 | 0.192114 | -6.06429 | 3.53E-08 | 4.00E-06 | 8.555306 |
| HEATR9 | 0.724642 | -0.11633 | 6.055862 | 3.66E-08 | 4.03E-06 | 8.520362 |
| PIP4K2C | -0.54946 | -0.00178 | -6.05534 | 3.67E-08 | 4.03E-06 | 8.518196 |
| HK1 | -0.54392 | 0.380601 | -6.05089 | 3.74E-08 | 4.08E-06 | 8.499774 |
| TXNDC15 | -0.58046 | -0.05787 | -6.04787 | 3.79E-08 | 4.09E-06 | 8.487274 |
| NELL2 | 0.734237 | 0.02913 | 6.043287 | 3.87E-08 | 4.13E-06 | 8.468286 |
| TMEM14A | -0.88624 | 0.069237 | -6.04065 | 3.91E-08 | 4.15E-06 | 8.457366 |
| PLXNB1 | -0.67594 | -0.04614 | -6.02268 | 4.23E-08 | 4.37E-06 | 8.38302 |
| TRAPPC4 | -0.78093 | -0.23542 | -6.01223 | 4.42E-08 | 4.53E-06 | 8.33986 |
| PLA2G16 | -0.73531 | -0.0237 | -6.01147 | 4.44E-08 | 4.53E-06 | 8.336708 |
| SUN2 | -0.80054 | -0.16568 | -5.99598 | 4.74E-08 | 4.80E-06 | 8.272752 |
| C22orf23 | 0.564113 | -0.02846 | 5.990404 | 4.86E-08 | 4.86E-06 | 8.249733 |
| OR2A7 | -0.66712 | 0.064362 | -5.98647 | 4.94E-08 | 4.92E-06 | 8.233523 |
| DNTTIP1 | -0.54855 | -0.04063 | -5.98502 | 4.97E-08 | 4.92E-06 | 8.22754 |
| BIN2 | 0.514221 | -0.00749 | 5.977674 | 5.14E-08 | 5.06E-06 | 8.197235 |
| PLCH1 | 0.539983 | -0.11284 | 5.967387 | 5.37E-08 | 5.23E-06 | 8.154844 |
| KIAA1456 | 0.684634 | -0.12681 | 5.952652 | 5.72E-08 | 5.49E-06 | 8.094174 |
| DNM1L | -0.52842 | 0.212097 | -5.94513 | 5.91E-08 | 5.59E-06 | 8.06324 |
| RBPJL | 0.602921 | -0.10599 | 5.942804 | 5.97E-08 | 5.61E-06 | 8.053657 |
| MS4A10 | 0.598965 | -0.1119 | 5.940432 | 6.03E-08 | 5.64E-06 | 8.0439 |
| LOC100288893 | 0.824535 | -0.04493 | 5.939267 | 6.06E-08 | 5.64E-06 | 8.039111 |
| DIMT1 | -0.77127 | -0.06428 | -5.93041 | 6.30E-08 | 5.81E-06 | 8.002698 |
| TMED4 | -0.5519 | 0.109056 | -5.91953 | 6.60E-08 | 6.06E-06 | 7.958002 |
| PACSIN2 | -0.79443 | -0.19368 | -5.90943 | 6.89E-08 | 6.29E-06 | 7.916536 |
| TSPAN2 | -0.51018 | 0.127298 | -5.89818 | 7.23E-08 | 6.51E-06 | 7.870417 |
| MCAM | -0.95114 | 0.00629 | -5.89527 | 7.32E-08 | 6.56E-06 | 7.858453 |
| ZNF177 | 0.54228 | -0.0263 | 5.866107 | 8.30E-08 | 7.33E-06 | 7.739027 |
| MRPS16 | -0.60204 | -0.0838 | -5.85746 | 8.62E-08 | 7.54E-06 | 7.703661 |
| JAZF1 | -0.70468 | -0.08124 | -5.85516 | 8.70E-08 | 7.58E-06 | 7.694249 |
| DUSP3 | -0.89582 | -0.25761 | -5.8498 | 8.90E-08 | 7.69E-06 | 7.67235 |
| NABP2 | -0.64542 | -0.09993 | -5.84802 | 8.97E-08 | 7.71E-06 | 7.665091 |
| JAG1 | -0.98574 | -0.20499 | -5.8345 | 9.51E-08 | 8.02E-06 | 7.609846 |
| IL2RB | 0.871823 | 0.131334 | 5.826096 | 9.85E-08 | 8.24E-06 | 7.575549 |
| MTMR6 | -0.77623 | 0.005071 | -5.82409 | 9.94E-08 | 8.28E-06 | 7.567366 |
| HPS6 | -0.68317 | -0.18836 | -5.8161 | 1.03E-07 | 8.45E-06 | 7.534786 |
| ICMT | -0.58858 | -0.07991 | -5.81417 | 1.04E-07 | 8.49E-06 | 7.526893 |
| LSM3 | -0.80393 | 0.122427 | -5.81228 | 1.05E-07 | 8.52E-06 | 7.519222 |
| CLMP | -0.683 | 0.056925 | -5.80507 | 1.08E-07 | 8.75E-06 | 7.489824 |
| CNIH1 | -0.87469 | 0.262099 | -5.79162 | 1.14E-07 | 9.15E-06 | 7.435066 |
| LOC283713 | 0.652399 | -0.0757 | 5.787493 | 1.16E-07 | 9.27E-06 | 7.418264 |
| LGR6 | -0.54271 | 0.371862 | -5.78604 | 1.17E-07 | 9.29E-06 | 7.41234 |
| DEFB4A | 0.5332 | -0.13185 | 5.775441 | 1.22E-07 | 9.64E-06 | 7.369246 |
| AXIN2 | -0.70206 | -0.02152 | -5.76084 | 1.30E-07 | 1.02E-05 | 7.309906 |
| LINC00938 | -0.69972 | -0.15543 | -5.75813 | 1.32E-07 | 1.02E-05 | 7.298899 |
| ENAM | 0.509851 | -0.09174 | 5.752017 | 1.35E-07 | 1.04E-05 | 7.274102 |
| SUGT1P3 | 0.526322 | -0.07233 | 5.744569 | 1.40E-07 | 1.06E-05 | 7.243882 |
| TPTE2P5 | 0.526322 | -0.07233 | 5.744569 | 1.40E-07 | 1.06E-05 | 7.243882 |
| ZNF791 | -0.67035 | 0.025306 | -5.74186 | 1.41E-07 | 1.07E-05 | 7.232907 |
| MRAS | 0.602331 | -0.05188 | 5.736581 | 1.44E-07 | 1.09E-05 | 7.211491 |
| SOHLH1 | 0.562418 | -0.12085 | 5.730791 | 1.48E-07 | 1.11E-05 | 7.188025 |
| CBX6 | -0.88526 | -0.15437 | -5.72808 | 1.50E-07 | 1.11E-05 | 7.177037 |
| PHF2 | -0.59187 | 0.297083 | -5.7256 | 1.51E-07 | 1.12E-05 | 7.167008 |
| NFIA | -0.89463 | 0.13322 | -5.72391 | 1.52E-07 | 1.12E-05 | 7.160154 |
| ALOX15B | -0.98864 | 0.427409 | -5.71722 | 1.57E-07 | 1.14E-05 | 7.13307 |
| KATNBL1 | -0.60434 | 0.003936 | -5.71193 | 1.60E-07 | 1.16E-05 | 7.111648 |
| TLK1 | -0.62641 | -0.02977 | -5.71134 | 1.61E-07 | 1.16E-05 | 7.109267 |
| FGFBP2 | 0.761337 | 0.016842 | 5.706911 | 1.64E-07 | 1.17E-05 | 7.091349 |
| PCNT | -0.51638 | 0.03566 | -5.69229 | 1.74E-07 | 1.22E-05 | 7.032234 |
| OR1L8 | 0.561197 | -0.10911 | 5.690107 | 1.76E-07 | 1.23E-05 | 7.023424 |
| PSMC3 | -0.51793 | -0.13505 | -5.66135 | 1.99E-07 | 1.35E-05 | 6.907367 |
| FADD | -0.58154 | -0.09213 | -5.65821 | 2.01E-07 | 1.35E-05 | 6.894714 |
| ELK1 | -0.5529 | -0.043 | -5.64352 | 2.14E-07 | 1.41E-05 | 6.835548 |
| PSMG1 | -0.53542 | 0.070056 | -5.64189 | 2.16E-07 | 1.42E-05 | 6.828991 |
| DCUN1D3 | -0.79209 | 0.029849 | -5.62305 | 2.34E-07 | 1.51E-05 | 6.753223 |
| NARF | -0.55544 | -0.05045 | -5.61756 | 2.39E-07 | 1.52E-05 | 6.731178 |
| SMYD4 | -0.70996 | -0.00679 | -5.61403 | 2.43E-07 | 1.53E-05 | 6.717009 |
| H2AFJ | -0.60015 | -0.08313 | -5.60466 | 2.52E-07 | 1.58E-05 | 6.679373 |
| SHMT2 | -0.64462 | -0.11776 | -5.59667 | 2.61E-07 | 1.61E-05 | 6.64733 |
| CLASP1 | -0.72872 | -0.08208 | -5.58963 | 2.69E-07 | 1.65E-05 | 6.6191 |
| FEZ2 | -0.77873 | -0.00128 | -5.58919 | 2.69E-07 | 1.65E-05 | 6.617322 |
| CASKIN1 | 0.625952 | -0.08992 | 5.583574 | 2.76E-07 | 1.67E-05 | 6.594834 |
| FOXL2NB | 0.608543 | -0.0673 | 5.579788 | 2.80E-07 | 1.69E-05 | 6.579668 |
| TMEM181 | -0.94783 | -0.12481 | -5.57675 | 2.84E-07 | 1.70E-05 | 6.567499 |
| SETMAR | -0.5718 | -0.04862 | -5.57128 | 2.90E-07 | 1.71E-05 | 6.545606 |
| LOC100287837 | 0.567387 | -0.18831 | 5.57112 | 2.91E-07 | 1.71E-05 | 6.544966 |
| FBXO32 | -0.67366 | 0.407815 | -5.57111 | 2.91E-07 | 1.71E-05 | 6.544909 |
| ZFHX3 | -0.65035 | -0.04005 | -5.55768 | 3.08E-07 | 1.79E-05 | 6.491194 |
| FIGF | 0.599894 | -0.17475 | 5.555541 | 3.10E-07 | 1.80E-05 | 6.482654 |
| RRAGA | -0.64125 | -0.1076 | -5.5551 | 3.11E-07 | 1.80E-05 | 6.480872 |
| ANP32B | -0.64656 | -0.13453 | -5.55117 | 3.16E-07 | 1.82E-05 | 6.465188 |
| GNG10 | -0.68699 | 0.215972 | -5.55 | 3.18E-07 | 1.83E-05 | 6.46051 |
| DYNLRB1 | -0.6402 | 0.263272 | -5.54638 | 3.22E-07 | 1.85E-05 | 6.446036 |
| PGRMC2 | -0.68834 | -0.00252 | -5.54109 | 3.30E-07 | 1.88E-05 | 6.42491 |
| ADSL | -0.59624 | -0.07609 | -5.53014 | 3.45E-07 | 1.95E-05 | 6.381239 |
| MXD4 | -0.65644 | -0.10919 | -5.52487 | 3.53E-07 | 1.99E-05 | 6.360217 |
| MGMT | -0.63816 | -0.15937 | -5.51679 | 3.65E-07 | 2.05E-05 | 6.328004 |
| ATG14 | -0.69761 | -0.07169 | -5.50769 | 3.79E-07 | 2.12E-05 | 6.291745 |
| MRPS28 | -0.57943 | -0.19302 | -5.50654 | 3.81E-07 | 2.13E-05 | 6.28716 |
| TIMM22 | -0.63741 | -0.05081 | -5.48782 | 4.12E-07 | 2.26E-05 | 6.212725 |
| RAB11FIP3 | -0.69684 | -0.02591 | -5.48754 | 4.13E-07 | 2.26E-05 | 6.211598 |
| C3orf70 | -0.83631 | 0.209306 | -5.48582 | 4.16E-07 | 2.27E-05 | 6.204752 |
| DDRGK1 | -0.56678 | -0.12151 | -5.48112 | 4.24E-07 | 2.31E-05 | 6.186088 |
| ZNF260 | -0.52025 | -0.10728 | -5.48003 | 4.26E-07 | 2.31E-05 | 6.181774 |
| AFF4 | 0.774554 | 0.00774 | 5.478389 | 4.29E-07 | 2.32E-05 | 6.175237 |
| SLC30A7 | -0.58197 | -0.05311 | -5.4672 | 4.49E-07 | 2.42E-05 | 6.130837 |
| ANKRD46 | -0.77279 | -0.09652 | -5.4562 | 4.70E-07 | 2.51E-05 | 6.087175 |
| MRPL37 | -0.5006 | -0.1239 | -5.44585 | 4.91E-07 | 2.59E-05 | 6.04617 |
| DENND6B | 0.543728 | 0.005872 | 5.443387 | 4.96E-07 | 2.61E-05 | 6.036425 |
| THAP11 | -0.64979 | -0.16102 | -5.43884 | 5.05E-07 | 2.65E-05 | 6.018428 |
| TAAR9 | 0.674513 | -0.04012 | 5.437742 | 5.08E-07 | 2.65E-05 | 6.014076 |
| SERPINC1 | 0.604559 | -0.16115 | 5.433679 | 5.16E-07 | 2.69E-05 | 5.997998 |
| PTPRG | -0.50283 | -0.04024 | -5.4227 | 5.41E-07 | 2.78E-05 | 5.954589 |
| PHB | -0.55118 | -0.16353 | -5.41998 | 5.47E-07 | 2.81E-05 | 5.943835 |
| CASP14 | 0.599891 | -0.12564 | 5.41178 | 5.66E-07 | 2.86E-05 | 5.911433 |
| MSTN | -0.93634 | 0.128046 | -5.4114 | 5.67E-07 | 2.86E-05 | 5.909927 |
| DLX2 | 0.626684 | -0.11185 | 5.37796 | 6.51E-07 | 3.18E-05 | 5.778075 |
| DNLZ | -0.5261 | 0.007688 | -5.36802 | 6.78E-07 | 3.28E-05 | 5.738955 |
| ZFAND1 | -0.52216 | 0.122505 | -5.36745 | 6.80E-07 | 3.28E-05 | 5.736706 |
| DRG1 | -0.6229 | -0.18862 | -5.3639 | 6.90E-07 | 3.31E-05 | 5.722739 |
| CCK | 0.61311 | -0.01759 | 5.362486 | 6.94E-07 | 3.32E-05 | 5.717187 |
| PRKRA | -0.67275 | -0.093 | -5.35173 | 7.25E-07 | 3.44E-05 | 5.674899 |
| KLK4 | 0.565839 | -0.12303 | 5.345004 | 7.46E-07 | 3.49E-05 | 5.648504 |
| FAM210B | -0.62256 | -0.09969 | -5.34194 | 7.55E-07 | 3.52E-05 | 5.63648 |
| LRRC39 | 0.571791 | -0.00133 | 5.332452 | 7.86E-07 | 3.59E-05 | 5.599255 |
| TLE1 | -0.65155 | -0.00375 | -5.31748 | 8.36E-07 | 3.75E-05 | 5.540589 |
| ZNF233 | 0.50843 | -0.01898 | 5.315731 | 8.42E-07 | 3.75E-05 | 5.533739 |
| LRRC47 | -0.57292 | -0.11097 | -5.31164 | 8.56E-07 | 3.81E-05 | 5.517714 |
| OR2T3 | 0.646036 | -0.13578 | 5.31027 | 8.61E-07 | 3.81E-05 | 5.512363 |
| PADI3 | 0.53779 | -0.15439 | 5.302553 | 8.89E-07 | 3.91E-05 | 5.482175 |
| NSMCE4A | -0.66995 | -0.1244 | -5.29693 | 9.09E-07 | 3.98E-05 | 5.460187 |
| MRPS22 | -0.55281 | -0.06217 | -5.29389 | 9.21E-07 | 4.01E-05 | 5.448315 |
| NPTX2 | -0.9135 | 0.031199 | -5.27733 | 9.86E-07 | 4.21E-05 | 5.383644 |
| SLC16A12 | 0.530912 | 0.171827 | 5.272587 | 1.01E-06 | 4.25E-05 | 5.365149 |
| PIGK | -0.67484 | -0.00375 | -5.26831 | 1.02E-06 | 4.31E-05 | 5.348456 |
| CAND2 | -0.57917 | -0.0701 | -5.26631 | 1.03E-06 | 4.31E-05 | 5.340678 |
| UBQLN4 | -0.56989 | -0.03202 | -5.26595 | 1.03E-06 | 4.31E-05 | 5.339281 |
| HSPB2 | -0.63263 | -0.13401 | -5.26477 | 1.04E-06 | 4.32E-05 | 5.334693 |
| PGRMC1 | -0.77851 | -0.20856 | -5.2638 | 1.04E-06 | 4.33E-05 | 5.330886 |
| PRSS35 | 0.518973 | -0.06789 | 5.260332 | 1.06E-06 | 4.38E-05 | 5.317388 |
| SNHG10 | 0.631618 | -0.05063 | 5.258539 | 1.06E-06 | 4.39E-05 | 5.310404 |
| ADORA2A | 0.530283 | 0.102462 | 5.250532 | 1.10E-06 | 4.49E-05 | 5.279231 |
| SATB1 | 0.547929 | 0.065001 | 5.247904 | 1.11E-06 | 4.52E-05 | 5.269006 |
| FAM234A | -0.53152 | 0.010193 | -5.24643 | 1.12E-06 | 4.54E-05 | 5.263258 |
| RBM15B | 0.62963 | -0.07803 | 5.239955 | 1.15E-06 | 4.63E-05 | 5.238088 |
| ZNF581 | -0.60935 | 0.007395 | -5.23987 | 1.15E-06 | 4.63E-05 | 5.237775 |
| RNF220 | -0.55571 | -0.17264 | -5.23572 | 1.17E-06 | 4.67E-05 | 5.221621 |
| KIFC2 | 0.820219 | 0.065038 | 5.234584 | 1.17E-06 | 4.68E-05 | 5.217213 |
| CHCHD5 | -0.5126 | -0.15273 | -5.23203 | 1.19E-06 | 4.69E-05 | 5.207305 |
| AHR | 0.522528 | 0.193136 | 5.231004 | 1.19E-06 | 4.69E-05 | 5.203305 |
| TPRG1L | -0.55812 | -0.03422 | -5.22334 | 1.23E-06 | 4.81E-05 | 5.173532 |
| SRBD1 | -0.65035 | -0.02104 | -5.22197 | 1.24E-06 | 4.83E-05 | 5.16824 |
| P2RY8 | 0.788427 | 0.204755 | 5.220348 | 1.24E-06 | 4.84E-05 | 5.161932 |
| SGTA | -0.59806 | -0.01512 | -5.21959 | 1.25E-06 | 4.85E-05 | 5.158999 |
| RAB2B | -0.65141 | -0.00626 | -5.21463 | 1.27E-06 | 4.93E-05 | 5.139766 |
| COMTD1 | -0.62383 | 0.019746 | -5.20597 | 1.32E-06 | 5.06E-05 | 5.106182 |
| REXO4 | -0.54118 | -0.02091 | -5.19687 | 1.37E-06 | 5.22E-05 | 5.070933 |
| MECOM | -0.53568 | 0.282995 | -5.19487 | 1.38E-06 | 5.23E-05 | 5.063173 |
| CCDC102A | -0.76905 | -0.16232 | -5.19422 | 1.39E-06 | 5.24E-05 | 5.060653 |
| SPTSSA | -0.62284 | -0.16454 | -5.19267 | 1.39E-06 | 5.26E-05 | 5.054684 |
| SAMD4B | -0.51543 | -0.01567 | -5.19061 | 1.41E-06 | 5.29E-05 | 5.046704 |
| MAP3K5 | -0.57982 | -0.10455 | -5.19021 | 1.41E-06 | 5.29E-05 | 5.04514 |
| UQCRQ | -0.71165 | -0.17026 | -5.18938 | 1.41E-06 | 5.29E-05 | 5.041956 |
| GNB4 | -0.50597 | 0.096538 | -5.18914 | 1.41E-06 | 5.29E-05 | 5.041029 |
| ADGRE3 | 0.513538 | 0.185565 | 5.186332 | 1.43E-06 | 5.34E-05 | 5.030148 |
| FOXD1 | -0.54749 | -0.1423 | -5.18553 | 1.43E-06 | 5.35E-05 | 5.027054 |
| DRD3 | -0.5512 | 0.204385 | -5.17326 | 1.51E-06 | 5.57E-05 | 4.979608 |
| FZD8 | 0.549545 | -0.05906 | 5.172212 | 1.51E-06 | 5.58E-05 | 4.975571 |
| SPECC1L | -0.66687 | -0.1487 | -5.17154 | 1.52E-06 | 5.58E-05 | 4.972962 |
| SSFA2 | 0.653939 | -0.00489 | 5.168367 | 1.54E-06 | 5.63E-05 | 4.960725 |
| BACH2 | 0.687845 | 0.380011 | 5.168257 | 1.54E-06 | 5.63E-05 | 4.960298 |
| NDUFA9 | -0.53518 | -0.14766 | -5.16629 | 1.55E-06 | 5.66E-05 | 4.952699 |
| FAM214B | -0.52843 | 0.040811 | -5.16437 | 1.56E-06 | 5.69E-05 | 4.945309 |
| PADI4 | 0.984134 | 0.283965 | 5.163751 | 1.57E-06 | 5.69E-05 | 4.942906 |
| GTF2A2 | -0.56898 | -0.10149 | -5.16189 | 1.58E-06 | 5.71E-05 | 4.935715 |
| WDR36 | -0.56299 | -0.14549 | -5.15714 | 1.61E-06 | 5.80E-05 | 4.917406 |
| FAM50A | -0.55401 | -0.11676 | -5.15639 | 1.62E-06 | 5.81E-05 | 4.914492 |
| ESAM | -0.58133 | -0.07197 | -5.14044 | 1.72E-06 | 6.09E-05 | 4.853041 |
| LINC00626 | 0.584481 | 0.030441 | 5.138773 | 1.74E-06 | 6.12E-05 | 4.846629 |
| FKBP5 | -0.83968 | -0.01863 | -5.13063 | 1.79E-06 | 6.27E-05 | 4.815301 |
| SMUG1 | -0.55702 | -0.14372 | -5.12314 | 1.85E-06 | 6.38E-05 | 4.786485 |
| NAP1L4 | -0.52208 | -0.1015 | -5.1191 | 1.88E-06 | 6.46E-05 | 4.770966 |
| DAAM1 | -0.52533 | 0.195282 | -5.1154 | 1.91E-06 | 6.52E-05 | 4.75674 |
| AIF1L | -0.6788 | -0.00096 | -5.10551 | 1.99E-06 | 6.66E-05 | 4.718776 |
| PHRF1 | 0.602148 | 0.069854 | 5.105138 | 1.99E-06 | 6.66E-05 | 4.717361 |
| FABP3 | -0.57364 | -0.02068 | -5.09541 | 2.07E-06 | 6.85E-05 | 4.680069 |
| SERPINB4 | -0.52224 | -0.02637 | -5.07629 | 2.23E-06 | 7.31E-05 | 4.606844 |
| GALM | -0.60714 | -0.03223 | -5.06384 | 2.35E-06 | 7.63E-05 | 4.559247 |
| PNMA1 | -0.50929 | -0.05133 | -5.06213 | 2.37E-06 | 7.66E-05 | 4.552708 |
| PBX2 | -0.67379 | -0.17037 | -5.06199 | 2.37E-06 | 7.66E-05 | 4.552162 |
| HIGD1A | -0.73372 | 0.244125 | -5.06053 | 2.38E-06 | 7.70E-05 | 4.546618 |
| SLC34A1 | 0.656603 | 0.008035 | 5.059667 | 2.39E-06 | 7.71E-05 | 4.543309 |
| HDHD2 | -0.66623 | -0.05221 | -5.05193 | 2.47E-06 | 7.85E-05 | 4.513762 |
| ACADL | -0.578 | -0.10975 | -5.05147 | 2.47E-06 | 7.85E-05 | 4.512006 |
| FBXW7 | 0.529186 | 0.572918 | 5.050225 | 2.48E-06 | 7.87E-05 | 4.507268 |
| DPY19L1 | -0.53939 | -0.13974 | -5.04306 | 2.55E-06 | 8.06E-05 | 4.479927 |
| RNF170 | -0.68971 | -0.09711 | -5.029 | 2.70E-06 | 8.47E-05 | 4.426385 |
| PARVA | -0.67542 | -0.0854 | -5.02539 | 2.74E-06 | 8.54E-05 | 4.412643 |
| C12orf29 | -0.51769 | -0.05216 | -5.02357 | 2.76E-06 | 8.57E-05 | 4.405721 |
| TRIM44 | -0.78422 | -0.10014 | -5.02288 | 2.77E-06 | 8.57E-05 | 4.403082 |
| FHOD1 | -0.77483 | 0.112412 | -5.00599 | 2.96E-06 | 8.93E-05 | 4.338891 |
| RPUSD4 | -0.5574 | -0.09954 | -5.00188 | 3.01E-06 | 9.02E-05 | 4.323309 |
| LSM7 | -0.7198 | -0.19539 | -5.00108 | 3.02E-06 | 9.02E-05 | 4.320236 |
| INSIG2 | -0.72032 | -0.08909 | -4.98164 | 3.27E-06 | 9.64E-05 | 4.246551 |
| MOAP1 | -0.53982 | -0.04678 | -4.97921 | 3.30E-06 | 9.69E-05 | 4.237347 |
| NDUFB2 | -0.51928 | -0.08493 | -4.97827 | 3.31E-06 | 9.70E-05 | 4.233794 |
| TMEM261 | -0.54218 | -0.01007 | -4.97781 | 3.32E-06 | 9.70E-05 | 4.232058 |
| SPDYE2 | 0.514792 | -0.00915 | 4.975085 | 3.36E-06 | 9.77E-05 | 4.221729 |
| RAB5C | -0.77 | 0.146409 | -4.96273 | 3.52E-06 | 0.000102 | 4.17501 |
| SPNS3 | 0.675968 | 0.064748 | 4.959734 | 3.57E-06 | 0.000103 | 4.163675 |
| STX1A | 0.544994 | -0.01635 | 4.957949 | 3.59E-06 | 0.000103 | 4.15693 |
| APOA4 | -0.53112 | 0.050319 | -4.95386 | 3.65E-06 | 0.000104 | 4.141501 |
| XXYLT1 | -0.51231 | -0.07663 | -4.95328 | 3.66E-06 | 0.000104 | 4.139312 |
| GORASP2 | -0.56045 | -0.10584 | -4.93909 | 3.87E-06 | 0.000109 | 4.085741 |
| YIPF6 | -0.74643 | -0.1597 | -4.9356 | 3.93E-06 | 0.00011 | 4.072597 |
| RPL27A | -0.50399 | -0.19495 | -4.90177 | 4.49E-06 | 0.000122 | 3.945343 |
| ALDH1L1 | -0.60345 | -0.19602 | -4.89551 | 4.61E-06 | 0.000124 | 3.921841 |
| TBX2 | -0.53007 | -0.1204 | -4.88987 | 4.71E-06 | 0.000127 | 3.900711 |
| ADAM18 | -0.5461 | 0.00512 | -4.88946 | 4.72E-06 | 0.000127 | 3.899154 |
| ZSCAN18 | -0.63598 | -0.02044 | -4.88436 | 4.81E-06 | 0.000128 | 3.880032 |
| LOC105372672 | 0.507367 | -0.06365 | 4.881153 | 4.88E-06 | 0.00013 | 3.868026 |
| ACTG1 | -0.71579 | 0.416341 | -4.87945 | 4.91E-06 | 0.00013 | 3.861652 |
| PNISR | 0.773119 | 0.148452 | 4.875473 | 4.99E-06 | 0.000132 | 3.846754 |
| STARD8 | -0.51395 | -0.1203 | -4.87396 | 5.02E-06 | 0.000132 | 3.841086 |
| HSPB11 | -0.65334 | -0.22591 | -4.8727 | 5.04E-06 | 0.000133 | 3.836366 |
| ZMAT2 | -0.57763 | -0.09243 | -4.86877 | 5.12E-06 | 0.000134 | 3.821688 |
| KIAA1841 | 0.538913 | 0.002771 | 4.868593 | 5.12E-06 | 0.000134 | 3.821009 |
| ZNF689 | -0.53923 | -0.10326 | -4.86811 | 5.13E-06 | 0.000134 | 3.819198 |
| LSM14A | -0.57813 | -0.12444 | -4.86583 | 5.18E-06 | 0.000135 | 3.810692 |
| RASGRP4 | 0.517929 | 0.083637 | 4.84704 | 5.58E-06 | 0.000143 | 3.740485 |
| EEF2K | -0.53139 | -0.07218 | -4.83531 | 5.84E-06 | 0.000148 | 3.696743 |
| BTBD6 | -0.51237 | -0.12826 | -4.83318 | 5.89E-06 | 0.000149 | 3.688795 |
| H1F0 | -0.74109 | -0.01967 | -4.82846 | 6.00E-06 | 0.000151 | 3.671229 |
| SETD4 | 0.612765 | -0.10966 | 4.828275 | 6.01E-06 | 0.000151 | 3.670541 |
| CRYBA4 | 0.505366 | -0.0812 | 4.822779 | 6.14E-06 | 0.000153 | 3.650084 |
| OLIG1 | 0.607816 | -0.11201 | 4.816261 | 6.30E-06 | 0.000156 | 3.625837 |
| RPPH1 | 0.705614 | 0.052819 | 4.813257 | 6.37E-06 | 0.000157 | 3.614671 |
| KAAG1 | -0.8496 | -0.19583 | -4.81316 | 6.38E-06 | 0.000157 | 3.614321 |
| EDNRA | -0.64194 | -0.13588 | -4.80729 | 6.52E-06 | 0.000159 | 3.592516 |
| NKPD1 | 0.521533 | 0.162673 | 4.804122 | 6.61E-06 | 0.000161 | 3.580736 |
| CDS2 | -0.54707 | -0.036 | -4.80361 | 6.62E-06 | 0.000161 | 3.578828 |
| LINC00560 | 0.505978 | -0.11581 | 4.800057 | 6.71E-06 | 0.000162 | 3.565645 |
| CTSZ | -1.36377 | -0.44573 | -4.79216 | 6.92E-06 | 0.000167 | 3.536344 |
| ELP3 | -0.52286 | -0.07197 | -4.7913 | 6.95E-06 | 0.000167 | 3.533166 |
| MRPL40 | -0.51529 | -0.12705 | -4.78956 | 6.99E-06 | 0.000167 | 3.52672 |
| RIMS3 | -0.6162 | -0.07467 | -4.78801 | 7.04E-06 | 0.000168 | 3.520979 |
| POLR2H | -0.70697 | -0.14211 | -4.78745 | 7.05E-06 | 0.000168 | 3.518907 |
| TEX2 | -0.58262 | -0.113 | -4.78097 | 7.23E-06 | 0.000172 | 3.494906 |
| OR5T1 | 0.541506 | -0.07826 | 4.774528 | 7.42E-06 | 0.000175 | 3.471042 |
| ACVR1 | -0.6575 | -0.14641 | -4.77122 | 7.52E-06 | 0.000176 | 3.458795 |
| NR4A1 | 0.532162 | 0.173724 | 4.764869 | 7.71E-06 | 0.000179 | 3.435325 |
| CDH8 | 0.510904 | -0.13145 | 4.761519 | 7.81E-06 | 0.000181 | 3.422946 |
| WDR55 | -0.56091 | -0.05809 | -4.75013 | 8.16E-06 | 0.000187 | 3.380905 |
| SCARB2 | -0.57324 | -0.21267 | -4.7463 | 8.29E-06 | 0.00019 | 3.366763 |
| FAM188A | -0.55107 | -0.16942 | -4.7428 | 8.40E-06 | 0.000191 | 3.353852 |
| IFT74 | -0.53897 | -0.08982 | -4.73091 | 8.80E-06 | 0.000198 | 3.310067 |
| FHL5 | -0.76721 | -0.14337 | -4.72594 | 8.97E-06 | 0.0002 | 3.291784 |
| SERBP1 | -0.56694 | -0.05141 | -4.72478 | 9.01E-06 | 0.000201 | 3.287514 |
| PFKM | -0.5922 | -0.07385 | -4.72248 | 9.09E-06 | 0.000202 | 3.279046 |
| CTPS1 | -0.54824 | -0.04653 | -4.72097 | 9.14E-06 | 0.000202 | 3.273508 |
| ID3 | -0.57689 | -0.22072 | -4.71984 | 9.19E-06 | 0.000203 | 3.269347 |
| OR5B3 | 0.502043 | -0.12984 | 4.719359 | 9.20E-06 | 0.000203 | 3.267585 |
| PRF1 | 0.536117 | 0.03035 | 4.717552 | 9.27E-06 | 0.000204 | 3.260942 |
| CABP5 | 0.507167 | -0.13372 | 4.71251 | 9.45E-06 | 0.000208 | 3.24242 |
| COQ8A | -0.56211 | -0.11194 | -4.71071 | 9.52E-06 | 0.000209 | 3.23581 |
| NDUFA7 | -0.52342 | -0.16712 | -4.70916 | 9.58E-06 | 0.00021 | 3.230102 |
| FCHO1 | 0.508618 | 0.082626 | 4.70714 | 9.65E-06 | 0.000211 | 3.222702 |
| ASAP2 | -0.63396 | -0.16381 | -4.70563 | 9.71E-06 | 0.000211 | 3.217146 |
| HRCT1 | -0.74557 | 0.061868 | -4.70306 | 9.80E-06 | 0.000213 | 3.207735 |
| ZNF442 | 0.5024 | -0.02047 | 4.701333 | 9.87E-06 | 0.000214 | 3.201398 |
| KIAA1211 | 0.551553 | -0.0603 | 4.698415 | 9.98E-06 | 0.000216 | 3.190699 |
| CLDN22 | 0.580906 | -0.09846 | 4.693408 | 1.02E-05 | 0.000219 | 3.172347 |
| PMP22 | -0.60504 | 0.435159 | -4.69034 | 1.03E-05 | 0.000221 | 3.161122 |
| SNRPB2 | -0.59579 | 0.010075 | -4.68796 | 1.04E-05 | 0.000222 | 3.152402 |
| VASH1 | 0.612877 | 0.07503 | 4.686131 | 1.05E-05 | 0.000223 | 3.145694 |
| COX6A1 | -0.5254 | -0.08997 | -4.67678 | 1.09E-05 | 0.000229 | 3.1115 |
| GRASP | 0.800732 | 0.091877 | 4.674037 | 1.10E-05 | 0.000231 | 3.101456 |
| KCNS1 | 0.539971 | -0.0556 | 4.661869 | 1.15E-05 | 0.000238 | 3.057011 |
| IGSF9 | 0.513321 | -0.067 | 4.653997 | 1.19E-05 | 0.000244 | 3.028295 |
| LOC392196 | 0.560255 | -0.12923 | 4.640268 | 1.25E-05 | 0.000254 | 2.978278 |
| ATP1A2 | -0.83866 | 0.024553 | -4.63415 | 1.28E-05 | 0.00026 | 2.956029 |
| PTGIR | -0.55522 | -0.17649 | -4.63034 | 1.30E-05 | 0.000263 | 2.942168 |
| PIK3R2 | -0.59951 | -0.19036 | -4.62471 | 1.33E-05 | 0.000268 | 2.921708 |
| TMEM252 | -0.66203 | 0.019801 | -4.62457 | 1.33E-05 | 0.000268 | 2.921189 |
| ITK | 0.808646 | 0.364483 | 4.620095 | 1.35E-05 | 0.000271 | 2.904943 |
| POLR2F | -0.53317 | -0.09627 | -4.61302 | 1.39E-05 | 0.000276 | 2.879271 |
| ALDH6A1 | -0.56036 | 0.048259 | -4.60847 | 1.41E-05 | 0.000279 | 2.862786 |
| FAM136A | -0.52296 | -0.09377 | -4.60663 | 1.42E-05 | 0.00028 | 2.856091 |
| LEPR | -0.73026 | 0.603051 | -4.59956 | 1.46E-05 | 0.000286 | 2.830486 |
| CCDC107 | -0.58642 | -0.01277 | -4.598 | 1.47E-05 | 0.000288 | 2.824847 |
| TOX2 | -0.75626 | -0.0961 | -4.59646 | 1.48E-05 | 0.000289 | 2.819262 |
| RRP36 | -0.55792 | -0.08857 | -4.59188 | 1.51E-05 | 0.000293 | 2.802705 |
| MED1 | -0.55045 | -0.12126 | -4.59085 | 1.51E-05 | 0.000293 | 2.798971 |
| HAUS7 | -0.52924 | -0.13242 | -4.59079 | 1.51E-05 | 0.000293 | 2.798735 |
| DYSF | 0.566394 | 0.13622 | 4.587499 | 1.53E-05 | 0.000296 | 2.786847 |
| IL22RA1 | 0.525395 | 0.024561 | 4.579674 | 1.58E-05 | 0.000302 | 2.758568 |
| ZC2HC1A | -0.52949 | 0.133712 | -4.57843 | 1.59E-05 | 0.000302 | 2.754081 |
| SERGEF | -0.5251 | -0.03213 | -4.57666 | 1.60E-05 | 0.000304 | 2.747702 |
| CAV2 | -0.58546 | 0.365058 | -4.57172 | 1.63E-05 | 0.000308 | 2.729867 |
| E2F3 | -0.60354 | -0.05858 | -4.56218 | 1.69E-05 | 0.000318 | 2.695467 |
| COX16 | -0.55699 | -0.14493 | -4.56012 | 1.70E-05 | 0.00032 | 2.688033 |
| PREP | -0.55966 | -0.06869 | -4.54184 | 1.82E-05 | 0.000338 | 2.622258 |
| RDH11 | -0.55448 | 0.150836 | -4.53374 | 1.88E-05 | 0.000345 | 2.593156 |
| ZNF532 | -0.64863 | -0.1553 | -4.52089 | 1.98E-05 | 0.000361 | 2.54707 |
| UBE4A | -0.61319 | -0.04448 | -4.51667 | 2.01E-05 | 0.000366 | 2.531937 |
| TMED2 | -0.68233 | -0.15891 | -4.51638 | 2.01E-05 | 0.000366 | 2.530919 |
| MDH1 | -0.72342 | -0.13365 | -4.51043 | 2.06E-05 | 0.000372 | 2.509598 |
| ACPT | 0.597534 | 0.268131 | 4.508765 | 2.07E-05 | 0.000374 | 2.503643 |
| SEMA4A | 0.680198 | 0.208578 | 4.504106 | 2.11E-05 | 0.000379 | 2.486978 |
| TC2N | -0.68132 | -0.07866 | -4.50391 | 2.11E-05 | 0.000379 | 2.486283 |
| ZC3H12A | 0.738606 | 0.275011 | 4.503587 | 2.11E-05 | 0.00038 | 2.485124 |
| LAIR1 | 0.612161 | 0.465958 | 4.502011 | 2.12E-05 | 0.000381 | 2.479489 |
| UFSP2 | -0.51359 | -0.1351 | -4.50116 | 2.13E-05 | 0.000381 | 2.476443 |
| SNAI3 | 0.703223 | -0.04961 | 4.499329 | 2.14E-05 | 0.000382 | 2.4699 |
| PA2G4 | -0.721 | -0.00366 | -4.49932 | 2.14E-05 | 0.000382 | 2.469858 |
| DENND4C | -0.60639 | -0.07831 | -4.49907 | 2.15E-05 | 0.000382 | 2.468985 |
| SNORD27 | 0.510168 | -0.14084 | 4.498866 | 2.15E-05 | 0.000382 | 2.468248 |
| FOXD2 | -0.56101 | -0.05899 | -4.4966 | 2.17E-05 | 0.000385 | 2.460154 |
| RBPMS2 | -0.81361 | 0.010783 | -4.49248 | 2.20E-05 | 0.00039 | 2.445419 |
| COX5B | -0.89158 | -0.4154 | -4.4922 | 2.20E-05 | 0.00039 | 2.444437 |
| SMIM19 | -0.5552 | -0.0867 | -4.49096 | 2.21E-05 | 0.000391 | 2.439997 |
| TMEM43 | -0.54917 | -0.1243 | -4.49092 | 2.21E-05 | 0.000391 | 2.439865 |
| ZC3H8 | 0.563012 | -0.06368 | 4.489941 | 2.22E-05 | 0.000391 | 2.436372 |
| OLFM1 | -0.67103 | 0.163 | -4.48862 | 2.23E-05 | 0.000392 | 2.431643 |
| PCSK9 | 0.502901 | -0.07381 | 4.485178 | 2.26E-05 | 0.000396 | 2.419377 |
| CITED2 | -0.7321 | -0.04851 | -4.48272 | 2.28E-05 | 0.000399 | 2.410594 |
| G0S2 | 0.796316 | 0.246593 | 4.478759 | 2.32E-05 | 0.000403 | 2.396491 |
| CXCL5 | 0.599382 | 0.216764 | 4.474123 | 2.36E-05 | 0.000407 | 2.379975 |
| UBB | -0.74457 | 0.201694 | -4.46336 | 2.46E-05 | 0.000419 | 2.341668 |
| PLCG1 | -0.53588 | 0.26485 | -4.46146 | 2.48E-05 | 0.000422 | 2.334905 |
| MYO1D | -0.84565 | -0.1949 | -4.458 | 2.51E-05 | 0.000426 | 2.322628 |
| BTG2 | 0.808854 | 0.223557 | 4.453658 | 2.55E-05 | 0.00043 | 2.307189 |
| BTNL8 | -0.62881 | 0.293792 | -4.44932 | 2.59E-05 | 0.000436 | 2.291785 |
| ZNF503 | -0.60914 | -0.09353 | -4.4479 | 2.61E-05 | 0.000437 | 2.286756 |
| SARNP | -0.59385 | -0.17052 | -4.43226 | 2.76E-05 | 0.000458 | 2.231295 |
| INTS3 | -0.52548 | -0.02745 | -4.43166 | 2.77E-05 | 0.000458 | 2.229194 |
| ZNF831 | 0.604475 | 0.283583 | 4.42994 | 2.79E-05 | 0.00046 | 2.22309 |
| FOXJ2 | -0.51517 | -0.12337 | -4.41623 | 2.94E-05 | 0.000477 | 2.174604 |
| LYPD8 | 0.580867 | -0.07714 | 4.413098 | 2.97E-05 | 0.000479 | 2.163537 |
| PHF20 | -0.50596 | -0.08447 | -4.41145 | 2.99E-05 | 0.00048 | 2.157707 |
| RPLP0 | -1.22844 | 0.883525 | -4.41052 | 3.00E-05 | 0.000481 | 2.154418 |
| SESN1 | -0.59001 | -0.13367 | -4.41016 | 3.00E-05 | 0.000481 | 2.15318 |
| MED20 | -0.54799 | -0.15892 | -4.40967 | 3.01E-05 | 0.000481 | 2.151437 |
| CANX | -0.94714 | 0.218762 | -4.40822 | 3.03E-05 | 0.000483 | 2.146325 |
| COX7B | -0.56042 | -0.1865 | -4.40798 | 3.03E-05 | 0.000483 | 2.145455 |
| TPPP3 | 0.627813 | 0.271475 | 4.402344 | 3.09E-05 | 0.000491 | 2.125588 |
| SPINT2 | -0.60956 | -0.12313 | -4.40184 | 3.10E-05 | 0.000491 | 2.123813 |
| LCE1E | 0.504723 | 0.247549 | 4.401001 | 3.11E-05 | 0.000492 | 2.120852 |
| RAX2 | 0.709899 | 0.006082 | 4.399953 | 3.12E-05 | 0.000494 | 2.117157 |
| CHSY3 | -0.63647 | -0.24909 | -4.39827 | 3.14E-05 | 0.000496 | 2.111208 |
| COX6C | -0.62943 | -0.13374 | -4.39797 | 3.14E-05 | 0.000496 | 2.110167 |
| MRPL33 | -0.57125 | 4.65E-05 | -4.39524 | 3.18E-05 | 0.0005 | 2.100536 |
| TMEM115 | -0.58312 | -0.08725 | -4.39286 | 3.20E-05 | 0.000502 | 2.092162 |
| P3H3 | -0.6481 | 0.038547 | -4.38978 | 3.24E-05 | 0.000507 | 2.081334 |
| ATP6V1D | -0.59952 | -0.14807 | -4.38494 | 3.30E-05 | 0.000514 | 2.0643 |
| UBR4 | 0.66135 | 0.104312 | 4.381671 | 3.34E-05 | 0.000519 | 2.052791 |
| PNPO | -0.70031 | -0.12539 | -4.3791 | 3.37E-05 | 0.000522 | 2.04376 |
| SNURF | -0.56925 | 0.22107 | -4.37908 | 3.37E-05 | 0.000522 | 2.04367 |
| CLDN23 | -0.5601 | -0.13617 | -4.37019 | 3.49E-05 | 0.000537 | 2.012462 |
| AHCYL1 | -0.70744 | -0.05777 | -4.36712 | 3.53E-05 | 0.000541 | 2.001692 |
| MRPL13 | -0.50136 | -0.3202 | -4.35999 | 3.62E-05 | 0.000554 | 1.976689 |
| YIF1A | -0.51542 | -0.13631 | -4.35856 | 3.64E-05 | 0.000555 | 1.971681 |
| GPD1L | -0.69371 | -0.19049 | -4.35835 | 3.65E-05 | 0.000555 | 1.970923 |
| GSDMB | 0.672356 | 0.560551 | 4.357037 | 3.66E-05 | 0.000557 | 1.966328 |
| GALNT15 | -0.64476 | 0.033742 | -4.35366 | 3.71E-05 | 0.000561 | 1.954512 |
| UTP6 | -0.60389 | -0.20309 | -4.35325 | 3.72E-05 | 0.000561 | 1.953079 |
| KIAA1191 | -0.55254 | 0.109211 | -4.35214 | 3.73E-05 | 0.000562 | 1.949187 |
| IFIT3 | -0.52856 | 0.331342 | -4.35192 | 3.73E-05 | 0.000562 | 1.948394 |
| 10-Mar | -0.53186 | 0.027563 | -4.35064 | 3.75E-05 | 0.000563 | 1.943921 |
| SLC2A1 | -0.71732 | -0.06812 | -4.34833 | 3.79E-05 | 0.000567 | 1.935822 |
| S100A6 | -0.50466 | -0.08819 | -4.34293 | 3.86E-05 | 0.000574 | 1.916951 |
| ASNS | -0.61552 | 0.336559 | -4.33713 | 3.95E-05 | 0.000583 | 1.896687 |
| SEC22B | -0.52478 | -0.09757 | -4.33225 | 4.02E-05 | 0.000591 | 1.879646 |
| TMEM243 | -0.51464 | -0.07991 | -4.32735 | 4.09E-05 | 0.0006 | 1.862515 |
| CEACAM6 | 0.532318 | -0.05849 | 4.325231 | 4.12E-05 | 0.000601 | 1.85514 |
| SV2B | 0.518666 | -0.01401 | 4.316282 | 4.26E-05 | 0.000616 | 1.823947 |
| FGFR1 | 0.542908 | 0.239358 | 4.312902 | 4.32E-05 | 0.000623 | 1.81218 |
| WFS1 | -0.66352 | -0.2272 | -4.31247 | 4.32E-05 | 0.000623 | 1.810674 |
| FAM84A | 0.545255 | 0.013237 | 4.30821 | 4.39E-05 | 0.000631 | 1.795851 |
| SPEG | -0.58869 | -0.0435 | -4.30427 | 4.46E-05 | 0.000639 | 1.78216 |
| EVA1C | -0.56822 | 0.048278 | -4.30389 | 4.46E-05 | 0.000639 | 1.780823 |
| NCOA5 | -0.54659 | 0.101411 | -4.30132 | 4.51E-05 | 0.000644 | 1.771878 |
| LIN28A | 0.563986 | -0.10451 | 4.293598 | 4.64E-05 | 0.000657 | 1.745069 |
| TMED6 | 0.580706 | 0.050177 | 4.288153 | 4.73E-05 | 0.000667 | 1.726173 |
| SMU1 | -0.62571 | -0.0453 | -4.28772 | 4.74E-05 | 0.000667 | 1.72468 |
| GNS | -0.65683 | -0.12066 | -4.28677 | 4.76E-05 | 0.000669 | 1.721366 |
| EFNB2 | -0.68642 | -0.03355 | -4.27932 | 4.89E-05 | 0.000681 | 1.69554 |
| PPP1R2 | -0.51905 | -0.05302 | -4.27773 | 4.92E-05 | 0.000683 | 1.690036 |
| EPHB4 | -0.59853 | -0.05602 | -4.26497 | 5.16E-05 | 0.000704 | 1.645911 |
| JTB | -0.52258 | -0.14417 | -4.25638 | 5.32E-05 | 0.000723 | 1.616221 |
| BLVRA | -0.61264 | -0.22052 | -4.25377 | 5.37E-05 | 0.000728 | 1.607201 |
| PCYOX1 | -0.65341 | 0.135048 | -4.2532 | 5.38E-05 | 0.000729 | 1.605245 |
| KLHL9 | -0.79387 | 0.01087 | -4.24872 | 5.47E-05 | 0.000738 | 1.589788 |
| RASL11A | -0.54678 | -0.19282 | -4.24381 | 5.57E-05 | 0.000749 | 1.572875 |
| CERS6 | -0.57117 | -0.03004 | -4.24139 | 5.62E-05 | 0.000755 | 1.56451 |
| DNAJB6 | -0.50044 | 0.190172 | -4.23521 | 5.75E-05 | 0.000769 | 1.543262 |
| FBXO11 | -0.51275 | 0.167661 | -4.23366 | 5.78E-05 | 0.000771 | 1.537921 |
| BORCS8-MEF2B | 0.502137 | 0.004874 | 4.230454 | 5.85E-05 | 0.000779 | 1.526887 |
| TM9SF3 | -0.5113 | -0.09316 | -4.23015 | 5.86E-05 | 0.000779 | 1.525826 |
| NDUFA8 | -0.64139 | -0.15626 | -4.22792 | 5.91E-05 | 0.000783 | 1.518184 |
| CSPG4 | -0.73244 | -0.19441 | -4.22781 | 5.91E-05 | 0.000783 | 1.517785 |
| RB1CC1 | -0.52707 | -0.0399 | -4.21794 | 6.13E-05 | 0.000806 | 1.483888 |
| PREPL | -0.50378 | 0.191214 | -4.21581 | 6.18E-05 | 0.000811 | 1.476567 |
| PTEN | -0.52292 | -0.03475 | -4.20164 | 6.50E-05 | 0.000843 | 1.428009 |
| BTF3L4 | -0.59017 | 0.165681 | -4.19625 | 6.63E-05 | 0.000857 | 1.409578 |
| PSMG2 | -0.56758 | -0.23485 | -4.19056 | 6.77E-05 | 0.000869 | 1.390122 |
| NARS | -0.59018 | -0.19466 | -4.18992 | 6.79E-05 | 0.000869 | 1.387927 |
| CDC42EP4 | -0.533 | -0.04995 | -4.18612 | 6.88E-05 | 0.000878 | 1.374946 |
| GOLPH3 | -0.61454 | -0.12587 | -4.17945 | 7.05E-05 | 0.00089 | 1.352167 |
| PRMT1 | -0.54108 | -0.00026 | -4.17831 | 7.08E-05 | 0.000891 | 1.348283 |
| DHRS7 | -0.5595 | -0.21753 | -4.17778 | 7.10E-05 | 0.000891 | 1.346492 |
| VEZF1 | -0.61923 | -0.15236 | -4.17327 | 7.21E-05 | 0.000903 | 1.331081 |
| PLCB3 | 0.536952 | -0.07871 | 4.171609 | 7.26E-05 | 0.000907 | 1.325434 |
| IRF2BPL | -0.75054 | -0.06541 | -4.17027 | 7.29E-05 | 0.000911 | 1.320861 |
| RPA2 | -0.55294 | -0.10862 | -4.16529 | 7.43E-05 | 0.000924 | 1.303907 |
| NDUFAF2 | -0.50227 | -0.07753 | -4.16145 | 7.53E-05 | 0.000937 | 1.290838 |
| MAP1S | 0.546793 | -0.03261 | 4.158441 | 7.61E-05 | 0.000945 | 1.280606 |
| PEG3 | -0.50463 | 0.002682 | -4.15332 | 7.76E-05 | 0.000959 | 1.263206 |
| PXDN | -0.80319 | -0.14382 | -4.14821 | 7.90E-05 | 0.000972 | 1.245849 |
| SPIRE1 | -0.51719 | -0.17003 | -4.14781 | 7.91E-05 | 0.000972 | 1.244472 |
| LRRC10 | 0.706466 | -0.19484 | 4.145373 | 7.98E-05 | 0.00098 | 1.236208 |
| FAM46B | -0.63739 | -0.15552 | -4.14339 | 8.04E-05 | 0.000985 | 1.229495 |
| TAF13 | 0.592059 | 0.23425 | 4.142187 | 8.08E-05 | 0.000987 | 1.225399 |
| DEFB127 | 0.543473 | 0.00104 | 4.140386 | 8.13E-05 | 0.000992 | 1.219292 |
| CXCL1 | 0.514405 | 0.233926 | 4.139077 | 8.17E-05 | 0.000994 | 1.214852 |
| TIMP4 | -0.83318 | 0.105567 | -4.12542 | 8.58E-05 | 0.001037 | 1.168591 |
| ANKRD34C | 0.583451 | -0.17767 | 4.120925 | 8.72E-05 | 0.001047 | 1.153396 |
| CRBN | -0.52873 | 0.122018 | -4.11951 | 8.77E-05 | 0.001051 | 1.148597 |
| PIK3CD | 0.506204 | 0.034984 | 4.116737 | 8.86E-05 | 0.001058 | 1.139245 |
| LINC00901 | 0.6131 | -0.09624 | 4.115231 | 8.90E-05 | 0.001061 | 1.134158 |
| TSPYL1 | -0.52686 | -0.05281 | -4.11395 | 8.95E-05 | 0.001063 | 1.129826 |
| RNASEH1 | -0.5643 | -0.15996 | -4.10953 | 9.09E-05 | 0.001077 | 1.114913 |
| ATP13A1 | 0.593923 | 0.234366 | 4.105861 | 9.21E-05 | 0.00109 | 1.102531 |
| SPPL2A | -0.55994 | -0.13699 | -4.103 | 9.31E-05 | 0.001096 | 1.092879 |
| MAP3K6 | -0.63711 | -0.01614 | -4.09814 | 9.47E-05 | 0.00111 | 1.076514 |
| RNF150 | -0.57325 | -0.06878 | -4.09786 | 9.48E-05 | 0.00111 | 1.07557 |
| KLHDC2 | -0.68121 | -0.17948 | -4.09676 | 9.52E-05 | 0.001113 | 1.071859 |
| ZNF573 | -0.64066 | -0.02031 | -4.09431 | 9.60E-05 | 0.001121 | 1.063595 |
| DBI | -0.52229 | 0.031359 | -4.08672 | 9.87E-05 | 0.001139 | 1.038091 |
| DERL1 | -0.64362 | -0.1165 | -4.0843 | 9.95E-05 | 0.001148 | 1.029951 |
| LAMA5 | -0.54258 | -0.09804 | -4.08267 | 0.0001 | 0.001154 | 1.024471 |
| TRIP11 | -0.51956 | 0.003201 | -4.07928 | 0.000101 | 0.001165 | 1.013088 |
| SPATA31A3 | 0.545032 | -0.13216 | 4.07912 | 0.000101 | 0.001165 | 1.012541 |
| NDUFA12 | -0.65581 | -0.27453 | -4.06685 | 0.000106 | 0.001203 | 0.971389 |
| RBP1 | -0.52213 | -0.14459 | -4.06633 | 0.000106 | 0.001204 | 0.969642 |
| CCNI | -0.51912 | -0.16935 | -4.05632 | 0.00011 | 0.001239 | 0.936124 |
| 5-Sep | 0.501803 | 0.449129 | 4.052346 | 0.000112 | 0.00125 | 0.92283 |
| SMIM7 | -0.54285 | -0.06652 | -4.04324 | 0.000115 | 0.001284 | 0.892404 |
| UBE4B | -0.53199 | -0.1391 | -4.03574 | 0.000118 | 0.001309 | 0.867377 |
| WDR41 | -0.5333 | -0.07409 | -4.03153 | 0.00012 | 0.001324 | 0.853355 |
| SIK1 | 1.107399 | 0.478715 | 4.031174 | 0.00012 | 0.001324 | 0.852169 |
| PEBP1 | -0.65163 | -0.20303 | -4.02849 | 0.000122 | 0.001336 | 0.843234 |
| UQCRFS1 | -0.50358 | -0.09841 | -4.02101 | 0.000125 | 0.001362 | 0.818344 |
| CCR7 | 0.867436 | 0.420206 | 4.019021 | 0.000126 | 0.001369 | 0.81172 |
| CAMLG | -0.5503 | -0.24642 | -4.0177 | 0.000126 | 0.001374 | 0.807321 |
| ZNHIT3 | -0.53222 | 0.089027 | -4.01473 | 0.000128 | 0.001383 | 0.797469 |
| CAMK2G | -0.50508 | -0.08138 | -4.01431 | 0.000128 | 0.001383 | 0.796057 |
| GNL2 | -0.54748 | -0.19085 | -4.01244 | 0.000129 | 0.001389 | 0.789869 |
| CST6 | -0.61392 | -0.09827 | -4.00902 | 0.00013 | 0.001402 | 0.778505 |
| GMDS | -0.8111 | -0.27963 | -4.00874 | 0.00013 | 0.001403 | 0.777553 |
| AZIN2 | 0.622307 | 0.064849 | 4.008027 | 0.000131 | 0.001405 | 0.7752 |
| EIF2S3 | -0.52014 | -0.02554 | -4.00004 | 0.000135 | 0.001438 | 0.74871 |
| IARS2 | -0.55138 | -0.19314 | -3.99817 | 0.000135 | 0.001444 | 0.742515 |
| BRD3 | -0.51237 | -0.07397 | -3.99566 | 0.000137 | 0.001455 | 0.734215 |
| TTTY3 | 0.555362 | 0.053211 | 3.994433 | 0.000137 | 0.001459 | 0.730138 |
| MAP2K2 | -0.55786 | -0.06276 | -3.9941 | 0.000137 | 0.00146 | 0.72903 |
| DLST | -0.61724 | -0.27179 | -3.98887 | 0.00014 | 0.001483 | 0.711738 |
| ZNF575 | -0.60001 | -0.04449 | -3.9874 | 0.000141 | 0.001489 | 0.706864 |
| VPS29 | -0.57109 | -0.26388 | -3.98313 | 0.000143 | 0.001506 | 0.692752 |
| BOD1 | -0.55191 | -0.11695 | -3.97497 | 0.000147 | 0.001539 | 0.665809 |
| SUMF1 | -0.55528 | -0.12223 | -3.95746 | 0.000156 | 0.001615 | 0.608087 |
| HCK | 0.715676 | 0.057404 | 3.954481 | 0.000158 | 0.001627 | 0.598301 |
| ATOH8 | -0.58231 | -0.01124 | -3.94314 | 0.000165 | 0.001676 | 0.561048 |
| FCN1 | 0.812169 | 0.080154 | 3.941331 | 0.000166 | 0.001683 | 0.555105 |
| SNAI2 | -0.5111 | 0.165595 | -3.93944 | 0.000167 | 0.001689 | 0.548893 |
| ACO2 | 0.575808 | 0.095754 | 3.934265 | 0.00017 | 0.001714 | 0.531935 |
| DEFA1B | 0.611589 | 0.286176 | 3.931921 | 0.000171 | 0.001723 | 0.524255 |
| TCEA3 | -0.5618 | -0.07546 | -3.92397 | 0.000176 | 0.001755 | 0.498238 |
| COX7C | -0.71454 | -0.3548 | -3.91733 | 0.00018 | 0.001785 | 0.476521 |
| SAMD4A | 0.527306 | -0.09204 | 3.915052 | 0.000182 | 0.001794 | 0.469075 |
| ZBED5 | -0.54059 | 0.03541 | -3.91381 | 0.000182 | 0.001798 | 0.465014 |
| IL1B | 0.826498 | 0.394036 | 3.909164 | 0.000185 | 0.001817 | 0.449854 |
| APLNR | 0.579075 | 0.146137 | 3.908389 | 0.000186 | 0.001821 | 0.447327 |
| MRAP2 | -0.77689 | -0.16163 | -3.9015 | 0.00019 | 0.001857 | 0.424867 |
| LYRM2 | -0.52941 | -0.09849 | -3.90052 | 0.000191 | 0.001861 | 0.42168 |
| EFR3A | -0.51512 | -0.14005 | -3.89943 | 0.000192 | 0.001866 | 0.418111 |
| C5orf46 | -0.96996 | 0.036574 | -3.89106 | 0.000198 | 0.001904 | 0.390873 |
| TGFBR3 | -0.68934 | -0.07183 | -3.89095 | 0.000198 | 0.001904 | 0.39051 |
| HERC2 | -0.55904 | -0.02339 | -3.89074 | 0.000198 | 0.001904 | 0.389839 |
| RHOF | 0.502014 | -0.06028 | 3.884878 | 0.000202 | 0.001931 | 0.370783 |
| THY1 | 0.572989 | 0.147586 | 3.880827 | 0.000205 | 0.00195 | 0.357626 |
| PDE8B | -0.51136 | 0.101028 | -3.87643 | 0.000208 | 0.001972 | 0.34336 |
| PHGDH | -0.59188 | -0.08861 | -3.87228 | 0.000211 | 0.001995 | 0.329912 |
| ZNF25 | -0.56927 | -0.13413 | -3.86778 | 0.000214 | 0.002014 | 0.315328 |
| COQ9 | -0.50614 | -0.01074 | -3.86049 | 0.00022 | 0.002054 | 0.29172 |
| SNORD14A | 0.529405 | 0.071133 | 3.857028 | 0.000222 | 0.002069 | 0.28053 |
| CAP2 | -0.79153 | -0.2154 | -3.85505 | 0.000224 | 0.002078 | 0.274135 |
| AKT1S1 | -0.6252 | 0.116503 | -3.85238 | 0.000226 | 0.002094 | 0.26551 |
| COA5 | -0.5033 | -0.08519 | -3.85047 | 0.000228 | 0.002104 | 0.259349 |
| RPL13AP6 | -0.50765 | -0.17575 | -3.84732 | 0.00023 | 0.002121 | 0.249165 |
| RNA28S5 | -1.58302 | 0.452937 | -3.8404 | 0.000236 | 0.002159 | 0.226855 |
| METTL7A | -0.65817 | -0.26997 | -3.83582 | 0.000239 | 0.002184 | 0.212109 |
| SPRN | 0.525211 | 0.227778 | 3.833186 | 0.000242 | 0.002196 | 0.203619 |
| CST7 | 0.549173 | 0.060982 | 3.828514 | 0.000246 | 0.002224 | 0.18859 |
| PLIN3 | -0.52473 | -0.24496 | -3.8245 | 0.000249 | 0.00225 | 0.175672 |
| DCD | 0.543614 | -0.24594 | 3.818954 | 0.000254 | 0.002281 | 0.15787 |
| NUP88 | -0.64589 | -0.24885 | -3.81108 | 0.000261 | 0.002326 | 0.132627 |
| CSDC2 | -0.52916 | -0.16857 | -3.81101 | 0.000261 | 0.002326 | 0.132394 |
| NDN | -0.61653 | -0.18947 | -3.80412 | 0.000267 | 0.002362 | 0.110323 |
| LSM5 | -0.57859 | 0.134216 | -3.80337 | 0.000268 | 0.002367 | 0.107906 |
| XPA | -0.6515 | -0.14415 | -3.80266 | 0.000269 | 0.002369 | 0.105652 |
| NOTCH3 | -0.5285 | -0.19417 | -3.79917 | 0.000272 | 0.002386 | 0.094481 |
| SPARC | -0.75413 | -0.0431 | -3.79742 | 0.000273 | 0.002398 | 0.088877 |
| BARD1 | -0.53673 | -0.06709 | -3.78824 | 0.000282 | 0.002459 | 0.059554 |
| AKR1C3 | -0.63954 | -0.21011 | -3.78179 | 0.000289 | 0.002491 | 0.038971 |
| SCOC | -0.60955 | -0.15158 | -3.78138 | 0.000289 | 0.002493 | 0.037657 |
| APLF | 0.507265 | -0.07618 | 3.771692 | 0.000299 | 0.002557 | 0.006801 |
| HBD | 0.534828 | 0.112912 | 3.771626 | 0.000299 | 0.002557 | 0.006593 |
| CLLU1OS | 0.646478 | -0.00786 | 3.764045 | 0.000307 | 0.002604 | -0.01752 |
| HELZ2 | 0.532541 | 0.089251 | 3.763672 | 0.000307 | 0.002604 | -0.0187 |
| SPIB | 0.540642 | 0.149789 | 3.763041 | 0.000308 | 0.002607 | -0.02071 |
| C15orf52 | -0.58697 | -0.12213 | -3.7629 | 0.000308 | 0.002608 | -0.02116 |
| PTGS2 | 0.759874 | 0.347523 | 3.758758 | 0.000312 | 0.002633 | -0.03431 |
| ANP32AP1 | -0.85236 | -0.0079 | -3.75469 | 0.000317 | 0.002659 | -0.04721 |
| TACC1 | -0.51661 | -0.20345 | -3.75003 | 0.000322 | 0.002689 | -0.06201 |
| ACTN1 | -0.53844 | -0.28232 | -3.747 | 0.000325 | 0.002711 | -0.07158 |
| LIMCH1 | -0.79331 | -0.30463 | -3.7403 | 0.000333 | 0.002754 | -0.09281 |
| ETFA | -0.51228 | -0.12908 | -3.73893 | 0.000334 | 0.002762 | -0.09713 |
| ADAMTS8 | -0.59107 | 0.002418 | -3.73575 | 0.000338 | 0.002784 | -0.10719 |
| GAS1 | -0.68499 | 0.320123 | -3.73455 | 0.000339 | 0.002793 | -0.11098 |
| ADAM8 | 0.774386 | 0.229181 | 3.732798 | 0.000341 | 0.002807 | -0.11652 |
| HBEGF | 0.75986 | 0.188165 | 3.719611 | 0.000357 | 0.002904 | -0.15813 |
| ESYT2 | -0.68394 | -0.32499 | -3.71366 | 0.000364 | 0.002944 | -0.17689 |
| GNG12 | -0.53584 | -0.07846 | -3.71359 | 0.000364 | 0.002944 | -0.17708 |
| SNCG | -0.50996 | -0.07944 | -3.6998 | 0.000382 | 0.00305 | -0.22044 |
| MX1 | 0.696318 | -0.02914 | 3.699337 | 0.000382 | 0.003054 | -0.22189 |
| PHPT1 | -0.52443 | -0.14103 | -3.69495 | 0.000388 | 0.003085 | -0.23566 |
| KIAA1468 | 0.525916 | 0.083153 | 3.694263 | 0.000389 | 0.00309 | -0.23781 |
| CD300A | 0.649936 | -0.05415 | 3.69005 | 0.000395 | 0.003115 | -0.25102 |
| SPRYD3 | -0.51028 | -0.14361 | -3.67794 | 0.000411 | 0.003214 | -0.28892 |
| SLIRP | -0.67695 | -0.26827 | -3.67469 | 0.000416 | 0.00323 | -0.29909 |
| ZNF223 | 0.679312 | -0.01638 | 3.672826 | 0.000418 | 0.003247 | -0.3049 |
| HAUS2 | 0.607828 | -0.04208 | 3.669029 | 0.000424 | 0.003279 | -0.31675 |
| SORT1 | -0.55085 | -0.18617 | -3.66715 | 0.000426 | 0.003289 | -0.32262 |
| RAB10 | -0.52116 | -0.16023 | -3.65395 | 0.000446 | 0.003402 | -0.36373 |
| MT1X | -0.91996 | -0.38503 | -3.65303 | 0.000447 | 0.003405 | -0.3666 |
| CORO1C | -0.52537 | -0.12365 | -3.64843 | 0.000454 | 0.003451 | -0.38089 |
| ZBTB40 | -0.53331 | -0.11635 | -3.648 | 0.000455 | 0.003455 | -0.38223 |
| C2orf40 | -0.7611 | -0.06189 | -3.6442 | 0.00046 | 0.003487 | -0.39402 |
| MGST3 | -0.56987 | -0.21764 | -3.64369 | 0.000461 | 0.003488 | -0.39563 |
| NDUFB10 | -0.61143 | -0.16931 | -3.63779 | 0.000471 | 0.003541 | -0.41393 |
| ALPP | -0.51247 | -0.06638 | -3.63502 | 0.000475 | 0.003566 | -0.4225 |
| SGCA | -0.69593 | -0.18472 | -3.63223 | 0.000479 | 0.003593 | -0.43116 |
| CX3CR1 | 0.626333 | 0.504275 | 3.62735 | 0.000487 | 0.003637 | -0.44627 |
| BAG3 | -0.56451 | -0.08124 | -3.62713 | 0.000488 | 0.003637 | -0.44695 |
| RN7SL1 | 0.590986 | -0.06106 | 3.625931 | 0.00049 | 0.003648 | -0.45066 |
| CALU | -0.5283 | -0.18853 | -3.62253 | 0.000495 | 0.003683 | -0.46118 |
| NDUFA4 | -0.50166 | -0.19384 | -3.60942 | 0.000517 | 0.003794 | -0.50167 |
| PCNP | -0.53473 | -0.2072 | -3.60826 | 0.000519 | 0.003806 | -0.50525 |
| LPIN1 | -0.61556 | -0.18923 | -3.59842 | 0.000537 | 0.003907 | -0.53555 |
| TNNI3 | 0.50257 | -0.09956 | 3.595735 | 0.000542 | 0.003932 | -0.5438 |
| TM4SF5 | 0.606701 | 0.029509 | 3.580892 | 0.000569 | 0.004092 | -0.58938 |
| GSTCD | 0.614199 | 0.556649 | 3.579738 | 0.000571 | 0.004103 | -0.59291 |
| MANF | -0.54316 | -0.15057 | -3.57971 | 0.000571 | 0.004103 | -0.59299 |
| COX17 | -0.72454 | -0.42999 | -3.57544 | 0.000579 | 0.004151 | -0.60608 |
| FMOD | -0.56851 | -0.04729 | -3.57282 | 0.000584 | 0.004179 | -0.61411 |
| COX7A1 | -0.60493 | -0.18441 | -3.57223 | 0.000586 | 0.004186 | -0.61592 |
| P2RX6 | -0.50562 | 0.05746 | -3.56958 | 0.000591 | 0.004211 | -0.62403 |
| KIAA0355 | -0.51926 | -0.0941 | -3.56673 | 0.000596 | 0.004245 | -0.63274 |
| UCP2 | 0.743704 | 0.257083 | 3.562679 | 0.000604 | 0.004289 | -0.64511 |
| GLRX | -0.54313 | -0.10669 | -3.56127 | 0.000607 | 0.004303 | -0.64941 |
| GOLGA8A | 0.574938 | 0.110326 | 3.5603 | 0.000609 | 0.004312 | -0.65238 |
| KLHL42 | -0.53607 | -0.13118 | -3.55996 | 0.00061 | 0.004315 | -0.65342 |
| WDR74 | -0.54616 | -0.03077 | -3.5408 | 0.00065 | 0.004521 | -0.7118 |
| TSHZ2 | 0.672552 | 0.070511 | 3.536567 | 0.000659 | 0.004571 | -0.72465 |
| USF1 | -0.54956 | 0.040203 | -3.53317 | 0.000666 | 0.004612 | -0.73497 |
| MRPL20 | -0.67649 | 0.218362 | -3.53267 | 0.000667 | 0.004618 | -0.73649 |
| SSTR2 | -0.50723 | -0.15808 | -3.52995 | 0.000673 | 0.004651 | -0.74475 |
| RAB7B | 0.524571 | 0.02273 | 3.526734 | 0.000681 | 0.004691 | -0.75449 |
| SLC11A2 | -0.5149 | -0.10243 | -3.52659 | 0.000681 | 0.004691 | -0.75493 |
| ST13 | -0.50708 | -0.22796 | -3.52429 | 0.000686 | 0.004715 | -0.7619 |
| WAS | 0.529269 | 0.01031 | 3.51887 | 0.000698 | 0.004775 | -0.77832 |
| PARP4 | -0.52549 | -0.1166 | -3.51074 | 0.000717 | 0.004876 | -0.8029 |
| CD151 | -0.62184 | 0.395654 | -3.50925 | 0.000721 | 0.004895 | -0.8074 |
| RABGAP1 | -0.60688 | -0.20517 | -3.49527 | 0.000755 | 0.005067 | -0.84956 |
| SNX3 | -0.51346 | -0.17675 | -3.49373 | 0.000758 | 0.005083 | -0.85421 |
| OSM | 0.596556 | 0.279346 | 3.492558 | 0.000761 | 0.005089 | -0.85774 |
| SLC2A3 | 0.742341 | 0.34675 | 3.477664 | 0.000799 | 0.005278 | -0.9025 |
| GNG5 | 0.586434 | -0.01636 | 3.474912 | 0.000806 | 0.005312 | -0.91075 |
| NSMAF | -0.56726 | -0.00781 | -3.47456 | 0.000807 | 0.005315 | -0.91179 |
| CRYZ | -0.51552 | -0.11329 | -3.47139 | 0.000816 | 0.005353 | -0.92131 |
| SSR1 | -0.54364 | -0.15658 | -3.47096 | 0.000817 | 0.005359 | -0.9226 |
| HES3 | 0.679014 | -0.19462 | 3.469881 | 0.00082 | 0.005372 | -0.92583 |
| SOCS3 | 0.657104 | 0.541004 | 3.469488 | 0.000821 | 0.005377 | -0.927 |
| OTOF | 0.517783 | 0.343758 | 3.466835 | 0.000828 | 0.005417 | -0.93495 |
| CD3D | 0.516959 | 0.639281 | 3.46475 | 0.000834 | 0.005436 | -0.94119 |
| NUPR1 | -0.60679 | 0.055358 | -3.46195 | 0.000841 | 0.005471 | -0.94956 |
| GAL3ST4 | 0.577619 | -0.07359 | 3.460993 | 0.000844 | 0.005483 | -0.95243 |
| MEF2C | -0.63876 | -0.15934 | -3.45928 | 0.000849 | 0.005506 | -0.95753 |
| SERF2 | -0.51675 | -0.18324 | -3.44833 | 0.000879 | 0.005658 | -0.99023 |
| SLC25A44 | -0.6115 | 0.059733 | -3.43755 | 0.000911 | 0.0058 | -1.02233 |
| NDUFB5 | -0.51729 | -0.25496 | -3.43489 | 0.000918 | 0.005839 | -1.03023 |
| TUBB1 | 0.516369 | 0.098881 | 3.430554 | 0.000931 | 0.005899 | -1.04313 |
| OXR1 | -0.57149 | -0.23333 | -3.42951 | 0.000935 | 0.005913 | -1.04624 |
| MTHFD2 | -0.54828 | -0.10565 | -3.4287 | 0.000937 | 0.005921 | -1.04865 |
| GLDN | -0.67024 | -0.07731 | -3.41049 | 0.000994 | 0.006186 | -1.10257 |
| IGIP | -0.52886 | -0.15909 | -3.40851 | 0.001 | 0.006212 | -1.10845 |
| ACSL1 | -0.64924 | -0.01821 | -3.40661 | 0.001006 | 0.006233 | -1.11407 |
| ZYG11B | -0.51233 | -0.23821 | -3.40563 | 0.001009 | 0.006245 | -1.11695 |
| SRP14 | -0.52499 | -0.01609 | -3.39517 | 0.001044 | 0.006387 | -1.14782 |
| NDUFA3 | -0.55641 | -0.28203 | -3.39469 | 0.001046 | 0.006393 | -1.14922 |
| TREM2 | 0.649441 | 0.022418 | 3.38755 | 0.00107 | 0.006499 | -1.17024 |
| SERPINA3 | -0.69348 | -0.35297 | -3.3858 | 0.001076 | 0.006529 | -1.17538 |
| MAGOHB | -0.55199 | 0.06421 | -3.37262 | 0.001122 | 0.006725 | -1.21409 |
| SLC17A9 | 0.535792 | 0.081467 | 3.371638 | 0.001126 | 0.006742 | -1.21697 |
| KLHL30 | -0.53016 | -0.06441 | -3.35839 | 0.001174 | 0.006969 | -1.25576 |
| IL13RA1 | -0.54474 | -0.26876 | -3.34888 | 0.001211 | 0.007132 | -1.28352 |
| JAML | 0.533505 | -0.10501 | 3.348103 | 0.001214 | 0.007139 | -1.28578 |
| HOXA5 | -0.60424 | -0.18808 | -3.3439 | 0.00123 | 0.00721 | -1.29802 |
| RAMP1 | -0.53848 | -0.03815 | -3.34211 | 0.001237 | 0.007242 | -1.30323 |
| GFPT2 | 0.593554 | 0.352304 | 3.339099 | 0.001249 | 0.007296 | -1.31201 |
| TBCA | -0.5118 | -0.32903 | -3.32512 | 0.001306 | 0.00754 | -1.35261 |
| PPP1R3C | -0.83932 | -0.26338 | -3.32448 | 0.001308 | 0.007547 | -1.35448 |
| ZNF331 | 0.50176 | 0.543031 | 3.315993 | 0.001344 | 0.007701 | -1.37906 |
| BRCC3 | -0.5532 | -0.10332 | -3.30309 | 0.0014 | 0.007952 | -1.41635 |
| S100A12 | 0.532481 | 0.079728 | 3.301069 | 0.001409 | 0.007991 | -1.42218 |
| HBG2 | 0.924277 | 0.572848 | 3.293467 | 0.001443 | 0.008136 | -1.44409 |
| APP | -0.50238 | 0.363637 | -3.29235 | 0.001448 | 0.008157 | -1.44731 |
| CDH13 | -0.54094 | -0.18031 | -3.28803 | 0.001468 | 0.008252 | -1.45974 |
| FICD | -0.54571 | -0.12059 | -3.26717 | 0.001567 | 0.008671 | -1.51957 |
| RGS16 | 0.564427 | 0.251421 | 3.266604 | 0.00157 | 0.008677 | -1.5212 |
| CACNG8 | 0.613337 | 0.364468 | 3.254865 | 0.001629 | 0.008939 | -1.55474 |
| RPS19BP1 | 0.558833 | -0.23063 | 3.242174 | 0.001695 | 0.00922 | -1.5909 |
| MYOM1 | -0.56655 | -0.15133 | -3.23247 | 0.001747 | 0.009456 | -1.61848 |
| RPL24 | -0.85732 | -0.65752 | -3.22918 | 0.001765 | 0.009521 | -1.6278 |
| CPED1 | -0.65074 | -0.50056 | -3.22158 | 0.001807 | 0.00971 | -1.64933 |
| TMEM14B | -0.54113 | -0.09324 | -3.21198 | 0.001861 | 0.009933 | -1.67648 |
| HLA-G | 0.571876 | 0.030285 | 3.192238 | 0.001979 | 0.010389 | -1.7321 |
| SLMAP | -0.67949 | -0.34201 | -3.19093 | 0.001987 | 0.010425 | -1.73578 |
| ITGAL | 0.517351 | 0.062357 | 3.18257 | 0.002039 | 0.010624 | -1.75924 |
| CLEC14A | -0.50787 | -0.08471 | -3.17646 | 0.002077 | 0.010778 | -1.77636 |
| TMED10P1 | -0.52003 | -0.17844 | -3.16003 | 0.002185 | 0.011226 | -1.82227 |
| RPS21 | -0.55098 | -0.31003 | -3.15818 | 0.002197 | 0.011274 | -1.82741 |
| SLC26A3 | 0.50443 | -0.10242 | 3.14883 | 0.002261 | 0.01152 | -1.85345 |
| TMEM30B | -0.50496 | 0.028098 | -3.14717 | 0.002273 | 0.01156 | -1.85807 |
| VPS35 | -0.51292 | -0.15391 | -3.13858 | 0.002333 | 0.011804 | -1.88192 |
| MAOA | -0.67331 | -0.17524 | -3.13594 | 0.002352 | 0.011877 | -1.88924 |
| RAB11A | -0.54882 | -0.16436 | -3.13438 | 0.002363 | 0.011921 | -1.89356 |
| MRPL23 | -0.5572 | -0.28643 | -3.13209 | 0.00238 | 0.011976 | -1.8999 |
| PRUNE2 | -0.73956 | -0.28643 | -3.11365 | 0.002517 | 0.012541 | -1.95084 |
| RNF19B | 0.537039 | 0.166612 | 3.111937 | 0.00253 | 0.012586 | -1.95557 |
| C1R | -0.52125 | 0.755546 | -3.11003 | 0.002545 | 0.012639 | -1.96081 |
| FYCO1 | -0.51084 | -0.13626 | -3.10296 | 0.0026 | 0.012849 | -1.98026 |
| PALLD | -0.62129 | -0.42487 | -3.09918 | 0.00263 | 0.012953 | -1.99064 |
| LDHB | -0.50847 | -0.18001 | -3.09781 | 0.002641 | 0.012977 | -1.99442 |
| S100A8 | 0.768104 | -0.13515 | 3.065157 | 0.002915 | 0.013937 | -2.08367 |
| HADH | -0.50706 | -0.2399 | -3.06138 | 0.002948 | 0.014046 | -2.09396 |
| DUSP2 | 0.574532 | 0.338751 | 3.05779 | 0.00298 | 0.014159 | -2.1037 |
| CCL3 | 0.720827 | 0.191296 | 3.052862 | 0.003024 | 0.014336 | -2.11708 |
| CSRP1 | -0.55729 | -0.3185 | -3.02744 | 0.003263 | 0.015154 | -2.18584 |
| LMCD1 | -0.50991 | -0.13086 | -3.02259 | 0.003311 | 0.015288 | -2.1989 |
| MAP3K7CL | -0.52649 | -0.09664 | -3.01692 | 0.003367 | 0.01548 | -2.21415 |
| VAMP5 | -0.52862 | -0.19106 | -3.01496 | 0.003387 | 0.015545 | -2.2194 |
| ERGIC1 | -0.5031 | -0.20096 | -2.99544 | 0.003589 | 0.016197 | -2.27171 |
| CYP1B1 | -0.52447 | -0.00588 | -2.97395 | 0.003824 | 0.01706 | -2.32897 |
| PTPRCAP | 0.64426 | 0.186902 | 2.967221 | 0.003901 | 0.017307 | -2.34684 |
| NDUFA4L2 | -0.61536 | 0.014394 | -2.96524 | 0.003924 | 0.01738 | -2.3521 |
| IFI6 | -0.51743 | 0.245388 | -2.96455 | 0.003931 | 0.017407 | -2.35392 |
| RPL10A | -0.64052 | 0.495306 | -2.95277 | 0.00407 | 0.017853 | -2.38509 |
| NCF2 | 0.518748 | 0.075762 | 2.949795 | 0.004106 | 0.017966 | -2.39294 |
| SBSPON | -0.67903 | -0.33255 | -2.94961 | 0.004108 | 0.017969 | -2.39343 |
| TUBB4A | 0.508699 | -0.00865 | 2.927513 | 0.004382 | 0.018894 | -2.45157 |
| CR2 | 0.713119 | 0.797917 | 2.9261 | 0.0044 | 0.01895 | -2.45528 |
| HSPB7 | -0.56724 | -0.16399 | -2.92525 | 0.004411 | 0.018979 | -2.4575 |
| ISM1 | -0.52459 | -0.15973 | -2.91699 | 0.004518 | 0.019331 | -2.47913 |
| CARD8 | -0.50146 | 0.045633 | -2.90764 | 0.004643 | 0.019766 | -2.50356 |
| FAM180B | 0.516687 | 0.074527 | 2.907277 | 0.004648 | 0.019778 | -2.50451 |
| CPVL | 0.508391 | 0.007112 | 2.895024 | 0.004816 | 0.020383 | -2.53641 |
| DLD | 0.595079 | -0.121 | 2.881776 | 0.005004 | 0.020959 | -2.57079 |
| GANAB | -0.51984 | -0.39029 | -2.86931 | 0.005187 | 0.021501 | -2.60301 |
| SLC25A4 | -0.51463 | 0.007188 | -2.85854 | 0.00535 | 0.021957 | -2.63077 |
| HBG1 | 0.774402 | 0.563335 | 2.847484 | 0.005523 | 0.022512 | -2.65916 |
| MCM5 | 0.589567 | 0.051013 | 2.839134 | 0.005656 | 0.022903 | -2.68055 |
| MFAP4 | -0.54393 | -0.29542 | -2.82139 | 0.00595 | 0.023792 | -2.72583 |
| PPP1R14A | -0.62008 | -0.27611 | -2.79932 | 0.006335 | 0.024987 | -2.78182 |
| OLR1 | 0.615233 | 0.064652 | 2.792995 | 0.006449 | 0.025309 | -2.7978 |
| SELL | 0.56132 | 0.281412 | 2.787045 | 0.006558 | 0.025624 | -2.8128 |
| SLC1A3 | -0.53711 | -0.07864 | -2.78704 | 0.006558 | 0.025624 | -2.81282 |
| IL6 | 0.933859 | 0.527839 | 2.765349 | 0.006972 | 0.026799 | -2.86729 |
| SFRP4 | 0.502563 | 0.518991 | 2.761145 | 0.007054 | 0.027014 | -2.8778 |
| SCRG1 | -0.76796 | -0.20078 | -2.7533 | 0.007211 | 0.02747 | -2.8974 |
| NOV | -0.72698 | -0.45653 | -2.75298 | 0.007218 | 0.027489 | -2.89818 |
| ADH1A | -0.66784 | -0.38063 | -2.7452 | 0.007376 | 0.027948 | -2.91758 |
| DACT3 | -0.60501 | -0.27679 | -2.7423 | 0.007436 | 0.028119 | -2.92478 |
| RPL39 | -0.6728 | -0.63228 | -2.73082 | 0.007678 | 0.028796 | -2.95328 |
| SHROOM3 | -0.50645 | -0.17506 | -2.72493 | 0.007805 | 0.029117 | -2.96784 |
| CHURC1 | -0.53951 | -0.20932 | -2.67711 | 0.008909 | 0.032171 | -3.08523 |
| FRZB | -0.58678 | -0.27922 | -2.67693 | 0.008913 | 0.032171 | -3.08567 |
| CXCL8 | 0.70412 | 0.618409 | 2.671778 | 0.00904 | 0.032486 | -3.0982 |
| SMOC2 | -0.50931 | -0.34071 | -2.66846 | 0.009123 | 0.032709 | -3.10627 |
| SRPX | -0.53725 | -0.09051 | -2.63368 | 0.010031 | 0.035147 | -3.1903 |
| FOSB | 0.789282 | -0.10167 | 2.601866 | 0.010934 | 0.037537 | -3.26636 |
| RPL35A | -0.80295 | -0.68026 | -2.58377 | 0.011479 | 0.038943 | -3.30927 |
| PHLDA1 | 0.520898 | 0.326841 | 2.510599 | 0.013944 | 0.045026 | -3.48017 |
| RPL21 | -0.84487 | -0.62312 | -2.4746 | 0.015323 | 0.048269 | -3.56271 |
| SNAR-A1 | 0.679132 | 0.227435 | 2.453899 | 0.01617 | 0.050349 | -3.60969 |
| RPL26 | -0.92761 | -0.82149 | -2.43833 | 0.016835 | 0.051889 | -3.64481 |
| NDUFA1 | -0.53623 | -0.36829 | -2.42161 | 0.017576 | 0.053612 | -3.68231 |
| TIMM17A | 0.508856 | 0.11546 | 2.395391 | 0.018796 | 0.056352 | -3.74066 |
| TXNIP | -0.55839 | -0.3072 | -2.39432 | 0.018847 | 0.056488 | -3.74303 |
| IER3 | 0.541074 | 0.184301 | 2.38098 | 0.019498 | 0.057898 | -3.7725 |
| HSPA1A | -0.64087 | 0.037009 | -2.37221 | 0.019937 | 0.058813 | -3.7918 |
| SBDS | -0.50091 | -0.14725 | -2.36987 | 0.020056 | 0.059089 | -3.79694 |
| CIDEA | 0.685766 | 0.36582 | 2.356388 | 0.020751 | 0.060645 | -3.82644 |
| FCER1A | 0.511165 | 0.243635 | 2.332097 | 0.022057 | 0.06356 | -3.87924 |
| KCNMB1 | -0.56206 | -0.45673 | -2.33125 | 0.022105 | 0.063667 | -3.88108 |
| HLA-DRB6 | 0.507438 | 0.437824 | 2.301613 | 0.0238 | 0.067049 | -3.94483 |
| MYH10 | -0.52099 | -0.51747 | -2.29352 | 0.024282 | 0.068083 | -3.9621 |
| RPL7A | -0.58125 | -0.5432 | -2.27433 | 0.025462 | 0.070746 | -4.00288 |
| MYL9 | -0.5127 | 0.333281 | -2.12243 | 0.036707 | 0.093177 | -4.31482 |
| SERPINE1 | 0.597974 | 0.119455 | 2.081048 | 0.040435 | 0.100284 | -4.39647 |
| COL1A1 | 0.521966 | 0.253644 | 2.042748 | 0.044172 | 0.107327 | -4.47074 |
| BCYRN1 | 0.543447 | 0.076478 | 2.034189 | 0.045046 | 0.108726 | -4.48717 |

**Supplementary Table 4. The results of GSVA enrichment analysis of the differential expression genes from the high and low PRGs groups patients.**

|  | **logFC** | **Average expression** | **T value** | **P. Value** | **Adjust P. Value** | **Bold** |
| --- | --- | --- | --- | --- | --- | --- |
| PID_INTEGRIN2_PATHWAY | 0.261795 | -0.00905 | 5.31748 | 1.35E-06 | 0.008582 | 4.942713 |
| WP_LUNG_FIBROSIS | 0.166207 | -0.22089 | 4.982884 | 4.80E-06 | 0.010915 | 3.833902 |
| WP_CILIOPATHIES | -0.16847 | -0.03351 | -4.94834 | 5.47E-06 | 0.010915 | 3.720971 |
| BLANCO_MELO_COVID19_SARS_COV_2_INFECTION_A594_CELLS_UP | 0.155771 | -0.13724 | 4.887533 | 6.86E-06 | 0.010915 | 3.522969 |
| ONDER_CDH1_TARGETS_2_DN | 0.106112 | -0.07719 | 4.326263 | 5.26E-05 | 0.042781 | 1.74746 |
| REACTOME_CLASS_I_PEROXISOMAL_MEMBRANE_PROTEIN_IMPORT | -0.22933 | -0.05282 | -4.3249 | 5.29E-05 | 0.042781 | 1.743284 |
| GHANDHI_DIRECT_IRRADIATION_UP | 0.181662 | -0.14928 | 4.322154 | 5.34E-05 | 0.042781 | 1.734849 |
| AUJLA_IL22_AND_IL17A_SIGNALING | 0.229701 | -0.20924 | 4.316181 | 5.45E-05 | 0.042781 | 1.716527 |
| BIOCARTA_STEM_PATHWAY | 0.337421 | 0.014618 | 4.27338 | 6.34E-05 | 0.042781 | 1.585619 |
| ONDER_CDH1_TARGETS_3_DN | 0.21645 | -0.02557 | 4.25661 | 6.72E-05 | 0.042781 | 1.534512 |
| ABBUD_LIF_SIGNALING_2_DN | 0.262061 | -0.11591 | 4.160369 | 9.40E-05 | 0.054379 | 1.243284 |
| GAUSSMANN_MLL_AF4_FUSION_TARGETS_D_UP | 0.14196 | -0.06144 | 4.082082 | 0.000123 | 0.065289 | 1.009053 |
| LIAN_LIPA_TARGETS_6M | 0.182402 | -0.16258 | 4.010856 | 0.000157 | 0.068325 | 0.798104 |
| HASEGAWA_TUMORIGENESIS_BY_RET_C634R | 0.273557 | -0.10805 | 3.978081 | 0.000175 | 0.068325 | 0.701743 |
| TAKEDA_TARGETS_OF_NUP98_HOXA9_FUSION_6HR_DN | 0.192329 | -0.10115 | 3.957499 | 0.000188 | 0.068325 | 0.641461 |
| BIOCARTA_DC_PATHWAY | 0.244759 | -0.01638 | 3.954575 | 0.00019 | 0.068325 | 0.632913 |
| WP_GLIAL_CELL_DIFFERENTIATION | 0.264407 | -0.00582 | 3.94093 | 0.000199 | 0.068325 | 0.593067 |
| CHARAFE_BREAST_CANCER_BASAL_VS_MESENCHYMAL_UP | 0.118989 | -0.07807 | 3.900593 | 0.000228 | 0.068325 | 0.47574 |
| REACTOME_CHYLOMICRON_CLEARANCE | 0.260301 | -0.00764 | 3.895779 | 0.000232 | 0.068325 | 0.461785 |
| BIOCARTA_NOS1_PATHWAY | -0.20515 | -0.06251 | -3.86982 | 0.000253 | 0.068325 | 0.386716 |
| VERRECCHIA_RESPONSE_TO_TGFB1_C2 | 0.302067 | -0.02042 | 3.86206 | 0.000259 | 0.068325 | 0.364328 |
| GALINDO_IMMUNE_RESPONSE_TO_ENTEROTOXIN | 0.196313 | -0.22596 | 3.857572 | 0.000263 | 0.068325 | 0.351393 |
| GRAHAM_CML_QUIESCENT_VS_CML_DIVIDING_UP | 0.250295 | -0.07605 | 3.85234 | 0.000268 | 0.068325 | 0.336327 |
| WP_HEMATOPOIETIC_STEM_CELL_DIFFERENTIATION | 0.158711 | -0.13586 | 3.849977 | 0.00027 | 0.068325 | 0.329525 |
| ALTEMEIER_RESPONSE_TO_LPS_WITH_MECHANICAL_VENTILATION | 0.192058 | -0.11874 | 3.834868 | 0.000284 | 0.068325 | 0.2861 |
| REACTOME_HDL_REMODELING | 0.267421 | -0.00679 | 3.828238 | 0.00029 | 0.068325 | 0.267077 |
| PIONTEK_PKD1_TARGETS_UP | 0.142793 | -0.17038 | 3.81592 | 0.000302 | 0.068325 | 0.231787 |
| VILIMAS_NOTCH1_TARGETS_DN | 0.198269 | -0.20865 | 3.808593 | 0.000309 | 0.068325 | 0.210827 |
| THILLAINADESAN_ZNF217_TARGETS_DN | -0.37043 | -0.01947 | -3.80679 | 0.000311 | 0.068325 | 0.205675 |
| ZWANG_DOWN_BY_2ND_EGF_PULSE | -0.09718 | -0.0917 | -3.77813 | 0.000342 | 0.06892 | 0.123941 |
| REACTOME_BBSOME_MEDIATED_CARGO_TARGETING_TO_CILIUM | -0.27289 | -0.01151 | -3.77481 | 0.000346 | 0.06892 | 0.114508 |
| REACTOME_SCAVENGING_OF_HEME_FROM_PLASMA | 0.260666 | 0.003048 | 3.762843 | 0.00036 | 0.06892 | 0.080508 |
| TAKEDA_TARGETS_OF_NUP98_HOXA9_FUSION_10D_DN | 0.151514 | -0.15208 | 3.754941 | 0.000369 | 0.06892 | 0.058097 |
| KEGG_BASAL_TRANSCRIPTION_FACTORS | -0.18024 | -0.20424 | -3.75344 | 0.000371 | 0.06892 | 0.053833 |
| BAFNA_MUC4_TARGETS_UP | 0.406131 | 0.004633 | 3.741257 | 0.000386 | 0.06892 | 0.019356 |
| JEPSEN_SMRT_TARGETS | 0.171563 | -0.00978 | 3.730367 | 0.0004 | 0.06892 | -0.01141 |
| XU_HGF_TARGETS_REPRESSED_BY_AKT1_UP | 0.281532 | -0.03015 | 3.726803 | 0.000405 | 0.06892 | -0.02147 |
| WP_GENES_RELATED_TO_PRIMARY_CILIUM_DEVELOPMENT_BASED_ON_CRISPR | -0.17137 | -0.02522 | -3.72196 | 0.000412 | 0.06892 | -0.03514 |
| BLANCO_MELO_COVID19_BRONCHIAL_EPITHELIAL_CELLS_SARS_COV_2_INFECTION_UP | 0.124097 | -0.22415 | 3.707209 | 0.000432 | 0.070465 | -0.07666 |
| HESS_TARGETS_OF_HOXA9_AND_MEIS1_DN | 0.203236 | -0.04896 | 3.684384 | 0.000465 | 0.074004 | -0.14072 |
| REACTOME_SYNTHESIS_OF_PI | 0.262288 | -0.01841 | 3.668905 | 0.000489 | 0.075429 | -0.18403 |
| WP_IL1_AND_MEGAKARYOCYTES_IN_OBESITY | 0.269263 | -0.00295 | 3.647239 | 0.000525 | 0.075429 | -0.24445 |
| WP_FAMILIAL_HYPERLIPIDEMIA_TYPE_3 | 0.238269 | -0.01636 | 3.638118 | 0.00054 | 0.075429 | -0.26981 |
| NAKAMURA_BRONCHIAL_AND_BRONCHIOLAR_EPITHELIA | 0.306714 | 0.005757 | 3.635732 | 0.000545 | 0.075429 | -0.27644 |
| LIANG_SILENCED_BY_METHYLATION_2 | 0.164253 | -0.09191 | 3.629347 | 0.000556 | 0.075429 | -0.29417 |
| LINDSTEDT_DENDRITIC_CELL_MATURATION_A | 0.17407 | -0.17498 | 3.628338 | 0.000558 | 0.075429 | -0.29697 |
| REACTOME_CHEMOKINE_RECEPTORS_BIND_CHEMOKINES | 0.147869 | -0.126 | 3.623185 | 0.000567 | 0.075429 | -0.31126 |
| WARTERS_IR_RESPONSE_5GY | 0.121908 | -0.1555 | 3.621586 | 0.00057 | 0.075429 | -0.31569 |
| MIKKELSEN_ES_LCP_WITH_H3K4ME3_AND_H3K27ME3 | 0.331727 | -0.09352 | 3.60969 | 0.000592 | 0.075429 | -0.34862 |
| REACTOME_THE_FATTY_ACID_CYCLING_MODEL | 0.305098 | -0.02151 | 3.60215 | 0.000607 | 0.075429 | -0.36946 |
| HOLLEMAN_PREDNISOLONE_RESISTANCE_B_ALL_DN | 0.280697 | -0.00789 | 3.59737 | 0.000616 | 0.075429 | -0.38265 |
| HAHTOLA_MYCOSIS_FUNGOIDES_CD4_UP | 0.127939 | -0.47134 | 3.581574 | 0.000648 | 0.075429 | -0.42618 |
| KRIEG_HYPOXIA_VIA_KDM3A | 0.209741 | -0.04997 | 3.578416 | 0.000655 | 0.075429 | -0.43486 |
| AGARWAL_AKT_PATHWAY_TARGETS | 0.321381 | -0.0265 | 3.576269 | 0.000659 | 0.075429 | -0.44076 |
| WP_NUCLEAR_RECEPTORS_METAPATHWAY | 0.095694 | -0.13158 | 3.56862 | 0.000675 | 0.075429 | -0.46177 |
| REACTOME_DEFECTS_IN_BIOTIN_BTN_METABOLISM | -0.31221 | -0.11211 | -3.56569 | 0.000682 | 0.075429 | -0.46981 |
| WP_KETONE_BODIES_SYNTHESIS_AND_DEGRADATION | -0.3201 | -0.15278 | -3.56351 | 0.000686 | 0.075429 | -0.47579 |
| BLANCO_MELO_RESPIRATORY_SYNCYTIAL_VIRUS_INFECTION_A594_CELLS_UP | 0.139616 | -0.13177 | 3.560556 | 0.000693 | 0.075429 | -0.4839 |
| REACTOME_BINDING_AND_UPTAKE_OF_LIGANDS_BY_SCAVENGER_RECEPTORS | 0.198336 | -0.05407 | 3.554731 | 0.000706 | 0.075429 | -0.49985 |
| WP_TP53_NETWORK | 0.184605 | -0.00196 | 3.549849 | 0.000717 | 0.075429 | -0.51322 |
| HERNANDEZ_ABERRANT_MITOSIS_BY_DOCETACEL_4NM_UP | 0.197611 | -0.0502 | 3.546432 | 0.000725 | 0.075429 | -0.52256 |
| BIOCARTA_LYM_PATHWAY | 0.208517 | -0.00366 | 3.54214 | 0.000735 | 0.075429 | -0.53429 |
| BLANCO_MELO_COVID19_SARS_COV_2_POS_PATIENT_LUNG_TISSUE_UP | 0.140858 | -0.12254 | 3.534986 | 0.000752 | 0.075939 | -0.55382 |
| WP_PLATELETMEDIATED_INTERACTIONS_WITH_VASCULAR_AND_CIRCULATING_CELLS | 0.199276 | -0.06249 | 3.522206 | 0.000783 | 0.076433 | -0.58865 |
| WP_OVERVIEW_OF_PROINFLAMMATORY_AND_PROFIBROTIC_MEDIATORS | 0.140426 | -0.17707 | 3.522188 | 0.000783 | 0.076433 | -0.58869 |
| ZHANG_RESPONSE_TO_IKK_INHIBITOR_AND_TNF_UP | 0.149356 | -0.10892 | 3.514296 | 0.000803 | 0.076433 | -0.61016 |
| REACTOME_CHYLOMICRON_REMODELING | 0.262578 | 0.011779 | 3.509049 | 0.000816 | 0.076433 | -0.62441 |
| LIU_IL13_MEMORY_MODEL_UP | 0.238961 | -0.06276 | 3.508844 | 0.000817 | 0.076433 | -0.62497 |
| REACTOME_ACTIVATION_OF_MATRIX_METALLOPROTEINASES | 0.19406 | -0.1071 | 3.478005 | 0.0009 | 0.082036 | -0.70846 |
| REACTOME_HYALURONAN_BIOSYNTHESIS_AND_EXPORT | 0.267042 | 0.010864 | 3.477229 | 0.000902 | 0.082036 | -0.71055 |
| WANG_TNF_TARGETS | 0.206482 | -0.08844 | 3.455797 | 0.000965 | 0.086235 | -0.76828 |
| WP_STATIN_INHIBITION_OF_CHOLESTEROL_PRODUCTION | 0.134712 | -0.10908 | 3.452362 | 0.000976 | 0.086235 | -0.77751 |
| REACTOME_INTERLEUKIN_10_SIGNALING | 0.158109 | -0.22562 | 3.447616 | 0.00099 | 0.086327 | -0.79025 |
| BARRIER_CANCER_RELAPSE_TUMOR_SAMPLE_DN | 0.236438 | 0.025241 | 3.435459 | 0.001029 | 0.088461 | -0.82284 |
| BIOCARTA_IL5_PATHWAY | 0.096977 | -0.55481 | 3.424806 | 0.001063 | 0.089416 | -0.85133 |
| REACTOME_DECTIN_2_FAMILY | 0.163871 | -0.08314 | 3.419347 | 0.001082 | 0.089416 | -0.86591 |
| WU_ALZHEIMER_DISEASE_UP | 0.26905 | 0.00075 | 3.419286 | 0.001082 | 0.089416 | -0.86607 |
| GRAHAM_CML_QUIESCENT_VS_NORMAL_DIVIDING_UP | 0.170456 | -0.07262 | 3.40839 | 0.001119 | 0.09053 | -0.89512 |
| ZHAN_VARIABLE_EARLY_DIFFERENTIATION_GENES_UP | 0.158867 | -0.23234 | 3.40707 | 0.001124 | 0.09053 | -0.89864 |
| STEARMAN_TUMOR_FIELD_EFFECT_UP | 0.202262 | -0.17973 | 3.391154 | 0.001181 | 0.092412 | -0.94095 |
| BIOCARTA_TERC_PATHWAY | -0.32994 | -0.02823 | -3.38889 | 0.001189 | 0.092412 | -0.94695 |
| REACTOME_PTK6_PROMOTES_HIF1A_STABILIZATION | 0.269932 | -0.00388 | 3.378114 | 0.001229 | 0.092412 | -0.97551 |
| KEGG_CYTOKINE_CYTOKINE_RECEPTOR_INTERACTION | 0.10533 | -0.11921 | 3.376426 | 0.001236 | 0.092412 | -0.97998 |
| BLANCO_MELO_COVID19_SARS_COV_2_INFECTION_CALU3_CELLS_UP | 0.111753 | -0.14048 | 3.375109 | 0.001241 | 0.092412 | -0.98346 |
| REACTOME_CARGO_TRAFFICKING_TO_THE_PERICILIARY_MEMBRANE | -0.21755 | -0.02956 | -3.37293 | 0.001249 | 0.092412 | -0.98922 |
| WP_TYROBP_CAUSAL_NETWORK_IN_MICROGLIA | 0.215322 | -0.15387 | 3.368771 | 0.001265 | 0.092412 | -1.00022 |
| BIOCARTA_NEUTROPHIL_PATHWAY | 0.322003 | -0.01405 | 3.366389 | 0.001275 | 0.092412 | -1.00651 |
| HINATA_NFKB_TARGETS_KERATINOCYTE_UP | 0.167558 | -0.17554 | 3.363915 | 0.001285 | 0.092412 | -1.01304 |
| BLANCO_MELO_BRONCHIAL_EPITHELIAL_CELLS_INFLUENZA_A_INFECTION_UP | 0.098988 | -0.19807 | 3.361956 | 0.001292 | 0.092412 | -1.01822 |
| MISHRA_CARCINOMA_ASSOCIATED_FIBROBLAST_UP | 0.234776 | -0.05442 | 3.353401 | 0.001327 | 0.093036 | -1.04077 |
| PID_FRA_PATHWAY | 0.21781 | -0.04223 | 3.342696 | 0.001371 | 0.093036 | -1.06894 |
| WORSCHECH_TUMOR_EVASION_AND_TOLEROGENICITY_UP | 0.167392 | -0.06261 | 3.341013 | 0.001378 | 0.093036 | -1.07336 |
| SMID_BREAST_CANCER_RELAPSE_IN_BONE_DN | 0.085299 | -0.13338 | 3.340624 | 0.00138 | 0.093036 | -1.07438 |
| BIOCARTA_FIBRINOLYSIS_PATHWAY | 0.248488 | -0.01049 | 3.340071 | 0.001382 | 0.093036 | -1.07583 |
| AMIT_EGF_RESPONSE_120_HELA | 0.191795 | -0.11199 | 3.338587 | 0.001389 | 0.093036 | -1.07973 |
| GOLUB_ALL_VS_AML_DN | 0.202581 | -0.18231 | 3.333014 | 0.001413 | 0.093657 | -1.09436 |
| VALK_AML_CLUSTER_5 | 0.147208 | -0.34146 | 3.324766 | 0.001449 | 0.095067 | -1.11598 |
| PEDERSEN_METASTASIS_BY_ERBB2_ISOFORM_1 | 0.221174 | -0.0089 | 3.308007 | 0.001525 | 0.097031 | -1.15979 |
| WP_KREBS_CYCLE_DISORDERS | -0.39597 | -0.04502 | -3.30741 | 0.001528 | 0.097031 | -1.16134 |
| TONKS_TARGETS_OF_RUNX1_RUNX1T1_FUSION_HSC_DN | 0.126572 | -0.10043 | 3.306703 | 0.001531 | 0.097031 | -1.16319 |
| XIE_LT_HSC_S1PR3_OE_UP | 0.184854 | -0.01031 | 3.304883 | 0.00154 | 0.097031 | -1.16794 |
| BROWNE_HCMV_INFECTION_2HR_UP | 0.186896 | -0.06417 | 3.291952 | 0.001602 | 0.099476 | -1.20161 |
| WP_VITAMIN_A_AND_CAROTENOID_METABOLISM | 0.139603 | -0.13898 | 3.287378 | 0.001624 | 0.099476 | -1.21351 |
| BIOCARTA_RHO_PATHWAY | 0.204928 | -0.05582 | 3.287127 | 0.001626 | 0.099476 | -1.21416 |
| RASHI_NFKB1_TARGETS | 0.213031 | -0.14832 | 3.277709 | 0.001673 | 0.101225 | -1.2386 |
| REACTOME_SMAC_XIAP_REGULATED_APOPTOTIC_RESPONSE | -0.29099 | 0.005566 | -3.27499 | 0.001687 | 0.101225 | -1.24566 |
| MARSON_FOXP3_CORE_DIRECT_TARGETS | 0.195641 | -0.04613 | 3.269315 | 0.001716 | 0.101225 | -1.26035 |
| MUELLER_METHYLATED_IN_GLIOBLASTOMA | 0.134274 | -0.15985 | 3.266579 | 0.00173 | 0.101225 | -1.26743 |
| FONTAINE_PAPILLARY_THYROID_CARCINOMA_DN | 0.098128 | -0.08757 | 3.263722 | 0.001745 | 0.101225 | -1.27482 |
| REACTOME_OTHER_SEMAPHORIN_INTERACTIONS | 0.135614 | -0.09159 | 3.262936 | 0.00175 | 0.101225 | -1.27685 |
| MIKKELSEN_MEF_LCP_WITH_H3K4ME3 | 0.112941 | -0.17884 | 3.257264 | 0.00178 | 0.102053 | -1.29151 |
| ZHANG_RESPONSE_TO_IKK_INHIBITOR_AND_TNF_DN | -0.13469 | -0.01942 | -3.25149 | 0.001811 | 0.102925 | -1.3064 |
| WP_BURN_WOUND_HEALING | 0.12692 | -0.10518 | 3.241695 | 0.001866 | 0.105081 | -1.33163 |
| DALESSIO_TSA_RESPONSE | 0.20221 | -0.15234 | 3.229995 | 0.001933 | 0.107058 | -1.3617 |
| HINATA_NFKB_TARGETS_KERATINOCYTE_DN | 0.137045 | -0.03008 | 3.229702 | 0.001935 | 0.107058 | -1.36246 |
| CHO_NR4A1_TARGETS | 0.137826 | -0.06771 | 3.210128 | 0.002052 | 0.112573 | -1.41259 |
| XU_HGF_TARGETS_INDUCED_BY_AKT1_48HR_UP | 0.240143 | -0.02227 | 3.205524 | 0.00208 | 0.113165 | -1.42435 |
| WP_MITOCHONDRIAL_FATTY_ACID_SYNTHESIS_PATHWAY | -0.28182 | -0.15556 | -3.20268 | 0.002098 | 0.113167 | -1.43161 |
| PID_ERB_GENOMIC_PATHWAY | -0.18299 | -0.12611 | -3.19928 | 0.00212 | 0.113367 | -1.44029 |
| SWEET_KRAS_TARGETS_DN | 0.105567 | -0.04868 | 3.191202 | 0.002172 | 0.114456 | -1.46085 |
| HANSON_HRAS_SIGNALING_VIA_NFKB | 0.19268 | -0.04573 | 3.190518 | 0.002176 | 0.114456 | -1.4626 |
| WP_OREXIN_RECEPTOR_PATHWAY | 0.106476 | -0.11623 | 3.180542 | 0.002242 | 0.116954 | -1.48795 |
| AIGNER_ZEB1_TARGETS | 0.130689 | -0.08347 | 3.17184 | 0.002301 | 0.117388 | -1.51002 |
| WP_CYTOKINES_AND_INFLAMMATORY_RESPONSE | 0.115589 | -0.42441 | 3.170179 | 0.002312 | 0.117388 | -1.51423 |
| LIEN_BREAST_CARCINOMA_METAPLASTIC_VS_DUCTAL_UP | 0.170605 | -0.02547 | 3.168475 | 0.002324 | 0.117388 | -1.51855 |
| REACTOME_DEFECTS_IN_VITAMIN_AND_COFACTOR_METABOLISM | -0.20614 | -0.05715 | -3.16695 | 0.002335 | 0.117388 | -1.5224 |
| WP_SARSCOV2_ALTERING_ANGIOGENESIS_VIA_NRP1 | 0.281775 | -0.02179 | 3.165834 | 0.002343 | 0.117388 | -1.52523 |
| MATTHEWS_SKIN_CARCINOGENESIS_VIA_JUN | 0.18935 | -0.00991 | 3.142794 | 0.002509 | 0.120022 | -1.58339 |
| REACTOME_CELL_SURFACE_INTERACTIONS_AT_THE_VASCULAR_WALL | 0.0976 | -0.08635 | 3.142194 | 0.002513 | 0.120022 | -1.5849 |
| LIAN_LIPA_TARGETS_3M | 0.157755 | -0.18198 | 3.135817 | 0.002561 | 0.120022 | -1.60094 |
| REACTOME_SYNTHESIS_OF_DIPHTHAMIDE_EEF2 | -0.28638 | -0.00467 | -3.13483 | 0.002569 | 0.120022 | -1.60343 |
| MARTIN_NFKB_TARGETS_UP | 0.164654 | -0.03217 | 3.134738 | 0.002569 | 0.120022 | -1.60365 |
| MARTINELLI_IMMATURE_NEUTROPHIL_DN | 0.266146 | -0.0161 | 3.134382 | 0.002572 | 0.120022 | -1.60454 |
| KANG_GLIS3_TARGETS | 0.138894 | -0.09297 | 3.133953 | 0.002575 | 0.120022 | -1.60562 |
| REACTOME_DISSOLUTION_OF_FIBRIN_CLOT | 0.23116 | -0.08451 | 3.133639 | 0.002578 | 0.120022 | -1.60641 |
| PHONG_TNF_TARGETS_UP | 0.204888 | -0.15295 | 3.132522 | 0.002586 | 0.120022 | -1.60922 |
| REACTOME_MODULATION_BY_MTB_OF_HOST_IMMUNE_SYSTEM | 0.275832 | -0.11315 | 3.130403 | 0.002602 | 0.120022 | -1.61454 |
| KIM_RESPONSE_TO_TSA_AND_DECITABINE_UP | 0.094841 | -0.06851 | 3.130375 | 0.002603 | 0.120022 | -1.61461 |
| REACTOME_SCAVENGING_BY_CLASS_B_RECEPTORS | 0.235066 | -0.00645 | 3.121515 | 0.002672 | 0.121339 | -1.63683 |
| LEE_AGING_CEREBELLUM_DN | 0.094775 | -0.05848 | 3.11539 | 0.00272 | 0.121339 | -1.65216 |
| WP_VITAMIN_D_RECEPTOR_PATHWAY | 0.080357 | -0.14995 | 3.115285 | 0.002721 | 0.121339 | -1.65242 |
| REACTOME_RNA_POLYMERASE_III_TRANSCRIPTION_INITIATION_FROM_TYPE_3_PROMOTER | -0.22866 | -0.04944 | -3.11521 | 0.002722 | 0.121339 | -1.65262 |
| XU_HGF_SIGNALING_NOT_VIA_AKT1_6HR | 0.192001 | -0.10794 | 3.114631 | 0.002727 | 0.121339 | -1.65406 |
| REACTOME_REGULATION_OF_GENE_EXPRESSION_IN_ENDOCRINE_COMMITTED_NEUROG3_PROGENITOR_CELLS | 0.333608 | 0.033323 | 3.105201 | 0.002803 | 0.123892 | -1.67762 |
| WP_VITAMIN_D_METABOLISM | 0.152875 | -0.3123 | 3.093817 | 0.002899 | 0.125386 | -1.70599 |
| REACTOME_DISEASES_OF_DNA_REPAIR | -0.15475 | -0.02335 | -3.09229 | 0.002912 | 0.125386 | -1.7098 |
| MARIADASON_RESPONSE_TO_BUTYRATE_CURCUMIN_SULINDAC_TSA_1 | 0.262943 | -0.03415 | 3.087167 | 0.002956 | 0.125386 | -1.72253 |
| RODRIGUES_NTN1_TARGETS_UP | -0.1763 | 0.000895 | -3.08651 | 0.002962 | 0.125386 | -1.72417 |
| TORCHIA_TARGETS_OF_EWSR1_FLI1_FUSION_TOP20_UP | 0.123587 | -0.08535 | 3.086503 | 0.002962 | 0.125386 | -1.72418 |
| PARENT_MTOR_SIGNALING_DN | 0.132328 | -0.02392 | 3.085862 | 0.002967 | 0.125386 | -1.72578 |
| GHANDHI_BYSTANDER_IRRADIATION_UP | 0.157488 | -0.0179 | 3.083834 | 0.002985 | 0.125386 | -1.73082 |
| SATO_SILENCED_EPIGENETICALLY_IN_PANCREATIC_CANCER | 0.110136 | -0.2461 | 3.082714 | 0.002995 | 0.125386 | -1.7336 |
| WP_BIOTIN_METABOLISM_INCLUDING_IEMS | -0.22044 | -0.24786 | -3.0793 | 0.003025 | 0.125783 | -1.74207 |
| VILIMAS_NOTCH1_TARGETS_UP | 0.189384 | -0.14549 | 3.077173 | 0.003044 | 0.125783 | -1.74734 |
| TARTE_PLASMA_CELL_VS_PLASMABLAST_UP | 0.070808 | -0.21505 | 3.073189 | 0.003079 | 0.126437 | -1.75722 |
| WP_INTRAFLAGELLAR_TRANSPORT_PROTEINS_BINDING_TO_DYNEIN | -0.22441 | 0.00081 | -3.06828 | 0.003124 | 0.126688 | -1.76937 |
| FERRANDO_TAL1_NEIGHBORS | 0.198102 | -0.00737 | 3.065036 | 0.003154 | 0.126688 | -1.7774 |
| SCIAN_INVERSED_TARGETS_OF_TP53_AND_TP73_UP | 0.210665 | 0.023273 | 3.062046 | 0.003181 | 0.126688 | -1.78478 |
| BIOCARTA_LEPTIN_PATHWAY | -0.20422 | -0.08754 | -3.0607 | 0.003194 | 0.126688 | -1.78811 |
| NABA_ECM_AFFILIATED | 0.094726 | -0.16471 | 3.059918 | 0.003201 | 0.126688 | -1.79004 |
| ABE_VEGFA_TARGETS_30MIN | 0.133496 | -0.18576 | 3.059512 | 0.003205 | 0.126688 | -1.79104 |
| RICKMAN_METASTASIS_UP | -0.1483 | -0.08727 | -3.0527 | 0.003269 | 0.128432 | -1.80785 |
| NABA_ECM_REGULATORS | 0.120045 | -0.09247 | 3.046565 | 0.003328 | 0.129932 | -1.82296 |
| YAMASHITA_METHYLATED_IN_PROSTATE_CANCER | 0.121674 | -0.02174 | 3.044496 | 0.003348 | 0.129932 | -1.82805 |
| SEKI_INFLAMMATORY_RESPONSE_LPS_UP | 0.177972 | -0.10006 | 3.038646 | 0.003406 | 0.131107 | -1.84244 |
| MCLACHLAN_DENTAL_CARIES_UP | 0.131773 | -0.28509 | 3.034254 | 0.00345 | 0.131107 | -1.85322 |
| WP_FAMILIAL_HYPERLIPIDEMIA_TYPE_5 | 0.17297 | -0.00347 | 3.031656 | 0.003476 | 0.131107 | -1.85959 |
| ONO_AML1_TARGETS_DN | 0.156986 | -0.04846 | 3.031612 | 0.003476 | 0.131107 | -1.8597 |
| FONTAINE_THYROID_TUMOR_UNCERTAIN_MALIGNANCY_DN | 0.129971 | -0.10175 | 3.031061 | 0.003482 | 0.131107 | -1.86106 |
| SA_MMP_CYTOKINE_CONNECTION | 0.142511 | -0.31824 | 3.026234 | 0.003531 | 0.132173 | -1.87288 |
| REACTOME_TRANSPORT_OF_NUCLEOTIDE_SUGARS | -0.24876 | -0.01142 | -3.0205 | 0.00359 | 0.133142 | -1.88692 |
| IIZUKA_LIVER_CANCER_PROGRESSION_G2_G3_DN | 0.253478 | -0.00329 | 3.019675 | 0.003598 | 0.133142 | -1.88894 |
| REACTOME_PEPTIDE_LIGAND_BINDING_RECEPTORS | 0.145842 | -0.0505 | 3.013483 | 0.003663 | 0.134764 | -1.90407 |
| AZARE_NEOPLASTIC_TRANSFORMATION_BY_STAT3_DN | 0.161942 | 0.007445 | 3.009351 | 0.003707 | 0.135446 | -1.91416 |
| MUELLER_COMMON_TARGETS_OF_AML_FUSIONS_DN | 0.125254 | -0.06646 | 3.007757 | 0.003725 | 0.135446 | -1.91804 |
| LU_TUMOR_ANGIOGENESIS_UP | 0.185407 | -0.00156 | 2.999493 | 0.003814 | 0.137021 | -1.93817 |
| REACTOME_RNA_POLYMERASE_III_TRANSCRIPTION_TERMINATION | -0.23078 | -0.01751 | -2.99712 | 0.003841 | 0.137021 | -1.94394 |
| NOJIMA_SFRP2_TARGETS_DN | 0.163537 | -0.0083 | 2.997064 | 0.003841 | 0.137021 | -1.94408 |
| REACTOME_SENSORY_PROCESSING_OF_SOUND_BY_OUTER_HAIR_CELLS_OF_THE_COCHLEA | 0.137439 | 0.010433 | 2.995913 | 0.003854 | 0.137021 | -1.94688 |
| ZHOU_INFLAMMATORY_RESPONSE_LPS_UP | 0.086717 | -0.11866 | 2.98276 | 0.004003 | 0.141515 | -1.97881 |
| WP_APOE_AND_MIR146_IN_INFLAMMATION_AND_ATHEROSCLEROSIS | 0.269345 | -0.00659 | 2.978868 | 0.004048 | 0.142314 | -1.98824 |
| KANG_AR_TARGETS_DN | 0.188202 | -0.01242 | 2.976356 | 0.004077 | 0.142557 | -1.99432 |
| DOANE_BREAST_CANCER_ESR1_DN | 0.129402 | -0.05005 | 2.971336 | 0.004136 | 0.143834 | -2.00646 |
| SAENZ_DETOX_PATHWAY_AND_CARCINOGENESIS_DN | 0.150533 | -0.28733 | 2.969013 | 0.004164 | 0.144007 | -2.01207 |
| REACTOME_TP53_REGULATES_TRANSCRIPTION_OF_GENES_INVOLVED_IN_G1_CELL_CYCLE_ARREST | 0.162205 | 0.004418 | 2.96535 | 0.004208 | 0.144739 | -2.02091 |
| REACTOME_DEFECTIVE_CSF2RB_CAUSES_SMDP5 | 0.301396 | 0.015071 | 2.96222 | 0.004245 | 0.145256 | -2.02846 |
| CHEOK_RESPONSE_TO_HD_MTX_UP | 0.156806 | -0.12417 | 2.955045 | 0.004333 | 0.147473 | -2.04575 |
| BROWNE_HCMV_INFECTION_30MIN_DN | 0.073156 | -0.14157 | 2.950868 | 0.004385 | 0.148449 | -2.05579 |
| WP_FOLATE_METABOLISM | 0.106023 | -0.14835 | 2.948876 | 0.00441 | 0.148505 | -2.06058 |
| REACTOME_ORGANELLE_BIOGENESIS_AND_MAINTENANCE | -0.12526 | -0.07617 | -2.93359 | 0.004607 | 0.154295 | -2.09723 |
| LIN_TUMOR_ESCAPE_FROM_IMMUNE_ATTACK | 0.159345 | -0.00297 | 2.931646 | 0.004632 | 0.15434 | -2.1019 |
| PID_IL23_PATHWAY | 0.128324 | -0.166 | 2.925527 | 0.004713 | 0.156229 | -2.11653 |
| PID_REG_GR_PATHWAY | 0.109193 | -0.05735 | 2.908954 | 0.00494 | 0.161503 | -2.15603 |
| REACTOME_TOXICITY_OF_BOTULINUM_TOXIN_TYPE_D_BOTD | -0.29441 | -0.00844 | -2.90776 | 0.004957 | 0.161503 | -2.15886 |
| WANG_ESOPHAGUS_CANCER_PROGRESSION_UP | 0.245013 | 0.010304 | 2.907476 | 0.004961 | 0.161503 | -2.15955 |
| HOLLEMAN_DAUNORUBICIN_B_ALL_UP | 0.200435 | -0.01014 | 2.906149 | 0.004979 | 0.161503 | -2.1627 |
| AMUNDSON_DNA_DAMAGE_RESPONSE_TP53 | 0.20764 | -0.00016 | 2.904729 | 0.004999 | 0.161503 | -2.16607 |
| REACTOME_TRNA_MODIFICATION_IN_THE_MITOCHONDRION | -0.30764 | 0.00188 | -2.90184 | 0.00504 | 0.16183 | -2.17294 |
| REACTOME_PLASMA_LIPOPROTEIN_CLEARANCE | 0.167389 | -0.01169 | 2.90044 | 0.00506 | 0.16183 | -2.17626 |
| REACTOME_SCAVENGING_BY_CLASS_A_RECEPTORS | 0.214531 | -0.01036 | 2.894244 | 0.00515 | 0.163747 | -2.19095 |
| SMID_BREAST_CANCER_RELAPSE_IN_PLEURA_DN | 0.087306 | -0.39743 | 2.892721 | 0.005172 | 0.163747 | -2.19456 |
| RICKMAN_TUMOR_DIFFERENTIATED_WELL_VS_MODERATELY_DN | 0.108523 | -0.07981 | 2.889974 | 0.005212 | 0.164203 | -2.20107 |
| WP_METABOLIC_PATHWAY_OF_LDL_HDL_AND_TG_INCLUDING_DISEASES | 0.138244 | 0.003852 | 2.886637 | 0.005261 | 0.16483 | -2.20896 |
| ANDERSEN_CHOLANGIOCARCINOMA_CLASS2 | 0.112275 | -0.10265 | 2.884768 | 0.005289 | 0.16483 | -2.21338 |
| BROWNE_HCMV_INFECTION_6HR_UP | 0.091288 | -0.15712 | 2.883384 | 0.00531 | 0.16483 | -2.21665 |
| SAGIV_CD24_TARGETS_UP | -0.18299 | -0.05216 | -2.87736 | 0.0054 | 0.166832 | -2.23087 |
| WP_GLUCOCORTICOID_RECEPTOR_PATHWAY | 0.140131 | -0.11163 | 2.867937 | 0.005545 | 0.170393 | -2.25308 |
| REACTOME_MOLYBDENUM_COFACTOR_BIOSYNTHESIS | -0.27581 | 0.010603 | -2.86447 | 0.005599 | 0.170393 | -2.26124 |
| LIM_MAMMARY_LUMINAL_PROGENITOR_UP | 0.107803 | -0.03276 | 2.863888 | 0.005608 | 0.170393 | -2.2626 |
| TERAMOTO_OPN_TARGETS_CLUSTER_4 | 0.155232 | -0.07769 | 2.862977 | 0.005623 | 0.170393 | -2.26474 |
| REACTOME_RNA_POLYMERASE_III_CHAIN_ELONGATION | -0.23265 | -0.01806 | -2.85599 | 0.005734 | 0.172933 | -2.28114 |
| MIKKELSEN_MCV6_LCP_WITH_H3K4ME3 | 0.074042 | -0.21589 | 2.854176 | 0.005763 | 0.172994 | -2.2854 |
| DAZARD_UV_RESPONSE_CLUSTER_G4 | 0.198995 | -0.11462 | 2.849815 | 0.005834 | 0.173183 | -2.29562 |
| HUNSBERGER_EXERCISE_REGULATED_GENES | 0.153886 | -0.07428 | 2.849487 | 0.005839 | 0.173183 | -2.29639 |
| REACTOME_RECYCLING_OF_EIF2_GDP | -0.30825 | -0.01169 | -2.84876 | 0.005851 | 0.173183 | -2.2981 |
| SUZUKI_RESPONSE_TO_TSA | -0.16506 | 0.00521 | -2.84579 | 0.0059 | 0.173317 | -2.30505 |
| MYLLYKANGAS_AMPLIFICATION_HOT_SPOT_1 | 0.213429 | 0.0123 | 2.845164 | 0.00591 | 0.173317 | -2.30651 |
| REACTOME_ACTIVATION_OF_CASPASES_THROUGH_APOPTOSOME_MEDIATED_CLEAVAGE | -0.24923 | 0.009476 | -2.84144 | 0.005971 | 0.174323 | -2.31521 |
| REACTOME_PLASMA_LIPOPROTEIN_ASSEMBLY_REMODELING_AND_CLEARANCE | 0.11531 | -0.02333 | 2.838572 | 0.006019 | 0.17492 | -2.32191 |
| WP_NRF2_PATHWAY | 0.080697 | -0.16737 | 2.834982 | 0.00608 | 0.175399 | -2.33029 |
| SATO_SILENCED_BY_DEACETYLATION_IN_PANCREATIC_CANCER | 0.119402 | -0.09304 | 2.832335 | 0.006125 | 0.175399 | -2.33646 |
| SMID_BREAST_CANCER_BASAL_UP | 0.070383 | -0.11445 | 2.831641 | 0.006137 | 0.175399 | -2.33808 |
| WP_FAMILIAL_HYPERLIPIDEMIA_TYPE_1 | 0.173527 | -0.00419 | 2.828857 | 0.006184 | 0.175399 | -2.34457 |
| PEDERSEN_METASTASIS_BY_ERBB2_ISOFORM_3 | 0.153384 | 0.000518 | 2.828014 | 0.006199 | 0.175399 | -2.34653 |
| TIAN_TNF_SIGNALING_VIA_NFKB | 0.244401 | -0.00012 | 2.827878 | 0.006201 | 0.175399 | -2.34684 |
| BIOCARTA_BLYMPHOCYTE_PATHWAY | 0.088306 | -0.54408 | 2.819985 | 0.006339 | 0.177709 | -2.3652 |
| BOYLAN_MULTIPLE_MYELOMA_C_UP | -0.13281 | -0.04087 | -2.81854 | 0.006364 | 0.177709 | -2.36856 |
| REACTOME_RNA_POLYMERASE_I_PROMOTER_ESCAPE | -0.13695 | -0.25993 | -2.8184 | 0.006367 | 0.177709 | -2.36889 |
| WP_VITAMIN_B12_METABOLISM | 0.104886 | -0.16585 | 2.81287 | 0.006465 | 0.179566 | -2.38172 |
| REACTOME_RNA_POLYMERASE_I_TRANSCRIPTION_TERMINATION | -0.1357 | -0.24632 | -2.81124 | 0.006494 | 0.179566 | -2.38549 |
| REACTOME_CILIUM_ASSEMBLY | -0.11527 | -0.09113 | -2.80993 | 0.006518 | 0.179566 | -2.38853 |
| REACTOME_INTERLEUKIN_4_AND_INTERLEUKIN_13_SIGNALING | 0.13144 | -0.06358 | 2.806563 | 0.006579 | 0.180465 | -2.39633 |
| DIRMEIER_LMP1_RESPONSE_EARLY | 0.128444 | -0.32578 | 2.801087 | 0.006679 | 0.18168 | -2.409 |
| WORSCHECH_TUMOR_EVASION_AND_TOLEROGENICITY_DN | 0.173862 | -0.06334 | 2.799813 | 0.006703 | 0.18168 | -2.41194 |
| JAATINEN_HEMATOPOIETIC_STEM_CELL_DN | 0.13123 | -0.07473 | 2.799489 | 0.006709 | 0.18168 | -2.41269 |
| LEE_LIVER_CANCER_MYC_E2F1_DN | 0.091839 | -0.07918 | 2.795995 | 0.006774 | 0.182226 | -2.42076 |
| WP_MAMMARY_GLAND_DEVELOPMENT_PATHWAY_PUBERTY_STAGE_2_OF_4 | 0.233191 | -0.02372 | 2.795334 | 0.006786 | 0.182226 | -2.42228 |
| WP_LET7_INHIBITION_OF_ES_CELL_REPROGRAMMING | 0.201548 | -0.4471 | 2.790556 | 0.006876 | 0.183868 | -2.4333 |
| REACTOME_SULFUR_AMINO_ACID_METABOLISM | -0.19463 | -0.01323 | -2.77656 | 0.007146 | 0.190014 | -2.46548 |
| WP_MIRNA_BIOGENESIS | -0.29883 | -0.00514 | -2.77493 | 0.007178 | 0.190014 | -2.46923 |
| ODONNELL_TFRC_TARGETS_UP | 0.062879 | -0.16997 | 2.774054 | 0.007196 | 0.190014 | -2.47124 |
| HAN_JNK_SINGALING_DN | 0.143501 | -0.05107 | 2.771387 | 0.007249 | 0.190621 | -2.47736 |
| BLANCO_MELO_COVID19_SARS_COV_2_LOW_MOI_INFECTION_A594_ACE2_EXPRESSING_CELLS_UP | 0.094681 | -0.20808 | 2.76736 | 0.007329 | 0.190698 | -2.48659 |
| LEI_HOXC8_TARGETS_UP | 0.165889 | -0.07518 | 2.766627 | 0.007344 | 0.190698 | -2.48827 |
| BIOCARTA_EPONFKB_PATHWAY | 0.203019 | -0.01901 | 2.765648 | 0.007364 | 0.190698 | -2.49051 |
| NAKAJIMA_MAST_CELL | 0.156475 | -0.0895 | 2.765262 | 0.007371 | 0.190698 | -2.49139 |
| CHIBA_RESPONSE_TO_TSA_UP | 0.109992 | -0.22325 | 2.757036 | 0.007539 | 0.193879 | -2.51019 |
| WANG_METHYLATED_IN_BREAST_CANCER | 0.162995 | -0.20424 | 2.756265 | 0.007555 | 0.193879 | -2.51195 |
| KIM_RESPONSE_TO_TSA_AND_DECITABINE_DN | -0.16487 | 0.002069 | -2.75361 | 0.00761 | 0.194507 | -2.51801 |
| REACTOME_ERYTHROCYTES_TAKE_UP_CARBON_DIOXIDE_AND_RELEASE_OXYGEN | 0.184917 | 0.008656 | 2.750054 | 0.007685 | 0.195081 | -2.52612 |
| REACTOME_INTESTINAL_ABSORPTION | 0.23644 | 0.020332 | 2.749605 | 0.007694 | 0.195081 | -2.52714 |
| DELPUECH_FOXO3_TARGETS_UP | 0.123738 | -0.09792 | 2.744606 | 0.0078 | 0.196977 | -2.53852 |
| DASU_IL6_SIGNALING_UP | 0.104687 | -0.29262 | 2.738547 | 0.00793 | 0.197572 | -2.5523 |
| GARGALOVIC_RESPONSE_TO_OXIDIZED_PHOSPHOLIPIDS_GREEN_DN | -0.14402 | -0.19408 | -2.73852 | 0.00793 | 0.197572 | -2.55236 |
| MAHAJAN_RESPONSE_TO_IL1A_UP | 0.110671 | -0.05138 | 2.737665 | 0.007949 | 0.197572 | -2.5543 |
| REACTOME_PHOSPHATE_BOND_HYDROLYSIS_BY_NUDT_PROTEINS | -0.29794 | -0.0182 | -2.73657 | 0.007973 | 0.197572 | -2.55679 |
| KHETCHOUMIAN_TRIM24_TARGETS_UP | 0.171924 | -0.00486 | 2.734989 | 0.008007 | 0.197572 | -2.56038 |
| REACTOME_UBIQUINOL_BIOSYNTHESIS | -0.25905 | -0.01463 | -2.73326 | 0.008045 | 0.197572 | -2.5643 |
| BIOCARTA_IL17_PATHWAY | 0.194044 | -0.0174 | 2.728044 | 0.00816 | 0.197572 | -2.57612 |
| XU_AKT1_TARGETS_48HR | -0.1673 | -0.09549 | -2.72781 | 0.008165 | 0.197572 | -2.57665 |
| OISHI_CHOLANGIOMA_STEM_CELL_LIKE_UP | -0.13084 | -0.13515 | -2.72725 | 0.008177 | 0.197572 | -2.57792 |
| REACTOME_DEGRADATION_OF_THE_EXTRACELLULAR_MATRIX | 0.124326 | -0.06237 | 2.726453 | 0.008195 | 0.197572 | -2.57972 |
| IYENGAR_RESPONSE_TO_ADIPOCYTE_FACTORS | 0.185282 | -0.09973 | 2.725182 | 0.008223 | 0.197572 | -2.5826 |
| WP_OXIDATION_BY_CYTOCHROME_P450 | 0.114228 | -0.08719 | 2.724964 | 0.008228 | 0.197572 | -2.58309 |
| RASHI_RESPONSE_TO_IONIZING_RADIATION_2 | 0.123387 | -0.10456 | 2.723506 | 0.008261 | 0.197572 | -2.58639 |
| BILD_HRAS_ONCOGENIC_SIGNATURE | 0.118739 | -0.05632 | 2.722947 | 0.008273 | 0.197572 | -2.58765 |
| REACTOME_HDR_THROUGH_SINGLE_STRAND_ANNEALING_SSA | -0.14577 | -0.05692 | -2.72218 | 0.008291 | 0.197572 | -2.58939 |
| DELYS_THYROID_CANCER_UP | 0.103635 | -0.11142 | 2.720875 | 0.00832 | 0.197572 | -2.59234 |
| REACTOME_PTK6_REGULATES_RTKS_AND_THEIR_EFFECTORS_AKT1_AND_DOK1 | 0.218725 | 0.006954 | 2.71657 | 0.008418 | 0.198791 | -2.60206 |
| ZHOU_INFLAMMATORY_RESPONSE_LIVE_UP | 0.081205 | -0.15939 | 2.715867 | 0.008434 | 0.198791 | -2.60365 |
| PARK_APL_PATHOGENESIS_DN | 0.128573 | -0.2184 | 2.714054 | 0.008475 | 0.199033 | -2.60774 |
| REACTOME_MITOCHONDRIAL_BIOGENESIS | -0.14034 | -0.03547 | -2.70927 | 0.008586 | 0.200884 | -2.61851 |
| CROMER_TUMORIGENESIS_UP | 0.161992 | -0.09756 | 2.704703 | 0.008693 | 0.20205 | -2.6288 |
| SMID_BREAST_CANCER_NORMAL_LIKE_DN | 0.256652 | 0.001315 | 2.704426 | 0.008699 | 0.20205 | -2.62942 |
| MIKKELSEN_IPS_LCP_WITH_H3K4ME3 | 0.077477 | -0.20642 | 2.700952 | 0.008781 | 0.202151 | -2.63723 |
| CROMER_TUMORIGENESIS_DN | 0.100814 | -0.22547 | 2.700551 | 0.008791 | 0.202151 | -2.63813 |
| REACTOME_DAG_AND_IP3_SIGNALING | -0.11736 | -0.05946 | -2.70021 | 0.008799 | 0.202151 | -2.6389 |
| WP_COMPLEMENT_SYSTEM | 0.082282 | -0.17008 | 2.694743 | 0.00893 | 0.204419 | -2.65117 |
| REACTOME_PLATELET_ADHESION_TO_EXPOSED_COLLAGEN | 0.139821 | -0.35625 | 2.692854 | 0.008975 | 0.204575 | -2.65541 |
| CERVERA_SDHB_TARGETS_1_UP | 0.097987 | -0.04743 | 2.690783 | 0.009026 | 0.204575 | -2.66005 |
| STARK_HYPPOCAMPUS_22Q11_DELETION_DN | -0.15175 | -0.01359 | -2.69048 | 0.009033 | 0.204575 | -2.66073 |
| LIU_SMARCA4_TARGETS | 0.145203 | -0.05494 | 2.688143 | 0.00909 | 0.205136 | -2.66596 |
| SALVADOR_MARTIN_PEDIATRIC_TBD_ANTI_TNF_THERAPY_NONRESPONDER_POST_TREATMENT_UP | 0.120231 | -0.34986 | 2.684044 | 0.009191 | 0.206314 | -2.67513 |
| WP_MITOCHONDRIAL_COMPLEX_II_ASSEMBLY | -0.28432 | -0.13697 | -2.68339 | 0.009207 | 0.206314 | -2.6766 |
| SANA_TNF_SIGNALING_UP | 0.121043 | -0.28172 | 2.679888 | 0.009294 | 0.207112 | -2.68442 |
| WP_PROSTAGLANDIN_SIGNALING | 0.116669 | -0.2256 | 2.679345 | 0.009308 | 0.207112 | -2.68563 |
| AMIT_EGF_RESPONSE_240_MCF10A | 0.19611 | -0.01043 | 2.676295 | 0.009384 | 0.207506 | -2.69244 |
| WP_SARSCOV2_AND_COVID19_PATHWAY | 0.175994 | -0.01798 | 2.676043 | 0.009391 | 0.207506 | -2.693 |
| HUMMERICH_MALIGNANT_SKIN_TUMOR_UP | 0.170768 | 0.011021 | 2.674034 | 0.009441 | 0.207906 | -2.69748 |
| REACTOME_BASIGIN_INTERACTIONS | 0.115821 | -0.03911 | 2.67069 | 0.009526 | 0.208954 | -2.70493 |
| PLASARI_TGFB1_TARGETS_10HR_UP | 0.113754 | -0.06547 | 2.66959 | 0.009555 | 0.208954 | -2.70738 |
| REACTOME_MITOCHONDRIAL_UNCOUPLING | 0.233455 | -0.01692 | 2.667335 | 0.009613 | 0.209501 | -2.7124 |
| BERGER_MBD2_TARGETS | 0.204348 | -0.00754 | 2.664514 | 0.009685 | 0.209835 | -2.71868 |
| KORKOLA_CORRELATED_WITH_POU5F1 | 0.081617 | -0.33073 | 2.663274 | 0.009718 | 0.209835 | -2.72143 |
| REACTOME_RNA_POLYMERASE_III_TRANSCRIPTION | -0.18505 | -0.03645 | -2.66256 | 0.009736 | 0.209835 | -2.72301 |
| MIKKELSEN_ES_LCP_WITH_H3K4ME3 | 0.066923 | -0.24709 | 2.66166 | 0.00976 | 0.209835 | -2.72502 |
| PID_INTEGRIN_A9B1_PATHWAY | 0.180852 | -0.08772 | 2.658768 | 0.009836 | 0.210674 | -2.73144 |
| REACTOME_RNA_POLYMERASE_I_TRANSCRIPTION_INITIATION | -0.12161 | -0.26488 | -2.65765 | 0.009865 | 0.210674 | -2.73392 |
| EHLERS_ANEUPLOIDY_UP | -0.20807 | -0.08508 | -2.65282 | 0.009993 | 0.212496 | -2.74462 |
| GRAHAM_NORMAL_QUIESCENT_VS_NORMAL_DIVIDING_UP | 0.137241 | -0.06065 | 2.651925 | 0.010017 | 0.212496 | -2.74661 |
| NABA_MATRISOME_ASSOCIATED | 0.096036 | -0.12281 | 2.649347 | 0.010086 | 0.213252 | -2.75232 |
| REACTOME_KETONE_BODY_METABOLISM | -0.16247 | -0.08202 | -2.64366 | 0.01024 | 0.2145 | -2.7649 |
| LINDGREN_BLADDER_CANCER_CLUSTER_1_UP | -0.15804 | -0.05367 | -2.64352 | 0.010244 | 0.2145 | -2.7652 |
| SPIRA_SMOKERS_LUNG_CANCER_DN | -0.16569 | 0.015639 | -2.64344 | 0.010246 | 0.2145 | -2.76538 |
| WP_COVID19_ADVERSE_OUTCOME_PATHWAY | 0.123884 | -0.36357 | 2.637141 | 0.01042 | 0.216096 | -2.77928 |
| BIOCARTA_PEPI_PATHWAY | 0.177409 | -0.0964 | 2.636959 | 0.010425 | 0.216096 | -2.77968 |
| ZHENG_IL22_SIGNALING_UP | 0.097964 | -0.21301 | 2.635537 | 0.010464 | 0.216096 | -2.78282 |
| LEE_LIVER_CANCER_ACOX1_UP | 0.118151 | -0.06156 | 2.63475 | 0.010486 | 0.216096 | -2.78455 |
| KOHOUTEK_CCNT2_TARGETS | 0.075176 | -0.24812 | 2.633397 | 0.010524 | 0.216096 | -2.78753 |
| YIH_RESPONSE_TO_ARSENITE_C5 | 0.184809 | -0.0049 | 2.632307 | 0.010555 | 0.216096 | -2.78994 |
| REACTOME_G_ALPHA_I_SIGNALLING_EVENTS | 0.077172 | -0.1223 | 2.632101 | 0.01056 | 0.216096 | -2.79039 |
| WP_MICROGLIA_PATHOGEN_PHAGOCYTOSIS_PATHWAY | 0.213945 | -0.01856 | 2.628367 | 0.010666 | 0.21755 | -2.79861 |
| GROSS_HIF1A_TARGETS_UP | 0.190477 | -0.00686 | 2.625981 | 0.010733 | 0.218234 | -2.80385 |
| SCHLESINGER_METHYLATED_DE_NOVO_IN_CANCER | 0.120509 | -0.0652 | 2.623 | 0.010819 | 0.219266 | -2.8104 |
| BIOCARTA_ACTINY_PATHWAY | -0.19973 | -0.00016 | -2.62124 | 0.010869 | 0.219593 | -2.81427 |
| REACTOME_NR1H2_AND_NR1H3_MEDIATED_SIGNALING | 0.115536 | -0.12842 | 2.616028 | 0.01102 | 0.22194 | -2.82569 |
| REACTOME_CONVERSION_FROM_APC_C_CDC20_TO_APC_C_CDH1_IN_LATE_ANAPHASE | -0.1783 | -0.00915 | -2.61159 | 0.01115 | 0.223854 | -2.83541 |
| AMIT_EGF_RESPONSE_120_MCF10A | 0.176078 | -0.03291 | 2.605971 | 0.011317 | 0.226087 | -2.84769 |
| HUPER_BREAST_BASAL_VS_LUMINAL_UP | 0.113929 | -0.01056 | 2.60513 | 0.011342 | 0.226087 | -2.84953 |
| PID_DELTA_NP63_PATHWAY | 0.097793 | -0.02177 | 2.604169 | 0.011371 | 0.226087 | -2.85163 |
| REACTOME_BIOTIN_TRANSPORT_AND_METABOLISM | -0.18703 | -0.09546 | -2.60308 | 0.011404 | 0.226087 | -2.854 |
| NAKAMURA_METASTASIS_MODEL_DN | 0.127224 | -0.07551 | 2.599797 | 0.011503 | 0.227348 | -2.86117 |
| INAMURA_LUNG_CANCER_SCC_UP | 0.173111 | -0.00782 | 2.597393 | 0.011576 | 0.228085 | -2.86641 |
| WANG_BARRETTS_ESOPHAGUS_UP | 0.098328 | -0.09987 | 2.59408 | 0.011678 | 0.229374 | -2.87362 |
| WP_LTF_DANGER_SIGNAL_RESPONSE_PATHWAY | 0.117085 | -0.39739 | 2.591002 | 0.011773 | 0.23022 | -2.88032 |
| REACTOME_GLYOXYLATE_METABOLISM_AND_GLYCINE_DEGRADATION | -0.10932 | -0.0039 | -2.58932 | 0.011825 | 0.23022 | -2.88398 |
| WP_VITAMIN_B6DEPENDENT_AND_RESPONSIVE_DISORDERS | -0.27142 | -0.02128 | -2.58835 | 0.011855 | 0.23022 | -2.88608 |
| LEIN_CHOROID_PLEXUS_MARKERS | 0.076081 | -0.05572 | 2.587028 | 0.011896 | 0.23022 | -2.88895 |
| WP_DNA_REPAIR_PATHWAYS_FULL_NETWORK | -0.13589 | -0.09245 | -2.58686 | 0.011902 | 0.23022 | -2.88932 |
| REACTOME_ERYTHROCYTES_TAKE_UP_OXYGEN_AND_RELEASE_CARBON_DIOXIDE | 0.230817 | -0.01331 | 2.585524 | 0.011944 | 0.23033 | -2.89222 |
| NABA_MATRISOME | 0.092124 | -0.11462 | 2.583775 | 0.011999 | 0.230692 | -2.89601 |
| HOLLEMAN_DAUNORUBICIN_ALL_UP | 0.175637 | -0.00743 | 2.580831 | 0.012092 | 0.231781 | -2.9024 |
| REACTOME_ABERRANT_REGULATION_OF_MITOTIC_EXIT_IN_CANCER_DUE_TO_RB1_DEFECTS | -0.18462 | -0.00883 | -2.57867 | 0.01216 | 0.2324 | -2.90708 |
| PALOMERO_GSI_SENSITIVITY_DN | -0.3101 | 0.012347 | -2.57666 | 0.012225 | 0.232929 | -2.91143 |
| BIOCARTA_ASBCELL_PATHWAY | 0.082063 | -0.51649 | 2.571833 | 0.01238 | 0.23459 | -2.92187 |
| WP_MITOCHONDRIAL_COMPLEX_IV_ASSEMBLY | -0.24012 | -0.0451 | -2.57053 | 0.012423 | 0.23459 | -2.92469 |
| WP_DEVELOPMENT_OF_PULMONARY_DENDRITIC_CELLS_AND_MACROPHAGE_SUBSETS | 0.204606 | -0.08346 | 2.570434 | 0.012426 | 0.23459 | -2.9249 |
| BYSTRYKH_SCP2_QTL | -0.16605 | -0.26733 | -2.5694 | 0.012459 | 0.23459 | -2.92713 |
| FUJIWARA_PARK2_IN_LIVER_CANCER_UP | 0.192435 | -0.00734 | 2.567558 | 0.01252 | 0.23503 | -2.93111 |
| GAZDA_DIAMOND_BLACKFAN_ANEMIA_MYELOID_DN | -0.17282 | -0.11112 | -2.56542 | 0.01259 | 0.235651 | -2.93572 |
| NAKAJIMA_EOSINOPHIL | 0.096229 | -0.19121 | 2.563037 | 0.012669 | 0.235784 | -2.94086 |
| PID_LPA4_PATHWAY | -0.14983 | -0.11067 | -2.56296 | 0.012671 | 0.235784 | -2.94102 |
| BIOCARTA_RECK_PATHWAY | 0.208424 | -0.09463 | 2.55961 | 0.012782 | 0.237165 | -2.94824 |
| NAGASHIMA_NRG1_SIGNALING_UP | 0.166216 | -0.08316 | 2.557642 | 0.012848 | 0.237693 | -2.95248 |
| MARTIN_VIRAL_GPCR_SIGNALING_UP | 0.096587 | -0.01208 | 2.555197 | 0.012931 | 0.238326 | -2.95774 |
| ONGUSAHA_BRCA1_TARGETS_UP | 0.208534 | -0.09135 | 2.5544 | 0.012957 | 0.238326 | -2.95945 |
| REACTOME_DAP12_INTERACTIONS | 0.085402 | -0.37387 | 2.552642 | 0.013017 | 0.238731 | -2.96323 |
| REACTOME_AMINO_ACID_TRANSPORT_ACROSS_THE_PLASMA_MEMBRANE | 0.116161 | -0.02976 | 2.546037 | 0.013243 | 0.242176 | -2.97741 |
| REACTOME_HDL_CLEARANCE | 0.234088 | -0.0158 | 2.54342 | 0.013333 | 0.243131 | -2.98302 |
| MOREIRA_RESPONSE_TO_TSA_DN | 0.138864 | -0.00465 | 2.540388 | 0.013439 | 0.243625 | -2.98951 |
| SHANK_TAL1_TARGETS_DN | 0.185364 | 0.006308 | 2.539843 | 0.013458 | 0.243625 | -2.99067 |
| DELACROIX_RAR_TARGETS_UP | 0.129783 | -0.09408 | 2.538309 | 0.013512 | 0.243625 | -2.99396 |
| KEGG_REGULATION_OF_AUTOPHAGY | -0.08917 | 0.002119 | -2.53766 | 0.013534 | 0.243625 | -2.99535 |
| SUMI_HNF4A_TARGETS | 0.173896 | -0.02132 | 2.536234 | 0.013585 | 0.243625 | -2.99839 |
| WP_MAMMARY_GLAND_DEVELOPMENT_PATHWAY_INVOLUTION_STAGE_4_OF_4 | 0.207998 | -0.02818 | 2.536078 | 0.01359 | 0.243625 | -2.99873 |
| RICKMAN_METASTASIS_DN | 0.096713 | -0.1019 | 2.533334 | 0.013687 | 0.244677 | -3.00459 |
| WP_LIVER_X_RECEPTOR_PATHWAY | 0.159596 | -0.08798 | 2.527215 | 0.013906 | 0.247892 | -3.01764 |
| TAKEDA_TARGETS_OF_NUP98_HOXA9_FUSION_3D_DN | 0.136348 | -0.03443 | 2.526056 | 0.013948 | 0.247943 | -3.02011 |
| BIOCARTA_BARD1_PATHWAY | -0.19208 | 0.010293 | -2.52345 | 0.014042 | 0.248923 | -3.02565 |
| RAY_ALZHEIMERS_DISEASE | 0.098768 | -0.45538 | 2.520808 | 0.014138 | 0.249817 | -3.03128 |
| WP_RIBOFLAVIN_AND_COQ_DISORDERS | -0.1966 | -0.05425 | -2.51992 | 0.014171 | 0.249817 | -3.03317 |
| WP_TRANSCRIPTIONAL_CASCADE_REGULATING_ADIPOGENESIS | 0.206413 | -0.01213 | 2.517753 | 0.014251 | 0.250526 | -3.03778 |
| NAKAYAMA_SOFT_TISSUE_TUMORS_PCA1_UP | 0.105667 | -0.37876 | 2.515619 | 0.014329 | 0.250931 | -3.04231 |
| REACTOME_SULFIDE_OXIDATION_TO_SULFATE | -0.23442 | -0.02097 | -2.51499 | 0.014352 | 0.250931 | -3.04364 |
| PID_VEGF_VEGFR_PATHWAY | 0.18545 | -0.02229 | 2.512158 | 0.014458 | 0.251566 | -3.04966 |
| PEPPER_CHRONIC_LYMPHOCYTIC_LEUKEMIA_UP | 0.097292 | -0.51841 | 2.511642 | 0.014477 | 0.251566 | -3.05075 |
| REACTOME_ALPHA_OXIDATION_OF_PHYTANATE | -0.27731 | -0.01477 | -2.51084 | 0.014507 | 0.251566 | -3.05246 |
| GAZDA_DIAMOND_BLACKFAN_ANEMIA_PROGENITOR_DN | -0.1805 | -0.05701 | -2.50973 | 0.014549 | 0.251598 | -3.0548 |
| BOSCO_EPITHELIAL_DIFFERENTIATION_MODULE | 0.11705 | -0.21723 | 2.506932 | 0.014654 | 0.252582 | -3.06073 |
| REACTOME_DISEASES_ASSOCIATED_WITH_SURFACTANT_METABOLISM | 0.223132 | 0.019077 | 2.506113 | 0.014685 | 0.252582 | -3.06247 |
| KEGG_JAK_STAT_SIGNALING_PATHWAY | 0.076398 | -0.0246 | 2.50123 | 0.014871 | 0.254467 | -3.07279 |
| OHGUCHI_LIVER_HNF4A_TARGETS_DN | 0.100699 | -0.11398 | 2.501131 | 0.014875 | 0.254467 | -3.073 |
| MANN_RESPONSE_TO_AMIFOSTINE_DN | -0.22429 | 0.008058 | -2.49691 | 0.015037 | 0.256556 | -3.08192 |
| BIOCARTA_GRANULOCYTES_PATHWAY | 0.116497 | -0.30342 | 2.492689 | 0.015201 | 0.258659 | -3.09082 |
| REACTOME_HOMOLOGOUS_DNA_PAIRING_AND_STRAND_EXCHANGE | -0.13618 | -0.05425 | -2.49017 | 0.015299 | 0.259639 | -3.09612 |
| WP_TUMOR_SUPPRESSOR_ACTIVITY_OF_SMARCB1 | -0.1429 | -0.0313 | -2.48803 | 0.015384 | 0.260375 | -3.10063 |
| WP_COVID19_THROMBOSIS_AND_ANTICOAGULATION | 0.230012 | 0.004712 | 2.485026 | 0.015503 | 0.261301 | -3.10695 |
| NUMATA_CSF3_SIGNALING_VIA_STAT3 | 0.126608 | 0.003389 | 2.484579 | 0.01552 | 0.261301 | -3.10789 |
| KEGG_HEMATOPOIETIC_CELL_LINEAGE | 0.097108 | -0.21988 | 2.481863 | 0.015629 | 0.26243 | -3.1136 |
| REACTOME_VXPX_CARGO_TARGETING_TO_CILIUM | -0.16561 | -0.04911 | -2.48067 | 0.015677 | 0.26254 | -3.1161 |
| SMIRNOV_CIRCULATING_ENDOTHELIOCYTES_IN_CANCER_DN | 0.239946 | 0.003018 | 2.478399 | 0.015768 | 0.263309 | -3.12087 |
| BARIS_THYROID_CANCER_UP | -0.19646 | -0.01273 | -2.47666 | 0.015838 | 0.263309 | -3.12451 |
| REACTOME_P75NTR_RECRUITS_SIGNALLING_COMPLEXES | 0.189925 | -0.0833 | 2.474988 | 0.015906 | 0.263309 | -3.12802 |
| RODRIGUES_NTN1_TARGETS_DN | 0.062837 | -0.14346 | 2.474801 | 0.015914 | 0.263309 | -3.12841 |
| REACTOME_MITOCHONDRIAL_TRANSLATION | -0.21445 | -0.10317 | -2.47355 | 0.015965 | 0.263309 | -3.13104 |
| REACTOME_GENE_SILENCING_BY_RNA | -0.15734 | -0.03634 | -2.47313 | 0.015982 | 0.263309 | -3.13191 |
| REACTOME_PLASMA_LIPOPROTEIN_REMODELING | 0.126815 | 0.005644 | 2.472388 | 0.016012 | 0.263309 | -3.13347 |
| GARGALOVIC_RESPONSE_TO_OXIDIZED_PHOSPHOLIPIDS_MAGENTA_DN | -0.22391 | 0.00432 | -2.4699 | 0.016114 | 0.264306 | -3.13868 |
| JAZAG_TGFB1_SIGNALING_VIA_SMAD4_UP | 0.071741 | -0.13313 | 2.465325 | 0.016303 | 0.265052 | -3.14824 |
| KEGG_VASCULAR_SMOOTH_MUSCLE_CONTRACTION | -0.08878 | -0.04347 | -2.46486 | 0.016323 | 0.265052 | -3.14921 |
| ACEVEDO_LIVER_CANCER_WITH_H3K9ME3_UP | 0.082948 | -0.21281 | 2.464577 | 0.016334 | 0.265052 | -3.1498 |
| TAYLOR_METHYLATED_IN_ACUTE_LYMPHOBLASTIC_LEUKEMIA | -0.10393 | -0.12024 | -2.46403 | 0.016357 | 0.265052 | -3.15093 |
| NOJIMA_SFRP2_TARGETS_UP | 0.131776 | -0.20711 | 2.461761 | 0.016452 | 0.265052 | -3.15568 |
| REACTOME_MATURATION_OF_SARS_COV_1_SPIKE_PROTEIN | 0.210738 | -0.16968 | 2.460577 | 0.016502 | 0.265052 | -3.15814 |
| LENAOUR_DENDRITIC_CELL_MATURATION_UP | 0.105123 | -0.15947 | 2.458998 | 0.016568 | 0.265052 | -3.16144 |
| REACTOME_DEFECTIVE_F9_ACTIVATION | 0.253574 | 0.017465 | 2.458187 | 0.016602 | 0.265052 | -3.16313 |
| REACTOME_CYTOCHROME_C_MEDIATED_APOPTOTIC_RESPONSE | -0.23074 | -0.01804 | -2.4556 | 0.016712 | 0.265052 | -3.16852 |
| REACTOME_HATS_ACETYLATE_HISTONES | -0.16793 | -0.05341 | -2.45542 | 0.01672 | 0.265052 | -3.16889 |
| TURJANSKI_MAPK7_TARGETS | 0.23777 | -0.00925 | 2.455389 | 0.016721 | 0.265052 | -3.16896 |
| REACTOME_LEISHMANIA_INFECTION | 0.056869 | -0.04516 | 2.454524 | 0.016758 | 0.265052 | -3.17076 |
| WP_NAD_BIOSYNTHESIS_II_FROM_TRYPTOPHAN | 0.173651 | -0.11065 | 2.454287 | 0.016768 | 0.265052 | -3.17125 |
| WP_NUCLEOTIDEBINDING_OLIGOMERIZATION_DOMAIN_NOD_PATHWAY | 0.079491 | -0.3441 | 2.452589 | 0.01684 | 0.265052 | -3.17478 |
| HUMMERICH_BENIGN_SKIN_TUMOR_UP | 0.145983 | 0.007183 | 2.451994 | 0.016866 | 0.265052 | -3.17602 |
| WP_CELLULAR_PROTEOSTASIS | -0.18795 | -0.35469 | -2.4516 | 0.016883 | 0.265052 | -3.17684 |
| BIERIE_INFLAMMATORY_RESPONSE_TGFB1 | 0.296474 | 0.003314 | 2.451082 | 0.016905 | 0.265052 | -3.17792 |
| MEISSNER_BRAIN_HCP_WITH_H3K4ME3_AND_H3K27ME3 | 0.078869 | -0.05515 | 2.448236 | 0.017028 | 0.265052 | -3.18383 |
| SHIN_B_CELL_LYMPHOMA_CLUSTER_8 | 0.140238 | -0.03782 | 2.448213 | 0.017029 | 0.265052 | -3.18388 |
| MISHRA_CARCINOMA_ASSOCIATED_FIBROBLAST_DN | 0.127224 | -0.14658 | 2.448154 | 0.017031 | 0.265052 | -3.184 |
| KEGG_GLYCOSAMINOGLYCAN_DEGRADATION | 0.142766 | -0.00498 | 2.447511 | 0.017059 | 0.265052 | -3.18534 |
| REACTOME_SENSORY_PROCESSING_OF_SOUND | 0.107626 | 0.013453 | 2.44712 | 0.017076 | 0.265052 | -3.18615 |
| REACTOME_SIGNALING_BY_TYPE_1_INSULIN_LIKE_GROWTH_FACTOR_1_RECEPTOR_IGF1R | -0.09524 | -0.00723 | -2.44574 | 0.017136 | 0.265322 | -3.18902 |
| STARK_BRAIN_22Q11_DELETION | 0.165667 | -0.08582 | 2.4448 | 0.017177 | 0.265322 | -3.19096 |
| PID_ATR_PATHWAY | -0.13611 | -0.02859 | -2.44311 | 0.01725 | 0.265814 | -3.19446 |
| XIE_ST_HSC_S1PR3_OE_DN | 0.156597 | 0.00644 | 2.440653 | 0.017358 | 0.26683 | -3.19956 |
| TESAR_ALK_AND_JAK_TARGETS_MOUSE_ES_D4_UP | 0.262534 | -0.01274 | 2.436965 | 0.017521 | 0.268418 | -3.20719 |
| BIOCARTA_MSP_PATHWAY | 0.128944 | -0.51498 | 2.436121 | 0.017559 | 0.268418 | -3.20894 |
| SUH_COEXPRESSED_WITH_ID1_AND_ID2_UP | 0.203873 | -0.10257 | 2.435001 | 0.017608 | 0.268418 | -3.21126 |
| JOHNSTONE_PARVB_TARGETS_2_UP | 0.101648 | -0.19728 | 2.434511 | 0.01763 | 0.268418 | -3.21227 |
| TERAMOTO_OPN_TARGETS_CLUSTER_8 | 0.150854 | -0.22771 | 2.432388 | 0.017725 | 0.269218 | -3.21666 |
| REACTOME_HEMOSTASIS | 0.063588 | -0.05622 | 2.430143 | 0.017826 | 0.270024 | -3.22129 |
| KAMIKUBO_MYELOID_CEBPA_NETWORK | 0.168358 | -0.04781 | 2.429321 | 0.017863 | 0.270024 | -3.22299 |
| DURCHDEWALD_SKIN_CARCINOGENESIS_UP | -0.09096 | -0.04466 | -2.42687 | 0.017974 | 0.271053 | -3.22804 |
| LEE_LIVER_CANCER_MYC_DN | 0.086264 | -0.09007 | 2.423212 | 0.01814 | 0.271726 | -3.23559 |
| REACTOME_SYNTHESIS_OF_BILE_ACIDS_AND_BILE_SALTS_VIA_24_HYDROXYCHOLESTEROL | 0.142601 | 0.000682 | 2.41943 | 0.018314 | 0.271726 | -3.24337 |
| YOSHIMURA_MAPK8_TARGETS_UP | 0.060997 | -0.14048 | 2.41861 | 0.018352 | 0.271726 | -3.24506 |
| VANDESLUIS_COMMD1_TARGETS_GROUP_4_UP | 0.126614 | -0.25564 | 2.41665 | 0.018443 | 0.271726 | -3.24909 |
| REACTOME_CLASS_A_1_RHODOPSIN_LIKE_RECEPTORS | 0.121724 | -0.04127 | 2.415994 | 0.018473 | 0.271726 | -3.25043 |
| REACTOME_COLLAGEN_DEGRADATION | 0.1358 | -0.11255 | 2.415707 | 0.018487 | 0.271726 | -3.25102 |
| WP_TRIACYLGLYCERIDE_SYNTHESIS | 0.109968 | -0.2629 | 2.41207 | 0.018657 | 0.271726 | -3.25849 |
| WP_CIRCADIAN_RHYTHM_GENES | 0.06735 | -0.0437 | 2.411647 | 0.018676 | 0.271726 | -3.25936 |
| FREDERICK_PRKCI_TARGETS | 0.144579 | -0.37653 | 2.411172 | 0.018699 | 0.271726 | -3.26033 |
| REACTOME_HDR_THROUGH_MMEJ_ALT_NHEJ | -0.20249 | -0.01039 | -2.4108 | 0.018716 | 0.271726 | -3.26109 |
| REACTOME_ALTERNATIVE_COMPLEMENT_ACTIVATION | 0.117434 | -0.53527 | 2.410241 | 0.018743 | 0.271726 | -3.26224 |
| BRUINS_UVC_RESPONSE_EARLY_LATE | -0.10366 | -0.12386 | -2.41007 | 0.01875 | 0.271726 | -3.26258 |
| LI_INDUCED_T_TO_NATURAL_KILLER_UP | 0.090962 | -0.19154 | 2.409502 | 0.018777 | 0.271726 | -3.26376 |
| KUUSELO_PANCREATIC_CANCER_19Q13_AMPLIFICATION | 0.087853 | -0.19227 | 2.409016 | 0.0188 | 0.271726 | -3.26475 |
| WENDT_COHESIN_TARGETS_UP | -0.19066 | -0.0142 | -2.40887 | 0.018807 | 0.271726 | -3.26504 |
| RICKMAN_HEAD_AND_NECK_CANCER_E | 0.13545 | -0.1006 | 2.407584 | 0.018868 | 0.271726 | -3.26769 |
| LIU_IL13_MEMORY_MODEL_DN | 0.180707 | 0.007572 | 2.407335 | 0.01888 | 0.271726 | -3.2682 |
| WP_MACROPHAGE_MARKERS | 0.266054 | 0.015586 | 2.406559 | 0.018917 | 0.271726 | -3.26979 |
| WP_CELL_DIFFERENTIATION_EXPANDED_INDEX | 0.15262 | -0.00345 | 2.403731 | 0.019052 | 0.271726 | -3.27558 |
| WP_MELATONIN_METABOLISM_AND_EFFECTS | 0.091861 | -0.11098 | 2.403056 | 0.019084 | 0.271726 | -3.27696 |
| WP_EUKARYOTIC_TRANSCRIPTION_INITIATION | -0.15414 | -0.20402 | -2.40297 | 0.019088 | 0.271726 | -3.27714 |
| REACTOME_PHENYLALANINE_AND_TYROSINE_METABOLISM | 0.136269 | -0.0026 | 2.402387 | 0.019116 | 0.271726 | -3.27833 |
| REACTOME_RNA_POLYMERASE_I_TRANSCRIPTION | -0.11011 | -0.2507 | -2.402 | 0.019135 | 0.271726 | -3.27912 |
| REACTOME_PROTEIN_METHYLATION | -0.21457 | -0.00311 | -2.40189 | 0.01914 | 0.271726 | -3.27935 |
| SABATES_COLORECTAL_ADENOMA_UP | 0.096923 | -0.07012 | 2.400432 | 0.01921 | 0.271726 | -3.28232 |
| HOFFMAN_CLOCK_TARGETS_DN | 0.154833 | -0.1406 | 2.399973 | 0.019232 | 0.271726 | -3.28326 |
| LI_PROSTATE_CANCER_EPIGENETIC | 0.109254 | -0.00228 | 2.399689 | 0.019246 | 0.271726 | -3.28384 |
| DORSEY_GAB2_TARGETS | 0.13683 | -0.06911 | 2.399467 | 0.019256 | 0.271726 | -3.28429 |
| REACTOME_HIV_TRANSCRIPTION_INITIATION | -0.14696 | -0.18248 | -2.3968 | 0.019386 | 0.271726 | -3.28975 |
| WP_MITOCHONDRIAL_COMPLEX_III_ASSEMBLY | -0.25779 | -0.00735 | -2.39651 | 0.019399 | 0.271726 | -3.29032 |
| FERRARI_RESPONSE_TO_FENRETINIDE_UP | 0.210621 | -0.0164 | 2.396116 | 0.019419 | 0.271726 | -3.29114 |
| LEIN_PONS_MARKERS | 0.079432 | -0.04857 | 2.395783 | 0.019435 | 0.271726 | -3.29181 |
| WAGNER_APO2_SENSITIVITY | 0.118496 | -0.06408 | 2.395038 | 0.019471 | 0.271726 | -3.29333 |
| REACTOME_MICRORNA_MIRNA_BIOGENESIS | -0.22976 | -0.02283 | -2.39489 | 0.019478 | 0.271726 | -3.29363 |
| VERNOCHET_ADIPOGENESIS | 0.148893 | -0.00109 | 2.394185 | 0.019513 | 0.271726 | -3.29507 |
| TIAN_TNF_SIGNALING_NOT_VIA_NFKB | 0.121785 | -0.34395 | 2.392938 | 0.019574 | 0.27198 | -3.29762 |
| REACTOME_NR1H2_NR1H3_REGULATE_GENE_EXPRESSION_TO_CONTROL_BILE_ACID_HOMEOSTASIS | 0.11977 | -0.33931 | 2.390147 | 0.019711 | 0.272616 | -3.3033 |
| STOSSI_RESPONSE_TO_ESTRADIOL | 0.094505 | -0.07248 | 2.388974 | 0.019769 | 0.272616 | -3.30569 |
| WP_PROXIMAL_TUBULE_TRANSPORT | 0.102099 | -0.04416 | 2.388938 | 0.01977 | 0.272616 | -3.30576 |
| WILENSKY_RESPONSE_TO_DARAPLADIB | 0.153035 | -0.2883 | 2.387271 | 0.019853 | 0.272616 | -3.30916 |
| VALK_AML_WITH_11Q23_REARRANGED | 0.109779 | -0.04361 | 2.386676 | 0.019882 | 0.272616 | -3.31037 |
| REACTOME_DEGRADATION_OF_CYSTEINE_AND_HOMOCYSTEINE | -0.17122 | -0.01388 | -2.38638 | 0.019897 | 0.272616 | -3.31098 |
| NAKAMURA_CANCER_MICROENVIRONMENT_UP | 0.112502 | -0.12082 | 2.385934 | 0.019919 | 0.272616 | -3.31187 |
| KUWANO_RNA_STABILIZED_BY_NO | 0.240575 | -0.14149 | 2.382951 | 0.020068 | 0.273483 | -3.31794 |
| REACTOME_TACHYKININ_RECEPTORS_BIND_TACHYKININS | 0.261595 | 0.046942 | 2.382942 | 0.020069 | 0.273483 | -3.31796 |
| SATO_SILENCED_BY_METHYLATION_IN_PANCREATIC_CANCER_2 | 0.09536 | -0.16925 | 2.38045 | 0.020194 | 0.27393 | -3.32302 |
| HUMMEL_BURKITTS_LYMPHOMA_DN | 0.216134 | -0.05554 | 2.379534 | 0.02024 | 0.27393 | -3.32487 |
| ABE_VEGFA_TARGETS_2HR | 0.11058 | -0.18529 | 2.37888 | 0.020273 | 0.27393 | -3.3262 |
| FURUKAWA_DUSP6_TARGETS_PCI35_UP | 0.08025 | -0.18909 | 2.377752 | 0.02033 | 0.27393 | -3.32849 |
| PASINI_SUZ12_TARGETS_UP | 0.063152 | -0.09905 | 2.377645 | 0.020335 | 0.27393 | -3.32871 |
| REACTOME_SYNTHESIS_OF_PC | -0.10804 | -0.2822 | -2.37717 | 0.02036 | 0.27393 | -3.32968 |
| WP_MATRIX_METALLOPROTEINASES | 0.083896 | -0.37413 | 2.375795 | 0.020429 | 0.27407 | -3.33246 |
| KEGG_SELENOAMINO_ACID_METABOLISM | -0.15431 | -0.0057 | -2.37496 | 0.020472 | 0.27407 | -3.33414 |
| YANG_BREAST_CANCER_ESR1_BULK_DN | 0.13435 | -0.07903 | 2.374423 | 0.020499 | 0.27407 | -3.33524 |
| GARCIA_TARGETS_OF_FLI1_AND_DAX1_DN | -0.09187 | -0.07114 | -2.37115 | 0.020667 | 0.275734 | -3.34187 |
| ZHOU_PANCREATIC_ENDOCRINE_PROGENITOR | 0.154779 | -0.03511 | 2.366265 | 0.020919 | 0.277184 | -3.35173 |
| MARIADASON_RESPONSE_TO_BUTYRATE_SULINDAC_4 | -0.16392 | -0.06567 | -2.36598 | 0.020934 | 0.277184 | -3.3523 |
| WILSON_PROTEASES_AT_TUMOR_BONE_INTERFACE_DN | 0.171806 | -0.18831 | 2.365708 | 0.020948 | 0.277184 | -3.35286 |
| REACTOME_ANTIMICROBIAL_PEPTIDES | 0.107368 | -0.20682 | 2.365675 | 0.02095 | 0.277184 | -3.35292 |
| KEGG_NUCLEOTIDE_EXCISION_REPAIR | -0.1427 | -0.17777 | -2.36395 | 0.02104 | 0.277597 | -3.35641 |
| WIKMAN_ASBESTOS_LUNG_CANCER_UP | -0.14371 | -0.26368 | -2.36339 | 0.021069 | 0.277597 | -3.35754 |
| PID_NFAT_TFPATHWAY | 0.105608 | -0.17296 | 2.3621 | 0.021137 | 0.277597 | -3.36014 |
| WORSCHECH_TUMOR_REJECTION_UP | 0.105527 | -0.06117 | 2.361275 | 0.02118 | 0.277597 | -3.3618 |
| HUMMERICH_SKIN_CANCER_PROGRESSION_UP | 0.138014 | -0.05645 | 2.360908 | 0.021199 | 0.277597 | -3.36254 |
| SENGUPTA_EBNA1_ANTICORRELATED | 0.077494 | -0.17226 | 2.359353 | 0.021281 | 0.277908 | -3.36568 |
| SATO_SILENCED_BY_METHYLATION_IN_PANCREATIC_CANCER_1 | 0.053066 | -0.2462 | 2.3588 | 0.02131 | 0.277908 | -3.36679 |
| LUDWICZEK_TREATING_IRON_OVERLOAD | 0.182319 | -0.02636 | 2.357623 | 0.021373 | 0.27815 | -3.36916 |
| CHEOK_RESPONSE_TO_MERCAPTOPURINE_AND_HD_MTX_DN | -0.13202 | -0.17537 | -2.35531 | 0.021495 | 0.279175 | -3.37381 |
| PID_FANCONI_PATHWAY | -0.12096 | -0.07274 | -2.35308 | 0.021614 | 0.280149 | -3.37829 |
| SENGUPTA_NASOPHARYNGEAL_CARCINOMA_WITH_LMP1_DN | 0.082228 | -0.12286 | 2.352107 | 0.021667 | 0.280201 | -3.38025 |
| LEE_LIVER_CANCER_MYC_TGFA_UP | 0.132096 | -0.06243 | 2.351365 | 0.021706 | 0.280201 | -3.38175 |
| WP_PPARALPHA_PATHWAY | 0.095513 | 0.001985 | 2.34811 | 0.021882 | 0.281081 | -3.38828 |
| SMID_BREAST_CANCER_ERBB2_UP | 0.06474 | -0.13538 | 2.347117 | 0.021936 | 0.281081 | -3.39027 |
| HOEGERKORP_CD44_TARGETS_DIRECT_UP | 0.117244 | -0.03554 | 2.347045 | 0.021939 | 0.281081 | -3.39042 |
| BROWN_MYELOID_CELL_DEVELOPMENT_UP | 0.122353 | -0.13176 | 2.346631 | 0.021962 | 0.281081 | -3.39125 |
| GARGALOVIC_RESPONSE_TO_OXIDIZED_PHOSPHOLIPIDS_BLUE_DN | -0.10613 | -0.09329 | -2.34573 | 0.022011 | 0.281081 | -3.39306 |
| JAZAG_TGFB1_SIGNALING_VIA_SMAD4_DN | 0.067366 | -0.17214 | 2.345203 | 0.022039 | 0.281081 | -3.39411 |
| KIM_ALL_DISORDERS_CALB1_CORR_DN | 0.125096 | -0.08016 | 2.343503 | 0.022132 | 0.281698 | -3.39752 |
| KEGG_GLYCEROLIPID_METABOLISM | 0.076104 | -0.16832 | 2.342664 | 0.022178 | 0.281719 | -3.3992 |
| SERVITJA_ISLET_HNF1A_TARGETS_DN | 0.078799 | -0.17877 | 2.339126 | 0.022372 | 0.283584 | -3.40628 |
| WP_CELL_DIFFERENTIATION_INDEX | 0.1662 | 0.00234 | 2.337483 | 0.022463 | 0.283584 | -3.40956 |
| AMIT_SERUM_RESPONSE_40_MCF10A | 0.183675 | -0.0175 | 2.337124 | 0.022483 | 0.283584 | -3.41028 |
| REACTOME_RORA_ACTIVATES_GENE_EXPRESSION | -0.18413 | -0.01021 | -2.33676 | 0.022503 | 0.283584 | -3.411 |
| REACTOME_CHYLOMICRON_ASSEMBLY | 0.148779 | 0.01245 | 2.33511 | 0.022595 | 0.283697 | -3.41431 |
| REACTOME_POLYMERASE_SWITCHING | -0.18954 | -0.00464 | -2.335 | 0.022601 | 0.283697 | -3.41453 |
| REACTOME_CREB1_PHOSPHORYLATION_THROUGH_NMDA_RECEPTOR_MEDIATED_ACTIVATION_OF_RAS_SIGNALING | -0.11039 | -0.05249 | -2.3298 | 0.022892 | 0.286786 | -3.42491 |
| REACTOME_DEACTIVATION_OF_THE_BETA_CATENIN_TRANSACTIVATING_COMPLEX | -0.10269 | -0.01748 | -2.32826 | 0.022979 | 0.287305 | -3.42797 |
| LIU_LIVER_CANCER | 0.072258 | -0.1562 | 2.327123 | 0.023043 | 0.287544 | -3.43024 |
| REACTOME_CREB1_PHOSPHORYLATION_THROUGH_THE_ACTIVATION_OF_ADENYLATE_CYCLASE | -0.14174 | -0.14036 | -2.32545 | 0.023138 | 0.288161 | -3.43356 |
| BOYAULT_LIVER_CANCER_SUBCLASS_G123_DN | -0.08519 | -0.04431 | -2.32311 | 0.023271 | 0.289162 | -3.43822 |
| KOHOUTEK_CCNT1_TARGETS | 0.096495 | -0.08104 | 2.32245 | 0.023309 | 0.289162 | -3.43953 |
| WHITEHURST_PACLITAXEL_SENSITIVITY | 0.092236 | -0.04542 | 2.320464 | 0.023423 | 0.289618 | -3.44348 |
| PID_RHOA_REG_PATHWAY | 0.088024 | -0.09921 | 2.319845 | 0.023459 | 0.289618 | -3.44471 |
| NAGY_STAGA_COMPONENTS_HUMAN | -0.1653 | -0.00408 | -2.31943 | 0.023483 | 0.289618 | -3.44554 |
| REACTOME_REVERSIBLE_HYDRATION_OF_CARBON_DIOXIDE | 0.19355 | -0.00571 | 2.317887 | 0.023572 | 0.290154 | -3.4486 |
| SMIRNOV_CIRCULATING_ENDOTHELIOCYTES_IN_CANCER_UP | 0.129757 | -0.05837 | 2.315856 | 0.023689 | 0.291039 | -3.45263 |
| REACTOME_FORMATION_OF_APOPTOSOME | -0.23505 | -0.0265 | -2.31249 | 0.023886 | 0.292886 | -3.45931 |
| MCDOWELL_ACUTE_LUNG_INJURY_UP | 0.156162 | -0.03504 | 2.309606 | 0.024054 | 0.294389 | -3.46501 |
| FUNG_IL2_SIGNALING_2 | 0.137265 | -0.1497 | 2.308229 | 0.024136 | 0.294566 | -3.46774 |
| WP_HIF1A_AND_PPARG_REGULATION_OF_GLYCOLYSIS | 0.203386 | -0.1119 | 2.306157 | 0.024258 | 0.294566 | -3.47184 |
| BIOCARTA_PLCD_PATHWAY | -0.23568 | -0.0207 | -2.30432 | 0.024367 | 0.294566 | -3.47546 |
| DAZARD_UV_RESPONSE_CLUSTER_G6 | -0.13015 | -0.09056 | -2.30324 | 0.024431 | 0.294566 | -3.4776 |
| MINGUEZ_LIVER_CANCER_VASCULAR_INVASION_UP | -0.2047 | -0.00543 | -2.30286 | 0.024454 | 0.294566 | -3.47835 |
| TESAR_ALK_TARGETS_HUMAN_ES_5D_UP | 0.233426 | 0.015467 | 2.302163 | 0.024496 | 0.294566 | -3.47972 |
| FARMER_BREAST_CANCER_CLUSTER_3 | 0.156533 | 0.000706 | 2.301614 | 0.024529 | 0.294566 | -3.48081 |
| WP_LEPTIN_AND_ADIPONECTIN | -0.17259 | -0.17603 | -2.30101 | 0.024565 | 0.294566 | -3.48199 |
| JECHLINGER_EPITHELIAL_TO_MESENCHYMAL_TRANSITION_UP | 0.119474 | -0.19071 | 2.300101 | 0.024619 | 0.294566 | -3.48379 |
| WESTON_VEGFA_TARGETS_12HR | 0.127946 | -0.19272 | 2.300096 | 0.02462 | 0.294566 | -3.4838 |
| WP_NUCLEOTIDE_EXCISION_REPAIR | -0.14018 | -0.18176 | -2.29998 | 0.024626 | 0.294566 | -3.48403 |
| REACTOME_P75NTR_REGULATES_AXONOGENESIS | 0.167425 | -0.00823 | 2.299153 | 0.024676 | 0.294566 | -3.48566 |
| REACTOME_ATTACHMENT_AND_ENTRY | 0.213649 | -0.00465 | 2.298857 | 0.024694 | 0.294566 | -3.48625 |
| REACTOME_TP53_REGULATES_TRANSCRIPTION_OF_ADDITIONAL_CELL_CYCLE_GENES_WHOSE_EXACT_ROLE_IN_THE_P53_PATHWAY_REMAIN_UNCERTAIN | 0.121984 | -0.28492 | 2.29848 | 0.024717 | 0.294566 | -3.48699 |
| TONKS_TARGETS_OF_RUNX1_RUNX1T1_FUSION_ERYTHROCYTE_UP | 0.098925 | -0.2198 | 2.297274 | 0.02479 | 0.294716 | -3.48936 |
| TAKEDA_TARGETS_OF_NUP98_HOXA9_FUSION_16D_DN | 0.072605 | -0.14058 | 2.296737 | 0.024822 | 0.294716 | -3.49042 |
| BIOCARTA_ERBB4_PATHWAY | -0.164 | -0.01063 | -2.29543 | 0.024901 | 0.294788 | -3.493 |
| DACOSTA_ERCC3_ALLELE_XPCS_VS_TTD_UP | 0.109051 | -0.04271 | 2.295109 | 0.024921 | 0.294788 | -3.49363 |
| BIOCARTA_PLK3_PATHWAY | 0.162659 | 0.002405 | 2.293285 | 0.025032 | 0.295551 | -3.49722 |
| JAEGER_METASTASIS_DN | 0.087397 | -0.03251 | 2.291689 | 0.025129 | 0.296151 | -3.50036 |
| TOMIDA_METASTASIS_DN | 0.180783 | -0.02202 | 2.287729 | 0.025372 | 0.298189 | -3.50814 |
| LU_TUMOR_VASCULATURE_UP | 0.14889 | -0.00496 | 2.28649 | 0.025449 | 0.298189 | -3.51057 |
| REACTOME_NEGATIVE_REGULATION_OF_NMDA_RECEPTOR_MEDIATED_NEURONAL_TRANSMISSION | -0.14231 | 0.000672 | -2.28494 | 0.025545 | 0.298189 | -3.5136 |
| WENG_POR_DOSAGE | 0.115672 | -0.05018 | 2.284732 | 0.025558 | 0.298189 | -3.51402 |
| SCIAN_CELL_CYCLE_TARGETS_OF_TP53_AND_TP73_UP | 0.195069 | -0.14003 | 2.284598 | 0.025566 | 0.298189 | -3.51428 |
| PARK_TRETINOIN_RESPONSE_AND_PML_RARA_FUSION | 0.1318 | -0.17411 | 2.284322 | 0.025583 | 0.298189 | -3.51482 |
| REACTOME_MITOCHONDRIAL_PROTEIN_IMPORT | -0.17883 | -0.07354 | -2.27841 | 0.025953 | 0.301944 | -3.5264 |
| FOSTER_KDM1A_TARGETS_DN | -0.07901 | -0.07854 | -2.27507 | 0.026163 | 0.303291 | -3.53293 |
| MACLACHLAN_BRCA1_TARGETS_DN | 0.132138 | -0.25052 | 2.275068 | 0.026164 | 0.303291 | -3.53294 |
| REACTOME_INTERLEUKIN_2_FAMILY_SIGNALING | 0.107787 | -0.03404 | 2.273741 | 0.026248 | 0.303714 | -3.53553 |
| TAKEDA_TARGETS_OF_NUP98_HOXA9_FUSION_8D_DN | 0.090089 | -0.12228 | 2.266148 | 0.026735 | 0.307145 | -3.55034 |
| REACTOME_RNA_POLYMERASE_III_TRANSCRIPTION_INITIATION_FROM_TYPE_1_PROMOTER | -0.16404 | -0.05237 | -2.26573 | 0.026762 | 0.307145 | -3.55116 |
| GROSS_HYPOXIA_VIA_ELK3_ONLY_UP | 0.156941 | -0.06686 | 2.264856 | 0.026818 | 0.307145 | -3.55286 |
| REACTOME_SIGNALING_BY_ERBB2_ECD_MUTANTS | -0.14094 | -0.05065 | -2.26303 | 0.026937 | 0.307145 | -3.55641 |
| DAZARD_UV_RESPONSE_CLUSTER_G5 | 0.183167 | -0.08148 | 2.262451 | 0.026974 | 0.307145 | -3.55754 |
| BLANCO_MELO_COVID19_SARS_COV_2_LOW_MOI_INFECTION_A594_ACE2_EXPRESSING_CELLS_DN | 0.154292 | 0.010213 | 2.261796 | 0.027017 | 0.307145 | -3.55881 |
| MEISSNER_NPC_HCP_WITH_H3_UNMETHYLATED | 0.086504 | -0.07944 | 2.261517 | 0.027035 | 0.307145 | -3.55935 |
| REACTOME_TRNA_PROCESSING | -0.12983 | -0.13982 | -2.26138 | 0.027044 | 0.307145 | -3.55962 |
| XU_GH1_EXOGENOUS_TARGETS_UP | 0.11362 | -0.16858 | 2.261236 | 0.027054 | 0.307145 | -3.5599 |
| REACTOME_FATTY_ACIDS | 0.213672 | -0.03771 | 2.261143 | 0.02706 | 0.307145 | -3.56008 |
| KONDO_HYPOXIA | 0.174758 | -0.02019 | 2.2609 | 0.027076 | 0.307145 | -3.56056 |
| REACTOME_BETA_OXIDATION_OF_BUTANOYL_COA_TO_ACETYL_COA | -0.16498 | -0.02098 | -2.25879 | 0.027214 | 0.308104 | -3.56465 |
| BIOCARTA_AKAP95_PATHWAY | -0.1545 | -0.07432 | -2.25813 | 0.027257 | 0.308104 | -3.56593 |
| HOLLERN_SOLID_NODULAR_BREAST_TUMOR_DN | 0.120286 | -0.01379 | 2.254501 | 0.027496 | 0.309968 | -3.57298 |
| REACTOME_MET_RECEPTOR_ACTIVATION | 0.181342 | 0.006258 | 2.254156 | 0.027519 | 0.309968 | -3.57365 |
| REACTOME_CAMK_IV_MEDIATED_PHOSPHORYLATION_OF_CREB | -0.15194 | 0.022365 | -2.25127 | 0.027711 | 0.311119 | -3.57923 |
| REACTOME_SIGNALING_BY_FGFR4_IN_DISEASE | -0.14365 | -0.06961 | -2.25044 | 0.027766 | 0.311119 | -3.58085 |
| CALVET_IRINOTECAN_SENSITIVE_VS_RESISTANT_UP | -0.19991 | 0.004341 | -2.25041 | 0.027768 | 0.311119 | -3.5809 |
| VERHAAK_GLIOBLASTOMA_MESENCHYMAL | 0.139701 | -0.10023 | 2.246512 | 0.02803 | 0.313124 | -3.58845 |
| PID_AMB2_NEUTROPHILS_PATHWAY | 0.097593 | -0.25223 | 2.246281 | 0.028045 | 0.313124 | -3.5889 |
| REACTOME_ROLE_OF_SECOND_MESSENGERS_IN_NETRIN_1_SIGNALING | -0.14426 | 0.013853 | -2.24473 | 0.02815 | 0.313325 | -3.5919 |
| ZHAN_MULTIPLE_MYELOMA_MF_UP | 0.092376 | -0.06407 | 2.244556 | 0.028162 | 0.313325 | -3.59223 |
| NIKOLSKY_BREAST_CANCER_15Q26_AMPLICON | 0.103264 | -0.01321 | 2.242832 | 0.028279 | 0.314075 | -3.59556 |
| HARRIS_HYPOXIA | 0.145387 | -0.04047 | 2.239031 | 0.028538 | 0.3164 | -3.60289 |
| FIGUEROA_AML_METHYLATION_CLUSTER_4_UP | -0.05473 | -0.26716 | -2.23629 | 0.028726 | 0.317095 | -3.60818 |
| LEE_LIVER_CANCER_ACOX1_DN | 0.079069 | -0.11245 | 2.235991 | 0.028746 | 0.317095 | -3.60875 |
| FONTAINE_PAPILLARY_THYROID_CARCINOMA_UP | 0.085215 | -0.02558 | 2.235599 | 0.028773 | 0.317095 | -3.60951 |
| POS_HISTAMINE_RESPONSE_NETWORK | 0.128586 | -0.14059 | 2.233479 | 0.02892 | 0.317095 | -3.61359 |
| BIOCARTA_DNAFRAGMENT_PATHWAY | -0.19962 | -0.00279 | -2.23281 | 0.028966 | 0.317095 | -3.61488 |
| REACTOME_ALPHA_DEFENSINS | 0.101934 | -0.3639 | 2.232715 | 0.028973 | 0.317095 | -3.61506 |
| REACTOME_FORMATION_OF_THE_BETA_CATENIN_TCF_TRANSACTIVATING_COMPLEX | -0.13247 | -0.0304 | -2.23224 | 0.029005 | 0.317095 | -3.61596 |
| HOLLEMAN_DAUNORUBICIN_ALL_DN | -0.20091 | -0.10029 | -2.2314 | 0.029064 | 0.317095 | -3.61759 |
| ISHIDA_TARGETS_OF_SYT_SSX_FUSIONS | 0.204625 | -0.16998 | 2.231149 | 0.029081 | 0.317095 | -3.61807 |
| MISSIAGLIA_REGULATED_BY_METHYLATION_UP | 0.089072 | -0.23409 | 2.230903 | 0.029099 | 0.317095 | -3.61854 |
| NAGY_TFTC_COMPONENTS_HUMAN | -0.14866 | -0.06254 | -2.23006 | 0.029157 | 0.317193 | -3.62017 |
| BIOCARTA_PGC1A_PATHWAY | -0.15454 | -0.05812 | -2.22912 | 0.029223 | 0.317359 | -3.62196 |
| MOOTHA_VOXPHOS | -0.15462 | -0.16484 | -2.2265 | 0.029407 | 0.318275 | -3.627 |
| YAO_TEMPORAL_RESPONSE_TO_PROGESTERONE_CLUSTER_5 | 0.09058 | -0.26012 | 2.226493 | 0.029407 | 0.318275 | -3.62701 |
| GOZGIT_ESR1_TARGETS_UP | 0.064219 | -0.11754 | 2.225182 | 0.029499 | 0.318731 | -3.62953 |
| SILIGAN_TARGETS_OF_EWS_FLI1_FUSION_DN | -0.19592 | -0.05899 | -2.2228 | 0.029668 | 0.320007 | -3.6341 |
| VART_KSHV_INFECTION_ANGIOGENIC_MARKERS_DN | 0.092535 | -0.04734 | 2.218856 | 0.029948 | 0.321703 | -3.64164 |
| REACTOME_PKA_MEDIATED_PHOSPHORYLATION_OF_CREB | -0.11216 | -0.12757 | -2.21855 | 0.02997 | 0.321703 | -3.64223 |
| RHEIN_ALL_GLUCOCORTICOID_THERAPY_UP | 0.126658 | -0.1169 | 2.218455 | 0.029976 | 0.321703 | -3.64241 |
| WP_TRYPTOPHAN_CATABOLISM_LEADING_TO_NAD_PRODUCTION | 0.106382 | -0.11372 | 2.21712 | 0.030072 | 0.322185 | -3.64497 |
| DUAN_PRDM5_TARGETS | 0.062682 | -0.20451 | 2.215824 | 0.030165 | 0.322278 | -3.64744 |
| REACTOME_STIMULI_SENSING_CHANNELS | 0.080589 | -0.05556 | 2.215448 | 0.030192 | 0.322278 | -3.64816 |
| PID_UPA_UPAR_PATHWAY | 0.137155 | -0.07181 | 2.213812 | 0.03031 | 0.322278 | -3.65129 |
| ZHANG_BREAST_CANCER_PROGENITORS_UP | -0.13236 | -0.05149 | -2.21352 | 0.030331 | 0.322278 | -3.65183 |
| AMIT_DELAYED_EARLY_GENES | 0.20734 | -0.00693 | 2.213479 | 0.030334 | 0.322278 | -3.65192 |
| ISSAEVA_MLL2_TARGETS | 0.125774 | -0.00167 | 2.212129 | 0.030431 | 0.322776 | -3.6545 |
| VANDESLUIS_COMMD1_TARGETS_GROUP_3_UP | 0.074187 | -0.08873 | 2.208856 | 0.030669 | 0.324149 | -3.66074 |
| WANG_RECURRENT_LIVER_CANCER_UP | -0.13986 | -0.00552 | -2.20883 | 0.030671 | 0.324149 | -3.66079 |
| REACTOME_INHIBITION_OF_THE_PROTEOLYTIC_ACTIVITY_OF_APC_C_REQUIRED_FOR_THE_ONSET_OF_ANAPHASE_BY_MITOTIC_SPINDLE_CHECKPOINT_COMPONENTS | -0.16228 | -0.0182 | -2.20825 | 0.030714 | 0.324149 | -3.6619 |
| REACTOME_ROBO_RECEPTORS_BIND_AKAP5 | -0.15904 | -0.10427 | -2.2068 | 0.030819 | 0.32437 | -3.66465 |
| WP_BLADDER_CANCER | 0.111458 | -0.04253 | 2.206566 | 0.030837 | 0.32437 | -3.6651 |
| BOYAULT_LIVER_CANCER_SUBCLASS_G123_UP | -0.16448 | -0.00992 | -2.20536 | 0.030925 | 0.32476 | -3.66739 |
| SCHOEN_NFKB_SIGNALING | 0.173496 | -0.00232 | 2.2045 | 0.030988 | 0.324847 | -3.66904 |
| WATANABE_ULCERATIVE_COLITIS_WITH_CANCER_UP | -0.17188 | -0.11025 | -2.20365 | 0.03105 | 0.324847 | -3.67065 |
| REACTOME_MITOTIC_TELOPHASE_CYTOKINESIS | -0.21954 | -0.00933 | -2.20317 | 0.031086 | 0.324847 | -3.67156 |
| REACTOME_REPRESSION_OF_WNT_TARGET_GENES | -0.1291 | 0.006717 | -2.20219 | 0.031159 | 0.32507 | -3.67343 |
| REACTOME_NEDDYLATION | -0.10582 | -0.10504 | -2.20066 | 0.031272 | 0.32572 | -3.67634 |
| YAMASHITA_LIVER_CANCER_STEM_CELL_UP | 0.082217 | -0.21956 | 2.191688 | 0.031943 | 0.332169 | -3.69336 |
| REN_MIF_TARGETS_DN | 0.165662 | -0.17104 | 2.189282 | 0.032126 | 0.333347 | -3.69791 |
| BURTON_ADIPOGENESIS_12 | -0.14316 | -0.05126 | -2.18881 | 0.032161 | 0.333347 | -3.6988 |
| CHOI_ATL_ACUTE_STAGE | 0.070534 | -0.80814 | 2.18526 | 0.032432 | 0.335223 | -3.70551 |
| REACTOME_RMTS_METHYLATE_HISTONE_ARGININES | -0.15998 | -0.04228 | -2.18506 | 0.032448 | 0.335223 | -3.70589 |
| WP_PEPTIDE_GPCRS | 0.134394 | -0.00788 | 2.181987 | 0.032684 | 0.336562 | -3.71169 |
| WP_MIR124_PREDICTED_INTERACTIONS_WITH_CELL_CYCLE_AND_DIFFERENTIATION | -0.20592 | -0.02516 | -2.18093 | 0.032765 | 0.336562 | -3.71368 |
| BLANCO_MELO_RESPIRATORY_SYNCYTIAL_VIRUS_INFECTION_A594_CELLS_DN | 0.098958 | -0.18794 | 2.180075 | 0.032831 | 0.336562 | -3.71529 |
| TAGHAVI_NEOPLASTIC_TRANSFORMATION | 0.154819 | -0.06313 | 2.179636 | 0.032865 | 0.336562 | -3.71612 |
| WP_NETWORK_MAP_OF_SARSCOV2_SIGNALING_PATHWAY | 0.075265 | -0.17577 | 2.179056 | 0.03291 | 0.336562 | -3.71721 |
| SHIRAISHI_PLZF_TARGETS_DN | -0.15114 | 0.009197 | -2.17843 | 0.032959 | 0.336562 | -3.7184 |
| REACTOME_RESPIRATORY_ELECTRON_TRANSPORT | -0.16603 | -0.1243 | -2.17819 | 0.032977 | 0.336562 | -3.71884 |
| GINESTIER_BREAST_CANCER_ZNF217_AMPLIFIED_UP | -0.16486 | -0.01107 | -2.17789 | 0.033 | 0.336562 | -3.7194 |
| MOREAUX_B_LYMPHOCYTE_MATURATION_BY_TACI_UP | 0.072298 | -0.22458 | 2.17584 | 0.033161 | 0.337538 | -3.72327 |
| GRAHAM_CML_QUIESCENT_VS_NORMAL_QUIESCENT_UP | 0.09424 | -0.08361 | 2.174773 | 0.033244 | 0.337538 | -3.72528 |
| HINATA_NFKB_TARGETS_FIBROBLAST_UP | 0.091074 | -0.21251 | 2.174608 | 0.033257 | 0.337538 | -3.72559 |
| NAGY_PCAF_COMPONENTS_HUMAN | -0.16868 | -0.00977 | -2.17323 | 0.033365 | 0.337538 | -3.72818 |
| REACTOME_PHOSPHORYLATION_OF_THE_APC_C | -0.16034 | -0.00815 | -2.17226 | 0.033441 | 0.337538 | -3.73 |
| BIOCARTA_THELPER_PATHWAY | 0.211449 | -0.08996 | 2.172151 | 0.03345 | 0.337538 | -3.73021 |
| REACTOME_BETA_OXIDATION_OF_VERY_LONG_CHAIN_FATTY_ACIDS | -0.15021 | -0.07656 | -2.17154 | 0.033498 | 0.337538 | -3.73136 |
| REACTOME_RRNA_PROCESSING_IN_THE_MITOCHONDRION | -0.19549 | -0.08767 | -2.17041 | 0.033587 | 0.337538 | -3.73347 |
| LI_WILMS_TUMOR_VS_FETAL_KIDNEY_2_UP | -0.11401 | -0.04204 | -2.16995 | 0.033623 | 0.337538 | -3.73434 |
| VERRECCHIA_EARLY_RESPONSE_TO_TGFB1 | 0.146764 | -0.03542 | 2.168237 | 0.033759 | 0.337538 | -3.73756 |
| REACTOME_NR1H2_NR1H3_REGULATE_GENE_EXPRESSION_LINKED_TO_LIPOGENESIS | 0.130761 | -0.32399 | 2.166129 | 0.033926 | 0.337538 | -3.74151 |
| WARTERS_RESPONSE_TO_IR_SKIN | 0.066664 | -0.10528 | 2.165426 | 0.033982 | 0.337538 | -3.74283 |
| WAGSCHAL_EHMT2_TARGETS_UP | 0.170309 | 0.013812 | 2.165125 | 0.034006 | 0.337538 | -3.74339 |
| BASSO_CD40_SIGNALING_UP | 0.077741 | -0.40264 | 2.165031 | 0.034014 | 0.337538 | -3.74357 |
| REACTOME_RAF_ACTIVATION | -0.14937 | -0.02173 | -2.16442 | 0.034063 | 0.337538 | -3.74471 |
| DAUER_STAT3_TARGETS_UP | 0.157247 | -0.02904 | 2.163564 | 0.034131 | 0.337538 | -3.74631 |
| NADLER_OBESITY_UP | 0.156135 | -0.09424 | 2.163029 | 0.034174 | 0.337538 | -3.74732 |
| WP_PHOTODYNAMIC_THERAPYINDUCED_NFKB_SURVIVAL_SIGNALING | 0.129658 | -0.18739 | 2.162459 | 0.03422 | 0.337538 | -3.74838 |
| NAGASHIMA_EGF_SIGNALING_UP | 0.158583 | -0.14477 | 2.161842 | 0.034269 | 0.337538 | -3.74954 |
| REACTOME_TRANSCRIPTIONAL_ACTIVATION_OF_MITOCHONDRIAL_BIOGENESIS | -0.12214 | -0.04799 | -2.16006 | 0.034413 | 0.337538 | -3.75288 |
| REACTOME_RNA_POLYMERASE_II_TRANSCRIPTION | -0.07692 | -0.10083 | -2.15961 | 0.034449 | 0.337538 | -3.75371 |
| CHESLER_BRAIN_D6MIT150_QTL_CIS | -0.16849 | -0.0083 | -2.15787 | 0.03459 | 0.337538 | -3.75697 |
| WP_MALE_INFERTILITY | 0.046784 | -0.22286 | 2.157621 | 0.03461 | 0.337538 | -3.75743 |
| AMIT_SERUM_RESPONSE_240_MCF10A | 0.10428 | -0.07842 | 2.157388 | 0.034629 | 0.337538 | -3.75787 |
| KEGG_PENTOSE_AND_GLUCURONATE_INTERCONVERSIONS | 0.126683 | -0.06726 | 2.157322 | 0.034634 | 0.337538 | -3.75799 |
| REACTOME_NF_KB_IS_ACTIVATED_AND_SIGNALS_SURVIVAL | 0.166611 | -0.0799 | 2.155679 | 0.034767 | 0.337538 | -3.76106 |
| REACTOME_COENZYME_A_BIOSYNTHESIS | -0.15326 | -0.10577 | -2.15563 | 0.034771 | 0.337538 | -3.76114 |
| GERY_CEBP_TARGETS | 0.102308 | -0.10023 | 2.155187 | 0.034807 | 0.337538 | -3.76198 |
| WP_FRAGILE_X_SYNDROME | -0.0704 | -0.04946 | -2.15479 | 0.03484 | 0.337538 | -3.76272 |
| SMID_BREAST_CANCER_RELAPSE_IN_LIVER_UP | 0.176868 | -0.00486 | 2.154644 | 0.034852 | 0.337538 | -3.76299 |
| REACTOME_SENSORY_PERCEPTION | 0.083699 | -0.18995 | 2.154119 | 0.034895 | 0.337538 | -3.76397 |
| REACTOME_CYP2E1_REACTIONS | 0.18925 | -0.06473 | 2.15391 | 0.034912 | 0.337538 | -3.76436 |
| CADWELL_ATG16L1_TARGETS_UP | 0.070684 | -0.10781 | 2.152996 | 0.034986 | 0.337538 | -3.76606 |
| WP_CONTROL_OF_IMMUNE_TOLERANCE_BY_VASOACTIVE_INTESTINAL_PEPTIDE | 0.145072 | -0.00137 | 2.152878 | 0.034996 | 0.337538 | -3.76628 |
| REACTOME_TRANSCRIPTIONAL_REGULATION_BY_THE_AP_2_TFAP2_FAMILY_OF_TRANSCRIPTION_FACTORS | 0.087244 | 0.003782 | 2.152857 | 0.034998 | 0.337538 | -3.76632 |
| GABRIELY_MIR21_TARGETS | -0.14023 | -0.02029 | -2.15276 | 0.035006 | 0.337538 | -3.7665 |
| WIERENGA_STAT5A_TARGETS_GROUP1 | 0.103151 | -0.09551 | 2.150918 | 0.035157 | 0.337735 | -3.76994 |
| REACTOME_NTRK2_ACTIVATES_RAC1 | 0.205644 | -0.02146 | 2.150525 | 0.035189 | 0.337735 | -3.77067 |
| SUZUKI_RESPONSE_TO_TSA_AND_DECITABINE_1A | 0.135262 | -0.01724 | 2.150177 | 0.035218 | 0.337735 | -3.77132 |
| NAISHIRO_CTNNB1_TARGETS_WITH_LEF1_MOTIF | 0.163703 | 0.008616 | 2.149122 | 0.035305 | 0.337735 | -3.77328 |
| HAMAI_APOPTOSIS_VIA_TRAIL_DN | 0.066014 | -0.1248 | 2.149062 | 0.035309 | 0.337735 | -3.77339 |
| DISTECHE_ESCAPED_FROM_X_INACTIVATION | -0.16942 | 0.007861 | -2.14864 | 0.035344 | 0.337735 | -3.77418 |
| WHITFIELD_CELL_CYCLE_G1_S | -0.10837 | -0.04767 | -2.14779 | 0.035415 | 0.3379 | -3.77577 |
| FINAK_BREAST_CANCER_SDPP_SIGNATURE | 0.145897 | -0.07644 | 2.146722 | 0.035503 | 0.338233 | -3.77775 |
| MATZUK_OVULATION | 0.134253 | -0.08145 | 2.143529 | 0.035768 | 0.340033 | -3.78368 |
| LEE_LIVER_CANCER_E2F1_DN | 0.067411 | -0.07957 | 2.143167 | 0.035799 | 0.340033 | -3.78436 |
| GAL_LEUKEMIC_STEM_CELL_DN | 0.0745 | -0.14987 | 2.141764 | 0.035916 | 0.340638 | -3.78696 |
| WIERENGA_STAT5A_TARGETS_GROUP2 | 0.143673 | -0.06128 | 2.138313 | 0.036206 | 0.342874 | -3.79336 |
| REACTOME_PURINERGIC_SIGNALING_IN_LEISHMANIASIS_INFECTION | 0.155932 | -0.04665 | 2.137677 | 0.036259 | 0.342874 | -3.79454 |
| KOBAYASHI_EGFR_SIGNALING_6HR_DN | 0.116473 | -0.28143 | 2.136973 | 0.036319 | 0.342927 | -3.79585 |
| MARTENS_TRETINOIN_RESPONSE_UP | 0.102083 | -0.09896 | 2.135907 | 0.036409 | 0.343269 | -3.79782 |
| REACTOME_ACTIVATION_OF_GENE_EXPRESSION_BY_SREBF_SREBP | -0.16605 | -0.02371 | -2.13006 | 0.036907 | 0.347449 | -3.80863 |
| CHIANG_LIVER_CANCER_SUBCLASS_UNANNOTATED_UP | 0.066341 | -0.10994 | 2.127999 | 0.037084 | 0.348603 | -3.81245 |
| BIOCARTA_CYTOKINE_PATHWAY | 0.097179 | -0.27601 | 2.127061 | 0.037165 | 0.348847 | -3.81418 |
| BLANCO_MELO_COVID19_SARS_COV_2_INFECTION_CALU3_CELLS_DN | 0.140661 | 0.025792 | 2.126249 | 0.037235 | 0.34899 | -3.81567 |
| KORKOLA_CHORIOCARCINOMA_UP | 0.231189 | -0.0006 | 2.124827 | 0.037358 | 0.349618 | -3.8183 |
| WP_GABA_METABOLISM_AKA_GHB | -0.19114 | -0.02039 | -2.12421 | 0.037412 | 0.349618 | -3.81944 |
| REACTOME_RUNX1_INTERACTS_WITH_CO_FACTORS_WHOSE_PRECISE_EFFECT_ON_RUNX1_TARGETS_IS_NOT_KNOWN | -0.11827 | -0.24538 | -2.12146 | 0.037651 | 0.351337 | -3.82451 |
| WP_OLIGODENDROCYTE_SPECIFICATION_AND_DIFFERENTIATION_LEADING_TO_MYELIN_COMPONENTS_FOR_CNS | 0.083436 | -0.33888 | 2.120453 | 0.037739 | 0.351633 | -3.82635 |
| WP_SELENIUM_MICRONUTRIENT_NETWORK | 0.079041 | -0.12174 | 2.119828 | 0.037793 | 0.351633 | -3.82751 |
| MEBARKI_HCC_PROGENITOR_WNT_UP_CTNNB1_INDEPENDENT | 0.146043 | -0.097 | 2.118953 | 0.03787 | 0.351831 | -3.82911 |
| TERAO_AOX4_TARGETS_SKIN_DN | -0.1056 | -0.09124 | -2.11764 | 0.037986 | 0.352391 | -3.83154 |
| REACTOME_FORMATION_OF_FIBRIN_CLOT_CLOTTING_CASCADE | 0.088565 | -0.02915 | 2.115409 | 0.038182 | 0.353695 | -3.83563 |
| REACTOME_MUSCARINIC_ACETYLCHOLINE_RECEPTORS | 0.247468 | 0.016794 | 2.114486 | 0.038263 | 0.353935 | -3.83732 |
| BLANCO_MELO_BRONCHIAL_EPITHELIAL_CELLS_INFLUENZA_A_DEL_NS1_INFECTION_UP | 0.066605 | -0.19335 | 2.112146 | 0.038471 | 0.354945 | -3.84162 |
| ZERBINI_RESPONSE_TO_SULINDAC_UP | 0.176353 | -0.01895 | 2.111785 | 0.038503 | 0.354945 | -3.84228 |
| REACTOME_MRNA_CAPPING | -0.1455 | -0.24078 | -2.1112 | 0.038554 | 0.354945 | -3.84335 |
| REACTOME_LAGGING_STRAND_SYNTHESIS | -0.18069 | 0.001525 | -2.11074 | 0.038596 | 0.354945 | -3.84419 |
| CHEN_METABOLIC_SYNDROM_NETWORK | 0.081756 | -0.1066 | 2.108977 | 0.038753 | 0.355881 | -3.84743 |
| TURASHVILI_BREAST_CARCINOMA_DUCTAL_VS_LOBULAR_DN | -0.17848 | 0.00671 | -2.10835 | 0.03881 | 0.355885 | -3.84858 |
| REACTOME_CD22_MEDIATED_BCR_REGULATION | 0.247063 | -0.03103 | 2.106324 | 0.038991 | 0.356872 | -3.85229 |
| LINDSTEDT_DENDRITIC_CELL_MATURATION_B | 0.125435 | -0.16139 | 2.105042 | 0.039107 | 0.356872 | -3.85463 |
| LEE_LIVER_CANCER_CIPROFIBRATE_DN | 0.094293 | -0.1014 | 2.104479 | 0.039157 | 0.356872 | -3.85566 |
| TSAI_DNAJB4_TARGETS_UP | 0.153779 | -0.13353 | 2.102345 | 0.03935 | 0.356872 | -3.85956 |
| KANG_CISPLATIN_RESISTANCE_DN | -0.19163 | -0.09878 | -2.10218 | 0.039365 | 0.356872 | -3.85986 |
| WP_ENDOCHONDRAL_OSSIFICATION | 0.08383 | -0.04569 | 2.101765 | 0.039403 | 0.356872 | -3.86062 |
| WP_ENDOCHONDRAL_OSSIFICATION_WITH_SKELETAL_DYSPLASIAS | 0.08383 | -0.04569 | 2.101765 | 0.039403 | 0.356872 | -3.86062 |
| KEGG_O_GLYCAN_BIOSYNTHESIS | 0.097027 | -0.09659 | 2.101624 | 0.039416 | 0.356872 | -3.86088 |
| FAELT_B_CLL_WITH_VH3_21_DN | -0.15608 | -0.17116 | -2.10135 | 0.039441 | 0.356872 | -3.86139 |
| REACTOME_RESPIRATORY_ELECTRON_TRANSPORT_ATP_SYNTHESIS_BY_CHEMIOSMOTIC_COUPLING_AND_HEAT_PRODUCTION_BY_UNCOUPLING_PROTEINS | -0.15196 | -0.11837 | -2.10031 | 0.039535 | 0.356872 | -3.86327 |
| RICKMAN_TUMOR_DIFFERENTIATED_WELL_VS_POORLY_UP | -0.09912 | -0.09529 | -2.09994 | 0.039568 | 0.356872 | -3.86394 |
| MIKKELSEN_IPS_LCP_WITH_H3K4ME3_AND_H3K27ME3 | 0.207732 | 0.030805 | 2.099706 | 0.03959 | 0.356872 | -3.86438 |
| BIOCARTA_INFLAM_PATHWAY | 0.066634 | -0.44429 | 2.096554 | 0.039878 | 0.357868 | -3.87013 |
| WP_LIPID_METABOLISM_PATHWAY | -0.0917 | -0.11968 | -2.0957 | 0.039957 | 0.357868 | -3.87169 |
| BANDRES_RESPONSE_TO_CARMUSTIN_WITHOUT_MGMT_48HR_UP | 0.081262 | -0.16804 | 2.095182 | 0.040004 | 0.357868 | -3.87263 |
| MCBRYAN_PUBERTAL_BREAST_3_4WK_UP | 0.069543 | -0.08841 | 2.095153 | 0.040007 | 0.357868 | -3.87268 |
| LEE_LIVER_CANCER_MYC_E2F1_UP | 0.097542 | -0.04058 | 2.095092 | 0.040012 | 0.357868 | -3.87279 |
| GAZDA_DIAMOND_BLACKFAN_ANEMIA_ERYTHROID_DN | -0.06855 | -0.12982 | -2.09481 | 0.040038 | 0.357868 | -3.8733 |
| REACTOME_SEMA3A_PAK_DEPENDENT_AXON_REPULSION | 0.163794 | -0.01754 | 2.092496 | 0.040252 | 0.358776 | -3.87752 |
| WP_ARACHIDONATE_EPOXYGENASE_EPOXIDE_HYDROLASE | -0.16863 | -0.00428 | -2.09171 | 0.040324 | 0.358776 | -3.87895 |
| RASHI_RESPONSE_TO_IONIZING_RADIATION_5 | -0.06721 | -0.12357 | -2.09146 | 0.040348 | 0.358776 | -3.87941 |
| REACTOME_FORMATION_OF_THE_EARLY_ELONGATION_COMPLEX | -0.12461 | -0.2936 | -2.09127 | 0.040365 | 0.358776 | -3.87975 |
| REACTOME_AMYLOID_FIBER_FORMATION | 0.098808 | -0.02082 | 2.089551 | 0.040525 | 0.359693 | -3.88288 |
| BIOCARTA_BBCELL_PATHWAY | 0.068976 | -0.58674 | 2.087523 | 0.040714 | 0.360866 | -3.88656 |
| HASINA_NOL7_TARGETS_UP | 0.189272 | -0.0052 | 2.085359 | 0.040916 | 0.361219 | -3.89049 |
| AMIT_EGF_RESPONSE_480_MCF10A | 0.108349 | -0.09569 | 2.084718 | 0.040976 | 0.361219 | -3.89165 |
| REACTOME_DARPP_32_EVENTS | -0.11553 | -0.0685 | -2.08434 | 0.041011 | 0.361219 | -3.89233 |
| WP_UREA_CYCLE_AND_ASSOCIATED_PATHWAYS | -0.12779 | -0.00514 | -2.0843 | 0.041015 | 0.361219 | -3.8924 |
| MULLIGHAN_NPM1_MUTATED_SIGNATURE_2_UP | -0.07755 | -0.05103 | -2.08363 | 0.041079 | 0.361219 | -3.89363 |
| REACTOME_REGULATION_OF_CHOLESTEROL_BIOSYNTHESIS_BY_SREBP_SREBF | -0.16784 | -0.01954 | -2.08346 | 0.041094 | 0.361219 | -3.89392 |
| WIERENGA_STAT5A_TARGETS_UP | 0.108264 | -0.07829 | 2.082802 | 0.041156 | 0.361268 | -3.89512 |
| LU_TUMOR_ENDOTHELIAL_MARKERS_UP | 0.146813 | -0.00455 | 2.081509 | 0.041278 | 0.36184 | -3.89747 |
| BIOCARTA_VEGF_PATHWAY | -0.13654 | -0.04548 | -2.08046 | 0.041377 | 0.362209 | -3.89936 |
| NICK_RESPONSE_TO_PROC_TREATMENT_DN | -0.12801 | -0.23232 | -2.07954 | 0.041465 | 0.3624 | -3.90104 |
| BRUNEAU_HEART_GREAT_VESSELS_AND_VALVULOGENESIS | -0.14357 | -0.01227 | -2.07903 | 0.041513 | 0.3624 | -3.90195 |
| CHIANG_LIVER_CANCER_SUBCLASS_POLYSOMY7_DN | -0.11733 | -0.00299 | -2.07833 | 0.041579 | 0.36248 | -3.90321 |
| DIRMEIER_LMP1_RESPONSE_LATE_UP | 0.150005 | -0.01241 | 2.07671 | 0.041734 | 0.362641 | -3.90615 |
| DING_LUNG_CANCER_EXPRESSION_BY_COPY_NUMBER | -0.16802 | -0.04791 | -2.0761 | 0.041792 | 0.362641 | -3.90725 |
| REACTOME_G_ALPHA_Q_SIGNALLING_EVENTS | 0.068724 | -0.02555 | 2.075678 | 0.041832 | 0.362641 | -3.90802 |
| NUYTTEN_EZH2_TARGETS_DN | -0.10084 | -0.10679 | -2.07537 | 0.041862 | 0.362641 | -3.90856 |
| KEGG_HISTIDINE_METABOLISM | -0.11484 | -0.03105 | -2.07515 | 0.041883 | 0.362641 | -3.90896 |
| REACTOME_GLOBAL_GENOME_NUCLEOTIDE_EXCISION_REPAIR_GG_NER | -0.13425 | -0.18793 | -2.07408 | 0.041985 | 0.362771 | -3.9109 |
| ROZANOV_MMP14_CORRELATED | -0.20566 | -0.00648 | -2.0729 | 0.042099 | 0.362771 | -3.91304 |
| BIOCARTA_CARM1_PATHWAY | -0.14062 | -0.07018 | -2.07287 | 0.042101 | 0.362771 | -3.91308 |
| REACTOME_INOSITOL_PHOSPHATE_METABOLISM | -0.07221 | -0.08704 | -2.07262 | 0.042126 | 0.362771 | -3.91354 |
| TURASHVILI_BREAST_NORMAL_DUCTAL_VS_LOBULAR_DN | -0.18838 | -0.01077 | -2.0711 | 0.042272 | 0.363326 | -3.91627 |
| REACTOME_SHC_RELATED_EVENTS_TRIGGERED_BY_IGF1R | -0.15767 | -0.0922 | -2.06997 | 0.042381 | 0.363326 | -3.91831 |
| REACTOME_RELEASE_OF_APOPTOTIC_FACTORS_FROM_THE_MITOCHONDRIA | -0.1692 | -0.17445 | -2.06995 | 0.042383 | 0.363326 | -3.91834 |
| DAZARD_UV_RESPONSE_CLUSTER_G28 | 0.125619 | -0.27248 | 2.069582 | 0.042419 | 0.363326 | -3.91902 |
| BOYERINAS_ONCOFETAL_TARGETS_OF_LET7A1 | -0.11892 | -0.07069 | -2.06834 | 0.042538 | 0.363681 | -3.92124 |
| REACTOME_REGULATION_OF_INSULIN_LIKE_GROWTH_FACTOR_IGF_TRANSPORT_AND_UPTAKE_BY_INSULIN_LIKE_GROWTH_FACTOR_BINDING_PROTEINS_IGFBPS | 0.090634 | -0.03026 | 2.067975 | 0.042574 | 0.363681 | -3.92191 |
| ZHAN_MULTIPLE_MYELOMA_CD1_DN | 0.107949 | -0.05334 | 2.066803 | 0.042688 | 0.364164 | -3.92402 |
| MILI_PSEUDOPODIA_HAPTOTAXIS_UP | -0.14836 | -0.06343 | -2.06526 | 0.042838 | 0.364482 | -3.9268 |
| WP_ALTERNATIVE_PATHWAY_OF_FETAL_ANDROGEN_SYNTHESIS | 0.134321 | -0.07338 | 2.065245 | 0.04284 | 0.364482 | -3.92683 |
| MATZUK_SPERMATID_DIFFERENTIATION | -0.06905 | -0.07657 | -2.06441 | 0.042922 | 0.364691 | -3.92833 |
| HOFFMANN_IMMATURE_TO_MATURE_B_LYMPHOCYTE_UP | 0.087872 | -0.26752 | 2.062915 | 0.043068 | 0.365443 | -3.93102 |
| ZAIDI_OSTEOBLAST_TRANSCRIPTION_FACTORS | 0.159662 | 0.000641 | 2.06146 | 0.04321 | 0.366166 | -3.93363 |
| IIZUKA_LIVER_CANCER_PROGRESSION_L0_L1_UP | -0.12977 | 0.009623 | -2.06074 | 0.043281 | 0.366195 | -3.93492 |
| REACTOME_SIGNALING_BY_GPCR | 0.070584 | -0.08599 | 2.060256 | 0.043329 | 0.366195 | -3.93579 |
| REACTOME_CREATION_OF_C4_AND_C2_ACTIVATORS | 0.13507 | -0.01263 | 2.057226 | 0.043628 | 0.368237 | -3.94123 |
| REACTOME_TRNA_MODIFICATION_IN_THE_NUCLEUS_AND_CYTOSOL | -0.13964 | -0.00279 | -2.05549 | 0.043801 | 0.368646 | -3.94435 |
| REACTOME_ASPARTATE_AND_ASPARAGINE_METABOLISM | -0.16635 | -0.10132 | -2.0553 | 0.043819 | 0.368646 | -3.94468 |
| BIOCARTA_RANKL_PATHWAY | 0.104636 | -0.0663 | 2.054988 | 0.043851 | 0.368646 | -3.94524 |
| REACTOME_PLASMA_LIPOPROTEIN_ASSEMBLY | 0.1082 | -0.057 | 2.054175 | 0.043932 | 0.368839 | -3.94669 |
| REACTOME_IRS_MEDIATED_SIGNALLING | -0.08002 | -0.00703 | -2.053 | 0.044049 | 0.369254 | -3.94879 |
| BANDRES_RESPONSE_TO_CARMUSTIN_MGMT_48HR_DN | 0.055011 | -0.15026 | 2.052518 | 0.044097 | 0.369254 | -3.94966 |
| WP_PHOTODYNAMIC_THERAPYINDUCED_HIF1_SURVIVAL_SIGNALING | 0.132476 | -0.05832 | 2.051048 | 0.044244 | 0.369904 | -3.95229 |
| LIU_VAV3_PROSTATE_CARCINOGENESIS_UP | 0.074607 | -0.27336 | 2.050583 | 0.044291 | 0.369904 | -3.95312 |
| MA_MYELOID_DIFFERENTIATION_DN | 0.104899 | -0.07003 | 2.048665 | 0.044484 | 0.370221 | -3.95655 |
| RIEGE_DELTANP63_DIRECT_TARGETS_UP | 0.059102 | -0.04178 | 2.048637 | 0.044487 | 0.370221 | -3.9566 |
| REACTOME_PROTEIN_LOCALIZATION | -0.12021 | -0.09834 | -2.04763 | 0.044589 | 0.370221 | -3.9584 |
| REACTOME_RUNX2_REGULATES_GENES_INVOLVED_IN_CELL_MIGRATION | 0.17258 | -0.09066 | 2.047438 | 0.044608 | 0.370221 | -3.95874 |
| REACTOME_CELL_CELL_JUNCTION_ORGANIZATION | 0.089999 | -0.01772 | 2.047319 | 0.04462 | 0.370221 | -3.95895 |
| SU_PLACENTA | 0.13851 | -0.09206 | 2.045102 | 0.044844 | 0.371119 | -3.96291 |
| PID_AP1_PATHWAY | 0.103889 | -0.11306 | 2.045099 | 0.044845 | 0.371119 | -3.96292 |
| REACTOME_MISCELLANEOUS_SUBSTRATES | 0.161521 | -0.05499 | 2.04121 | 0.045241 | 0.373521 | -3.96985 |
| NAKAMURA_LUNG_CANCER_DIFFERENTIATION_MARKERS | 0.111009 | -0.34376 | 2.041097 | 0.045252 | 0.373521 | -3.97005 |
| REACTOME_SMALL_INTERFERING_RNA_SIRNA_BIOGENESIS | -0.19952 | -0.02576 | -2.03882 | 0.045486 | 0.374506 | -3.9741 |
| REACTOME_SYNTHESIS_OF_PIPS_AT_THE_LATE_ENDOSOME_MEMBRANE | -0.12825 | -0.08174 | -2.03837 | 0.045532 | 0.374506 | -3.9749 |
| REACTOME_TIE2_SIGNALING | -0.14844 | -0.03742 | -2.03801 | 0.045568 | 0.374506 | -3.97553 |
| BIOCARTA_WNT_LRP6_PATHWAY | 0.187113 | 0.019251 | 2.037641 | 0.045607 | 0.374506 | -3.9762 |
| REACTOME_DEFENSINS | 0.08326 | -0.31236 | 2.033227 | 0.046063 | 0.376645 | -3.98404 |
| REACTOME_RRNA_MODIFICATION_IN_THE_MITOCHONDRION | -0.19719 | -0.13129 | -2.03256 | 0.046133 | 0.376645 | -3.98522 |
| REACTOME_MITOTIC_SPINDLE_CHECKPOINT | -0.12019 | -0.04247 | -2.03249 | 0.04614 | 0.376645 | -3.98534 |
| REACTOME_SNRNP_ASSEMBLY | -0.15362 | -0.06162 | -2.03235 | 0.046154 | 0.376645 | -3.98559 |
| TESAR_ALK_TARGETS_EPISC_4D_UP | 0.208873 | -0.0035 | 2.031841 | 0.046208 | 0.376645 | -3.9865 |
| VANTVEER_BREAST_CANCER_ESR1_UP | -0.09178 | -0.07353 | -2.0314 | 0.046254 | 0.376645 | -3.98729 |
| ODONNELL_TARGETS_OF_MYC_AND_TFRC_UP | 0.115983 | -0.16562 | 2.031129 | 0.046282 | 0.376645 | -3.98776 |
| ZHOU_INFLAMMATORY_RESPONSE_FIMA_UP | 0.053685 | -0.16336 | 2.030223 | 0.046376 | 0.376933 | -3.98937 |
| REACTOME_VLDL_CLEARANCE | 0.171654 | 0.015296 | 2.029526 | 0.046449 | 0.376933 | -3.9906 |
| STEARMAN_LUNG_CANCER_EARLY_VS_LATE_DN | 0.10012 | -0.18821 | 2.028563 | 0.04655 | 0.376933 | -3.99231 |
| FIGUEROA_AML_METHYLATION_CLUSTER_4_DN | 0.113025 | -0.05698 | 2.028525 | 0.046554 | 0.376933 | -3.99238 |
| BILANGES_SERUM_SENSITIVE_VIA_TSC1 | 0.120676 | -0.14102 | 2.02752 | 0.046659 | 0.377274 | -3.99416 |
| REACTOME_P75NTR_SIGNALS_VIA_NF_KB | 0.16064 | -0.06304 | 2.026331 | 0.046784 | 0.377274 | -3.99626 |
| BIOCARTA_LYMPHOCYTE_PATHWAY | 0.179473 | -0.01436 | 2.025892 | 0.046831 | 0.377274 | -3.99704 |
| KEGG_UBIQUITIN_MEDIATED_PROTEOLYSIS | -0.11524 | -0.03923 | -2.02587 | 0.046833 | 0.377274 | -3.99708 |
| REACTOME_METHIONINE_SALVAGE_PATHWAY | -0.22999 | -0.0121 | -2.0253 | 0.046893 | 0.37728 | -3.99809 |
| MATZUK_SPERMATOGONIA | 0.09667 | -0.03195 | 2.023749 | 0.047057 | 0.377567 | -4.00083 |
| BEGUM_TARGETS_OF_PAX3_FOXO1_FUSION_DN | 0.112163 | -0.05926 | 2.023278 | 0.047107 | 0.377567 | -4.00166 |
| SCHAVOLT_TARGETS_OF_TP53_AND_TP63 | 0.100106 | -0.0013 | 2.023275 | 0.047107 | 0.377567 | -4.00166 |
| REACTOME_RESOLUTION_OF_D_LOOP_STRUCTURES_THROUGH_SYNTHESIS_DEPENDENT_STRAND_ANNEALING_SDSA | -0.12047 | -0.03055 | -2.02203 | 0.047239 | 0.378032 | -4.00387 |
| STARK_PREFRONTAL_CORTEX_22Q11_DELETION_DN | -0.13681 | -0.07548 | -2.0211 | 0.047338 | 0.378032 | -4.0055 |
| REACTOME_CROSS_PRESENTATION_OF_PARTICULATE_EXOGENOUS_ANTIGENS_PHAGOSOMES | 0.194589 | -0.00318 | 2.02105 | 0.047343 | 0.378032 | -4.0056 |
| LEIN_ASTROCYTE_MARKERS | 0.084239 | -0.06737 | 2.019667 | 0.04749 | 0.378732 | -4.00804 |
| REACTOME_O_LINKED_GLYCOSYLATION_OF_MUCINS | 0.07866 | -0.07855 | 2.018327 | 0.047633 | 0.379398 | -4.0104 |
| REACTOME_APEX1_INDEPENDENT_RESOLUTION_OF_AP_SITES_VIA_THE_SINGLE_NUCLEOTIDE_REPLACEMENT_PATHWAY | -0.2097 | 0.003091 | -2.01763 | 0.047708 | 0.379517 | -4.01163 |
| BROWNE_HCMV_INFECTION_6HR_DN | -0.08223 | -0.11664 | -2.01659 | 0.04782 | 0.379593 | -4.01347 |
| HELLER_HDAC_TARGETS_SILENCED_BY_METHYLATION_UP | 0.060786 | -0.16342 | 2.015858 | 0.047898 | 0.379593 | -4.01475 |
| ISHIKAWA_STING_SIGNALING | 0.099278 | -0.2262 | 2.015702 | 0.047915 | 0.379593 | -4.01503 |
| WP_MED_AND_PSEUDOACHONDROPLASIA_GENES | 0.200116 | 0.020997 | 2.015316 | 0.047956 | 0.379593 | -4.01571 |
| HOLLERN_MICROACINAR_BREAST_TUMOR_DN | 0.126424 | -0.0618 | 2.014699 | 0.048023 | 0.379647 | -4.01679 |
| REACTOME_INTERLEUKIN_1_PROCESSING | 0.163218 | -0.09602 | 2.013873 | 0.048111 | 0.379878 | -4.01825 |
| BYSTRYKH_HEMATOPOIESIS_STEM_CELL_SCP2_QTL_TRANS | 0.083852 | -0.00831 | 2.011979 | 0.048316 | 0.38057 | -4.02158 |
| BIOCARTA_AGPCR_PATHWAY | -0.12789 | -0.08947 | -2.01171 | 0.048345 | 0.38057 | -4.02205 |
| RICKMAN_TUMOR_DIFFERENTIATED_WELL_VS_MODERATELY_UP | -0.1036 | -0.07539 | -2.01121 | 0.0484 | 0.38057 | -4.02294 |
| REACTOME_CRMPS_IN_SEMA3A_SIGNALING | 0.142186 | -0.00198 | 2.010851 | 0.048438 | 0.38057 | -4.02356 |
| DIAZ_CHRONIC_MYELOGENOUS_LEUKEMIA_DN | 0.099323 | -0.14647 | 2.008196 | 0.048727 | 0.382329 | -4.02823 |
| SESTO_RESPONSE_TO_UV_C4 | -0.1515 | 0.000455 | -2.00677 | 0.048883 | 0.382329 | -4.03073 |
| REACTOME_RNA_POLYMERASE_II_TRANSCRIBES_SNRNA_GENES | -0.15932 | -0.03205 | -2.00669 | 0.048891 | 0.382329 | -4.03087 |
| CHEN_HOXA5_TARGETS_6HR_UP | 0.182913 | -0.09934 | 2.006587 | 0.048903 | 0.382329 | -4.03105 |
| REACTOME_CONSTITUTIVE_SIGNALING_BY_EGFRVIII | -0.12947 | -0.05789 | -2.00496 | 0.04908 | 0.383249 | -4.0339 |
| WP_HFE_EFFECT_ON_HEPCIDIN_PRODUCTION | 0.180533 | -0.00938 | 2.002774 | 0.049321 | 0.384527 | -4.03773 |
| MEBARKI_HCC_PROGENITOR_WNT_DN_CTNNB1_DEPENDENT | 0.079917 | -0.21951 | 2.002375 | 0.049365 | 0.384527 | -4.03843 |
| KEGG_PRIMARY_BILE_ACID_BIOSYNTHESIS | 0.104799 | -0.0525 | 1.999595 | 0.049672 | 0.38611 | -4.0433 |
| TERAO_AOX4_TARGETS_HG_DN | 0.186001 | -0.00619 | 1.999439 | 0.04969 | 0.38611 | -4.04357 |
| SA_G1_AND_S_PHASES | 0.106616 | -0.15864 | 1.997946 | 0.049855 | 0.386926 | -4.04618 |
| SHIN_B_CELL_LYMPHOMA_CLUSTER_5 | 0.077132 | -0.32333 | 1.99691 | 0.04997 | 0.387347 | -4.04799 |
| BLANCO_MELO_BETA_INTERFERON_TREATED_BRONCHIAL_EPITHELIAL_CELLS_UP | 0.080881 | -0.07917 | 1.995819 | 0.050092 | 0.387818 | -4.0499 |
| ZHONG_RESPONSE_TO_AZACITIDINE_AND_TSA_DN | -0.12189 | -0.02434 | -1.99402 | 0.050293 | 0.388643 | -4.05304 |
| JECHLINGER_EPITHELIAL_TO_MESENCHYMAL_TRANSITION_DN | 0.118617 | -0.02274 | 1.993775 | 0.050321 | 0.388643 | -4.05346 |
| REACTOME_CRISTAE_FORMATION | -0.17882 | -0.01163 | -1.99101 | 0.050631 | 0.389613 | -4.05828 |
| WP_GPCRS_OTHER | 0.09408 | -0.10888 | 1.990495 | 0.050689 | 0.389613 | -4.05918 |
| REACTOME_ANTI_INFLAMMATORY_RESPONSE_FAVOURING_LEISHMANIA_PARASITE_INFECTION | 0.061613 | -0.04845 | 1.989979 | 0.050748 | 0.389613 | -4.06008 |
| REACTOME_FREE_FATTY_ACID_RECEPTORS | 0.211381 | 0.006125 | 1.98965 | 0.050785 | 0.389613 | -4.06065 |
| REACTOME_RUNX3_REGULATES_CDKN1A_TRANSCRIPTION | 0.173834 | -0.01503 | 1.988558 | 0.050908 | 0.389613 | -4.06256 |
| REACTOME_REGULATION_OF_TP53_ACTIVITY_THROUGH_PHOSPHORYLATION | -0.09404 | -0.0889 | -1.98849 | 0.050916 | 0.389613 | -4.06267 |
| REACTOME_VEGF_LIGAND_RECEPTOR_INTERACTIONS | 0.154434 | -0.02864 | 1.98847 | 0.050918 | 0.389613 | -4.06271 |
| REACTOME_PLATELET_ACTIVATION_SIGNALING_AND_AGGREGATION | 0.071003 | -0.07828 | 1.988311 | 0.050936 | 0.389613 | -4.06299 |
| VERRECCHIA_RESPONSE_TO_TGFB1_C3 | 0.163493 | -0.06228 | 1.986954 | 0.05109 | 0.390321 | -4.06535 |
| BIOCARTA_PLCE_PATHWAY | -0.13535 | -0.08489 | -1.98537 | 0.05127 | 0.391228 | -4.0681 |
| LOPES_METHYLATED_IN_COLON_CANCER_UP | 0.085254 | -0.07541 | 1.984353 | 0.051386 | 0.391642 | -4.06987 |
| CHEN_HOXA5_TARGETS_9HR_DN | -0.12297 | -0.0505 | -1.9838 | 0.051449 | 0.391655 | -4.07083 |
| REACTOME_COMPLEX_I_BIOGENESIS | -0.12583 | -0.18718 | -1.98305 | 0.051535 | 0.391835 | -4.07213 |
| REACTOME_TRANSPORT_OF_NUCLEOSIDES_AND_FREE_PURINE_AND_PYRIMIDINE_BASES_ACROSS_THE_PLASMA_MEMBRANE | 0.110779 | -0.00762 | 1.981135 | 0.051754 | 0.393036 | -4.07546 |
| REACTOME_APOPTOTIC_FACTOR_MEDIATED_RESPONSE | -0.14679 | -0.06542 | -1.97999 | 0.051886 | 0.393404 | -4.07745 |
| REACTOME_ANTIGEN_PROCESSING_UBIQUITINATION_PROTEASOME_DEGRADATION | -0.09523 | -0.09098 | -1.97964 | 0.051926 | 0.393404 | -4.07805 |
| ZHAN_MULTIPLE_MYELOMA_DN | 0.091868 | -0.26244 | 1.978742 | 0.05203 | 0.393717 | -4.0796 |
| KERLEY_RESPONSE_TO_CISPLATIN_UP | 0.100287 | -0.13766 | 1.977572 | 0.052165 | 0.39393 | -4.08163 |
| SCHMIDT_POR_TARGETS_IN_LIMB_BUD_DN | 0.138619 | -0.17545 | 1.976717 | 0.052264 | 0.39393 | -4.08311 |
| PID_CMYB_PATHWAY | 0.102437 | -0.03734 | 1.976562 | 0.052281 | 0.39393 | -4.08338 |
| WP_METAPATHWAY_BIOTRANSFORMATION_PHASE_I_AND_II | 0.069729 | -0.08935 | 1.976357 | 0.052305 | 0.39393 | -4.08374 |
| REACTOME_REMOVAL_OF_AMINOTERMINAL_PROPEPTIDES_FROM_GAMMA_CARBOXYLATED_PROTEINS | 0.148283 | 0.014551 | 1.974967 | 0.052466 | 0.394677 | -4.08614 |
| REACTOME_HEME_BIOSYNTHESIS | -0.13741 | 0.008797 | -1.97372 | 0.052611 | 0.394886 | -4.0883 |
| JOHNSTONE_PARVB_TARGETS_3_DN | -0.11793 | -0.10831 | -1.97366 | 0.052618 | 0.394886 | -4.0884 |
| REACTOME_NEUROFASCIN_INTERACTIONS | 0.144753 | -0.00128 | 1.97251 | 0.052752 | 0.394943 | -4.09039 |
| SU_TESTIS | -0.08634 | -0.07031 | -1.97216 | 0.052794 | 0.394943 | -4.091 |
| ZWANG_CLASS_3_TRANSIENTLY_INDUCED_BY_EGF | 0.119714 | -0.07876 | 1.971998 | 0.052812 | 0.394943 | -4.09127 |
| PLASARI_TGFB1_TARGETS_1HR_UP | 0.123261 | -0.2017 | 1.970714 | 0.052962 | 0.395601 | -4.09349 |
| SANSOM_APC_TARGETS_DN | 0.058048 | -0.13225 | 1.968107 | 0.053268 | 0.397005 | -4.09799 |
| MYLLYKANGAS_AMPLIFICATION_HOT_SPOT_9 | -0.21503 | -0.00277 | -1.96805 | 0.053275 | 0.397005 | -4.09809 |
| VARELA_ZMPSTE24_TARGETS_DN | 0.073996 | -0.05677 | 1.966693 | 0.053435 | 0.397731 | -4.10043 |
| REACTOME_CA_DEPENDENT_EVENTS | -0.08556 | -0.06938 | -1.96476 | 0.053663 | 0.398623 | -4.10376 |
| ICHIBA_GRAFT_VERSUS_HOST_DISEASE_35D_UP | 0.072037 | -0.3355 | 1.963983 | 0.053755 | 0.398623 | -4.1051 |
| MEBARKI_HCC_PROGENITOR_WNT_UP | 0.090028 | -0.06628 | 1.963715 | 0.053787 | 0.398623 | -4.10556 |
| REACTOME_PREGNENOLONE_BIOSYNTHESIS | 0.113745 | -0.06815 | 1.963382 | 0.053827 | 0.398623 | -4.10613 |
| WP_BENZENE_METABOLISM | 0.161926 | -0.01626 | 1.963033 | 0.053868 | 0.398623 | -4.10673 |
| REACTOME_NUCLEOTIDE_EXCISION_REPAIR | -0.13156 | -0.15189 | -1.96192 | 0.054 | 0.398689 | -4.10864 |
| LINDGREN_BLADDER_CANCER_WITH_LOH_IN_CHR9Q | -0.14477 | -0.00626 | -1.96191 | 0.054002 | 0.398689 | -4.10868 |
| VANDESLUIS_NORMAL_EMBRYOS_DN | 0.124619 | -0.00614 | 1.96034 | 0.054189 | 0.399603 | -4.11137 |
| WEBER_METHYLATED_HCP_IN_SPERM_UP | 0.150093 | -0.04884 | 1.959243 | 0.05432 | 0.400106 | -4.11325 |
| REACTOME_ELECTRIC_TRANSMISSION_ACROSS_GAP_JUNCTIONS | 0.175773 | -0.16739 | 1.958441 | 0.054416 | 0.400118 | -4.11463 |
| CHIANG_LIVER_CANCER_SUBCLASS_CTNNB1_DN | 0.090357 | -0.02305 | 1.957547 | 0.054523 | 0.400118 | -4.11617 |
| REACTOME_TRNA_PROCESSING_IN_THE_NUCLEUS | -0.10164 | -0.22526 | -1.95735 | 0.054547 | 0.400118 | -4.11651 |
| WP_FIBRIN_COMPLEMENT_RECEPTOR_3_SIGNALING_PATHWAY | 0.099855 | -0.13878 | 1.955363 | 0.054786 | 0.400118 | -4.11991 |
| REACTOME_ASSEMBLY_OF_THE_ORC_COMPLEX_AT_THE_ORIGIN_OF_REPLICATION | -0.14839 | -0.00061 | -1.95481 | 0.054853 | 0.400118 | -4.12086 |
| POOLA_INVASIVE_BREAST_CANCER_UP | 0.099873 | -0.19003 | 1.954717 | 0.054864 | 0.400118 | -4.12102 |
| SHEPARD_BMYB_MORPHOLINO_DN | 0.057907 | -0.11218 | 1.954276 | 0.054917 | 0.400118 | -4.12178 |
| KERLEY_RESPONSE_TO_CISPLATIN_DN | -0.16357 | -0.00575 | -1.95425 | 0.05492 | 0.400118 | -4.12183 |
| BIOCARTA_MONOCYTE_PATHWAY | 0.177702 | -0.00605 | 1.954048 | 0.054944 | 0.400118 | -4.12217 |
| BIOCARTA_ACH_PATHWAY | -0.14068 | -0.05563 | -1.954 | 0.05495 | 0.400118 | -4.12225 |
| REACTOME_METABOLISM_OF_FAT_SOLUBLE_VITAMINS | 0.080479 | -0.09456 | 1.953128 | 0.055056 | 0.400427 | -4.12374 |
| HOUSTIS_ROS | 0.12181 | -0.06947 | 1.951635 | 0.055237 | 0.401055 | -4.1263 |
| MATHEW_FANCONI_ANEMIA_GENES | -0.15388 | 0.01035 | -1.9511 | 0.055301 | 0.401055 | -4.12721 |
| REACTOME_TNFS_BIND_THEIR_PHYSIOLOGICAL_RECEPTORS | 0.106307 | -0.08225 | 1.950857 | 0.055331 | 0.401055 | -4.12763 |
| MOREAUX_MULTIPLE_MYELOMA_BY_TACI_DN | -0.14122 | -0.06944 | -1.9498 | 0.055459 | 0.401239 | -4.12944 |
| GALI_TP53_TARGETS_APOPTOTIC_DN | 0.145149 | -0.01074 | 1.949181 | 0.055535 | 0.401239 | -4.1305 |
| GOUYER_TUMOR_INVASIVENESS | 0.13873 | -0.01319 | 1.949095 | 0.055546 | 0.401239 | -4.13065 |
| CREIGHTON_ENDOCRINE_THERAPY_RESISTANCE_2 | -0.04673 | -0.0292 | -1.94831 | 0.055642 | 0.401477 | -4.13199 |
| WANG_ESOPHAGUS_CANCER_VS_NORMAL_UP | 0.084583 | -0.1949 | 1.947358 | 0.055758 | 0.401859 | -4.13362 |
| YAO_TEMPORAL_RESPONSE_TO_PROGESTERONE_CLUSTER_13 | -0.12083 | -0.1956 | -1.94545 | 0.055991 | 0.40248 | -4.13687 |
| MIKKELSEN_MCV6_LCP_WITH_H3K27ME3 | 0.137699 | -0.07058 | 1.945257 | 0.056015 | 0.40248 | -4.13721 |
| REACTOME_MITOTIC_PROMETAPHASE | -0.09704 | -0.09586 | -1.94511 | 0.056033 | 0.40248 | -4.13746 |
| WP_INTERLEUKIN1_INDUCED_ACTIVATION_OF_NFKB | 0.128358 | -0.10687 | 1.942545 | 0.056349 | 0.40359 | -4.14183 |
| WP_IL18_SIGNALING_PATHWAY | 0.062934 | -0.18965 | 1.942013 | 0.056415 | 0.40359 | -4.14274 |
| WP_PROTEOGLYCAN_BIOSYNTHESIS | -0.17277 | -0.06887 | -1.94139 | 0.056491 | 0.40359 | -4.1438 |
| REACTOME_PROTON_COUPLED_MONOCARBOXYLATE_TRANSPORT | 0.176423 | -0.15141 | 1.941099 | 0.056528 | 0.40359 | -4.1443 |
| BIOCARTA_PLATELETAPP_PATHWAY | 0.128174 | -0.00095 | 1.941035 | 0.056536 | 0.40359 | -4.14441 |
| KEGG_RNA_POLYMERASE | -0.14743 | -0.08305 | -1.94059 | 0.056591 | 0.40359 | -4.14517 |
| MALIK_REPRESSED_BY_ESTROGEN | 0.146539 | -0.01175 | 1.939631 | 0.05671 | 0.40359 | -4.1468 |
| WP_SELECTIVE_EXPRESSION_OF_CHEMOKINE_RECEPTORS_DURING_TCELL_POLARIZATION | 0.096415 | -0.14464 | 1.939506 | 0.056725 | 0.40359 | -4.14701 |
| TONKS_TARGETS_OF_RUNX1_RUNX1T1_FUSION_SUSTAINDED_IN_ERYTHROCYTE_UP | 0.095439 | -0.17569 | 1.939235 | 0.056759 | 0.40359 | -4.14747 |
| PAPASPYRIDONOS_UNSTABLE_ATEROSCLEROTIC_PLAQUE_UP | 0.138685 | -0.14631 | 1.93636 | 0.057117 | 0.405513 | -4.15236 |
| REACTOME_TRANSLESION_SYNTHESIS_BY_POLH | -0.15425 | -0.00491 | -1.93593 | 0.057171 | 0.405513 | -4.1531 |
| REACTOME_SYNTHESIS_OF_KETONE_BODIES | -0.12986 | -0.1 | -1.93553 | 0.05722 | 0.405513 | -4.15377 |
| REACTOME_GPCR_LIGAND_BINDING | 0.091449 | -0.09157 | 1.934316 | 0.057372 | 0.406138 | -4.15584 |
| HOFMANN_MYELODYSPLASTIC_SYNDROM_HIGH_RISK_UP | -0.13177 | 0.009782 | -1.9327 | 0.057576 | 0.407123 | -4.15859 |
| REACTOME_THE_CANONICAL_RETINOID_CYCLE_IN_RODS_TWILIGHT_VISION | 0.093185 | -0.06491 | 1.931919 | 0.057674 | 0.407363 | -4.15991 |
| REACTOME_ADHERENS_JUNCTIONS_INTERACTIONS | 0.102562 | 0.009952 | 1.931044 | 0.057784 | 0.407604 | -4.16139 |
| WP_VASOPRESSINREGULATED_WATER_REABSORPTION | -0.10097 | -0.07101 | -1.93063 | 0.057836 | 0.407604 | -4.16209 |
| HAHTOLA_MYCOSIS_FUNGOIDES_UP | 0.128972 | -0.21637 | 1.928486 | 0.058107 | 0.407793 | -4.16573 |
| NIKOLSKY_BREAST_CANCER_16Q24_AMPLICON | 0.105413 | -0.007 | 1.928453 | 0.058111 | 0.407793 | -4.16579 |
| WP_TGFBETA_SIGNALING_IN_THYROID_CELLS_FOR_EPITHELIALMESENCHYMAL_TRANSITION | 0.144747 | -0.0032 | 1.928178 | 0.058146 | 0.407793 | -4.16625 |
| WP_INSULIN_SIGNALING_IN_ADIPOCYTES_DIABETIC_CONDITION | -0.14624 | -0.09094 | -1.92726 | 0.058263 | 0.407793 | -4.16781 |
| WP_INSULIN_SIGNALING_IN_ADIPOCYTES_NORMAL_CONDITION | -0.14624 | -0.09094 | -1.92726 | 0.058263 | 0.407793 | -4.16781 |
| REACTOME_ACTIVATION_OF_PPARGC1A_PGC_1ALPHA_BY_PHOSPHORYLATION | -0.12347 | 0.014871 | -1.92665 | 0.05834 | 0.407793 | -4.16884 |
| PID_TCR_CALCIUM_PATHWAY | 0.100222 | -0.0595 | 1.925704 | 0.05846 | 0.407793 | -4.17044 |
| WP_CCL18_SIGNALING_PATHWAY | 0.118393 | -0.06365 | 1.925481 | 0.058489 | 0.407793 | -4.17082 |
| KEGG_GLYCOSYLPHOSPHATIDYLINOSITOL_GPI_ANCHOR_BIOSYNTHESIS | -0.13383 | -0.07442 | -1.92547 | 0.05849 | 0.407793 | -4.17083 |
| HOLLERN_SQUAMOUS_BREAST_TUMOR | 0.08689 | -0.13476 | 1.925368 | 0.058503 | 0.407793 | -4.17101 |
| YANAGISAWA_LUNG_CANCER_RECURRENCE | -0.19672 | -0.02101 | -1.92397 | 0.058681 | 0.408586 | -4.17337 |
| BILANGES_SERUM_SENSITIVE_GENES | 0.060826 | -0.2569 | 1.923354 | 0.05876 | 0.40869 | -4.17441 |
| REACTOME_GPVI_MEDIATED_ACTIVATION_CASCADE | 0.105656 | -0.20049 | 1.921989 | 0.058935 | 0.408801 | -4.17672 |
| REACTOME_DUAL_INCISION_IN_TC_NER | -0.13026 | -0.12591 | -1.92192 | 0.058944 | 0.408801 | -4.17684 |
| NIKOLSKY_BREAST_CANCER_17P11_AMPLICON | -0.12439 | 0.009415 | -1.92173 | 0.058969 | 0.408801 | -4.17717 |
| LEE_LIVER_CANCER_HEPATOBLAST | 0.146473 | -0.06236 | 1.920681 | 0.059103 | 0.409059 | -4.17893 |
| BIOCARTA_TCYTOTOXIC_PATHWAY | 0.190235 | -0.08561 | 1.920369 | 0.059143 | 0.409059 | -4.17946 |
| REACTOME_M_PHASE | -0.10743 | -0.1131 | -1.91993 | 0.059199 | 0.409059 | -4.18019 |
| REACTOME_METABOLISM_OF_COFACTORS | -0.13724 | -0.01824 | -1.91774 | 0.059482 | 0.41057 | -4.18389 |
| REACTOME_INTERLEUKIN_9_SIGNALING | 0.132532 | -0.00648 | 1.917129 | 0.059561 | 0.410669 | -4.18492 |
| LEE_AGING_MUSCLE_DN | -0.07686 | -0.19076 | -1.91629 | 0.05967 | 0.410732 | -4.18634 |
| REACTOME_TICAM1_DEPENDENT_ACTIVATION_OF_IRF3_IRF7 | 0.150087 | -0.07059 | 1.915563 | 0.059764 | 0.410732 | -4.18756 |
| WANG_NEOPLASTIC_TRANSFORMATION_BY_CCND1_MYC | 0.104971 | -0.00867 | 1.915322 | 0.059795 | 0.410732 | -4.18796 |
| KIM_WT1_TARGETS_UP | 0.109991 | -0.05919 | 1.915067 | 0.059828 | 0.410732 | -4.18839 |
| CREIGHTON_AKT1_SIGNALING_VIA_MTOR_DN | 0.123127 | -0.2497 | 1.914286 | 0.05993 | 0.410986 | -4.18971 |
| FRASOR_RESPONSE_TO_SERM_OR_FULVESTRANT_UP | -0.12858 | -0.04471 | -1.91215 | 0.060209 | 0.412291 | -4.1933 |
| BIOCARTA_FEEDER_PATHWAY | 0.123858 | -0.01476 | 1.911832 | 0.06025 | 0.412291 | -4.19384 |
| KEGG_OOCYTE_MEIOSIS | -0.08396 | -0.05129 | -1.91109 | 0.060347 | 0.412512 | -4.19509 |
| REACTOME_POSTMITOTIC_NUCLEAR_PORE_COMPLEX_NPC_REFORMATION | -0.16623 | -0.00629 | -1.90956 | 0.060548 | 0.413002 | -4.19766 |
| MCGOWAN_RSP6_TARGETS_UP | 0.093645 | -0.1295 | 1.909339 | 0.060576 | 0.413002 | -4.19803 |
| WP_REGULATION_OF_APOPTOSIS_BY_PARATHYROID_HORMONERELATED_PROTEIN | 0.109502 | -0.02873 | 1.909057 | 0.060613 | 0.413002 | -4.1985 |
| REACTOME_DOWNSTREAM_SIGNALING_OF_ACTIVATED_FGFR4 | -0.08518 | -0.03167 | -1.90734 | 0.06084 | 0.4141 | -4.20139 |
| REACTOME_RAS_ACTIVATION_UPON_CA2_INFLUX_THROUGH_NMDA_RECEPTOR | -0.1107 | -0.03967 | -1.90425 | 0.061247 | 0.41643 | -4.20656 |
| REACTOME_ESTABLISHMENT_OF_SISTER_CHROMATID_COHESION | -0.20157 | -0.00421 | -1.90266 | 0.061459 | 0.417001 | -4.20923 |
| SMID_BREAST_CANCER_RELAPSE_IN_LIVER_DN | -0.11158 | -0.34044 | -1.90263 | 0.061462 | 0.417001 | -4.20928 |
| REACTOME_DEFECTS_IN_COBALAMIN_B12_METABOLISM | -0.11427 | -0.00479 | -1.90196 | 0.061552 | 0.417163 | -4.2104 |
| REACTOME_DNA_REPAIR | -0.09701 | -0.1091 | -1.90075 | 0.061712 | 0.417807 | -4.21242 |
| MCMURRAY_TP53_HRAS_COOPERATION_RESPONSE_UP | 0.091482 | -0.06946 | 1.899324 | 0.061903 | 0.418258 | -4.21481 |
| CONRAD_GERMLINE_STEM_CELL | 0.132418 | -0.00831 | 1.899267 | 0.061911 | 0.418258 | -4.2149 |
| AMIT_SERUM_RESPONSE_20_MCF10A | 0.091027 | 0.007731 | 1.898751 | 0.06198 | 0.418281 | -4.21576 |
| MARTIN_NFKB_TARGETS_DN | -0.12736 | 0.003064 | -1.89763 | 0.06213 | 0.418424 | -4.21764 |
| KASLER_HDAC7_TARGETS_2_UP | 0.173408 | 0.004468 | 1.89761 | 0.062132 | 0.418424 | -4.21767 |
| NATSUME_RESPONSE_TO_INTERFERON_BETA_UP | 0.065083 | -0.1491 | 1.897014 | 0.062212 | 0.41852 | -4.21866 |
| REACTOME_ESTROGEN_STIMULATED_SIGNALING_THROUGH_PRKCZ | -0.17108 | -0.10686 | -1.89613 | 0.062331 | 0.41854 | -4.22014 |
| REACTOME_RUNX3_REGULATES_YAP1_MEDIATED_TRANSCRIPTION | -0.15332 | -0.0029 | -1.89601 | 0.062347 | 0.41854 | -4.22034 |
| REACTOME_NR1H3_NR1H2_REGULATE_GENE_EXPRESSION_LINKED_TO_CHOLESTEROL_TRANSPORT_AND_EFFLUX | 0.094533 | -0.15977 | 1.893955 | 0.062624 | 0.419723 | -4.22377 |
| PETRETTO_HEART_MASS_QTL_CIS_DN | -0.12934 | -0.08494 | -1.89373 | 0.062655 | 0.419723 | -4.22415 |
| PHONG_TNF_RESPONSE_VIA_P38_PARTIAL | 0.101883 | -0.11556 | 1.892356 | 0.06284 | 0.419769 | -4.22643 |
| BIOCARTA_NKT_PATHWAY | 0.096244 | -0.16317 | 1.89224 | 0.062856 | 0.419769 | -4.22663 |
| WP_ONECARBON_METABOLISM_AND_RELATED_PATHWAYS | 0.068187 | -0.07509 | 1.892213 | 0.06286 | 0.419769 | -4.22667 |
| KEGG_NITROGEN_METABOLISM | 0.098996 | -0.0419 | 1.890566 | 0.063083 | 0.420819 | -4.22942 |
| REACTOME_CREB1_PHOSPHORYLATION_THROUGH_THE_ACTIVATION_OF_CAMKII_CAMKK_CAMKIV_CASCASDE | -0.15307 | 0.010292 | -1.88961 | 0.063214 | 0.421247 | -4.23101 |
| FAELT_B_CLL_WITH_VH3_21_UP | 0.078969 | -0.34865 | 1.888852 | 0.063316 | 0.42139 | -4.23227 |
| BIOCARTA_FXR_PATHWAY | 0.140427 | -0.02995 | 1.888477 | 0.063367 | 0.42139 | -4.23289 |
| REACTOME_PEROXISOMAL_LIPID_METABOLISM | -0.10853 | -0.03494 | -1.8863 | 0.063665 | 0.422639 | -4.23651 |
| BIOCARTA_FOSB_PATHWAY | 0.164703 | 0.012008 | 1.88613 | 0.063688 | 0.422639 | -4.23679 |
| REACTOME_ERCC6_CSB_AND_EHMT2_G9A_POSITIVELY_REGULATE_RRNA_EXPRESSION | -0.11458 | -0.25195 | -1.8851 | 0.06383 | 0.422836 | -4.23851 |
| PID_DNA_PK_PATHWAY | -0.11321 | -0.27712 | -1.88484 | 0.063865 | 0.422836 | -4.23893 |
| WP_G_PROTEIN_SIGNALING_PATHWAYS | -0.0734 | -0.06248 | -1.88424 | 0.063948 | 0.422836 | -4.23994 |
| VALK_AML_WITH_T_8_21_TRANSLOCATION | -0.16537 | -0.1512 | -1.88398 | 0.063984 | 0.422836 | -4.24037 |
| REACTOME_TRNA_PROCESSING_IN_THE_MITOCHONDRION | -0.22662 | 0.00754 | -1.88295 | 0.064125 | 0.423033 | -4.24208 |
| KONDO_PROSTATE_CANCER_WITH_H3K27ME3 | 0.10239 | -0.12775 | 1.882795 | 0.064146 | 0.423033 | -4.24233 |
| WANG_CLASSIC_ADIPOGENIC_TARGETS_OF_PPARG | 0.131815 | -0.11303 | 1.880962 | 0.064399 | 0.424261 | -4.24537 |
| WP_MITOCHONDRIAL_COMPLEX_I_ASSEMBLY_MODEL_OXPHOS_SYSTEM | -0.11404 | -0.19904 | -1.88032 | 0.064488 | 0.424405 | -4.24643 |
| REACTOME_TANDEM_PORE_DOMAIN_POTASSIUM_CHANNELS | 0.127109 | 0.015293 | 1.87971 | 0.064572 | 0.424523 | -4.24744 |
| HOLLEMAN_PREDNISOLONE_RESISTANCE_ALL_UP | -0.13453 | -0.1318 | -1.87701 | 0.064947 | 0.426126 | -4.25191 |
| WP_INTERACTIONS_BETWEEN_IMMUNE_CELLS_AND_MICRORNAS_IN_TUMOR_MICROENVIRONMENT | 0.111884 | -0.07014 | 1.876849 | 0.06497 | 0.426126 | -4.25218 |
| MEISSNER_BRAIN_HCP_WITH_H3_UNMETHYLATED | 0.149665 | -0.0054 | 1.87651 | 0.065017 | 0.426126 | -4.25274 |
| REACTOME_TRANSCRIPTIONAL_REGULATION_BY_E2F6 | -0.08805 | -0.24062 | -1.87463 | 0.065279 | 0.427403 | -4.25584 |
| MCBRYAN_PUBERTAL_TGFB1_TARGETS_DN | 0.096752 | -0.06363 | 1.873512 | 0.065436 | 0.42799 | -4.25769 |
| REACTOME_DUAL_INCISION_IN_GG_NER | -0.12153 | -0.16962 | -1.87244 | 0.065586 | 0.428385 | -4.25946 |
| VERHAAK_AML_WITH_NPM1_MUTATED_UP | 0.080327 | -0.22341 | 1.871732 | 0.065686 | 0.428385 | -4.26063 |
| TONKS_TARGETS_OF_RUNX1_RUNX1T1_FUSION_SUSTAINED_IN_MONOCYTE_UP | 0.127649 | -0.00173 | 1.871643 | 0.065698 | 0.428385 | -4.26078 |
| REACTOME_SYNTHESIS_OF_GLYCOSYLPHOSPHATIDYLINOSITOL_GPI | -0.13851 | -0.05514 | -1.87105 | 0.065782 | 0.42849 | -4.26176 |
| WP_GLYCOGEN_SYNTHESIS_AND_DEGRADATION | -0.10603 | -0.04427 | -1.8694 | 0.066014 | 0.42849 | -4.26447 |
| BLANCO_MELO_MERS_COV_INFECTION_MCR5_CELLS_UP | 0.062109 | -0.15556 | 1.869308 | 0.066027 | 0.42849 | -4.26463 |
| REACTOME_TRANSCRIPTION_OF_THE_HIV_GENOME | -0.11508 | -0.19098 | -1.86805 | 0.066205 | 0.42849 | -4.2667 |
| LIAN_NEUTROPHIL_GRANULE_CONSTITUENTS | 0.125213 | -0.09975 | 1.86798 | 0.066215 | 0.42849 | -4.26681 |
| BUCKANOVICH_T_LYMPHOCYTE_HOMING_ON_TUMOR_DN | -0.12817 | -0.0033 | -1.86752 | 0.06628 | 0.42849 | -4.26757 |
| KORKOLA_SEMINOMA_DN | 0.186787 | 0.045549 | 1.867348 | 0.066304 | 0.42849 | -4.26785 |
| KEGG_MISMATCH_REPAIR | -0.13287 | -0.05931 | -1.86703 | 0.06635 | 0.42849 | -4.26839 |
| PLASARI_TGFB1_TARGETS_1HR_DN | -0.18583 | -0.02887 | -1.86684 | 0.066377 | 0.42849 | -4.2687 |
| PEDERSEN_METASTASIS_BY_ERBB2_ISOFORM_4 | 0.063172 | -0.10259 | 1.86647 | 0.066429 | 0.42849 | -4.2693 |
| REACTOME_MITOCHONDRIAL_IRON_SULFUR_CLUSTER_BIOGENESIS | -0.15051 | -0.00722 | -1.86583 | 0.06652 | 0.42849 | -4.27035 |
| SMIRNOV_RESPONSE_TO_IR_2HR_UP | 0.091976 | -0.22533 | 1.865811 | 0.066522 | 0.42849 | -4.27039 |
| WP_EXRNA_MECHANISM_OF_ACTION_AND_BIOGENESIS | -0.16623 | -0.00225 | -1.86395 | 0.066787 | 0.429665 | -4.27344 |
| FIGUEROA_AML_METHYLATION_CLUSTER_3_DN | 0.085955 | -0.03875 | 1.86327 | 0.066884 | 0.429665 | -4.27456 |
| KEGG_PROXIMAL_TUBULE_BICARBONATE_RECLAMATION | 0.079901 | -0.12324 | 1.863077 | 0.066912 | 0.429665 | -4.27488 |
| REACTOME_NEGATIVE_REGULATION_OF_FLT3 | 0.145763 | 0.003074 | 1.862635 | 0.066975 | 0.429665 | -4.27561 |
| WP_FOXA2_PATHWAY | -0.08939 | -0.04525 | -1.8615 | 0.067137 | 0.429935 | -4.27747 |
| REACTOME_G2_PHASE | -0.15425 | 0.007966 | -1.86139 | 0.067152 | 0.429935 | -4.27765 |
| REACTOME_NEF_MEDIATED_DOWNREGULATION_OF_MHC_CLASS_I_COMPLEX_CELL_SURFACE_EXPRESSION | 0.150735 | -0.14827 | 1.860776 | 0.067241 | 0.429935 | -4.27866 |
| REACTOME_ASSEMBLY_OF_ACTIVE_LPL_AND_LIPC_LIPASE_COMPLEXES | 0.119627 | 0.0097 | 1.860454 | 0.067287 | 0.429935 | -4.27919 |
| REACTOME_FGFR3B_LIGAND_BINDING_AND_ACTIVATION | 0.164809 | 0.020369 | 1.859972 | 0.067356 | 0.42994 | -4.27998 |
| REACTOME_RUNX1_REGULATES_EXPRESSION_OF_COMPONENTS_OF_TIGHT_JUNCTIONS | -0.15653 | -0.13374 | -1.85951 | 0.067423 | 0.42994 | -4.28074 |
| MEBARKI_HCC_PROGENITOR_WNT_UP_CTNNB1_DEPENDENT | 0.099276 | -0.05259 | 1.85881 | 0.067523 | 0.430095 | -4.28188 |
| BREDEMEYER_RAG_SIGNALING_NOT_VIA_ATM_UP | -0.06893 | -0.01087 | -1.8584 | 0.067583 | 0.430095 | -4.28256 |
| SIMBULAN_UV_RESPONSE_NORMAL_UP | 0.144141 | -0.11488 | 1.85689 | 0.0678 | 0.430347 | -4.28503 |
| VANLOO_SP3_TARGETS_DN | 0.072749 | -0.09982 | 1.856853 | 0.067805 | 0.430347 | -4.28509 |
| WP_BLOOD_CLOTTING_CASCADE | 0.099739 | -0.04169 | 1.856703 | 0.067827 | 0.430347 | -4.28534 |
| REACTOME_ACTIVATED_NTRK3_SIGNALS_THROUGH_PI3K | -0.18786 | 0.034503 | -1.85625 | 0.067893 | 0.430347 | -4.28608 |
| WP_ENERGY_METABOLISM | -0.10939 | -0.00521 | -1.85383 | 0.068243 | 0.432136 | -4.29004 |
| WHITFIELD_CELL_CYCLE_S | -0.10016 | -0.00986 | -1.85302 | 0.06836 | 0.432292 | -4.29136 |
| CHANG_IMMORTALIZED_BY_HPV31_DN | 0.063213 | -0.0344 | 1.852725 | 0.068403 | 0.432292 | -4.29185 |
| REACTOME_TRANSCRIPTION_COUPLED_NUCLEOTIDE_EXCISION_REPAIR_TC_NER | -0.13014 | -0.10749 | -1.85204 | 0.068503 | 0.432413 | -4.29297 |
| BUYTAERT_PHOTODYNAMIC_THERAPY_STRESS_DN | -0.0853 | -0.13209 | -1.85128 | 0.068613 | 0.432413 | -4.29421 |
| RODWELL_AGING_KIDNEY_NO_BLOOD_DN | -0.09536 | -0.04066 | -1.85017 | 0.068776 | 0.432413 | -4.29603 |
| MIKKELSEN_NPC_ICP_WITH_H3K4ME3 | -0.05233 | -0.13123 | -1.85005 | 0.068794 | 0.432413 | -4.29623 |
| WP_TRANSCRIPTION_FACTORS_REGULATE_MIRNAS_RELATED_TO_CARDIAC_HYPERTROPHY | 0.100769 | -0.0081 | 1.849636 | 0.068854 | 0.432413 | -4.29689 |
| ZHAN_MULTIPLE_MYELOMA_SUBGROUPS | -0.12187 | -0.16796 | -1.84941 | 0.068887 | 0.432413 | -4.29726 |
| SCIAN_INVERSED_TARGETS_OF_TP53_AND_TP73_DN | 0.14101 | -0.02053 | 1.849334 | 0.068898 | 0.432413 | -4.29739 |
| KEGG_PROGESTERONE_MEDIATED_OOCYTE_MATURATION | -0.07477 | -0.0519 | -1.84713 | 0.069221 | 0.433988 | -4.30099 |
| BOYAULT_LIVER_CANCER_SUBCLASS_G12_UP | -0.11805 | -0.08086 | -1.84659 | 0.0693 | 0.433988 | -4.30186 |
| REACTOME_GDP_FUCOSE_BIOSYNTHESIS | -0.1743 | -0.00317 | -1.8457 | 0.069432 | 0.433988 | -4.30332 |
| KEGG_CHEMOKINE_SIGNALING_PATHWAY | 0.063401 | -0.10223 | 1.845575 | 0.06945 | 0.433988 | -4.30352 |
| ZHAN_MULTIPLE_MYELOMA_SPIKED | 0.078566 | -0.26061 | 1.845303 | 0.06949 | 0.433988 | -4.30396 |
| REACTOME_INSULIN_RECEPTOR_SIGNALLING_CASCADE | -0.06888 | -0.00682 | -1.84463 | 0.06959 | 0.434184 | -4.30506 |
| LEIN_MIDBRAIN_MARKERS | 0.068844 | -0.03281 | 1.843349 | 0.069778 | 0.434649 | -4.30714 |
| REACTOME_TCF_DEPENDENT_SIGNALING_IN_RESPONSE_TO_WNT | -0.07268 | -0.1461 | -1.8432 | 0.069801 | 0.434649 | -4.30739 |
| ZHOU_INFLAMMATORY_RESPONSE_LIVE_DN | -0.0745 | -0.09575 | -1.84196 | 0.069985 | 0.435369 | -4.30941 |
| REACTOME_REGULATION_OF_KIT_SIGNALING | 0.139041 | -0.01697 | 1.840871 | 0.070146 | 0.435945 | -4.31118 |
| REACTOME_METABOLISM_OF_ANGIOTENSINOGEN_TO_ANGIOTENSINS | 0.123468 | -0.04621 | 1.840088 | 0.070262 | 0.436241 | -4.31245 |
| PETRETTO_HEART_MASS_QTL_CIS_UP | -0.11479 | -0.25235 | -1.83954 | 0.070343 | 0.436242 | -4.31333 |
| WP_ANTIVIRAL_AND_ANTIINFLAMMATORY_EFFECTS_OF_NRF2_ON_SARSCOV2_PATHWAY | 0.090076 | -0.2192 | 1.839167 | 0.070399 | 0.436242 | -4.31394 |
| BIOCARTA_SALMONELLA_PATHWAY | -0.17911 | 0.003939 | -1.83752 | 0.070645 | 0.4371 | -4.31661 |
| REACTOME_FATTY_ACIDS_BOUND_TO_GPR40_FFAR1_REGULATE_INSULIN_SECRETION | 0.110611 | 0.006282 | 1.83719 | 0.070694 | 0.4371 | -4.31715 |
| REACTOME_EGFR_INTERACTS_WITH_PHOSPHOLIPASE_C_GAMMA | 0.142227 | 0.001134 | 1.83615 | 0.07085 | 0.4371 | -4.31884 |
| REACTOME_O_LINKED_GLYCOSYLATION | 0.070545 | -0.06892 | 1.83557 | 0.070937 | 0.4371 | -4.31978 |
| SASAI_TARGETS_OF_CXCR6_AND_PTCH1_UP | 0.114902 | 0.010913 | 1.835436 | 0.070957 | 0.4371 | -4.32 |
| WANG_THOC1_TARGETS_DN | 0.098904 | -0.02709 | 1.835007 | 0.071021 | 0.4371 | -4.32069 |
| WP_COHESIN_COMPLEX_CORNELIA_DE_LANGE_SYNDROME | -0.11898 | -0.03645 | -1.83498 | 0.071026 | 0.4371 | -4.32074 |
| REACTOME_TRANSPORT_OF_CONNEXONS_TO_THE_PLASMA_MEMBRANE | 0.108769 | -0.01522 | 1.834517 | 0.071095 | 0.4371 | -4.32149 |
| MODY_HIPPOCAMPUS_POSTNATAL | 0.089865 | -0.00873 | 1.83411 | 0.071156 | 0.4371 | -4.32215 |
| PID_IL8_CXCR2_PATHWAY | 0.130153 | 0.003894 | 1.833319 | 0.071275 | 0.437133 | -4.32343 |
| OUILLETTE_CLL_13Q14_DELETION_UP | -0.09652 | -0.01684 | -1.83288 | 0.071341 | 0.437133 | -4.32414 |
| REACTOME_HIV_TRANSCRIPTION_ELONGATION | -0.11226 | -0.25831 | -1.83226 | 0.071434 | 0.437133 | -4.32514 |
| GAUSSMANN_MLL_AF4_FUSION_TARGETS_G_DN | -0.0875 | -0.10421 | -1.83186 | 0.071494 | 0.437133 | -4.32578 |
| REACTOME_ACTIVATED_NTRK2_SIGNALS_THROUGH_FRS2_AND_FRS3 | -0.11859 | -0.06536 | -1.8318 | 0.071505 | 0.437133 | -4.3259 |
| AMIT_EGF_RESPONSE_60_HELA | 0.140927 | -0.02599 | 1.83033 | 0.071726 | 0.437298 | -4.32827 |
| ZHAN_MULTIPLE_MYELOMA_CD1_AND_CD2_UP | -0.10887 | -0.03817 | -1.82999 | 0.071778 | 0.437298 | -4.32882 |
| WONG_MITOCHONDRIA_GENE_MODULE | -0.12497 | -0.11282 | -1.82983 | 0.071801 | 0.437298 | -4.32907 |
| MOREAUX_MULTIPLE_MYELOMA_BY_TACI_UP | 0.043638 | -0.20586 | 1.8293 | 0.071882 | 0.437298 | -4.32993 |
| KEGG_BLADDER_CANCER | 0.098684 | -0.06261 | 1.82902 | 0.071925 | 0.437298 | -4.33038 |
| TCGA_GLIOBLASTOMA_COPY_NUMBER_DN | -0.09968 | -0.07195 | -1.82872 | 0.071969 | 0.437298 | -4.33086 |
| ROVERSI_GLIOMA_COPY_NUMBER_UP | 0.050732 | -0.05172 | 1.82844 | 0.072013 | 0.437298 | -4.33132 |
| WP_INFLAMMATORY_RESPONSE_PATHWAY | 0.106317 | -0.01256 | 1.827802 | 0.07211 | 0.437469 | -4.33235 |
| REACTOME_EICOSANOIDS | 0.142295 | 0.013976 | 1.827267 | 0.072191 | 0.437546 | -4.33322 |
| RODWELL_AGING_KIDNEY_DN | -0.07169 | -0.04073 | -1.8261 | 0.072368 | 0.438201 | -4.33509 |
| WP_GPR143_IN_MELANOCYTES_AND_RETINAL_PIGMENT_EPITHELIUM_CELLS | 0.065882 | -0.09235 | 1.825414 | 0.072473 | 0.438273 | -4.33621 |
| PID_HNF3B_PATHWAY | 0.07485 | -0.05823 | 1.825023 | 0.072533 | 0.438273 | -4.33684 |
| BIOCARTA_EOSINOPHILS_PATHWAY | 0.0533 | -0.60929 | 1.824674 | 0.072586 | 0.438273 | -4.3374 |
| RAMALHO_STEMNESS_DN | 0.073351 | -0.22249 | 1.823673 | 0.072739 | 0.438781 | -4.33902 |
| MOOTHA_GLYCOLYSIS | 0.114296 | -0.05142 | 1.822486 | 0.072921 | 0.439463 | -4.34093 |
| THUM_SYSTOLIC_HEART_FAILURE_DN | -0.07399 | -0.10543 | -1.82182 | 0.073024 | 0.439663 | -4.342 |
| WP_NUCLEOTIDE_EXCISION_REPAIR_IN_XERODERMA_PIGMENTOSUM | -0.10683 | -0.14207 | -1.81892 | 0.073471 | 0.441567 | -4.34668 |
| WP_HEDGEHOG_SIGNALING_PATHWAY_WP47 | -0.13761 | 0.009452 | -1.81886 | 0.073479 | 0.441567 | -4.34676 |
| REACTOME_HDR_THROUGH_HOMOLOGOUS_RECOMBINATION_HRR | -0.09455 | -0.03767 | -1.81813 | 0.073592 | 0.44183 | -4.34793 |
| REACTOME_SCAVENGING_BY_CLASS_F_RECEPTORS | 0.136575 | -0.24311 | 1.816806 | 0.073797 | 0.442634 | -4.35007 |
| LIU_IL13_PRIMING_MODEL | 0.1021 | -0.05979 | 1.816369 | 0.073865 | 0.442634 | -4.35077 |
| IVANOVA_HEMATOPOIESIS_INTERMEDIATE_PROGENITOR | -0.12407 | -0.0698 | -1.8148 | 0.074109 | 0.443231 | -4.35329 |
| REACTOME_SIGNALING_BY_FGFR2_IN_DISEASE | -0.0779 | -0.02309 | -1.81471 | 0.074123 | 0.443231 | -4.35343 |
| HOWLIN_CITED1_TARGETS_1_UP | 0.062566 | -0.30279 | 1.814321 | 0.074183 | 0.443231 | -4.35406 |
| WINNEPENNINCKX_MELANOMA_METASTASIS_DN | 0.069417 | -0.271 | 1.813366 | 0.074332 | 0.443231 | -4.35559 |
| MANALO_HYPOXIA_DN | -0.11843 | -0.03661 | -1.81331 | 0.074341 | 0.443231 | -4.35568 |
| BROWNE_HCMV_INFECTION_10HR_DN | -0.10282 | -0.05106 | -1.81304 | 0.074383 | 0.443231 | -4.35611 |
| YAO_TEMPORAL_RESPONSE_TO_PROGESTERONE_CLUSTER_17 | -0.1255 | -0.13645 | -1.81173 | 0.074587 | 0.443742 | -4.35821 |
| WINTER_HYPOXIA_METAGENE | 0.104246 | -0.07872 | 1.811601 | 0.074608 | 0.443742 | -4.35842 |
| REACTOME_KERATINIZATION | 0.129571 | -0.11653 | 1.809692 | 0.074907 | 0.444544 | -4.36148 |
| REACTOME_ACTIVATED_TAK1_MEDIATES_P38_MAPK_ACTIVATION | 0.108402 | 0.005045 | 1.809385 | 0.074955 | 0.444544 | -4.36197 |
| WP_DOPAMINERGIC_NEUROGENESIS | 0.128606 | -0.05256 | 1.808575 | 0.075082 | 0.444544 | -4.36326 |
| LI_WILMS_TUMOR_VS_FETAL_KIDNEY_1_DN | -0.11009 | -0.0699 | -1.80828 | 0.075129 | 0.444544 | -4.36374 |
| REACTOME_MET_INTERACTS_WITH_TNS_PROTEINS | 0.151582 | 2.82E-05 | 1.808123 | 0.075154 | 0.444544 | -4.36399 |
| VANTVEER_BREAST_CANCER_POOR_PROGNOSIS | -0.09207 | -0.05214 | -1.80802 | 0.075169 | 0.444544 | -4.36415 |
| REACTOME_CONDENSATION_OF_PROPHASE_CHROMOSOMES | -0.1331 | -0.09043 | -1.80763 | 0.075232 | 0.444544 | -4.36478 |
| DEBOSSCHER_NFKB_TARGETS_REPRESSED_BY_GLUCOCORTICOIDS | 0.077591 | -0.27239 | 1.807111 | 0.075313 | 0.444612 | -4.36561 |
| WALLACE_PROSTATE_CANCER_UP | 0.095718 | -0.05008 | 1.806161 | 0.075463 | 0.445083 | -4.36712 |
| REACTOME_AMINO_ACID_CONJUGATION | 0.190104 | 0.025908 | 1.805423 | 0.075579 | 0.445227 | -4.3683 |
| CAFFAREL_RESPONSE_TO_THC_8HR_3_UP | 0.170188 | 3.75E-05 | 1.805122 | 0.075627 | 0.445227 | -4.36878 |
| REACTOME_ACROSOME_REACTION_AND_SPERM_OOCYTE_MEMBRANE_BINDING | 0.173759 | -0.13771 | 1.804005 | 0.075804 | 0.445506 | -4.37057 |
| TESAR_ALK_TARGETS_EPISC_3D_UP | 0.170103 | 0.008529 | 1.802661 | 0.076017 | 0.445506 | -4.37271 |
| MEBARKI_HCC_PROGENITOR_FZD8CRD_DN | 0.05877 | -0.1228 | 1.802531 | 0.076038 | 0.445506 | -4.37292 |
| KEGG_METABOLISM_OF_XENOBIOTICS_BY_CYTOCHROME_P450 | 0.082338 | -0.05188 | 1.802246 | 0.076083 | 0.445506 | -4.37337 |
| REACTOME_NETRIN_1_SIGNALING | -0.08636 | -0.00774 | -1.80218 | 0.076094 | 0.445506 | -4.37349 |
| REACTOME_BMAL1_CLOCK_NPAS2_ACTIVATES_CIRCADIAN_GENE_EXPRESSION | -0.10749 | -0.01055 | -1.80217 | 0.076095 | 0.445506 | -4.37349 |
| SIMBULAN_UV_RESPONSE_NORMAL_DN | 0.125244 | -0.05439 | 1.801512 | 0.0762 | 0.445713 | -4.37455 |
| REACTOME_FANCONI_ANEMIA_PATHWAY | -0.10738 | -0.02928 | -1.80092 | 0.076294 | 0.445853 | -4.37549 |
| REACTOME_DISEASES_OF_MITOTIC_CELL_CYCLE | -0.10844 | -0.104 | -1.79995 | 0.076448 | 0.446346 | -4.37703 |
| WP_16P112_DISTAL_DELETION_SYNDROME | 0.076067 | -0.02996 | 1.798479 | 0.076684 | 0.44731 | -4.37938 |
| WP_PREIMPLANTATION_EMBRYO | 0.086735 | -0.10175 | 1.798042 | 0.076754 | 0.44731 | -4.38007 |
| REACTOME_BETA_DEFENSINS | 0.083654 | -0.29327 | 1.796725 | 0.076965 | 0.448062 | -4.38217 |
| REACTOME_TICAM1_RIP1_MEDIATED_IKK_COMPLEX_RECRUITMENT | 0.128932 | -0.04305 | 1.79526 | 0.077201 | 0.448062 | -4.3845 |
| WP_FGF23_SIGNALING_IN_HYPOPHOSPHATEMIC_RICKETS_AND_RELATED_DISORDERS | 0.091389 | -0.11468 | 1.795217 | 0.077208 | 0.448062 | -4.38457 |
| CERIBELLI_GENES_INACTIVE_AND_BOUND_BY_NFY | 0.100081 | 0.004169 | 1.794572 | 0.077311 | 0.448062 | -4.38559 |
| REACTOME_SIGNALING_BY_FGFR3_FUSIONS_IN_CANCER | -0.124 | -0.05608 | -1.7943 | 0.077356 | 0.448062 | -4.38603 |
| REACTOME_PASSIVE_TRANSPORT_BY_AQUAPORINS | 0.1406 | 0.021262 | 1.794182 | 0.077374 | 0.448062 | -4.38621 |
| IVANOVA_HEMATOPOIESIS_EARLY_PROGENITOR | -0.07587 | -0.10325 | -1.79403 | 0.077399 | 0.448062 | -4.38646 |
| HAMAI_APOPTOSIS_VIA_TRAIL_UP | -0.11976 | -0.05355 | -1.79373 | 0.077446 | 0.448062 | -4.38692 |
| WP_FATTY_ACID_OMEGAOXIDATION | 0.093822 | -0.22685 | 1.792499 | 0.077646 | 0.448809 | -4.38888 |
| BRUNEAU_SEPTATION_ATRIAL | 0.183097 | -0.1163 | 1.791926 | 0.077739 | 0.448937 | -4.38979 |
| BIOCARTA_MITOCHONDRIA_PATHWAY | -0.12255 | 0.006886 | -1.7889 | 0.078229 | 0.451361 | -4.39459 |
| WP_GPCRS_CLASS_A_RHODOPSINLIKE | 0.071187 | -0.25019 | 1.788107 | 0.078359 | 0.45157 | -4.39585 |
| BERENJENO_TRANSFORMED_BY_RHOA_FOREVER_UP | 0.161919 | 0.006677 | 1.787809 | 0.078407 | 0.45157 | -4.39632 |
| REACTOME_DNA_REPLICATION | -0.10984 | -0.13009 | -1.78481 | 0.078898 | 0.452995 | -4.40107 |
| REACTOME_CELL_CYCLE_MITOTIC | -0.09511 | -0.09544 | -1.78459 | 0.078933 | 0.452995 | -4.40141 |
| KEGG_PEROXISOME | -0.07819 | -0.09619 | -1.78451 | 0.078947 | 0.452995 | -4.40155 |
| KIM_LIVER_CANCER_POOR_SURVIVAL_DN | 0.064867 | -0.06109 | 1.783471 | 0.079117 | 0.452995 | -4.40318 |
| REACTOME_SYNTHESIS_OF_WYBUTOSINE_AT_G37_OF_TRNA_PHE | -0.15693 | -0.00638 | -1.7832 | 0.079162 | 0.452995 | -4.40361 |
| XU_HGF_SIGNALING_NOT_VIA_AKT1_48HR_UP | 0.096575 | -0.20891 | 1.783082 | 0.079181 | 0.452995 | -4.4038 |
| BIOCARTA_CACAM_PATHWAY | -0.12596 | 0.014929 | -1.78301 | 0.079193 | 0.452995 | -4.40391 |
| REACTOME_IMMUNOREGULATORY_INTERACTIONS_BETWEEN_A_LYMPHOID_AND_A_NON_LYMPHOID_CELL | 0.035009 | -0.54361 | 1.78282 | 0.079224 | 0.452995 | -4.40421 |
| DELLA_RESPONSE_TO_TSA_AND_BUTYRATE | 0.072749 | -0.33962 | 1.782382 | 0.079296 | 0.453 | -4.4049 |
| PEDERSEN_TARGETS_OF_611CTF_ISOFORM_OF_ERBB2 | 0.093878 | -0.05924 | 1.781739 | 0.079402 | 0.453198 | -4.40592 |
| REACTOME_FORMATION_OF_THE_CORNIFIED_ENVELOPE | 0.128763 | -0.06776 | 1.779875 | 0.07971 | 0.454546 | -4.40886 |
| REACTOME_RESPONSE_TO_ELEVATED_PLATELET_CYTOSOLIC_CA2 | 0.075942 | -0.0751 | 1.777808 | 0.080052 | 0.455851 | -4.41212 |
| MAINA_VHL_TARGETS_DN | 0.122619 | -0.07339 | 1.777585 | 0.080089 | 0.455851 | -4.41247 |
| WP_ELECTRON_TRANSPORT_CHAIN_OXPHOS_SYSTEM_IN_MITOCHONDRIA | -0.12433 | -0.15531 | -1.77653 | 0.080264 | 0.455851 | -4.41413 |
| LY_AGING_MIDDLE_UP | 0.165036 | -0.00749 | 1.776199 | 0.080319 | 0.455851 | -4.41465 |
| REACTOME_THE_CITRIC_ACID_TCA_CYCLE_AND_RESPIRATORY_ELECTRON_TRANSPORT | -0.12202 | -0.09117 | -1.7754 | 0.080452 | 0.455851 | -4.41591 |
| MIDORIKAWA_AMPLIFIED_IN_LIVER_CANCER | -0.04816 | -0.27185 | -1.77516 | 0.080492 | 0.455851 | -4.41628 |
| BLANCO_MELO_HUMAN_PARAINFLUENZA_VIRUS_3_INFECTION_A594_CELLS_UP | 0.084966 | -0.16742 | 1.774774 | 0.080557 | 0.455851 | -4.41689 |
| MACLACHLAN_BRCA1_TARGETS_UP | 0.126001 | 0.002689 | 1.774666 | 0.080575 | 0.455851 | -4.41706 |
| REACTOME_O_GLYCOSYLATION_OF_TSR_DOMAIN_CONTAINING_PROTEINS | 0.099746 | -0.04372 | 1.774613 | 0.080583 | 0.455851 | -4.41715 |
| KYNG_DNA_DAMAGE_BY_4NQO | -0.13462 | -0.05283 | -1.77416 | 0.08066 | 0.455878 | -4.41787 |
| WP_HIPPOYAP_SIGNALING_PATHWAY | -0.09568 | -0.03823 | -1.77306 | 0.080842 | 0.456053 | -4.41958 |
| REACTOME_ACTIVATION_OF_ANTERIOR_HOX_GENES_IN_HINDBRAIN_DEVELOPMENT_DURING_EARLY_EMBRYOGENESIS | -0.09561 | -0.02404 | -1.77304 | 0.080846 | 0.456053 | -4.41962 |
| SHIN_B_CELL_LYMPHOMA_CLUSTER_2 | 0.091413 | -0.06517 | 1.772684 | 0.080906 | 0.456053 | -4.42018 |
| REACTOME_RECEPTOR_MEDIATED_MITOPHAGY | -0.14291 | -0.31202 | -1.77172 | 0.081067 | 0.456137 | -4.42169 |
| BLANCO_MELO_COVID19_SARS_COV_2_INFECTION_A594_ACE2_EXPRESSING_CELLS_UP | 0.058251 | -0.14801 | 1.771457 | 0.081111 | 0.456137 | -4.42211 |
| BIOCARTA_TNFR1_PATHWAY | -0.11606 | -0.17719 | -1.77103 | 0.081184 | 0.456137 | -4.42279 |
| REACTOME_REGULATION_OF_PTEN_STABILITY_AND_ACTIVITY | -0.09022 | -0.29328 | -1.77088 | 0.081207 | 0.456137 | -4.42301 |
| BOYAULT_LIVER_CANCER_SUBCLASS_G56_UP | 0.094419 | -0.00824 | 1.769829 | 0.081385 | 0.45673 | -4.42466 |
| REACTOME_RRNA_MODIFICATION_IN_THE_NUCLEUS_AND_CYTOSOL | -0.11307 | -0.21542 | -1.76935 | 0.081466 | 0.456782 | -4.42542 |
| GERHOLD_ADIPOGENESIS_UP | 0.1293 | -0.05862 | 1.768485 | 0.081611 | 0.457193 | -4.42677 |
| FOSTER_TOLERANT_MACROPHAGE_UP | 0.072671 | -0.12002 | 1.76685 | 0.081887 | 0.457942 | -4.42934 |
| GAJATE_RESPONSE_TO_TRABECTEDIN_DN | -0.12875 | -0.05674 | -1.76684 | 0.081889 | 0.457942 | -4.42935 |
| MOOTHA_MITOCHONDRIA | -0.10705 | -0.08167 | -1.76624 | 0.08199 | 0.458107 | -4.43029 |
| WP_CATALYTIC_CYCLE_OF_MAMMALIAN_FLAVINCONTAINING_MONOOXYGENASES_FMOS | -0.19749 | 0.017651 | -1.76491 | 0.082214 | 0.458883 | -4.43237 |
| REACTOME_CONSTITUTIVE_SIGNALING_BY_OVEREXPRESSED_ERBB2 | -0.14716 | -0.0606 | -1.76457 | 0.082273 | 0.458883 | -4.43291 |
| REACTOME_SYNTHESIS_OF_PG | 0.125967 | -0.00104 | 1.763469 | 0.08246 | 0.459523 | -4.43463 |
| REACTOME_DOWNSTREAM_SIGNALING_OF_ACTIVATED_FGFR1 | -0.0874 | -0.02733 | -1.76247 | 0.082629 | 0.459694 | -4.43618 |
| GEORGES_TARGETS_OF_MIR192_AND_MIR215 | -0.07713 | -0.08364 | -1.76169 | 0.082763 | 0.459694 | -4.43741 |
| EBAUER_TARGETS_OF_PAX3_FOXO1_FUSION_UP | 0.051485 | -0.06004 | 1.761512 | 0.082793 | 0.459694 | -4.43769 |
| HENDRICKS_SMARCA4_TARGETS_DN | 0.080603 | -0.17864 | 1.761443 | 0.082805 | 0.459694 | -4.4378 |
| BENPORATH_EED_TARGETS | 0.064608 | -0.09454 | 1.761168 | 0.082852 | 0.459694 | -4.43823 |
| REACTOME_MITOTIC_METAPHASE_AND_ANAPHASE | -0.10475 | -0.09806 | -1.7599 | 0.083069 | 0.46034 | -4.44021 |
| JAIN_NFKB_SIGNALING | -0.08567 | -0.22015 | -1.75957 | 0.083125 | 0.46034 | -4.44072 |
| GAUSSMANN_MLL_AF4_FUSION_TARGETS_A_DN | 0.051589 | -0.20907 | 1.759218 | 0.083185 | 0.46034 | -4.44127 |
| REACTOME_DEFECTIVE_INTRINSIC_PATHWAY_FOR_APOPTOSIS | 0.081164 | -0.04145 | 1.758539 | 0.083302 | 0.460583 | -4.44233 |
| MOOTHA_HUMAN_MITODB_6_2002 | -0.10567 | -0.09559 | -1.757 | 0.083566 | 0.461405 | -4.44474 |
| DORN_ADENOVIRUS_INFECTION_24HR_UP | 0.131269 | -0.08637 | 1.756107 | 0.083719 | 0.461405 | -4.44612 |
| LIU_CDX2_TARGETS_UP | 0.059942 | -0.28231 | 1.755937 | 0.083749 | 0.461405 | -4.44639 |
| REACTOME_COHESIN_LOADING_ONTO_CHROMATIN | -0.20757 | -0.0126 | -1.75587 | 0.083761 | 0.461405 | -4.4465 |
| WP_AEROBIC_GLYCOLYSIS | 0.143404 | -0.13381 | 1.755206 | 0.083875 | 0.461405 | -4.44753 |
| GAL_LEUKEMIC_STEM_CELL_UP | -0.04845 | -0.11509 | -1.7551 | 0.083893 | 0.461405 | -4.44769 |
| WP_SOMATROPH_AXIS_GH_AND_ITS_RELATIONSHIP_TO_DIETARY_RESTRICTION_AND_AGING | -0.15757 | -0.1583 | -1.75472 | 0.083958 | 0.461405 | -4.44828 |
| MATZUK_POSTIMPLANTATION_AND_POSTPARTUM | 0.08945 | -0.07674 | 1.752037 | 0.084422 | 0.463439 | -4.45246 |
| SANCHEZ_MDM2_TARGETS | 0.159099 | 0.013771 | 1.751742 | 0.084473 | 0.463439 | -4.45292 |
| HADDAD_T_LYMPHOCYTE_AND_NK_PROGENITOR_DN | 0.064634 | -0.27314 | 1.750406 | 0.084706 | 0.464312 | -4.45499 |
| WP_IL10_ANTIINFLAMMATORY_SIGNALING_PATHWAY | 0.156606 | -0.00093 | 1.749431 | 0.084875 | 0.464841 | -4.45651 |
| MATZUK_EARLY_ANTRAL_FOLLICLE | -0.10084 | -0.00041 | -1.74898 | 0.084954 | 0.464871 | -4.45721 |
| REACTOME_CHROMATIN_MODIFYING_ENZYMES | -0.09383 | -0.07721 | -1.74845 | 0.085045 | 0.464972 | -4.45802 |
| REACTOME_SODIUM_COUPLED_SULPHATE_DI_AND_TRI_CARBOXYLATE_TRANSPORTERS | 0.198566 | 0.026374 | 1.747892 | 0.085143 | 0.46511 | -4.4589 |
| PID_SYNDECAN_1_PATHWAY | 0.095675 | -0.15775 | 1.746222 | 0.085435 | 0.466305 | -4.46149 |
| BIOCARTA_LIS1_PATHWAY | -0.10159 | -0.00593 | -1.74574 | 0.08552 | 0.466367 | -4.46224 |
| KEGG_GLYCOSPHINGOLIPID_BIOSYNTHESIS_GLOBO_SERIES | 0.11443 | -0.01611 | 1.744916 | 0.085664 | 0.466564 | -4.46351 |
| WP_GABA_RECEPTOR_SIGNALING | 0.102729 | -0.1888 | 1.744479 | 0.085741 | 0.466564 | -4.46419 |
| WP_AFLATOXIN_B1_METABOLISM | -0.10555 | -0.01395 | -1.74428 | 0.085776 | 0.466564 | -4.4645 |
| REACTOME_HOMOLOGY_DIRECTED_REPAIR | -0.08074 | -0.09851 | -1.74355 | 0.085905 | 0.466733 | -4.46563 |
| FERRANDO_T_ALL_WITH_MLL_ENL_FUSION_UP | 0.074951 | -0.23185 | 1.743267 | 0.085954 | 0.466733 | -4.46606 |
| REACTOME_INTERLEUKIN_18_SIGNALING | 0.131663 | -0.11108 | 1.741933 | 0.086189 | 0.46761 | -4.46813 |
| REN_BOUND_BY_E2F | -0.11116 | -0.13735 | -1.74035 | 0.086468 | 0.468722 | -4.47057 |
| MARKEY_RB1_ACUTE_LOF_UP | -0.10489 | -0.06523 | -1.73864 | 0.086771 | 0.469314 | -4.47321 |
| REACTOME_INTERCONVERSION_OF_NUCLEOTIDE_DI_AND_TRIPHOSPHATES | -0.12431 | -0.0346 | -1.73784 | 0.086914 | 0.469314 | -4.47446 |
| GENTILE_UV_HIGH_DOSE_UP | 0.121673 | -0.00054 | 1.737745 | 0.08693 | 0.469314 | -4.4746 |
| WP_OXIDATIVE_STRESS_RESPONSE | 0.100494 | -0.04351 | 1.737258 | 0.087017 | 0.469314 | -4.47535 |
| OKUMURA_INFLAMMATORY_RESPONSE_LPS | 0.062262 | -0.12316 | 1.736956 | 0.08707 | 0.469314 | -4.47582 |
| FARMER_BREAST_CANCER_CLUSTER_2 | -0.1489 | -0.05092 | -1.7367 | 0.087117 | 0.469314 | -4.47622 |
| REACTOME_ACTIVATED_NTRK2_SIGNALS_THROUGH_CDK5 | 0.126909 | 0.00068 | 1.73628 | 0.087191 | 0.469314 | -4.47686 |
| REACTOME_TGF_BETA_RECEPTOR_SIGNALING_IN_EMT_EPITHELIAL_TO_MESENCHYMAL_TRANSITION | 0.122115 | -0.07615 | 1.735911 | 0.087256 | 0.469314 | -4.47743 |
| VETTER_TARGETS_OF_PRKCA_AND_ETS1_DN | 0.07281 | -0.29281 | 1.735687 | 0.087296 | 0.469314 | -4.47777 |
| VANHARANTA_UTERINE_FIBROID_WITH_7Q_DELETION_UP | -0.11152 | -0.07519 | -1.73559 | 0.087314 | 0.469314 | -4.47793 |
| REACTOME_COMMON_PATHWAY_OF_FIBRIN_CLOT_FORMATION | 0.098288 | -0.0531 | 1.73462 | 0.087486 | 0.469694 | -4.47942 |
| GRAESSMANN_RESPONSE_TO_MC_AND_DOXORUBICIN_DN | -0.09027 | -0.11913 | -1.73436 | 0.087532 | 0.469694 | -4.47982 |
| WANG_LSD1_TARGETS_UP | 0.097231 | -0.0453 | 1.732225 | 0.087915 | 0.470254 | -4.48311 |
| KINSEY_TARGETS_OF_EWSR1_FLII_FUSION_UP | -0.09664 | -0.07193 | -1.73222 | 0.087915 | 0.470254 | -4.48311 |
| REACTOME_DNA_STRAND_ELONGATION | -0.14229 | -0.0028 | -1.73199 | 0.087957 | 0.470254 | -4.48347 |
| GAURNIER_PSMD4_TARGETS | 0.033233 | -0.645 | 1.731888 | 0.087975 | 0.470254 | -4.48363 |
| URS_ADIPOCYTE_DIFFERENTIATION_DN | 0.121565 | -0.00681 | 1.731227 | 0.088094 | 0.470254 | -4.48464 |
| AMIT_SERUM_RESPONSE_60_MCF10A | 0.126336 | -0.01069 | 1.730951 | 0.088144 | 0.470254 | -4.48507 |
| QI_PLASMACYTOMA_UP | 0.075207 | -0.19378 | 1.730892 | 0.088154 | 0.470254 | -4.48516 |
| WP_NUCLEAR_RECEPTORS_IN_LIPID_METABOLISM_AND_TOXICITY | 0.102704 | -0.03886 | 1.730064 | 0.088303 | 0.470654 | -4.48643 |
| REACTOME_FORMATION_OF_INCISION_COMPLEX_IN_GG_NER | -0.11467 | -0.1746 | -1.72899 | 0.088497 | 0.471294 | -4.48809 |
| REACTOME_RESOLUTION_OF_D_LOOP_STRUCTURES | -0.09602 | -0.02113 | -1.72741 | 0.088782 | 0.472282 | -4.49051 |
| REACTOME_REGULATION_OF_RUNX1_EXPRESSION_AND_ACTIVITY | -0.12263 | -0.04951 | -1.72666 | 0.088918 | 0.472282 | -4.49167 |
| TONKS_TARGETS_OF_RUNX1_RUNX1T1_FUSION_MONOCYTE_DN | 0.069017 | -0.21824 | 1.725864 | 0.089061 | 0.472282 | -4.49288 |
| NADLER_HYPERGLYCEMIA_AT_OBESITY | 0.063898 | -0.16725 | 1.725261 | 0.08917 | 0.472282 | -4.4938 |
| MIKKELSEN_NPC_HCP_WITH_H3K4ME3_AND_H3K27ME3 | 0.078223 | -0.0639 | 1.725204 | 0.089181 | 0.472282 | -4.49389 |
| KEGG_ECM_RECEPTOR_INTERACTION | 0.073694 | -0.20371 | 1.725048 | 0.089209 | 0.472282 | -4.49413 |
| MCBRYAN_PUBERTAL_BREAST_4_5WK_UP | 0.059312 | -0.09955 | 1.72471 | 0.08927 | 0.472282 | -4.49465 |
| DAVIES_MULTIPLE_MYELOMA_VS_MGUS_DN | 0.137481 | -0.05182 | 1.724678 | 0.089276 | 0.472282 | -4.4947 |
| SMID_BREAST_CANCER_LUMINAL_B_DN | 0.05945 | -0.14296 | 1.723519 | 0.089487 | 0.473002 | -4.49648 |
| REACTOME_CITRIC_ACID_CYCLE_TCA_CYCLE | -0.17532 | 0.006831 | -1.72265 | 0.089646 | 0.473157 | -4.49781 |
| RIZ_ERYTHROID_DIFFERENTIATION | -0.07567 | -0.16221 | -1.72232 | 0.089705 | 0.473157 | -4.49831 |
| REACTOME_CDC6_ASSOCIATION_WITH_THE_ORC_ORIGIN_COMPLEX | -0.09469 | -0.0686 | -1.72172 | 0.089815 | 0.473157 | -4.49923 |
| JI_CARCINOGENESIS_BY_KRAS_AND_STK11_UP | 0.142836 | 0.01291 | 1.720512 | 0.090035 | 0.473157 | -4.50108 |
| KEGG_HOMOLOGOUS_RECOMBINATION | -0.09253 | -0.07829 | -1.7204 | 0.090056 | 0.473157 | -4.50126 |
| REACTOME_OVARIAN_TUMOR_DOMAIN_PROTEASES | 0.085219 | -0.02473 | 1.720283 | 0.090077 | 0.473157 | -4.50143 |
| WU_CELL_MIGRATION | 0.087922 | -0.00506 | 1.720098 | 0.090111 | 0.473157 | -4.50171 |
| NABA_SECRETED_FACTORS | 0.07278 | -0.11386 | 1.72001 | 0.090127 | 0.473157 | -4.50185 |
| REACTOME_TRANSLATION | -0.13598 | -0.1016 | -1.71969 | 0.090185 | 0.473157 | -4.50233 |
| REACTOME_MECP2_REGULATES_NEURONAL_RECEPTORS_AND_CHANNELS | 0.098181 | -0.00568 | 1.718115 | 0.090474 | 0.473906 | -4.50474 |
| PID_IL3_PATHWAY | 0.095383 | -0.03229 | 1.717898 | 0.090514 | 0.473906 | -4.50507 |
| GENTILE_UV_RESPONSE_CLUSTER_D6 | -0.13158 | -0.07492 | -1.71718 | 0.090646 | 0.473906 | -4.50617 |
| SAGIV_CD24_TARGETS_DN | 0.084455 | -0.04224 | 1.716873 | 0.090702 | 0.473906 | -4.50664 |
| REACTOME_REGULATION_OF_MECP2_EXPRESSION_AND_ACTIVITY | -0.08597 | -0.07669 | -1.71647 | 0.090777 | 0.473906 | -4.50726 |
| PANGAS_TUMOR_SUPPRESSION_BY_SMAD1_AND_SMAD5_DN | -0.06265 | -0.11371 | -1.71639 | 0.090792 | 0.473906 | -4.50738 |
| TIMOFEEVA_GROWTH_STRESS_VIA_STAT1_DN | -0.12169 | -0.10676 | -1.71583 | 0.090895 | 0.473906 | -4.50824 |
| PID_RAC1_REG_PATHWAY | 0.07198 | -0.12294 | 1.715671 | 0.090924 | 0.473906 | -4.50847 |
| REACTOME_ANCHORING_OF_THE_BASAL_BODY_TO_THE_PLASMA_MEMBRANE | -0.07328 | -0.11541 | -1.71481 | 0.091083 | 0.474345 | -4.50979 |
| DEMAGALHAES_AGING_UP | 0.101102 | -0.18387 | 1.714299 | 0.091177 | 0.474448 | -4.51057 |
| FOROUTAN_INTEGRATED_TGFB_EMT_DN | 0.055623 | -0.12664 | 1.713269 | 0.091367 | 0.474858 | -4.51214 |
| PID_BARD1_PATHWAY | -0.09074 | -0.00717 | -1.71286 | 0.091443 | 0.474858 | -4.51276 |
| REACTOME_INTRACELLULAR_SIGNALING_BY_SECOND_MESSENGERS | -0.06317 | -0.12946 | -1.71266 | 0.09148 | 0.474858 | -4.51306 |
| WP_LNCRNAMEDIATED_MECHANISMS_OF_THERAPEUTIC_RESISTANCE | 0.127939 | -0.01777 | 1.712217 | 0.091562 | 0.474899 | -4.51374 |
| SMID_BREAST_CANCER_RELAPSE_IN_BRAIN_UP | 0.061651 | -0.08848 | 1.711299 | 0.091732 | 0.475013 | -4.51514 |
| VERNELL_RETINOBLASTOMA_PATHWAY_UP | -0.09021 | -0.13466 | -1.71101 | 0.091787 | 0.475013 | -4.51558 |
| BIDUS_METASTASIS_UP | -0.113 | -0.03147 | -1.71075 | 0.091834 | 0.475013 | -4.51597 |
| BARRIER_CANCER_RELAPSE_TUMOR_SAMPLE_UP | -0.17459 | -0.01305 | -1.71035 | 0.09191 | 0.475013 | -4.51659 |
| ADDYA_ERYTHROID_DIFFERENTIATION_BY_HEMIN | 0.083888 | -0.15279 | 1.710089 | 0.091957 | 0.475013 | -4.51698 |
| KAAB_FAILED_HEART_ATRIUM_UP | 0.062526 | -0.2403 | 1.708146 | 0.092319 | 0.476382 | -4.51993 |
| YAMANAKA_GLIOBLASTOMA_SURVIVAL_UP | 0.140032 | 0.004209 | 1.706947 | 0.092543 | 0.476382 | -4.52175 |
| BIOCARTA_DSP_PATHWAY | 0.104633 | -0.19439 | 1.706841 | 0.092563 | 0.476382 | -4.52192 |
| MIKKELSEN_MCV6_ICP_WITH_H3K27ME3 | 0.069971 | -0.24232 | 1.706725 | 0.092585 | 0.476382 | -4.52209 |
| WP_NEURODEGENERATION_WITH_BRAIN_IRON_ACCUMULATION_NBIA_SUBTYPES_PATHWAY | -0.09597 | -0.07352 | -1.70617 | 0.092689 | 0.476382 | -4.52294 |
| GRAESSMANN_APOPTOSIS_BY_DOXORUBICIN_DN | -0.09068 | -0.10505 | -1.70599 | 0.092722 | 0.476382 | -4.52321 |
| ONO_FOXP3_TARGETS_UP | 0.096388 | 0.007261 | 1.705672 | 0.092782 | 0.476382 | -4.52369 |
| LUI_THYROID_CANCER_PAX8_PPARG_UP | 0.073685 | -0.13284 | 1.705245 | 0.092862 | 0.476382 | -4.52434 |
| REACTOME_HDACS_DEACETYLATE_HISTONES | -0.12026 | -0.06223 | -1.7049 | 0.092927 | 0.476382 | -4.52487 |
| RODRIGUES_THYROID_CARCINOMA_POORLY_DIFFERENTIATED_UP | -0.11363 | -0.04292 | -1.7045 | 0.093001 | 0.476382 | -4.52547 |
| URS_ADIPOCYTE_DIFFERENTIATION_UP | 0.069523 | -0.18595 | 1.703722 | 0.093148 | 0.476382 | -4.52665 |
| REACTOME_COMPLEMENT_CASCADE | 0.049342 | -0.25432 | 1.703543 | 0.093181 | 0.476382 | -4.52692 |
| REACTOME_INACTIVATION_OF_CDC42_AND_RAC1 | -0.14392 | 0.00493 | -1.70347 | 0.093195 | 0.476382 | -4.52703 |
| LASTOWSKA_NEUROBLASTOMA_COPY_NUMBER_DN | -0.10359 | -0.04152 | -1.70246 | 0.093386 | 0.476972 | -4.52857 |
| CHEN_ETV5_TARGETS_SERTOLI | 0.111038 | -0.04615 | 1.701402 | 0.093585 | 0.477436 | -4.53016 |
| REACTOME_RUNX1_REGULATES_ESTROGEN_RECEPTOR_MEDIATED_TRANSCRIPTION | -0.18155 | -0.01199 | -1.70097 | 0.093667 | 0.477436 | -4.53082 |
| SEMBA_FHIT_TARGETS_UP | 0.13809 | 0.015277 | 1.700122 | 0.093826 | 0.477436 | -4.5321 |
| PETRETTO_LEFT_VENTRICLE_MASS_QTL_CIS_UP | -0.15331 | -0.19837 | -1.69928 | 0.093986 | 0.477436 | -4.53337 |
| REACTOME_DOWNREGULATION_OF_ERBB2_SIGNALING | 0.078293 | -0.04065 | 1.699164 | 0.094008 | 0.477436 | -4.53355 |
| KEGG_LONG_TERM_POTENTIATION | -0.06897 | -0.05439 | -1.69877 | 0.094082 | 0.477436 | -4.53414 |
| REACTOME_ENDOSOMAL_VACUOLAR_PATHWAY | 0.039719 | -0.72792 | 1.698726 | 0.094091 | 0.477436 | -4.53421 |
| WP_DDX1_AS_A_REGULATORY_COMPONENT_OF_THE_DROSHA_MICROPROCESSOR | -0.16177 | -0.02889 | -1.6986 | 0.094114 | 0.477436 | -4.5344 |
| PETROVA_PROX1_TARGETS_UP | -0.07875 | -0.04713 | -1.69797 | 0.094235 | 0.477436 | -4.53536 |
| REACTOME_PKA_ACTIVATION_IN_GLUCAGON_SIGNALLING | -0.08444 | -0.14555 | -1.6973 | 0.094362 | 0.477436 | -4.53637 |
| REACTOME_TP53_REGULATES_TRANSCRIPTION_OF_GENES_INVOLVED_IN_CYTOCHROME_C_RELEASE | 0.087441 | -0.01064 | 1.697037 | 0.094411 | 0.477436 | -4.53677 |
| REACTOME_SYNTHESIS_OF_DNA | -0.1041 | -0.13229 | -1.6969 | 0.094438 | 0.477436 | -4.53698 |
| REACTOME_OTHER_INTERLEUKIN_SIGNALING | 0.113071 | -0.02866 | 1.696825 | 0.094452 | 0.477436 | -4.53709 |
| REACTOME_PIWI_INTERACTING_RNA_PIRNA_BIOGENESIS | -0.08299 | -0.04366 | -1.69418 | 0.094956 | 0.4792 | -4.54108 |
| BENPORATH_ES_1 | -0.06775 | -0.0705 | -1.69377 | 0.095034 | 0.4792 | -4.5417 |
| REACTOME_HCMV_LATE_EVENTS | -0.13681 | -0.0339 | -1.69348 | 0.09509 | 0.4792 | -4.54214 |
| BIOCARTA_CPSF_PATHWAY | -0.15618 | -0.08712 | -1.69337 | 0.095111 | 0.4792 | -4.5423 |
| MEISSNER_NPC_ICP_WITH_H3_UNMETHYLATED | 0.10303 | -0.03934 | 1.692991 | 0.095183 | 0.4792 | -4.54287 |
| DACOSTA_LOW_DOSE_UV_RESPONSE_VIA_ERCC3_XPCS_UP | 0.097075 | 0.012158 | 1.692627 | 0.095253 | 0.4792 | -4.54342 |
| ZIRN_TRETINOIN_RESPONSE_WT1_DN | -0.15198 | 0.014921 | -1.69143 | 0.095481 | 0.479893 | -4.54522 |
| REACTOME_EXTRACELLULAR_MATRIX_ORGANIZATION | 0.074383 | -0.05159 | 1.691122 | 0.095541 | 0.479893 | -4.54569 |
| REACTOME_ANDROGEN_BIOSYNTHESIS | 0.130635 | 0.007884 | 1.690333 | 0.095693 | 0.480116 | -4.54687 |
| BIOCARTA_CREM_PATHWAY | -0.1331 | 0.018097 | -1.68869 | 0.096009 | 0.480116 | -4.54935 |
| REACTOME_FGFR2_ALTERNATIVE_SPLICING | -0.12477 | -0.07843 | -1.68867 | 0.096013 | 0.480116 | -4.54938 |
| JIANG_TIP30_TARGETS_DN | 0.090006 | -0.15639 | 1.688638 | 0.096019 | 0.480116 | -4.54942 |
| WP_CARDIAC_HYPERTROPHIC_RESPONSE | -0.08073 | -0.14393 | -1.68838 | 0.096069 | 0.480116 | -4.54982 |
| MEBARKI_HCC_PROGENITOR_WNT_UP_BLOCKED_BY_FZD8CRD | 0.075427 | -0.06363 | 1.688017 | 0.096139 | 0.480116 | -4.55036 |
| SHEN_SMARCA2_TARGETS_UP | -0.15158 | -0.0461 | -1.68782 | 0.096176 | 0.480116 | -4.55065 |
| WIEMANN_TELOMERE_SHORTENING_AND_CHRONIC_LIVER_DAMAGE_UP | 0.091739 | -0.24649 | 1.68755 | 0.096229 | 0.480116 | -4.55106 |
| CHEMNITZ_RESPONSE_TO_PROSTAGLANDIN_E2_DN | -0.04582 | -0.09234 | -1.68736 | 0.096265 | 0.480116 | -4.55134 |
| REACTOME_HCMV_INFECTION | -0.10818 | -0.05649 | -1.68652 | 0.096429 | 0.480558 | -4.55262 |
| REACTOME_ACTIVATED_NTRK3_SIGNALS_THROUGH_RAS | -0.14185 | -0.08592 | -1.68424 | 0.096869 | 0.480764 | -4.55603 |
| PID_HIF1_TFPATHWAY | 0.115037 | -0.02894 | 1.683779 | 0.096959 | 0.480764 | -4.55672 |
| WANG_LSD1_TARGETS_DN | 0.087747 | -0.02933 | 1.683314 | 0.09705 | 0.480764 | -4.55742 |
| CAVARD_LIVER_CANCER_MALIGNANT_VS_BENIGN | 0.05244 | -0.31759 | 1.683176 | 0.097076 | 0.480764 | -4.55763 |
| REACTOME_SERINE_BIOSYNTHESIS | 0.126444 | -0.00726 | 1.68315 | 0.097081 | 0.480764 | -4.55767 |
| REACTOME_SURFACTANT_METABOLISM | 0.081038 | 0.001998 | 1.682901 | 0.09713 | 0.480764 | -4.55804 |
| KAUFFMANN_DNA_REPLICATION_GENES | -0.08576 | -0.06964 | -1.68247 | 0.097213 | 0.480764 | -4.55868 |
| CHASSOT_SKIN_WOUND | 0.203503 | 0.02767 | 1.682373 | 0.097232 | 0.480764 | -4.55883 |
| NIKOLSKY_BREAST_CANCER_8Q12_Q22_AMPLICON | -0.07458 | -0.01015 | -1.68222 | 0.097263 | 0.480764 | -4.55907 |
| MCMURRAY_TP53_HRAS_COOPERATION_RESPONSE_DN | 0.06119 | -0.01252 | 1.681792 | 0.097346 | 0.480764 | -4.5597 |
| BROWNE_HCMV_INFECTION_4HR_DN | -0.05242 | -0.11176 | -1.6816 | 0.097382 | 0.480764 | -4.55998 |
| REACTOME_REGULATION_OF_TP53_ACTIVITY | -0.07383 | -0.11472 | -1.68087 | 0.097526 | 0.480764 | -4.56109 |
| BOYAULT_LIVER_CANCER_SUBCLASS_G23_DN | -0.14353 | -0.07838 | -1.68035 | 0.097628 | 0.480764 | -4.56186 |
| MOOTHA_PYR | -0.16351 | -0.02031 | -1.68026 | 0.097645 | 0.480764 | -4.562 |
| BIOCARTA_IGF1MTOR_PATHWAY | -0.11821 | -0.06141 | -1.67977 | 0.097741 | 0.480764 | -4.56273 |
| KEGG_GLYCOSAMINOGLYCAN_BIOSYNTHESIS_HEPARAN_SULFATE | -0.07123 | -0.04201 | -1.6795 | 0.097794 | 0.480764 | -4.56314 |
| REACTOME_APOPTOSIS_INDUCED_DNA_FRAGMENTATION | -0.18812 | 0.014154 | -1.6794 | 0.097813 | 0.480764 | -4.56328 |
| WP_PARKINSONS_DISEASE_PATHWAY | -0.08971 | -0.03056 | -1.67931 | 0.09783 | 0.480764 | -4.56341 |
| MIKKELSEN_MEF_HCP_WITH_H3_UNMETHYLATED | 0.105612 | -0.05754 | 1.678467 | 0.097996 | 0.480987 | -4.56468 |
| AMIT_SERUM_RESPONSE_480_MCF10A | -0.08836 | -0.0484 | -1.67807 | 0.098074 | 0.480987 | -4.56527 |
| SENESE_HDAC1_AND_HDAC2_TARGETS_UP | 0.071922 | -0.09233 | 1.677924 | 0.098102 | 0.480987 | -4.56549 |
| REACTOME_PHENYLALANINE_METABOLISM | 0.125187 | -0.00478 | 1.675667 | 0.098546 | 0.481973 | -4.56886 |
| REACTOME_PROSTANOID_LIGAND_RECEPTORS | -0.1131 | 0.003095 | -1.67564 | 0.098551 | 0.481973 | -4.56891 |
| MURATA_VIRULENCE_OF_H_PILORI | 0.078619 | -0.14716 | 1.675265 | 0.098625 | 0.481973 | -4.56946 |
| FIGUEROA_AML_METHYLATION_CLUSTER_6_DN | 0.073417 | -0.16376 | 1.67471 | 0.098734 | 0.481973 | -4.57029 |
| WP_OVERVIEW_OF_NANOPARTICLE_EFFECTS | 0.071996 | -0.2917 | 1.674672 | 0.098742 | 0.481973 | -4.57035 |
| RICKMAN_HEAD_AND_NECK_CANCER_D | 0.071163 | -0.24139 | 1.674591 | 0.098757 | 0.481973 | -4.57047 |
| LIN_SILENCED_BY_TUMOR_MICROENVIRONMENT | 0.061609 | -0.04261 | 1.673424 | 0.098988 | 0.482632 | -4.57221 |
| NIKOLSKY_BREAST_CANCER_20P13_AMPLICON | -0.11871 | 0.009123 | -1.67293 | 0.099085 | 0.482632 | -4.57295 |
| KIM_WT1_TARGETS_DN | -0.09057 | -0.13093 | -1.67222 | 0.099225 | 0.482632 | -4.574 |
| MOREAUX_B_LYMPHOCYTE_MATURATION_BY_TACI_DN | -0.12576 | -0.09491 | -1.6721 | 0.09925 | 0.482632 | -4.57418 |
| KEGG_VASOPRESSIN_REGULATED_WATER_REABSORPTION | -0.09064 | -0.07171 | -1.67162 | 0.099344 | 0.482632 | -4.57489 |
| REACTOME_VISUAL_PHOTOTRANSDUCTION | 0.05718 | -0.0792 | 1.671606 | 0.099348 | 0.482632 | -4.57492 |
| WP_HEME_BIOSYNTHESIS | -0.14241 | 0.00846 | -1.66951 | 0.099763 | 0.484279 | -4.57804 |
| POS_RESPONSE_TO_HISTAMINE_DN | -0.12736 | -0.01443 | -1.6688 | 0.099904 | 0.484373 | -4.5791 |
| PID_EPHA_FWDPATHWAY | 0.06568 | -0.05818 | 1.668652 | 0.099934 | 0.484373 | -4.57932 |
| BIOCARTA_ETS_PATHWAY | 0.115797 | -0.05672 | 1.667083 | 0.100247 | 0.485242 | -4.58165 |
| BORLAK_LIVER_CANCER_EGF_UP | 0.070048 | -0.22807 | 1.666988 | 0.100266 | 0.485242 | -4.58179 |
| REACTOME_GAP_JUNCTION_TRAFFICKING_AND_REGULATION | 0.07286 | -0.04693 | 1.666184 | 0.100427 | 0.48548 | -4.58299 |
| REACTOME_HCMV_EARLY_EVENTS | -0.10155 | -0.05361 | -1.66581 | 0.100501 | 0.48548 | -4.58354 |
| KINNEY_DNMT1_METHYLATION_TARGETS | 0.146482 | 0.037841 | 1.665598 | 0.100544 | 0.48548 | -4.58386 |
| REACTOME_SEPARATION_OF_SISTER_CHROMATIDS | -0.09533 | -0.10749 | -1.66397 | 0.100869 | 0.486682 | -4.58627 |
| BLANCO_MELO_BRONCHIAL_EPITHELIAL_CELLS_INFLUENZA_A_INFECTION_DN | 0.075881 | -0.03648 | 1.662685 | 0.101128 | 0.487151 | -4.58818 |
| NIKOLSKY_BREAST_CANCER_14Q22_AMPLICON | -0.11704 | -0.00757 | -1.6624 | 0.101186 | 0.487151 | -4.58861 |
| REACTOME_SIGNALING_BY_NTRK3_TRKC | -0.10693 | -0.03792 | -1.66235 | 0.101196 | 0.487151 | -4.58868 |
| WP_REGULATION_OF_WNT_BCATENIN_SIGNALING_BY_SMALL_MOLECULE_COMPOUNDS | 0.090767 | -0.06324 | 1.661513 | 0.101364 | 0.487279 | -4.58992 |
| REACTOME_REGULATION_OF_TP53_ACTIVITY_THROUGH_ACETYLATION | -0.10435 | -0.09979 | -1.66137 | 0.101394 | 0.487279 | -4.59014 |
| REACTOME_GLYCOGEN_BREAKDOWN_GLYCOGENOLYSIS | -0.12852 | -0.0111 | -1.66107 | 0.101453 | 0.487279 | -4.59057 |
| ICHIBA_GRAFT_VERSUS_HOST_DISEASE_D7_UP | 0.048331 | -0.425 | 1.660511 | 0.101566 | 0.487395 | -4.5914 |
| REACTOME_XENOBIOTICS | 0.138806 | -0.01637 | 1.660194 | 0.10163 | 0.487395 | -4.59187 |
| MARKEY_RB1_CHRONIC_LOF_DN | 0.088896 | -0.06244 | 1.659213 | 0.101828 | 0.487978 | -4.59332 |
| XU_RESPONSE_TO_TRETINOIN_UP | 0.100575 | -0.30713 | 1.657871 | 0.1021 | 0.488912 | -4.59531 |
| IVANOVA_HEMATOPOIESIS_STEM_CELL_AND_PROGENITOR | -0.05171 | -0.08105 | -1.65737 | 0.102202 | 0.489034 | -4.59606 |
| WP_SPINAL_CORD_INJURY | 0.055116 | -0.2162 | 1.656953 | 0.102286 | 0.489067 | -4.59667 |
| KEGG_COMPLEMENT_AND_COAGULATION_CASCADES | 0.04748 | -0.27227 | 1.655587 | 0.102563 | 0.489913 | -4.59869 |
| BIOCARTA_IGF1_PATHWAY | -0.14002 | -0.03698 | -1.65506 | 0.102671 | 0.489913 | -4.59947 |
| ZHOU_INFLAMMATORY_RESPONSE_FIMA_DN | -0.06369 | -0.03506 | -1.65495 | 0.102694 | 0.489913 | -4.59963 |
| CAIRO_LIVER_DEVELOPMENT_DN | 0.039376 | -0.13947 | 1.653741 | 0.102939 | 0.490717 | -4.60141 |
| STAMBOLSKY_RESPONSE_TO_VITAMIN_D3_DN | -0.06952 | -0.04212 | -1.65295 | 0.103101 | 0.490981 | -4.60258 |
| VILLANUEVA_LIVER_CANCER_KRT19_UP | -0.0814 | -0.03443 | -1.65223 | 0.103247 | 0.490981 | -4.60364 |
| GLASS_IGF2BP1_CLIP_TARGETS_KNOCKDOWN_DN | -0.11719 | -0.00997 | -1.65216 | 0.103263 | 0.490981 | -4.60374 |
| REACTOME_NUCLEAR_EVENTS_KINASE_AND_TRANSCRIPTION_FACTOR_ACTIVATION | 0.085926 | -0.03218 | 1.651499 | 0.103398 | 0.490981 | -4.60472 |
| MARSON_FOXP3_TARGETS_STIMULATED_UP | 0.076293 | -0.10165 | 1.650874 | 0.103526 | 0.490981 | -4.60564 |
| GARGALOVIC_RESPONSE_TO_OXIDIZED_PHOSPHOLIPIDS_YELLOW_DN | -0.1134 | -0.0788 | -1.65087 | 0.103526 | 0.490981 | -4.60564 |
| REACTOME_TRANSLATION_OF_REPLICASE_AND_ASSEMBLY_OF_THE_REPLICATION_TRANSCRIPTION_COMPLEX | -0.14049 | -0.08009 | -1.65036 | 0.10363 | 0.490981 | -4.60639 |
| PID_IL5_PATHWAY | 0.12681 | -0.00807 | 1.650145 | 0.103675 | 0.490981 | -4.60671 |
| BENPORATH_SUZ12_TARGETS | 0.076093 | -0.03013 | 1.649387 | 0.103831 | 0.490981 | -4.60783 |
| NELSON_RESPONSE_TO_ANDROGEN_DN | -0.11085 | -0.0023 | -1.64918 | 0.103874 | 0.490981 | -4.60814 |
| REACTOME_ACETYLCHOLINE_REGULATES_INSULIN_SECRETION | 0.107525 | -0.01038 | 1.648497 | 0.104014 | 0.490981 | -4.60914 |
| CROONQUIST_STROMAL_STIMULATION_UP | 0.099842 | -0.16249 | 1.647815 | 0.104154 | 0.490981 | -4.61014 |
| REACTOME_STING_MEDIATED_INDUCTION_OF_HOST_IMMUNE_RESPONSES | 0.131379 | -0.0093 | 1.647252 | 0.10427 | 0.490981 | -4.61097 |
| REACTOME_SIGNALING_BY_FGFR | -0.06999 | -0.03503 | -1.64643 | 0.104439 | 0.490981 | -4.61217 |
| REACTOME_TRANSCRIPTIONAL_REGULATION_BY_SMALL_RNAS | -0.14437 | -0.03394 | -1.64633 | 0.10446 | 0.490981 | -4.61232 |
| IGLESIAS_E2F_TARGETS_DN | 0.110103 | -0.00828 | 1.646271 | 0.104473 | 0.490981 | -4.61241 |
| GRAHAM_CML_DIVIDING_VS_NORMAL_QUIESCENT_DN | 0.071329 | -0.21759 | 1.645631 | 0.104605 | 0.490981 | -4.61335 |
| REACTOME_FORMATION_OF_TC_NER_PRE_INCISION_COMPLEX | -0.11756 | -0.14543 | -1.64491 | 0.104754 | 0.490981 | -4.61441 |
| REACTOME_SIGNALING_BY_FGFR_IN_DISEASE | -0.07372 | -0.01822 | -1.64466 | 0.104806 | 0.490981 | -4.61478 |
| REACTOME_RETROGRADE_TRANSPORT_AT_THE_TRANS_GOLGI_NETWORK | -0.10346 | -0.14136 | -1.64463 | 0.104812 | 0.490981 | -4.61482 |
| WP_THERMOGENESIS | -0.04661 | -0.04367 | -1.64387 | 0.104968 | 0.490981 | -4.61593 |
| REACTOME_REGULATION_OF_LIPID_METABOLISM_BY_PPARALPHA | -0.07471 | -0.07788 | -1.64368 | 0.105009 | 0.490981 | -4.61622 |
| DURAND_STROMA_NS_UP | 0.047508 | -0.08545 | 1.643298 | 0.105088 | 0.490981 | -4.61678 |
| GERHOLD_RESPONSE_TO_TZD_DN | 0.13746 | -0.06973 | 1.643102 | 0.105128 | 0.490981 | -4.61706 |
| WEBER_METHYLATED_HCP_IN_FIBROBLAST_DN | 0.133419 | -0.00823 | 1.642217 | 0.105312 | 0.490981 | -4.61836 |
| ACOSTA_PROLIFERATION_INDEPENDENT_MYC_TARGETS_DN | 0.069171 | -0.10971 | 1.641701 | 0.105419 | 0.490981 | -4.61912 |
| JOSEPH_RESPONSE_TO_SODIUM_BUTYRATE_DN | 0.081905 | -0.01079 | 1.641626 | 0.105435 | 0.490981 | -4.61923 |
| REACTOME_FORMATION_OF_RNA_POL_II_ELONGATION_COMPLEX | -0.09943 | -0.20655 | -1.64155 | 0.105451 | 0.490981 | -4.61934 |
| REACTOME_CELL_CYCLE | -0.08055 | -0.10734 | -1.64146 | 0.105469 | 0.490981 | -4.61947 |
| HALMOS_CEBPA_TARGETS_DN | 0.087334 | -0.03026 | 1.641358 | 0.105491 | 0.490981 | -4.61962 |
| NEMETH_INFLAMMATORY_RESPONSE_LPS_UP | 0.078984 | -0.27162 | 1.641141 | 0.105536 | 0.490981 | -4.61994 |
| BENPORATH_PRC2_TARGETS | 0.088592 | -0.03057 | 1.64078 | 0.105611 | 0.490981 | -4.62047 |
| KIM_ALL_DISORDERS_DURATION_CORR_UP | -0.12869 | 0.001778 | -1.64044 | 0.105682 | 0.490981 | -4.62097 |
| ZHENG_IL22_SIGNALING_DN | -0.05846 | 0.005753 | -1.64038 | 0.105694 | 0.490981 | -4.62105 |
| WU_HBX_TARGETS_3_DN | 0.097666 | -0.00296 | 1.640377 | 0.105695 | 0.490981 | -4.62106 |
| REACTOME_IRF3_MEDIATED_INDUCTION_OF_TYPE_I_IFN | 0.119872 | -0.00625 | 1.63957 | 0.105863 | 0.491018 | -4.62224 |
| REACTOME_S_PHASE | -0.09755 | -0.11129 | -1.63895 | 0.105993 | 0.491018 | -4.62315 |
| MARSHALL_VIRAL_INFECTION_RESPONSE_DN | 0.104005 | -0.13324 | 1.638671 | 0.106051 | 0.491018 | -4.62355 |
| NOUZOVA_TRETINOIN_AND_H4_ACETYLATION | -0.09891 | -0.17325 | -1.63854 | 0.106078 | 0.491018 | -4.62374 |
| REACTOME_METABOLISM_OF_STEROID_HORMONES | 0.075826 | -0.15763 | 1.638351 | 0.106118 | 0.491018 | -4.62402 |
| ROPERO_HDAC2_TARGETS | 0.037333 | -0.29386 | 1.638121 | 0.106166 | 0.491018 | -4.62436 |
| REACTOME_TRANSPORT_OF_INORGANIC_CATIONS_ANIONS_AND_AMINO_ACIDS_OLIGOPEPTIDES | 0.061536 | -0.02384 | 1.63748 | 0.1063 | 0.491281 | -4.6253 |
| REACTOME_METABOLISM_OF_RNA | -0.09685 | -0.16943 | -1.63597 | 0.106617 | 0.491852 | -4.62751 |
| HUMMEL_BURKITTS_LYMPHOMA_UP | -0.08542 | -0.06076 | -1.63584 | 0.106643 | 0.491852 | -4.62769 |
| REACTOME_GP1B_IX_V_ACTIVATION_SIGNALLING | 0.12735 | -0.0076 | 1.63547 | 0.106722 | 0.491852 | -4.62823 |
| KUROZUMI_RESPONSE_TO_ONCOCYTIC_VIRUS | 0.062221 | -0.33845 | 1.635417 | 0.106733 | 0.491852 | -4.62831 |
| SOUCEK_MYC_TARGETS | 0.083751 | -0.32568 | 1.634368 | 0.106953 | 0.492456 | -4.62984 |
| ABBUD_LIF_SIGNALING_1_UP | 0.079147 | -0.25375 | 1.634058 | 0.107019 | 0.492456 | -4.6303 |
| WP_SUDDEN_INFANT_DEATH_SYNDROME_SIDS_SUSCEPTIBILITY_PATHWAYS | 0.045233 | -0.16613 | 1.633172 | 0.107205 | 0.492502 | -4.63159 |
| WIEMANN_TELOMERE_SHORTENING_AND_CHRONIC_LIVER_DAMAGE_DN | 0.124221 | -0.16887 | 1.633107 | 0.107219 | 0.492502 | -4.63168 |
| WP_HAIR_FOLLICLE_DEVELOPMENT_CYTODIFFERENTIATION_PART_3_OF_3 | 0.062027 | -0.01035 | 1.632497 | 0.107348 | 0.492502 | -4.63257 |
| SHIN_B_CELL_LYMPHOMA_CLUSTER_6 | 0.078058 | -0.09178 | 1.632331 | 0.107383 | 0.492502 | -4.63281 |
| KALMA_E2F1_TARGETS | -0.19167 | -0.00792 | -1.63217 | 0.107416 | 0.492502 | -4.63304 |
| REACTOME_RELAXIN_RECEPTORS | 0.155236 | -0.18195 | 1.631425 | 0.107574 | 0.492874 | -4.63414 |
| CAIRO_HEPATOBLASTOMA_CLASSES_UP | -0.10303 | -0.10991 | -1.63077 | 0.107712 | 0.493148 | -4.63508 |
| REACTOME_PEPTIDE_HORMONE_BIOSYNTHESIS | 0.149645 | 0.035425 | 1.629738 | 0.107931 | 0.493798 | -4.63659 |
| REACTOME_RUNX1_REGULATES_TRANSCRIPTION_OF_GENES_INVOLVED_IN_BCR_SIGNALING | -0.16265 | -0.00834 | -1.62936 | 0.108012 | 0.493815 | -4.63715 |
| HANN_RESISTANCE_TO_BCL2_INHIBITOR_UP | 0.071999 | -0.01176 | 1.628515 | 0.108191 | 0.493973 | -4.63837 |
| HOLLEMAN_VINCRISTINE_RESISTANCE_B_ALL_UP | -0.10254 | -0.03486 | -1.62846 | 0.108202 | 0.493973 | -4.63845 |
| YIH_RESPONSE_TO_ARSENITE_C2 | -0.1427 | -0.0089 | -1.62762 | 0.10838 | 0.494431 | -4.63967 |
| DAVICIONI_MOLECULAR_ARMS_VS_ERMS_UP | -0.06733 | -0.12301 | -1.62682 | 0.108552 | 0.494746 | -4.64084 |
| TONKS_TARGETS_OF_RUNX1_RUNX1T1_FUSION_SUSTAINED_IN_MONOCYTE_DN | 0.091567 | -0.40903 | 1.625891 | 0.108749 | 0.494746 | -4.64219 |
| KEGG_NOD_LIKE_RECEPTOR_SIGNALING_PATHWAY | 0.077078 | -0.14999 | 1.62561 | 0.108809 | 0.494746 | -4.6426 |
| REACTOME_INTERLEUKIN_36_PATHWAY | 0.173164 | 0.038475 | 1.625281 | 0.108879 | 0.494746 | -4.64307 |
| ROY_WOUND_BLOOD_VESSEL_DN | 0.104946 | -0.02186 | 1.624954 | 0.108949 | 0.494746 | -4.64355 |
| REACTOME_TYSND1_CLEAVES_PEROXISOMAL_PROTEINS | -0.14947 | -0.00171 | -1.62459 | 0.109027 | 0.494746 | -4.64408 |
| FARMER_BREAST_CANCER_CLUSTER_7 | 0.084542 | -0.00084 | 1.62458 | 0.109029 | 0.494746 | -4.64409 |
| GALE_APL_WITH_FLT3_MUTATED_UP | -0.12245 | -0.03305 | -1.62427 | 0.109094 | 0.494746 | -4.64453 |
| CREIGHTON_ENDOCRINE_THERAPY_RESISTANCE_1 | -0.06691 | -0.09583 | -1.62402 | 0.109149 | 0.494746 | -4.64491 |
| CASTELLANO_HRAS_TARGETS_DN | 0.13339 | -0.20815 | 1.62331 | 0.109301 | 0.495081 | -4.64593 |
| WUNDER_INFLAMMATORY_RESPONSE_AND_CHOLESTEROL_DN | 0.074405 | -0.42425 | 1.622919 | 0.109384 | 0.495108 | -4.6465 |
| REACTOME_ABC_FAMILY_PROTEINS_MEDIATED_TRANSPORT | -0.07571 | -0.24405 | -1.62227 | 0.109524 | 0.495111 | -4.64744 |
| WEBER_METHYLATED_ICP_IN_FIBROBLAST | 0.122626 | -0.18888 | 1.622191 | 0.109541 | 0.495111 | -4.64756 |
| REACTOME_VIRAL_MESSENGER_RNA_SYNTHESIS | -0.14989 | -0.01325 | -1.62178 | 0.109629 | 0.495159 | -4.64815 |
| ZWANG_TRANSIENTLY_UP_BY_2ND_EGF_PULSE_ONLY | 0.061557 | -0.15428 | 1.620965 | 0.109804 | 0.495597 | -4.64933 |
| BIOCARTA_PCAF_PATHWAY | 0.111729 | -0.076 | 1.619423 | 0.110136 | 0.496275 | -4.65157 |
| REACTOME_DISORDERS_OF_TRANSMEMBRANE_TRANSPORTERS | -0.05978 | -0.14308 | -1.61926 | 0.11017 | 0.496275 | -4.6518 |
| SCHLINGEMANN_SKIN_CARCINOGENESIS_TPA_DN | 0.068553 | -0.05152 | 1.618748 | 0.110281 | 0.496275 | -4.65254 |
| XIE_ST_HSC_S1PR3_OE_UP | 0.075749 | -0.00518 | 1.618321 | 0.110373 | 0.496275 | -4.65316 |
| MAINA_HYPOXIA_VHL_TARGETS_UP | 0.133552 | -0.1501 | 1.618131 | 0.110414 | 0.496275 | -4.65344 |
| REACTOME_TELOMERE_C_STRAND_LAGGING_STRAND_SYNTHESIS | -0.12321 | -0.03604 | -1.61799 | 0.110445 | 0.496275 | -4.65364 |
| BIOCARTA_P53_PATHWAY | 0.111822 | -0.00325 | 1.617312 | 0.110591 | 0.496275 | -4.65462 |
| FOURNIER_ACINAR_DEVELOPMENT_EARLY_DN | 0.143681 | 0.002222 | 1.61696 | 0.110667 | 0.496275 | -4.65513 |
| PID_AURORA_B_PATHWAY | -0.10259 | -0.01329 | -1.61676 | 0.11071 | 0.496275 | -4.65542 |
| REACTOME_CELL_CELL_COMMUNICATION | 0.051045 | -0.02274 | 1.616653 | 0.110734 | 0.496275 | -4.65557 |
| PLASARI_TGFB1_SIGNALING_VIA_NFIC_1HR_DN | -0.0856 | -0.06291 | -1.61582 | 0.110914 | 0.496469 | -4.65678 |
| REACTOME_DNA_DAMAGE_REVERSAL | -0.18776 | -0.05387 | -1.61551 | 0.110981 | 0.496469 | -4.65722 |
| WP_LIPID_PARTICLES_COMPOSITION | 0.098621 | -0.01268 | 1.615074 | 0.111076 | 0.496469 | -4.65785 |
| JOHANSSON_GLIOMAGENESIS_BY_PDGFB_DN | -0.08236 | 0.006585 | -1.61501 | 0.111089 | 0.496469 | -4.65794 |
| ODONNELL_TFRC_TARGETS_DN | -0.07925 | -0.05779 | -1.61355 | 0.111406 | 0.496718 | -4.66005 |
| PACHER_TARGETS_OF_IGF1_AND_IGF2_UP | 0.076777 | -0.04274 | 1.613392 | 0.111441 | 0.496718 | -4.66028 |
| PID_P53_DOWNSTREAM_PATHWAY | 0.067433 | -0.07564 | 1.6132 | 0.111483 | 0.496718 | -4.66056 |
| BIOCARTA_GPCR_PATHWAY | -0.09905 | -0.08676 | -1.613 | 0.111526 | 0.496718 | -4.66084 |
| KEGG_NICOTINATE_AND_NICOTINAMIDE_METABOLISM | 0.083592 | -0.01268 | 1.611852 | 0.111777 | 0.496718 | -4.6625 |
| FISCHER_DREAM_TARGETS | -0.08804 | -0.09828 | -1.61153 | 0.111846 | 0.496718 | -4.66296 |
| WP_ENDOPLASMIC_RETICULUM_STRESS_RESPONSE_IN_CORONAVIRUS_INFECTION | -0.06467 | -0.24448 | -1.61132 | 0.111892 | 0.496718 | -4.66326 |
| REACTOME_CYTOPROTECTION_BY_HMOX1 | -0.09616 | -0.18095 | -1.61069 | 0.112031 | 0.496718 | -4.66418 |
| THILLAINADESAN_ZNF217_TARGETS_UP | -0.07325 | -0.02555 | -1.61054 | 0.112062 | 0.496718 | -4.66439 |
| ENGELMANN_CANCER_PROGENITORS_DN | 0.056051 | -0.04534 | 1.610419 | 0.11209 | 0.496718 | -4.66457 |
| REACTOME_SIRT1_NEGATIVELY_REGULATES_RRNA_EXPRESSION | -0.16787 | -0.01951 | -1.61032 | 0.11211 | 0.496718 | -4.6647 |
| GENTILE_UV_RESPONSE_CLUSTER_D5 | -0.1016 | -0.02968 | -1.60991 | 0.112202 | 0.496718 | -4.66531 |
| REACTOME_CELL_CYCLE_CHECKPOINTS | -0.08214 | -0.12112 | -1.60935 | 0.112323 | 0.496718 | -4.6661 |
| ROESSLER_LIVER_CANCER_METASTASIS_DN | -0.09437 | -0.12616 | -1.60862 | 0.112484 | 0.496718 | -4.66716 |
| REACTOME_INTERACTION_BETWEEN_L1_AND_ANKYRINS | 0.099074 | 0.004024 | 1.608368 | 0.112538 | 0.496718 | -4.66752 |
| FIGUEROA_AML_METHYLATION_CLUSTER_7_UP | -0.06204 | -0.06086 | -1.60802 | 0.112614 | 0.496718 | -4.66802 |
| REACTOME_ZINC_EFFLUX_AND_COMPARTMENTALIZATION_BY_THE_SLC30_FAMILY | -0.11392 | -0.0168 | -1.60761 | 0.112705 | 0.496718 | -4.66861 |
| PID_HDAC_CLASSII_PATHWAY | -0.09935 | -0.02852 | -1.60743 | 0.112744 | 0.496718 | -4.66886 |
| REACTOME_ARYL_HYDROCARBON_RECEPTOR_SIGNALLING | -0.11937 | -0.0038 | -1.60732 | 0.112769 | 0.496718 | -4.66903 |
| KRIGE_AMINO_ACID_DEPRIVATION | 0.107255 | -0.0385 | 1.607072 | 0.112823 | 0.496718 | -4.66938 |
| WP_PI3KAKTMTOR_VITD3_SIGNALING | 0.090364 | -0.24214 | 1.607068 | 0.112824 | 0.496718 | -4.66939 |
| REACTOME_RESOLUTION_OF_ABASIC_SITES_AP_SITES | -0.13382 | -0.00582 | -1.60689 | 0.112862 | 0.496718 | -4.66964 |
| WIELAND_UP_BY_HBV_INFECTION | 0.053411 | -0.49489 | 1.60641 | 0.112968 | 0.496741 | -4.67033 |
| REACTOME_TYROSINE_CATABOLISM | 0.12461 | 0.018173 | 1.606072 | 0.113043 | 0.496741 | -4.67082 |
| ACEVEDO_LIVER_CANCER_WITH_H3K9ME3_DN | -0.04152 | -0.16692 | -1.60579 | 0.113105 | 0.496741 | -4.67122 |
| KUMAMOTO_RESPONSE_TO_NUTLIN_3A_UP | 0.138233 | 0.005024 | 1.605126 | 0.113251 | 0.496741 | -4.67218 |
| FALVELLA_SMOKERS_WITH_LUNG_CANCER | 0.084026 | -0.05079 | 1.604847 | 0.113313 | 0.496741 | -4.67258 |
| REACTOME_DNA_DAMAGE_RECOGNITION_IN_GG_NER | -0.12519 | -0.19364 | -1.60474 | 0.113336 | 0.496741 | -4.67273 |
| WINNEPENNINCKX_MELANOMA_METASTASIS_UP | -0.11163 | -0.03246 | -1.60407 | 0.113484 | 0.497002 | -4.67369 |
| PEREZ_TP63_TARGETS | 0.048425 | -0.10787 | 1.603766 | 0.113551 | 0.497002 | -4.67413 |
| REACTOME_GAP_JUNCTION_ASSEMBLY | 0.089763 | -0.02769 | 1.602075 | 0.113925 | 0.498004 | -4.67655 |
| REACTOME_TP53_REGULATES_TRANSCRIPTION_OF_SEVERAL_ADDITIONAL_CELL_DEATH_GENES_WHOSE_SPECIFIC_ROLES_IN_P53_DEPENDENT_APOPTOSIS_REMAIN_UNCERTAIN | 0.085741 | -0.11935 | 1.602023 | 0.113937 | 0.498004 | -4.67663 |
| LASTOWSKA_COAMPLIFIED_WITH_MYCN | -0.06249 | -0.07993 | -1.60061 | 0.114249 | 0.498562 | -4.67865 |
| REACTOME_INTERLEUKIN_3_INTERLEUKIN_5_AND_GM_CSF_SIGNALING | 0.089012 | -0.03876 | 1.600455 | 0.114284 | 0.498562 | -4.67887 |
| RAMJAUN_APOPTOSIS_BY_TGFB1_VIA_SMAD4_DN | 0.100146 | -0.08228 | 1.600387 | 0.114299 | 0.498562 | -4.67897 |
| SCHLESINGER_H3K27ME3_IN_NORMAL_AND_METHYLATED_IN_CANCER | 0.116763 | -0.05877 | 1.599569 | 0.114481 | 0.498769 | -4.68014 |
| FUJIWARA_PARK2_HEPATOCYTE_PROLIFERATION_DN | 0.102877 | -0.1142 | 1.599468 | 0.114504 | 0.498769 | -4.68029 |
| WP_OVARIAN_INFERTILITY | 0.061016 | -0.2052 | 1.598782 | 0.114656 | 0.499091 | -4.68127 |
| WP_KALLMANN_SYNDROME | 0.059402 | -0.03374 | 1.597762 | 0.114883 | 0.499456 | -4.68273 |
| REACTOME_SIGNALING_BY_MAPK_MUTANTS | 0.094678 | -0.30114 | 1.597702 | 0.114897 | 0.499456 | -4.68281 |
| KEGG_PPAR_SIGNALING_PATHWAY | 0.063868 | -0.08781 | 1.596666 | 0.115128 | 0.500072 | -4.68429 |
| REACTOME_HEME_DEGRADATION | 0.110435 | -0.05842 | 1.595759 | 0.115331 | 0.500072 | -4.68559 |
| REACTOME_BASE_EXCISION_REPAIR | -0.12168 | -0.02883 | -1.59507 | 0.115485 | 0.500072 | -4.68657 |
| GREGORY_SYNTHETIC_LETHAL_WITH_IMATINIB | -0.07064 | -0.12193 | -1.59424 | 0.115671 | 0.500072 | -4.68776 |
| PID_EPHB_FWD_PATHWAY | -0.10226 | -0.02537 | -1.59409 | 0.115705 | 0.500072 | -4.68798 |
| REACTOME_APC_C_MEDIATED_DEGRADATION_OF_CELL_CYCLE_PROTEINS | -0.10125 | -0.16729 | -1.59408 | 0.115708 | 0.500072 | -4.68799 |
| REACTOME_OREXIN_AND_NEUROPEPTIDES_FF_AND_QRFP_BIND_TO_THEIR_RESPECTIVE_RECEPTORS | 0.164342 | 0.040489 | 1.593977 | 0.11573 | 0.500072 | -4.68813 |
| REACTOME_ACTIVATION_OF_RAS_IN_B_CELLS | -0.16602 | -0.1452 | -1.59392 | 0.115744 | 0.500072 | -4.68822 |
| PID_P38_MK2_PATHWAY | 0.098626 | -0.09411 | 1.593906 | 0.115746 | 0.500072 | -4.68824 |
| WP_CARDIAC_PROGENITOR_DIFFERENTIATION | 0.078956 | -0.11458 | 1.593035 | 0.115941 | 0.500376 | -4.68948 |
| WP_EXTRACELLULAR_VESICLES_IN_THE_CROSSTALK_OF_CARDIAC_CELLS | 0.097435 | -0.11391 | 1.592892 | 0.115973 | 0.500376 | -4.68968 |
| REACTOME_ENDOSOMAL_SORTING_COMPLEX_REQUIRED_FOR_TRANSPORT_ESCRT | -0.13511 | -0.04918 | -1.59202 | 0.11617 | 0.500612 | -4.69093 |
| DORMOY_ELAVL1_TARGETS | -0.08442 | -0.1022 | -1.59195 | 0.116185 | 0.500612 | -4.69103 |
| REACTOME_EXPORT_OF_VIRAL_RIBONUCLEOPROTEINS_FROM_NUCLEUS | -0.12949 | -0.10496 | -1.59115 | 0.116366 | 0.501052 | -4.69217 |
| MIKKELSEN_MCV6_HCP_WITH_H3K27ME3 | 0.096021 | -0.04196 | 1.589049 | 0.116839 | 0.502378 | -4.69515 |
| REACTOME_VOLTAGE_GATED_POTASSIUM_CHANNELS | 0.132737 | 0.028854 | 1.58891 | 0.11687 | 0.502378 | -4.69535 |
| REACTOME_FCGR_ACTIVATION | 0.145121 | ####### | 1.588359 | 0.116995 | 0.502378 | -4.69613 |
| JU_AGING_TERC_TARGETS_UP | 0.132576 | 0.01932 | 1.588339 | 0.117 | 0.502378 | -4.69616 |
| BOUDOUKHA_BOUND_BY_IGF2BP2 | -0.11405 | -0.03289 | -1.58803 | 0.117069 | 0.502378 | -4.6966 |
| MIKKELSEN_ES_ICP_WITH_H3K27ME3 | 0.070434 | -0.20246 | 1.587158 | 0.117267 | 0.502671 | -4.69784 |
| SCHAEFFER_PROSTATE_DEVELOPMENT_AND_CANCER_BOX4_UP | -0.15064 | 0.015965 | -1.58703 | 0.117295 | 0.502671 | -4.69802 |
| KIM_BIPOLAR_DISORDER_OLIGODENDROCYTE_DENSITY_CORR_DN | 0.052401 | -0.30844 | 1.586608 | 0.117392 | 0.502746 | -4.69862 |
| AMIT_EGF_RESPONSE_480_HELA | 0.090195 | -0.08578 | 1.585434 | 0.117658 | 0.503289 | -4.70029 |
| MARCHINI_TRABECTEDIN_RESISTANCE_DN | 0.097647 | -0.12507 | 1.585043 | 0.117747 | 0.503289 | -4.70085 |
| HENDRICKS_SMARCA4_TARGETS_UP | 0.079249 | -0.05583 | 1.58495 | 0.117768 | 0.503289 | -4.70098 |
| REACTOME_ABC_TRANSPORTER_DISORDERS | -0.07978 | -0.24933 | -1.58466 | 0.117835 | 0.503289 | -4.70139 |
| PID_TGFBR_PATHWAY | -0.08047 | -0.11291 | -1.58411 | 0.117959 | 0.50348 | -4.70216 |
| MEISSNER_NPC_HCP_WITH_H3K4ME2_AND_H3K27ME3 | 0.096287 | -0.04371 | 1.583712 | 0.11805 | 0.503533 | -4.70273 |
| BLANCO_MELO_BETA_INTERFERON_TREATED_BRONCHIAL_EPITHELIAL_CELLS_DN | 0.067664 | -0.05429 | 1.582602 | 0.118303 | 0.50367 | -4.70431 |
| BEGUM_TARGETS_OF_PAX3_FOXO1_FUSION_UP | -0.10295 | -0.05908 | -1.5824 | 0.118348 | 0.50367 | -4.70459 |
| VETTER_TARGETS_OF_PRKCA_AND_ETS1_UP | -0.09863 | -0.00757 | -1.58232 | 0.118368 | 0.50367 | -4.70471 |
| REACTOME_DIGESTION_AND_ABSORPTION | 0.098806 | 0.012106 | 1.581997 | 0.118441 | 0.50367 | -4.70516 |
| REACTOME_FORMATION_OF_XYLULOSE_5_PHOSPHATE | 0.135357 | -0.03612 | 1.581289 | 0.118603 | 0.50367 | -4.70617 |
| REACTOME_DEPOSITION_OF_NEW_CENPA_CONTAINING_NUCLEOSOMES_AT_THE_CENTROMERE | -0.09509 | -0.03364 | -1.58126 | 0.11861 | 0.50367 | -4.70621 |
| ZEILSTRA_CD44_TARGETS_UP | 0.132828 | -0.11358 | 1.581074 | 0.118652 | 0.50367 | -4.70647 |
| WP_TGIF_DISRUPTION_OF_SHH_SIGNALING | 0.139411 | -0.01021 | 1.580715 | 0.118735 | 0.50367 | -4.70698 |
| DIRMEIER_LMP1_RESPONSE_LATE_DN | 0.054764 | -0.47784 | 1.580379 | 0.118811 | 0.50367 | -4.70746 |
| MAYBURD_RESPONSE_TO_L663536_DN | -0.11866 | -0.05161 | -1.57956 | 0.118999 | 0.50367 | -4.70862 |
| BIOCARTA_TCAPOPTOSIS_PATHWAY | 0.152288 | -0.01513 | 1.579398 | 0.119036 | 0.50367 | -4.70884 |
| REACTOME_RESOLUTION_OF_SISTER_CHROMATID_COHESION | -0.08947 | -0.03785 | -1.57908 | 0.11911 | 0.50367 | -4.7093 |
| REACTOME_ACTIVATED_NTRK2_SIGNALS_THROUGH_RAS | -0.11389 | -0.09564 | -1.57907 | 0.119111 | 0.50367 | -4.70931 |
| SMID_BREAST_CANCER_RELAPSE_IN_BRAIN_DN | -0.04544 | -0.18125 | -1.57852 | 0.119239 | 0.503875 | -4.71009 |
| TIEN_INTESTINE_PROBIOTICS_24HR_UP | -0.11277 | -0.05916 | -1.578 | 0.119358 | 0.503939 | -4.71083 |
| REACTOME_NEF_MEDIATES_DOWN_MODULATION_OF_CELL_SURFACE_RECEPTORS_BY_RECRUITING_THEM_TO_CLATHRIN_ADAPTERS | 0.116786 | -0.08361 | 1.577424 | 0.11949 | 0.503939 | -4.71163 |
| WP_IRON_METABOLISM_IN_PLACENTA | 0.146003 | -0.02087 | 1.577417 | 0.119491 | 0.503939 | -4.71164 |
| MOTAMED_RESPONSE_TO_ANDROGEN_DN | -0.13662 | -0.00143 | -1.57585 | 0.119853 | 0.505128 | -4.71386 |
| REACTOME_PTEN_REGULATION | -0.07522 | -0.20848 | -1.57242 | 0.120646 | 0.506926 | -4.7187 |
| ZHAN_MULTIPLE_MYELOMA_HP_UP | -0.08424 | -0.00029 | -1.5724 | 0.120651 | 0.506926 | -4.71873 |
| FIGUEROA_AML_METHYLATION_CLUSTER_5_DN | 0.06012 | -0.11211 | 1.572306 | 0.120672 | 0.506926 | -4.71886 |
| KAUFFMANN_DNA_REPAIR_GENES | -0.06964 | -0.10052 | -1.57225 | 0.120685 | 0.506926 | -4.71893 |
| GRABARCZYK_BCL11B_TARGETS_UP | -0.09659 | -0.03071 | -1.57219 | 0.120699 | 0.506926 | -4.71902 |
| REACTOME_REGULATION_OF_GENE_EXPRESSION_IN_LATE_STAGE_BRANCHING_MORPHOGENESIS_PANCREATIC_BUD_PRECURSOR_CELLS | 0.069288 | -0.10019 | 1.571938 | 0.120757 | 0.506926 | -4.71937 |
| LOCKWOOD_AMPLIFIED_IN_LUNG_CANCER | -0.08738 | -0.07342 | -1.57096 | 0.120984 | 0.507302 | -4.72075 |
| BIOCARTA_NUCLEARRS_PATHWAY | 0.094636 | -0.03521 | 1.570639 | 0.121059 | 0.507302 | -4.7212 |
| KLEIN_TARGETS_OF_BCR_ABL1_FUSION | 0.103421 | -0.04315 | 1.570523 | 0.121086 | 0.507302 | -4.72137 |
| WP_PYRIMIDINE_METABOLISM | -0.0957 | -0.032 | -1.56946 | 0.121333 | 0.507834 | -4.72286 |
| BIOCARTA_HES_PATHWAY | 0.086987 | -0.07979 | 1.569043 | 0.121431 | 0.507834 | -4.72345 |
| REACTOME_LTC4_CYSLTR_MEDIATED_IL4_PRODUCTION | 0.129337 | 0.004488 | 1.568649 | 0.121522 | 0.507834 | -4.724 |
| WP_NANOPARTICLE_TRIGGERED_AUTOPHAGIC_CELL_DEATH | -0.09815 | -0.05783 | -1.56861 | 0.121532 | 0.507834 | -4.72406 |
| REACTOME_COOPERATION_OF_PDCL_PHLP1_AND_TRIC_CCT_IN_G_PROTEIN_BETA_FOLDING | -0.08138 | -0.14987 | -1.56806 | 0.12166 | 0.508035 | -4.72483 |
| WANG_LMO4_TARGETS_DN | -0.1007 | -0.08448 | -1.56757 | 0.121775 | 0.50818 | -4.72552 |
| BERENJENO_TRANSFORMED_BY_RHOA_REVERSIBLY_UP | 0.082847 | -0.17451 | 1.567177 | 0.121866 | 0.508228 | -4.72607 |
| MATZUK_FERTILIZATION | 0.097586 | -0.09952 | 1.56675 | 0.121966 | 0.508311 | -4.72667 |
| REACTOME_PROCESSING_OF_INTRONLESS_PRE_MRNAS | -0.13035 | -0.06375 | -1.56613 | 0.122112 | 0.508586 | -4.72754 |
| DODD_NASOPHARYNGEAL_CARCINOMA_DN | -0.09531 | -0.06797 | -1.56411 | 0.122585 | 0.510222 | -4.73037 |
| REACTOME_PROTEIN_REPAIR | -0.18854 | -0.13267 | -1.56317 | 0.122804 | 0.510351 | -4.73168 |
| WP_PPAR_SIGNALING_PATHWAY | 0.063486 | -0.08879 | 1.563102 | 0.122821 | 0.510351 | -4.73179 |
| BIOCARTA_AKAP13_PATHWAY | -0.07814 | -0.08852 | -1.56295 | 0.122856 | 0.510351 | -4.73199 |
| WALLACE_PROSTATE_CANCER_RACE_UP | 0.065937 | -0.25841 | 1.560891 | 0.123343 | 0.512023 | -4.73488 |
| REACTOME_TRANSPORT_OF_ORGANIC_ANIONS | 0.114339 | 0.004424 | 1.560564 | 0.12342 | 0.512023 | -4.73534 |
| HOLLEMAN_PREDNISOLONE_RESISTANCE_B_ALL_UP | -0.11987 | -0.11689 | -1.5593 | 0.123719 | 0.512224 | -4.73711 |
| DAZARD_RESPONSE_TO_UV_NHEK_DN | -0.08827 | -0.08855 | -1.55808 | 0.124008 | 0.512224 | -4.73882 |
| REACTOME_MTOR_SIGNALLING | -0.10569 | -0.00436 | -1.55806 | 0.124013 | 0.512224 | -4.73884 |
| HU_GENOTOXIN_ACTION_DIRECT_VS_INDIRECT_4HR | -0.0836 | -0.02865 | -1.55722 | 0.124211 | 0.512224 | -4.74001 |
| REACTOME_APC_C_CDH1_MEDIATED_DEGRADATION_OF_CDC20_AND_OTHER_APC_C_CDH1_TARGETED_PROTEINS_IN_LATE_MITOSIS_EARLY_G1 | -0.10203 | -0.19154 | -1.55718 | 0.12422 | 0.512224 | -4.74006 |
| WP_PURINERGIC_SIGNALING | 0.071159 | 0.011888 | 1.556879 | 0.124292 | 0.512224 | -4.74049 |
| CHYLA_CBFA2T3_TARGETS_DN | 0.042605 | -0.10538 | 1.556803 | 0.12431 | 0.512224 | -4.7406 |
| TENEDINI_MEGAKARYOCYTE_MARKERS | 0.060681 | -0.13865 | 1.556708 | 0.124333 | 0.512224 | -4.74073 |
| REACTOME_HUR_ELAVL1_BINDS_AND_STABILIZES_MRNA | -0.1672 | -0.00931 | -1.55669 | 0.124336 | 0.512224 | -4.74075 |
| HECKER_IFNB1_TARGETS | 0.083803 | -0.09448 | 1.556387 | 0.124409 | 0.512224 | -4.74118 |
| BIOCARTA_CHREBP_PATHWAY | -0.08315 | -0.04956 | -1.55593 | 0.124518 | 0.512224 | -4.74182 |
| FUNG_IL2_TARGETS_WITH_STAT5_BINDING_SITES_T1 | 0.087929 | -0.18253 | 1.555883 | 0.124529 | 0.512224 | -4.74188 |
| BYSTRYKH_HEMATOPOIESIS_STEM_CELL_RUNX1 | 0.092596 | 0.012047 | 1.55568 | 0.124577 | 0.512224 | -4.74216 |
| WP_DNA_IRDOUBLE_STRAND_BREAKS_AND_CELLULAR_RESPONSE_VIA_ATM | -0.05474 | -0.18111 | -1.55559 | 0.124598 | 0.512224 | -4.74229 |
| ZHAN_MULTIPLE_MYELOMA_MS_DN | -0.0816 | -0.04983 | -1.55527 | 0.124676 | 0.512224 | -4.74274 |
| MOLENAAR_TARGETS_OF_CCND1_AND_CDK4_DN | -0.08338 | -0.01844 | -1.55434 | 0.124897 | 0.512647 | -4.74403 |
| RODRIGUES_THYROID_CARCINOMA_UP | -0.13104 | -0.00758 | -1.55416 | 0.12494 | 0.512647 | -4.74428 |
| WEBER_METHYLATED_LCP_IN_FIBROBLAST_UP | 0.153357 | 0.010066 | 1.552438 | 0.12535 | 0.513929 | -4.74668 |
| REACTOME_ENERGY_DEPENDENT_REGULATION_OF_MTOR_BY_LKB1_AMPK | -0.11153 | -0.00715 | -1.55217 | 0.125414 | 0.513929 | -4.74705 |
| PUJANA_BRCA2_PCC_NETWORK | -0.09207 | -0.1094 | -1.55143 | 0.125591 | 0.514162 | -4.74808 |
| REACTOME_CD163_MEDIATING_AN_ANTI_INFLAMMATORY_RESPONSE | 0.111249 | 0.007079 | 1.551154 | 0.125657 | 0.514162 | -4.74847 |
| PAL_PRMT5_TARGETS_UP | -0.09113 | -0.13193 | -1.55092 | 0.125713 | 0.514162 | -4.74879 |
| CHEMNITZ_RESPONSE_TO_PROSTAGLANDIN_E2_UP | -0.07498 | -0.11505 | -1.54986 | 0.125967 | 0.514461 | -4.75027 |
| RYAN_MANTLE_CELL_LYMPHOMA_NOTCH_DIRECT_UP | 0.078239 | -0.09714 | 1.549656 | 0.126017 | 0.514461 | -4.75055 |
| REACTOME_SIGNALING_BY_FGFR2_IIIA_TM | -0.14966 | -0.01232 | -1.54961 | 0.126028 | 0.514461 | -4.75062 |
| REACTOME_SOS_MEDIATED_SIGNALLING | -0.14182 | -0.09368 | -1.54877 | 0.126229 | 0.514878 | -4.75178 |
| BOYAULT_LIVER_CANCER_SUBCLASS_G5_DN | 0.079436 | -0.32257 | 1.548314 | 0.126339 | 0.514878 | -4.75241 |
| PYEON_CANCER_HEAD_AND_NECK_VS_CERVICAL_UP | -0.06331 | -0.08182 | -1.54736 | 0.126568 | 0.514878 | -4.75374 |
| PID_TAP63_PATHWAY | 0.063048 | -0.01865 | 1.546195 | 0.12685 | 0.514878 | -4.75536 |
| NIKOLSKY_BREAST_CANCER_19P13_AMPLICON | -0.13749 | -0.00884 | -1.5459 | 0.12692 | 0.514878 | -4.75576 |
| LIANG_SILENCED_BY_METHYLATION_UP | 0.068749 | -0.14088 | 1.545771 | 0.126952 | 0.514878 | -4.75594 |
| REACTOME_TP53_REGULATES_TRANSCRIPTION_OF_DEATH_RECEPTORS_AND_LIGANDS | 0.103766 | -0.01252 | 1.54517 | 0.127097 | 0.514878 | -4.75678 |
| WONG_PROTEASOME_GENE_MODULE | -0.10997 | -0.1795 | -1.54484 | 0.127178 | 0.514878 | -4.75724 |
| WONG_ENDMETRIUM_CANCER_UP | 0.082346 | 0.010222 | 1.544008 | 0.127379 | 0.514878 | -4.75839 |
| KIM_WT1_TARGETS_8HR_DN | -0.04955 | -0.19576 | -1.54372 | 0.127448 | 0.514878 | -4.75879 |
| WP_FAMILIAL_HYPERLIPIDEMIA_TYPE_4 | 0.091188 | 0.009627 | 1.543672 | 0.12746 | 0.514878 | -4.75885 |
| REACTOME_TP53_REGULATES_TRANSCRIPTION_OF_CELL_CYCLE_GENES | 0.073138 | -0.14709 | 1.543559 | 0.127487 | 0.514878 | -4.75901 |
| REACTOME_POTASSIUM_CHANNELS | 0.087354 | -0.04811 | 1.543375 | 0.127532 | 0.514878 | -4.75927 |
| MCBRYAN_PUBERTAL_BREAST_5_6WK_DN | -0.05824 | -0.15772 | -1.54328 | 0.127554 | 0.514878 | -4.75939 |
| REACTOME_CELLULAR_RESPONSE_TO_HEAT_STRESS | -0.09365 | -0.11712 | -1.54294 | 0.127637 | 0.514878 | -4.75987 |
| SANSOM_APC_TARGETS_REQUIRE_MYC | -0.06565 | -0.0966 | -1.54263 | 0.127712 | 0.514878 | -4.76029 |
| TCGA_GLIOBLASTOMA_COPY_NUMBER_UP | -0.06948 | -0.04382 | -1.54254 | 0.127735 | 0.514878 | -4.76042 |
| TURASHVILI_BREAST_NORMAL_DUCTAL_VS_LOBULAR_UP | -0.08453 | -0.03252 | -1.54242 | 0.127763 | 0.514878 | -4.76059 |
| BIOCARTA_NO2IL12_PATHWAY | 0.139912 | -0.0165 | 1.542028 | 0.127859 | 0.514878 | -4.76113 |
| BENPORATH_ES_WITH_H3K27ME3 | 0.06576 | -0.0897 | 1.541781 | 0.127919 | 0.514878 | -4.76147 |
| REACTOME_MITOTIC_PROPHASE | -0.11924 | -0.02794 | -1.54135 | 0.128024 | 0.514878 | -4.76207 |
| LOPEZ_MESOTHELIOMA_SURVIVAL_UP | 0.105145 | -0.01047 | 1.541275 | 0.128042 | 0.514878 | -4.76217 |
| WP_ACTIVATION_OF_NLRP3_INFLAMMASOME_BY_SARSCOV2 | 0.151318 | 0.03417 | 1.541212 | 0.128057 | 0.514878 | -4.76226 |
| DACOSTA_UV_RESPONSE_VIA_ERCC3_TTD_UP | 0.064798 | -0.12986 | 1.541148 | 0.128072 | 0.514878 | -4.76235 |
| REACTOME_DOWNSTREAM_SIGNALING_OF_ACTIVATED_FGFR3 | -0.06405 | -0.0262 | -1.54022 | 0.128297 | 0.515457 | -4.76362 |
| PID_P38_ALPHA_BETA_PATHWAY | 0.06583 | -0.12782 | 1.539578 | 0.128455 | 0.515517 | -4.76452 |
| KEGG_PARKINSONS_DISEASE | -0.09542 | -0.13033 | -1.5395 | 0.128474 | 0.515517 | -4.76463 |
| BOYAULT_LIVER_CANCER_SUBCLASS_G3_UP | -0.1094 | -0.11089 | -1.53874 | 0.12866 | 0.515935 | -4.76568 |
| QI_HYPOXIA_TARGETS_OF_HIF1A_AND_FOXA2 | 0.05525 | -0.39394 | 1.538179 | 0.128796 | 0.516157 | -4.76645 |
| SHEDDEN_LUNG_CANCER_GOOD_SURVIVAL_A5 | -0.06318 | -0.05845 | -1.53583 | 0.12937 | 0.517643 | -4.76968 |
| REACTOME_ERKS_ARE_INACTIVATED | 0.108512 | 0.006617 | 1.5356 | 0.129427 | 0.517643 | -4.77001 |
| EPPERT_PROGENITOR | -0.08384 | -0.0498 | -1.53554 | 0.129441 | 0.517643 | -4.77008 |
| MUNSHI_MULTIPLE_MYELOMA_UP | 0.07964 | -0.12239 | 1.534916 | 0.129595 | 0.517643 | -4.77095 |
| REACTOME_SIGNALING_BY_INTERLEUKINS | 0.064638 | -0.09654 | 1.534378 | 0.129727 | 0.517643 | -4.77169 |
| HOEGERKORP_CD44_TARGETS_TEMPORAL_UP | 0.081648 | 0.021182 | 1.534107 | 0.129794 | 0.517643 | -4.77206 |
| PID_SYNDECAN_2_PATHWAY | 0.067609 | -0.08073 | 1.533935 | 0.129836 | 0.517643 | -4.7723 |
| WINZEN_DEGRADED_VIA_KHSRP | 0.076565 | -0.11258 | 1.533691 | 0.129896 | 0.517643 | -4.77264 |
| REACTOME_ACTIVATION_OF_PUMA_AND_TRANSLOCATION_TO_MITOCHONDRIA | 0.094111 | -0.01011 | 1.533075 | 0.130048 | 0.517643 | -4.77348 |
| RAO_BOUND_BY_SALL4_ISOFORM_B | -0.04942 | -0.09193 | -1.53238 | 0.130218 | 0.517643 | -4.77444 |
| REACTOME_DIGESTION_OF_DIETARY_LIPID | 0.159713 | 0.020374 | 1.532223 | 0.130257 | 0.517643 | -4.77466 |
| WP_TCA_CYCLE_AND_DEFICIENCY_OF_PYRUVATE_DEHYDROGENASE_COMPLEX_PDHC | -0.1513 | -0.00152 | -1.53217 | 0.13027 | 0.517643 | -4.77472 |
| ABDELMOHSEN_ELAVL4_TARGETS | -0.12699 | -0.13278 | -1.53194 | 0.130327 | 0.517643 | -4.77504 |
| TSUNODA_CISPLATIN_RESISTANCE_DN | 0.056169 | -0.25731 | 1.531653 | 0.130398 | 0.517643 | -4.77544 |
| WEBER_METHYLATED_ICP_IN_SPERM_DN | 0.125252 | -0.22163 | 1.531381 | 0.130465 | 0.517643 | -4.77581 |
| MALTA_CURATED_STEMNESS_MARKERS | 0.082453 | -0.20222 | 1.531369 | 0.130468 | 0.517643 | -4.77583 |
| LEE_LIVER_CANCER_E2F1_UP | 0.081448 | -0.05495 | 1.530307 | 0.13073 | 0.517732 | -4.77729 |
| MEISSNER_BRAIN_HCP_WITH_H3K4ME2_AND_H3K27ME3 | 0.083259 | -0.0506 | 1.530138 | 0.130772 | 0.517732 | -4.77752 |
| MARKEY_RB1_ACUTE_LOF_DN | 0.066545 | -0.24905 | 1.529867 | 0.130839 | 0.517732 | -4.77789 |
| KIM_GERMINAL_CENTER_T_HELPER_UP | -0.12879 | -0.02467 | -1.52973 | 0.130873 | 0.517732 | -4.77808 |
| LEIN_LOCALIZED_TO_PROXIMAL_DENDRITES | 0.065528 | -0.11446 | 1.529632 | 0.130897 | 0.517732 | -4.77822 |
| GARGALOVIC_RESPONSE_TO_OXIDIZED_PHOSPHOLIPIDS_PURPLE_DN | 0.109976 | 0.02366 | 1.528784 | 0.131107 | 0.518096 | -4.77938 |
| REACTOME_ABORTIVE_ELONGATION_OF_HIV_1_TRANSCRIPT_IN_THE_ABSENCE_OF_TAT | -0.124 | -0.17793 | -1.5286 | 0.131152 | 0.518096 | -4.77963 |
| REACTOME_TRYPTOPHAN_CATABOLISM | 0.089193 | -0.05318 | 1.528026 | 0.131295 | 0.518225 | -4.78042 |
| HERNANDEZ_MITOTIC_ARREST_BY_DOCETAXEL_2_UP | 0.08129 | -0.1389 | 1.527585 | 0.131404 | 0.518225 | -4.78103 |
| KEGG_PORPHYRIN_AND_CHLOROPHYLL_METABOLISM | 0.066751 | -0.07844 | 1.527414 | 0.131447 | 0.518225 | -4.78126 |
| REACTOME_HIV_LIFE_CYCLE | -0.10342 | -0.1099 | -1.52716 | 0.131511 | 0.518225 | -4.78161 |
| VALK_AML_CLUSTER_8 | -0.08911 | -0.03739 | -1.5261 | 0.131774 | 0.518692 | -4.78306 |
| BHAT_ESR1_TARGETS_NOT_VIA_AKT1_UP | 0.048969 | -0.03675 | 1.526025 | 0.131792 | 0.518692 | -4.78317 |
| WP_NEURAL_CREST_DIFFERENTIATION | 0.053962 | -0.13352 | 1.525327 | 0.131966 | 0.518862 | -4.78412 |
| REACTOME_SIGNALING_BY_WNT | -0.05873 | -0.11302 | -1.5252 | 0.131998 | 0.518862 | -4.7843 |
| REACTOME_SWITCHING_OF_ORIGINS_TO_A_POST_REPLICATIVE_STATE | -0.09123 | -0.16403 | -1.52332 | 0.132467 | 0.519754 | -4.78687 |
| WP_REGULATION_OF_SISTER_CHROMATID_SEPARATION_AT_THE_METAPHASEANAPHASE_TRANSITION | -0.12871 | -0.01115 | -1.52291 | 0.132568 | 0.519754 | -4.78742 |
| REACTOME_NR1H2_NR1H3_REGULATE_GENE_EXPRESSION_LINKED_TO_TRIGLYCERIDE_LIPOLYSIS_IN_ADIPOSE | 0.091718 | -0.45904 | 1.522781 | 0.132601 | 0.519754 | -4.78761 |
| REACTOME_TRANSLATION_OF_SARS_COV_1_STRUCTURAL_PROTEINS | 0.086893 | -0.04056 | 1.521671 | 0.132879 | 0.519754 | -4.78912 |
| MUELLER_PLURINET | -0.08603 | -0.09549 | -1.52162 | 0.132893 | 0.519754 | -4.7892 |
| JEON_SMAD6_TARGETS_UP | 0.064564 | -0.3452 | 1.521334 | 0.132963 | 0.519754 | -4.78958 |
| REACTOME_REGULATION_OF_GLUCOKINASE_BY_GLUCOKINASE_REGULATORY_PROTEIN | -0.12263 | -0.00043 | -1.52123 | 0.13299 | 0.519754 | -4.78973 |
| REACTOME_FOLDING_OF_ACTIN_BY_CCT_TRIC | -0.17289 | 0.001768 | -1.52041 | 0.133194 | 0.519754 | -4.79084 |
| LEE_AGING_CEREBELLUM_UP | 0.072345 | -0.10129 | 1.520354 | 0.133209 | 0.519754 | -4.79092 |
| LIU_TARGETS_OF_VMYB_VS_CMYB_UP | 0.083912 | -0.06124 | 1.519659 | 0.133383 | 0.519754 | -4.79187 |
| WP_HIJACK_OF_UBIQUITINATION_BY_SARSCOV2 | -0.19326 | -0.0358 | -1.51953 | 0.133415 | 0.519754 | -4.79204 |
| WP_GLYCOSAMINOGLYCAN_DEGRADATION | 0.104901 | -0.00642 | 1.519348 | 0.133461 | 0.519754 | -4.7923 |
| CHIARADONNA_NEOPLASTIC_TRANSFORMATION_CDC25_DN | 0.084028 | -0.09677 | 1.519332 | 0.133466 | 0.519754 | -4.79232 |
| REACTOME_POU5F1_OCT4_SOX2_NANOG_ACTIVATE_GENES_RELATED_TO_PROLIFERATION | 0.08884 | -0.32026 | 1.519022 | 0.133543 | 0.519754 | -4.79274 |
| FOSTER_KDM1A_TARGETS_UP | 0.03867 | -0.12099 | 1.518935 | 0.133565 | 0.519754 | -4.79286 |
| BIOCARTA_IL3_PATHWAY | 0.100925 | -0.05445 | 1.517867 | 0.133834 | 0.519754 | -4.79432 |
| REACTOME_REGULATION_OF_HSF1_MEDIATED_HEAT_SHOCK_RESPONSE | -0.09281 | -0.14199 | -1.51759 | 0.133904 | 0.519754 | -4.7947 |
| MOLENAAR_TARGETS_OF_CCND1_AND_CDK4_UP | -0.06973 | -0.071 | -1.51742 | 0.133947 | 0.519754 | -4.79493 |
| TAVAZOIE_METASTASIS | 0.053707 | -0.03557 | 1.517383 | 0.133956 | 0.519754 | -4.79498 |
| REACTOME_BIOLOGICAL_OXIDATIONS | 0.043134 | -0.04757 | 1.517198 | 0.134003 | 0.519754 | -4.79523 |
| WILLIAMS_ESR2_TARGETS_DN | -0.14983 | -0.02402 | -1.51706 | 0.134037 | 0.519754 | -4.79542 |
| REACTOME_INACTIVATION_OF_CSF3_G_CSF_SIGNALING | 0.120899 | 0.009689 | 1.516746 | 0.134117 | 0.519754 | -4.79584 |
| BIOCARTA_PITX2_PATHWAY | 0.077226 | -0.01632 | 1.516648 | 0.134141 | 0.519754 | -4.79598 |
| BOGNI_TREATMENT_RELATED_MYELOID_LEUKEMIA_UP | 0.059887 | -0.11486 | 1.516473 | 0.134185 | 0.519754 | -4.79622 |
| OHGUCHI_LIVER_HNF4A_TARGETS_UP | 0.058966 | -0.1523 | 1.515631 | 0.134398 | 0.520261 | -4.79736 |
| CHIANG_LIVER_CANCER_SUBCLASS_UNANNOTATED_DN | -0.11732 | -0.10143 | -1.51443 | 0.134702 | 0.520872 | -4.799 |
| CHEN_LVAD_SUPPORT_OF_FAILING_HEART_DN | 0.088762 | -0.04171 | 1.514126 | 0.134779 | 0.520872 | -4.79941 |
| WP_STEROID_BIOSYNTHESIS | 0.122112 | 0.005766 | 1.514037 | 0.134801 | 0.520872 | -4.79953 |
| BASSO_HAIRY_CELL_LEUKEMIA_DN | -0.15985 | -0.00613 | -1.51253 | 0.135184 | 0.521809 | -4.80158 |
| DESERT_PERIVENOUS_HEPATOCELLULAR_CARCINOMA_SUBCLASS_UP | -0.08135 | -0.00205 | -1.51213 | 0.135284 | 0.521809 | -4.80212 |
| PEREZ_TP53_AND_TP63_TARGETS | 0.048303 | -0.06597 | 1.511884 | 0.135347 | 0.521809 | -4.80246 |
| BALLIF_DEVELOPMENTAL_DISABILITY_P16_P12_DELETION | 0.099164 | -0.06695 | 1.51141 | 0.135468 | 0.521809 | -4.8031 |
| LU_EZH2_TARGETS_UP | -0.06021 | -0.18782 | -1.51125 | 0.135508 | 0.521809 | -4.80332 |
| MARIADASON_RESPONSE_TO_BUTYRATE_SULINDAC_6 | -0.07849 | -0.06869 | -1.51092 | 0.135591 | 0.521809 | -4.80376 |
| REACTOME_DEFECTIVE_CHST3_CAUSES_SEDCJD | 0.129073 | -0.01822 | 1.510436 | 0.135716 | 0.521809 | -4.80443 |
| SHIPP_DLBCL_CURED_VS_FATAL_UP | 0.075603 | -0.16844 | 1.510295 | 0.135751 | 0.521809 | -4.80462 |
| REACTOME_REGULATION_OF_BETA_CELL_DEVELOPMENT | 0.07622 | -0.06747 | 1.510176 | 0.135782 | 0.521809 | -4.80478 |
| BLANCO_MELO_COVID19_SARS_COV_2_INFECTION_A594_ACE2_EXPRESSING_CELLS_RUXOLITINIB_UP | 0.048575 | -0.12946 | 1.50937 | 0.135987 | 0.522283 | -4.80587 |
| MIKKELSEN_IPS_ICP_WITH_H3K4ME3_AND_H327ME3 | 0.053425 | -0.15134 | 1.508901 | 0.136107 | 0.522428 | -4.80651 |
| PURBEY_TARGETS_OF_CTBP1_NOT_SATB1_UP | -0.05402 | -0.09489 | -1.50672 | 0.136665 | 0.522986 | -4.80947 |
| WP_GDNFRET_SIGNALING_AXIS | 0.067848 | -0.14729 | 1.506559 | 0.136706 | 0.522986 | -4.80968 |
| WANG_RESPONSE_TO_FORSKOLIN_DN | -0.15398 | -0.01674 | -1.50639 | 0.136748 | 0.522986 | -4.80991 |
| REACTOME_CONDENSATION_OF_PROMETAPHASE_CHROMOSOMES | -0.13425 | -0.2991 | -1.50618 | 0.136802 | 0.522986 | -4.81019 |
| REACTOME_ION_CHANNEL_TRANSPORT | 0.041111 | -0.07655 | 1.506113 | 0.13682 | 0.522986 | -4.81029 |
| WP_TCA_CYCLE_AKA_KREBS_OR_CITRIC_ACID_CYCLE | -0.16147 | 0.013935 | -1.50608 | 0.136827 | 0.522986 | -4.81033 |
| HUTTMANN_B_CLL_POOR_SURVIVAL_DN | -0.08527 | -0.07225 | -1.50585 | 0.136887 | 0.522986 | -4.81064 |
| REACTOME_UCH_PROTEINASES | -0.08416 | -0.25702 | -1.50576 | 0.13691 | 0.522986 | -4.81076 |
| MIZUKAMI_HYPOXIA_DN | 0.152367 | 0.014333 | 1.505045 | 0.137094 | 0.523105 | -4.81173 |
| NABA_COLLAGENS | 0.099001 | -0.11305 | 1.505 | 0.137105 | 0.523105 | -4.81179 |
| TOYOTA_TARGETS_OF_MIR34B_AND_MIR34C | -0.05548 | -0.16424 | -1.50467 | 0.137189 | 0.52311 | -4.81223 |
| REACTOME_NUCLEAR_PORE_COMPLEX_NPC_DISASSEMBLY | -0.12638 | -0.00138 | -1.50423 | 0.137302 | 0.523228 | -4.81283 |
| REACTOME_CHROMOSOME_MAINTENANCE | -0.09 | -0.06355 | -1.5037 | 0.137439 | 0.523437 | -4.81355 |
| HUMMERICH_MALIGNANT_SKIN_TUMOR_DN | 0.077513 | -0.17222 | 1.502792 | 0.137673 | 0.524014 | -4.81478 |
| VANDESLUIS_COMMD1_TARGETS_GROUP_2_UP | 0.129634 | 0.002063 | 1.501058 | 0.13812 | 0.524854 | -4.81712 |
| REACTOME_PEPTIDE_HORMONE_METABOLISM | 0.048169 | -0.0197 | 1.500816 | 0.138183 | 0.524854 | -4.81745 |
| LOPEZ_MESOTELIOMA_SURVIVAL_TIME_DN | 0.159579 | 0.001676 | 1.500687 | 0.138216 | 0.524854 | -4.81762 |
| LEIN_MEDULLA_MARKERS | 0.043504 | -0.10535 | 1.500656 | 0.138224 | 0.524854 | -4.81766 |
| OSMAN_BLADDER_CANCER_DN | -0.09082 | -0.07904 | -1.50011 | 0.138366 | 0.524854 | -4.8184 |
| REACTOME_PLATELET_SENSITIZATION_BY_LDL | -0.10183 | 0.006871 | -1.49975 | 0.138458 | 0.524854 | -4.81888 |
| GRYDER_PAX3FOXO1_ENHANCERS_IN_TADS | -0.08527 | -0.04931 | -1.49937 | 0.138555 | 0.524854 | -4.81939 |
| HORIUCHI_WTAP_TARGETS_DN | -0.08978 | -0.06447 | -1.49929 | 0.138578 | 0.524854 | -4.81951 |
| UDAYAKUMAR_MED1_TARGETS_UP | -0.0881 | -0.03956 | -1.49891 | 0.138676 | 0.524854 | -4.82002 |
| REACTOME_MIRO_GTPASE_CYCLE | -0.11013 | -0.08932 | -1.49874 | 0.138719 | 0.524854 | -4.82024 |
| WP_CODEINE_AND_MORPHINE_METABOLISM | 0.103119 | -0.23843 | 1.496966 | 0.13918 | 0.526286 | -4.82263 |
| KANNAN_TP53_TARGETS_UP | 0.069247 | -0.07139 | 1.496647 | 0.139263 | 0.526287 | -4.82307 |
| KRIEG_KDM3A_TARGETS_NOT_HYPOXIA | -0.09902 | -0.07603 | -1.49615 | 0.139392 | 0.526377 | -4.82374 |
| WP_MIRNAS_INVOLVED_IN_DNA_DAMAGE_RESPONSE | 0.096284 | -0.00359 | 1.495919 | 0.139452 | 0.526377 | -4.82404 |
| DACOSTA_UV_RESPONSE_VIA_ERCC3_COMMON_DN | -0.10429 | -0.05067 | -1.49534 | 0.139602 | 0.526387 | -4.82482 |
| BIOCARTA_NKCELLS_PATHWAY | 0.072796 | -0.28468 | 1.49512 | 0.13966 | 0.526387 | -4.82512 |
| PURBEY_TARGETS_OF_CTBP1_AND_SATB1_DN | -0.05218 | -0.05645 | -1.49496 | 0.139703 | 0.526387 | -4.82534 |
| REACTOME_INHIBITION_OF_DNA_RECOMBINATION_AT_TELOMERE | -0.11249 | -0.17804 | -1.49378 | 0.14001 | 0.526745 | -4.82693 |
| LENAOUR_DENDRITIC_CELL_MATURATION_DN | 0.071597 | -0.26299 | 1.493699 | 0.14003 | 0.526745 | -4.82703 |
| BIOCARTA_DREAM_PATHWAY | -0.0716 | -0.06675 | -1.4936 | 0.140056 | 0.526745 | -4.82716 |
| WEBER_METHYLATED_HCP_IN_FIBROBLAST_UP | 0.13128 | -0.0104 | 1.49325 | 0.140147 | 0.526745 | -4.82763 |
| PID_CONE_PATHWAY | 0.121038 | -0.04847 | 1.491963 | 0.140484 | 0.526745 | -4.82936 |
| MIKKELSEN_NPC_ICP_WITH_H3K27ME3 | 0.101388 | -0.33724 | 1.491495 | 0.140606 | 0.526745 | -4.82999 |
| CHYLA_CBFA2T3_TARGETS_UP | 0.042536 | -0.04592 | 1.490787 | 0.140792 | 0.526745 | -4.83094 |
| REACTOME_MRNA_SPLICING | -0.10074 | -0.14232 | -1.49057 | 0.140848 | 0.526745 | -4.83123 |
| REACTOME_SUMOYLATION_OF_UBIQUITINYLATION_PROTEINS | -0.09728 | -0.1007 | -1.49005 | 0.140986 | 0.526745 | -4.83193 |
| WP_SYNAPTIC_SIGNALING_PATHWAYS_ASSOCIATED_WITH_AUTISM_SPECTRUM_DISORDER | -0.04987 | -0.09239 | -1.48956 | 0.141115 | 0.526745 | -4.83259 |
| DAIRKEE_CANCER_PRONE_RESPONSE_BPA | -0.10366 | -0.0234 | -1.48881 | 0.14131 | 0.526745 | -4.83359 |
| HEIDENBLAD_AMPLICON_12P11_12_UP | -0.0842 | -0.07371 | -1.48874 | 0.141329 | 0.526745 | -4.83368 |
| VART_KSHV_INFECTION_ANGIOGENIC_MARKERS_UP | 0.054767 | -0.11387 | 1.48871 | 0.141337 | 0.526745 | -4.83372 |
| DELACROIX_RARG_BOUND_MEF | 0.052132 | -0.10587 | 1.488401 | 0.141418 | 0.526745 | -4.83413 |
| REACTOME_CONSTITUTIVE_SIGNALING_BY_ABERRANT_PI3K_IN_CANCER | 0.050084 | 0.002139 | 1.487982 | 0.141529 | 0.526745 | -4.8347 |
| REACTOME_SIGNALING_BY_ROBO_RECEPTORS | -0.08907 | -0.13497 | -1.48753 | 0.141646 | 0.526745 | -4.83529 |
| BIOCARTA_ION_PATHWAY | -0.1464 | -0.01295 | -1.48715 | 0.141746 | 0.526745 | -4.8358 |
| BAKER_HEMATOPOESIS_STAT5_TARGETS | 0.146701 | 0.018979 | 1.486693 | 0.141868 | 0.526745 | -4.83642 |
| MARSON_BOUND_BY_E2F4_UNSTIMULATED | -0.06034 | -0.19672 | -1.48659 | 0.141894 | 0.526745 | -4.83655 |
| GARY_CD5_TARGETS_DN | -0.10134 | -0.10909 | -1.48594 | 0.142067 | 0.526745 | -4.83743 |
| DARWICHE_SKIN_TUMOR_PROMOTER_UP | 0.057089 | -0.04176 | 1.485748 | 0.142117 | 0.526745 | -4.83768 |
| REACTOME_ASSEMBLY_OF_COLLAGEN_FIBRILS_AND_OTHER_MULTIMERIC_STRUCTURES | 0.093964 | -0.07656 | 1.48559 | 0.142159 | 0.526745 | -4.83789 |
| CHIARADONNA_NEOPLASTIC_TRANSFORMATION_KRAS_CDC25_UP | 0.067657 | -0.13904 | 1.485239 | 0.142252 | 0.526745 | -4.83836 |
| BIOCARTA_NDKDYNAMIN_PATHWAY | -0.14116 | -0.00271 | -1.48512 | 0.142283 | 0.526745 | -4.83852 |
| REACTOME_RSK_ACTIVATION | -0.12721 | -0.1105 | -1.48504 | 0.142306 | 0.526745 | -4.83864 |
| JOHNSTONE_PARVB_TARGETS_1_DN | -0.09447 | -0.12214 | -1.48446 | 0.142458 | 0.526745 | -4.83941 |
| WP_GASTRIC_CANCER_NETWORK_2 | -0.09314 | -0.03681 | -1.48429 | 0.142503 | 0.526745 | -4.83963 |
| BANDRES_RESPONSE_TO_CARMUSTIN_WITHOUT_MGMT_24HR_DN | -0.10982 | -0.00157 | -1.48402 | 0.142574 | 0.526745 | -4.83999 |
| AMIT_SERUM_RESPONSE_120_MCF10A | 0.090124 | -0.07324 | 1.483779 | 0.142638 | 0.526745 | -4.84031 |
| MARZEC_IL2_SIGNALING_UP | 0.066617 | -0.12269 | 1.483623 | 0.142679 | 0.526745 | -4.84052 |
| JOHNSTONE_PARVB_TARGETS_2_DN | -0.08611 | -0.15581 | -1.48301 | 0.142841 | 0.526745 | -4.84134 |
| REACTOME_SLC_MEDIATED_TRANSMEMBRANE_TRANSPORT | 0.047341 | -0.073 | 1.482634 | 0.142941 | 0.526745 | -4.84184 |
| DUNNE_TARGETS_OF_AML1_MTG8_FUSION_UP | 0.07772 | -0.17717 | 1.482344 | 0.143018 | 0.526745 | -4.84223 |
| PUJANA_CHEK2_PCC_NETWORK | -0.09682 | -0.12041 | -1.48214 | 0.143072 | 0.526745 | -4.8425 |
| CLIMENT_BREAST_CANCER_COPY_NUMBER_DN | 0.106351 | -0.10019 | 1.481935 | 0.143127 | 0.526745 | -4.84278 |
| REACTOME_TYPE_I_HEMIDESMOSOME_ASSEMBLY | 0.111013 | 0.026148 | 1.481874 | 0.143143 | 0.526745 | -4.84286 |
| BAUS_TFF2_TARGETS_DN | 0.098362 | -0.34329 | 1.481637 | 0.143206 | 0.526745 | -4.84317 |
| REACTOME_ACTIVATED_PKN1_STIMULATES_TRANSCRIPTION_OF_AR_ANDROGEN_RECEPTOR_REGULATED_GENES_KLK2_AND_KLK3 | 0.111772 | -0.00375 | 1.48144 | 0.143259 | 0.526745 | -4.84344 |
| REACTOME_PRE_NOTCH_PROCESSING_IN_THE_ENDOPLASMIC_RETICULUM | -0.1117 | -0.29278 | -1.48139 | 0.143271 | 0.526745 | -4.8435 |
| WP_SCFA_AND_SKELETAL_MUSCLE_SUBSTRATE_METABOLISM | 0.134844 | -0.12686 | 1.480805 | 0.143427 | 0.526745 | -4.84428 |
| LEE_AGING_NEOCORTEX_UP | 0.072877 | -0.18173 | 1.480805 | 0.143427 | 0.526745 | -4.84428 |
| REACTOME_GAMMA_CARBOXYLATION_HYPUSINE_FORMATION_AND_ARYLSULFATASE_ACTIVATION | -0.07148 | -0.02459 | -1.48008 | 0.143621 | 0.526745 | -4.84525 |
| REACTOME_NUCLEAR_ENVELOPE_NE_REASSEMBLY | -0.09645 | -0.02839 | -1.47985 | 0.143681 | 0.526745 | -4.84555 |
| REACTOME_DEFECTIVE_CHSY1_CAUSES_TPBS | 0.125793 | -0.02321 | 1.479741 | 0.14371 | 0.526745 | -4.8457 |
| PID_INTEGRIN1_PATHWAY | 0.096273 | -0.08458 | 1.479498 | 0.143775 | 0.526745 | -4.84602 |
| BIOCARTA_P27_PATHWAY | -0.13545 | -0.0822 | -1.47948 | 0.143779 | 0.526745 | -4.84604 |
| LI_WILMS_TUMOR_VS_FETAL_KIDNEY_2_DN | 0.099508 | -0.02808 | 1.47905 | 0.143895 | 0.526745 | -4.84662 |
| WP_HOMOLOGOUS_RECOMBINATION | -0.10459 | 0.00328 | -1.47873 | 0.14398 | 0.526745 | -4.84704 |
| MATZUK_EMBRYONIC_GERM_CELL | -0.07475 | -0.05512 | -1.47867 | 0.143996 | 0.526745 | -4.84713 |
| SANSOM_APC_TARGETS | -0.03977 | -0.05304 | -1.4784 | 0.144067 | 0.526745 | -4.84748 |
| REACTOME_SODIUM_PROTON_EXCHANGERS | 0.081887 | -0.00936 | 1.47825 | 0.144108 | 0.526745 | -4.84768 |
| YAMANAKA_GLIOBLASTOMA_SURVIVAL_DN | 0.068975 | -0.4495 | 1.477969 | 0.144183 | 0.526745 | -4.84806 |
| BLALOCK_ALZHEIMERS_DISEASE_INCIPIENT_DN | -0.08675 | -0.11173 | -1.47796 | 0.144184 | 0.526745 | -4.84806 |
| CHEOK_RESPONSE_TO_MERCAPTOPURINE_AND_HD_MTX_UP | -0.12215 | -0.00982 | -1.47668 | 0.144528 | 0.527698 | -4.84978 |
| HOUNKPE_HOUSEKEEPING_GENES | -0.1123 | -0.10602 | -1.47578 | 0.144768 | 0.52818 | -4.85096 |
| BARRIER_COLON_CANCER_RECURRENCE_UP | -0.12288 | -0.03214 | -1.47556 | 0.144826 | 0.52818 | -4.85125 |
| REACTOME_POLYMERASE_SWITCHING_ON_THE_C_STRAND_OF_THE_TELOMERE | -0.10456 | -0.04276 | -1.47341 | 0.145403 | 0.529741 | -4.85411 |
| REACTOME_ACTIVATED_NTRK2_SIGNALS_THROUGH_FYN | 0.106142 | -0.01952 | 1.473349 | 0.145421 | 0.529741 | -4.85419 |
| REACTOME_PROCESSING_OF_CAPPED_INTRON_CONTAINING_PRE_MRNA | -0.09728 | -0.13725 | -1.47192 | 0.145804 | 0.530152 | -4.85608 |
| MIKKELSEN_MEF_HCP_WITH_H3K27ME3 | 0.0956 | -0.07762 | 1.471762 | 0.145848 | 0.530152 | -4.8563 |
| GENTILE_UV_RESPONSE_CLUSTER_D9 | -0.13498 | -0.04078 | -1.47149 | 0.145922 | 0.530152 | -4.85666 |
| WP_GPCRS_CLASS_C_METABOTROPIC_GLUTAMATE_PHEROMONE | 0.108125 | -0.23668 | 1.4714 | 0.145946 | 0.530152 | -4.85678 |
| REACTOME_RECRUITMENT_OF_MITOTIC_CENTROSOME_PROTEINS_AND_COMPLEXES | -0.06688 | -0.13311 | -1.47121 | 0.145997 | 0.530152 | -4.85703 |
| REACTOME_SUMOYLATION_OF_SUMOYLATION_PROTEINS | -0.12026 | 0.000325 | -1.47107 | 0.146033 | 0.530152 | -4.85721 |
| REACTOME_LDL_REMODELING | -0.10403 | -0.00299 | -1.47051 | 0.146185 | 0.530398 | -4.85795 |
| RODRIGUES_THYROID_CARCINOMA_ANAPLASTIC_DN | -0.07892 | -0.06137 | -1.46845 | 0.146742 | 0.532116 | -4.86068 |
| REACTOME_ION_HOMEOSTASIS | -0.06312 | -0.01229 | -1.46781 | 0.146915 | 0.532442 | -4.86153 |
| FOURNIER_ACINAR_DEVELOPMENT_LATE_UP | 0.104901 | -0.01554 | 1.466874 | 0.14717 | 0.53306 | -4.86276 |
| REACTOME_SIGNALING_BY_FGFR2 | -0.06413 | -0.03962 | -1.46594 | 0.147423 | 0.533576 | -4.864 |
| REACTOME_PHOSPHATE_BOND_HYDROLYSIS_BY_NTPDASE_PROTEINS | 0.098178 | 0.023918 | 1.465732 | 0.14748 | 0.533576 | -4.86427 |
| WHITESIDE_CISPLATIN_RESISTANCE_UP | 0.118569 | -0.00802 | 1.464949 | 0.147693 | 0.534043 | -4.86531 |
| KEGG_VALINE_LEUCINE_AND_ISOLEUCINE_DEGRADATION | -0.12411 | -0.05351 | -1.4645 | 0.147816 | 0.534055 | -4.8659 |
| BIOCARTA_P38MAPK_PATHWAY | -0.09051 | -0.12526 | -1.46416 | 0.147908 | 0.534055 | -4.86635 |
| REACTOME_FOXO_MEDIATED_TRANSCRIPTION_OF_CELL_DEATH_GENES | -0.10315 | -0.00387 | -1.46399 | 0.147954 | 0.534055 | -4.86657 |
| WP_MFAP5MEDIATED_OVARIAN_CANCER_CELL_MOTILITY_AND_INVASIVENESS | -0.12655 | -0.01899 | -1.46371 | 0.148031 | 0.534055 | -4.86695 |
| REACTOME_INTERACTIONS_OF_REV_WITH_HOST_CELLULAR_PROTEINS | -0.12617 | -0.00143 | -1.46317 | 0.148177 | 0.534278 | -4.86765 |
| WEBER_METHYLATED_HCP_IN_SPERM_DN | 0.127328 | 0.017045 | 1.462241 | 0.148431 | 0.53489 | -4.86888 |
| GAUSSMANN_MLL_AF4_FUSION_TARGETS_F_DN | 0.056215 | -0.05312 | 1.461299 | 0.148689 | 0.535514 | -4.87012 |
| REACTOME_SYNTHESIS_OF_PIPS_AT_THE_ER_MEMBRANE | -0.17313 | 0.004791 | -1.46061 | 0.148877 | 0.535889 | -4.87102 |
| WP_AMPACTIVATED_PROTEIN_KINASE_AMPK_SIGNALING | -0.05585 | -0.05363 | -1.46017 | 0.148998 | 0.535904 | -4.8716 |
| DONATO_CELL_CYCLE_TRETINOIN | 0.126959 | 0.009635 | 1.459858 | 0.149083 | 0.535904 | -4.87201 |
| HEIDENBLAD_AMPLICON_12P11_12_DN | -0.07866 | -0.03593 | -1.45967 | 0.149134 | 0.535904 | -4.87225 |
| WEI_MIR34A_TARGETS | -0.08633 | -0.02418 | -1.45891 | 0.149343 | 0.536073 | -4.87326 |
| KANG_DOXORUBICIN_RESISTANCE_DN | -0.10761 | -0.1073 | -1.45889 | 0.149349 | 0.536073 | -4.87329 |
| CHEN_LIVER_METABOLISM_QTL_CIS | -0.07919 | -0.03579 | -1.45794 | 0.149609 | 0.536704 | -4.87453 |
| MCCABE_HOXC6_TARGETS_UP | 0.102455 | 0.019693 | 1.457401 | 0.149758 | 0.536935 | -4.87524 |
| WP_BIOGENIC_AMINE_SYNTHESIS | 0.109518 | 0.018278 | 1.456442 | 0.150022 | 0.537419 | -4.8765 |
| WP_DEVELOPMENT_AND_HETEROGENEITY_OF_THE_ILC_FAMILY | 0.060401 | -0.17932 | 1.456297 | 0.150062 | 0.537419 | -4.87669 |
| KEGG_PROPANOATE_METABOLISM | -0.12398 | -0.06497 | -1.45591 | 0.150168 | 0.537495 | -4.87719 |
| TAKEDA_TARGETS_OF_NUP98_HOXA9_FUSION_8D_UP | 0.059788 | -0.05816 | 1.45462 | 0.150524 | 0.538469 | -4.87889 |
| LI_LUNG_CANCER | 0.074411 | -0.17207 | 1.453596 | 0.150807 | 0.53878 | -4.88023 |
| REACTOME_EPHA_MEDIATED_GROWTH_CONE_COLLAPSE | 0.063827 | -0.06057 | 1.453589 | 0.150809 | 0.53878 | -4.88024 |
| REACTOME_TRANSPORT_OF_MATURE_MRNAS_DERIVED_FROM_INTRONLESS_TRANSCRIPTS | -0.11783 | -0.02874 | -1.4533 | 0.150889 | 0.53878 | -4.88062 |
| WP_NEURAL_CREST_CELL_MIGRATION_IN_CANCER | 0.065243 | -0.04162 | 1.452974 | 0.150979 | 0.53878 | -4.88105 |
| SA_REG_CASCADE_OF_CYCLIN_EXPR | 0.067237 | -0.18122 | 1.452521 | 0.151105 | 0.53878 | -4.88164 |
| REACTOME_ACTIVATION_OF_NIMA_KINASES_NEK9_NEK6_NEK7 | -0.13645 | -0.01458 | -1.45247 | 0.151119 | 0.53878 | -4.88171 |
| REACTOME_SYNTHESIS_OF_LEUKOTRIENES_LT_AND_EOXINS_EX | 0.07589 | -0.08168 | 1.451954 | 0.151262 | 0.538829 | -4.88238 |
| PID_HEDGEHOG_GLI_PATHWAY | -0.08565 | -0.01352 | -1.45181 | 0.151302 | 0.538829 | -4.88257 |
| REACTOME_DNA_REPLICATION_PRE_INITIATION | -0.09159 | -0.1791 | -1.45123 | 0.151464 | 0.539102 | -4.88333 |
| ONGUSAHA_TP53_TARGETS | 0.059163 | -0.25802 | 1.450403 | 0.151692 | 0.539614 | -4.88441 |
| MANNE_COVID19_ICU_VS_HEALTHY_DONOR_PLATELETS_UP | 0.061352 | -0.00105 | 1.449497 | 0.151944 | 0.540034 | -4.88559 |
| REACTOME_CA2_PATHWAY | -0.05676 | -0.03421 | -1.44937 | 0.15198 | 0.540034 | -4.88576 |
| REACTOME_PROCESSING_OF_DNA_DOUBLE_STRAND_BREAK_ENDS | -0.06984 | -0.14636 | -1.44872 | 0.15216 | 0.54037 | -4.8866 |
| WP_NEPHROTIC_SYNDROME | -0.05928 | -0.02224 | -1.44781 | 0.152415 | 0.540432 | -4.8878 |
| FEVR_CTNNB1_TARGETS_DN | -0.07753 | -0.10691 | -1.44767 | 0.152454 | 0.540432 | -4.88798 |
| REACTOME_NEUROTOXICITY_OF_CLOSTRIDIUM_TOXINS | -0.09985 | 0.007647 | -1.44733 | 0.152549 | 0.540432 | -4.88842 |
| STANELLE_E2F1_TARGETS | 0.070558 | -0.05488 | 1.447118 | 0.152607 | 0.540432 | -4.8887 |
| CROONQUIST_NRAS_SIGNALING_DN | -0.09608 | -0.06338 | -1.44657 | 0.152759 | 0.540432 | -4.88941 |
| STAMBOLSKY_TARGETS_OF_MUTATED_TP53_DN | 0.070871 | -0.18441 | 1.44653 | 0.152772 | 0.540432 | -4.88946 |
| MEBARKI_HCC_PROGENITOR_WNT_UP_CTNNB1_DEPENDENT_BLOCKED_BY_FZD8CRD | 0.087482 | -0.03893 | 1.446304 | 0.152835 | 0.540432 | -4.88976 |
| REACTOME_ROS_AND_RNS_PRODUCTION_IN_PHAGOCYTES | 0.073872 | -0.16534 | 1.446226 | 0.152856 | 0.540432 | -4.88986 |
| DASU_IL6_SIGNALING_SCAR_UP | 0.066039 | -0.09139 | 1.445589 | 0.153034 | 0.540762 | -4.89069 |
| REACTOME_RHO_GTPASES_ACTIVATE_FORMINS | -0.07252 | -0.02903 | -1.44507 | 0.153179 | 0.540902 | -4.89136 |
| MEBARKI_HCC_PROGENITOR_WNT_DN_CTNNB1_DEPENDENT_BLOCKED_BY_FZD8CRD | 0.078597 | -0.19804 | 1.444457 | 0.153351 | 0.540902 | -4.89217 |
| REACTOME_STAT3_NUCLEAR_EVENTS_DOWNSTREAM_OF_ALK_SIGNALING | 0.158205 | -0.00473 | 1.444041 | 0.153468 | 0.540902 | -4.89271 |
| QUINTENS_EMBRYONIC_BRAIN_RESPONSE_TO_IR | 0.042033 | -0.10146 | 1.443807 | 0.153534 | 0.540902 | -4.89301 |
| LUI_THYROID_CANCER_CLUSTER_2 | 0.069223 | -0.03056 | 1.443724 | 0.153557 | 0.540902 | -4.89312 |
| PID_IL8_CXCR1_PATHWAY | 0.097975 | -0.0055 | 1.443628 | 0.153584 | 0.540902 | -4.89325 |
| DAIRKEE_CANCER_PRONE_RESPONSE_BPA_E2 | -0.06677 | -0.1018 | -1.44263 | 0.153863 | 0.541132 | -4.89454 |
| WP_FOXP3_IN_COVID19 | 0.103276 | -0.06766 | 1.442404 | 0.153928 | 0.541132 | -4.89484 |
| BIOCARTA_TH1TH2_PATHWAY | 0.052777 | -0.42065 | 1.442373 | 0.153936 | 0.541132 | -4.89488 |
| WP_CEREBRAL_ORGANIC_ACIDURIAS_INCLUDING_DISEASES | -0.14161 | -0.00303 | -1.44217 | 0.153992 | 0.541132 | -4.89514 |
| KEGG_DRUG_METABOLISM_OTHER_ENZYMES | 0.080883 | -0.0715 | 1.441763 | 0.154108 | 0.541132 | -4.89567 |
| HOLLERN_EMT_BREAST_TUMOR_DN | 0.061843 | -0.12871 | 1.441551 | 0.154167 | 0.541132 | -4.89595 |
| BIOCARTA_FLUMAZENIL_PATHWAY | 0.102392 | -0.0142 | 1.441158 | 0.154278 | 0.541132 | -4.89646 |
| REACTOME_TFAP2_AP_2_FAMILY_REGULATES_TRANSCRIPTION_OF_GROWTH_FACTORS_AND_THEIR_RECEPTORS | 0.072269 | 0.017838 | 1.440975 | 0.15433 | 0.541132 | -4.8967 |
| WP_TRANSLATION_FACTORS | -0.11267 | -0.07878 | -1.44011 | 0.154573 | 0.541461 | -4.89782 |
| JOHANSSON_BRAIN_CANCER_EARLY_VS_LATE_UP | 0.104319 | -0.00932 | 1.440038 | 0.154594 | 0.541461 | -4.89791 |
| FINETTI_BREAST_CANCER_KINOME_RED | -0.14919 | -0.03497 | -1.43926 | 0.154814 | 0.541889 | -4.89892 |
| HOLLMANN_APOPTOSIS_VIA_CD40_DN | -0.07268 | -0.23243 | -1.439 | 0.154886 | 0.541889 | -4.89926 |
| MEISSNER_NPC_HCP_WITH_H3K4ME3_AND_H3K27ME3 | 0.072438 | -0.03356 | 1.43869 | 0.154974 | 0.541899 | -4.89966 |
| WP_CANONICAL_AND_NONCANONICAL_TGFB_SIGNALING | -0.09977 | -0.10982 | -1.43701 | 0.15545 | 0.542808 | -4.90184 |
| PID_TXA2PATHWAY | 0.064923 | -0.03675 | 1.436906 | 0.155479 | 0.542808 | -4.90197 |
| KORKOLA_EMBRYONAL_CARCINOMA_DN | 0.139491 | 0.03572 | 1.436866 | 0.15549 | 0.542808 | -4.90203 |
| WP_FATTY_ACID_BIOSYNTHESIS | -0.12156 | -0.09287 | -1.43612 | 0.155701 | 0.543152 | -4.90299 |
| SEAVEY_EPITHELIOID_HEMANGIOENDOTHELIOMA | 0.060621 | -0.00343 | 1.435507 | 0.155875 | 0.543152 | -4.90379 |
| MIKKELSEN_ES_ICP_WITH_H3K4ME3_AND_H3K27ME3 | 0.058593 | -0.10151 | 1.435171 | 0.155971 | 0.543152 | -4.90422 |
| RAMALHO_STEMNESS_UP | -0.09465 | -0.08775 | -1.43502 | 0.156012 | 0.543152 | -4.90441 |
| BENPORATH_NANOG_TARGETS | -0.07247 | -0.12236 | -1.43501 | 0.156015 | 0.543152 | -4.90442 |
| NIKOLSKY_BREAST_CANCER_8P12_P11_AMPLICON | -0.04711 | -0.07372 | -1.43367 | 0.156399 | 0.544189 | -4.90617 |
| WONG_EMBRYONIC_STEM_CELL_CORE | -0.09533 | -0.09337 | -1.43262 | 0.156697 | 0.544838 | -4.90752 |
| KEGG_STARCH_AND_SUCROSE_METABOLISM | 0.055692 | -0.11181 | 1.432409 | 0.156756 | 0.544838 | -4.90779 |
| HAHTOLA_MYCOSIS_FUNGOIDES_DN | 0.093852 | -0.33708 | 1.431672 | 0.156967 | 0.545161 | -4.90875 |
| NABA_CORE_MATRISOME | 0.066399 | -0.08437 | 1.431483 | 0.157021 | 0.545161 | -4.90899 |
| UZONYI_RESPONSE_TO_LEUKOTRIENE_AND_THROMBIN | 0.147673 | -0.01157 | 1.430828 | 0.157208 | 0.545512 | -4.90983 |
| REACTOME_SENSORY_PERCEPTION_OF_TASTE | 0.063579 | -0.27961 | 1.430286 | 0.157362 | 0.545752 | -4.91053 |
| WANG_METASTASIS_OF_BREAST_CANCER_ESR1_UP | -0.10771 | -0.01723 | -1.42955 | 0.157574 | 0.546033 | -4.91149 |
| REACTOME_INTEGRIN_CELL_SURFACE_INTERACTIONS | 0.071396 | -0.00359 | 1.429403 | 0.157615 | 0.546033 | -4.91167 |
| REACTOME_PROCESSIVE_SYNTHESIS_ON_THE_C_STRAND_OF_THE_TELOMERE | -0.12146 | -0.04663 | -1.42878 | 0.157794 | 0.546354 | -4.91248 |
| REACTOME_NA_CL_DEPENDENT_NEUROTRANSMITTER_TRANSPORTERS | 0.122418 | -0.02545 | 1.428314 | 0.157927 | 0.546449 | -4.91308 |
| LEE_BMP2_TARGETS_DN | -0.08268 | -0.06872 | -1.42801 | 0.158014 | 0.546449 | -4.91347 |
| KEGG_AMINOACYL_TRNA_BIOSYNTHESIS | -0.07925 | -0.2368 | -1.42779 | 0.158079 | 0.546449 | -4.91376 |
| REACTOME_PROCESSIVE_SYNTHESIS_ON_THE_LAGGING_STRAND | -0.13223 | 0.00803 | -1.42746 | 0.158173 | 0.546479 | -4.91418 |
| NIKOLSKY_BREAST_CANCER_8Q23_Q24_AMPLICON | -0.03472 | -0.15894 | -1.4265 | 0.158449 | 0.547133 | -4.91542 |
| REACTOME_MUCOPOLYSACCHARIDOSES | 0.110487 | -0.01561 | 1.425103 | 0.15885 | 0.547456 | -4.91721 |
| KEGG_OXIDATIVE_PHOSPHORYLATION | -0.08551 | -0.18219 | -1.42499 | 0.158881 | 0.547456 | -4.91735 |
| KEGG_INSULIN_SIGNALING_PATHWAY | -0.0542 | -0.11299 | -1.42463 | 0.158987 | 0.547456 | -4.91782 |
| REACTOME_THE_ROLE_OF_NEF_IN_HIV_1_REPLICATION_AND_DISEASE_PATHOGENESIS | 0.105948 | -0.06298 | 1.424127 | 0.159131 | 0.547456 | -4.91846 |
| HAHTOLA_MYCOSIS_FUNGOIDES_SKIN_UP | -0.10651 | -0.06915 | -1.42356 | 0.159295 | 0.547456 | -4.91919 |
| SMIRNOV_RESPONSE_TO_IR_2HR_DN | -0.07627 | -0.15045 | -1.42308 | 0.159433 | 0.547456 | -4.91981 |
| REACTOME_METABOLISM_OF_INGESTED_SEMET_SEC_MESEC_INTO_H2SE | -0.10099 | -0.0003 | -1.42308 | 0.159434 | 0.547456 | -4.91981 |
| REACTOME_TRANSPORT_OF_SMALL_MOLECULES | 0.027909 | -0.10185 | 1.42276 | 0.159525 | 0.547456 | -4.92022 |
| WP_NEURAL_CREST_CELL_MIGRATION_DURING_DEVELOPMENT | 0.061102 | -0.04329 | 1.422388 | 0.159633 | 0.547456 | -4.9207 |
| JIANG_TIP30_TARGETS_UP | -0.10474 | -0.13864 | -1.42142 | 0.159912 | 0.547456 | -4.92193 |
| REACTOME_BETA_OXIDATION_OF_DECANOYL_COA_TO_OCTANOYL_COA_COA | -0.16124 | 0.000314 | -1.42133 | 0.159938 | 0.547456 | -4.92205 |
| COLIN_PILOCYTIC_ASTROCYTOMA_VS_GLIOBLASTOMA_UP | 0.07205 | -0.19715 | 1.421295 | 0.159949 | 0.547456 | -4.9221 |
| REACTOME_TRNA_AMINOACYLATION | -0.10925 | -0.08488 | -1.42127 | 0.159956 | 0.547456 | -4.92213 |
| REICHERT_MITOSIS_LIN9_TARGETS | -0.12167 | -0.02698 | -1.42084 | 0.160082 | 0.547456 | -4.92269 |
| WP_CANCER_IMMUNOTHERAPY_BY_PD1_BLOCKADE | 0.064785 | -0.35759 | 1.420514 | 0.160175 | 0.547456 | -4.9231 |
| DACOSTA_UV_RESPONSE_VIA_ERCC3_DN | -0.09767 | -0.05084 | -1.41997 | 0.160334 | 0.547456 | -4.9238 |
| ABE_VEGFA_TARGETS | 0.103686 | -0.00484 | 1.41981 | 0.16038 | 0.547456 | -4.924 |
| MEISSNER_NPC_HCP_WITH_H3K4ME2 | 0.047488 | -0.04724 | 1.419589 | 0.160444 | 0.547456 | -4.92429 |
| REACTOME_HDMS_DEMETHYLATE_HISTONES | 0.104879 | -0.00124 | 1.419569 | 0.16045 | 0.547456 | -4.92431 |
| BOYLAN_MULTIPLE_MYELOMA_D_CLUSTER_UP | -0.07691 | -0.00461 | -1.41947 | 0.160479 | 0.547456 | -4.92444 |
| KANG_IMMORTALIZED_BY_TERT_UP | 0.056242 | -0.03558 | 1.418936 | 0.160633 | 0.547456 | -4.92512 |
| NABA_PROTEOGLYCANS | 0.076374 | -0.02556 | 1.418731 | 0.160693 | 0.547456 | -4.92539 |
| BIOCARTA_BTG2_PATHWAY | 0.076709 | -0.00857 | 1.41849 | 0.160763 | 0.547456 | -4.92569 |
| VANHARANTA_UTERINE_FIBROID_WITH_7Q_DELETION_DN | -0.10784 | -0.13192 | -1.41846 | 0.16077 | 0.547456 | -4.92573 |
| KEGG_NEUROACTIVE_LIGAND_RECEPTOR_INTERACTION | 0.089083 | -0.05436 | 1.418457 | 0.160772 | 0.547456 | -4.92574 |
| VARELA_ZMPSTE24_TARGETS_UP | 0.088408 | -0.04493 | 1.418436 | 0.160778 | 0.547456 | -4.92576 |
| REACTOME_COLLAGEN_CHAIN_TRIMERIZATION | 0.094383 | -0.08282 | 1.417577 | 0.161028 | 0.547946 | -4.92686 |
| KEGG_BUTANOATE_METABOLISM | -0.08673 | -0.0352 | -1.41735 | 0.161095 | 0.547946 | -4.92715 |
| LIU_SOX4_TARGETS_DN | -0.0697 | -0.12081 | -1.41687 | 0.161235 | 0.547952 | -4.92777 |
| BLALOCK_ALZHEIMERS_DISEASE_DN | -0.08803 | -0.09174 | -1.41675 | 0.161269 | 0.547952 | -4.92792 |
| REACTOME_NGF_STIMULATED_TRANSCRIPTION | 0.085759 | -0.02759 | 1.416199 | 0.16143 | 0.548206 | -4.92862 |
| REACTOME_THE_RETINOID_CYCLE_IN_CONES_DAYLIGHT_VISION | 0.121613 | 0.02202 | 1.415723 | 0.161569 | 0.548271 | -4.92923 |
| PID_BMP_PATHWAY | -0.07022 | -0.06687 | -1.41507 | 0.161759 | 0.548271 | -4.93007 |
| APPEL_IMATINIB_RESPONSE | 0.095558 | -0.24432 | 1.414934 | 0.161799 | 0.548271 | -4.93024 |
| BIOCARTA_SLRP_PATHWAY | 0.148711 | -0.02419 | 1.414737 | 0.161857 | 0.548271 | -4.93049 |
| FIGUEROA_AML_METHYLATION_CLUSTER_7_DN | 0.148357 | 0.035569 | 1.414348 | 0.16197 | 0.548271 | -4.93099 |
| CAIRO_HEPATOBLASTOMA_DN | 0.036339 | -0.06811 | 1.41378 | 0.162136 | 0.548271 | -4.93171 |
| MATTHEWS_AP1_TARGETS | 0.111639 | 0.007481 | 1.412931 | 0.162385 | 0.548271 | -4.9328 |
| BOYLAN_MULTIPLE_MYELOMA_PCA3_DN | -0.06528 | -0.04315 | -1.41289 | 0.162398 | 0.548271 | -4.93285 |
| REACTOME_ADENYLATE_CYCLASE_ACTIVATING_PATHWAY | -0.09443 | -0.08881 | -1.4128 | 0.162422 | 0.548271 | -4.93296 |
| REACTOME_BRANCHED_CHAIN_AMINO_ACID_CATABOLISM | -0.12501 | -0.01012 | -1.41279 | 0.162425 | 0.548271 | -4.93297 |
| WP_MIRNA_TARGETS_IN_ECM_AND_MEMBRANE_RECEPTORS | 0.11824 | -0.23802 | 1.412277 | 0.162577 | 0.548271 | -4.93363 |
| REACTOME_SIGNALING_BY_NTRK2_TRKB | -0.06119 | -0.03248 | -1.41164 | 0.162762 | 0.548271 | -4.93444 |
| MORI_IMMATURE_B_LYMPHOCYTE_UP | 0.066809 | -0.311 | 1.411599 | 0.162775 | 0.548271 | -4.9345 |
| BIOCARTA_INSULIN_PATHWAY | -0.10734 | -0.07492 | -1.41159 | 0.162778 | 0.548271 | -4.93451 |
| ACEVEDO_METHYLATED_IN_LIVER_CANCER_DN | 0.043107 | -0.13183 | 1.411476 | 0.162812 | 0.548271 | -4.93465 |
| PID_MYC_ACTIV_PATHWAY | 0.063844 | -0.2872 | 1.411414 | 0.16283 | 0.548271 | -4.93473 |
| WP_TRANSSULFURATION_PATHWAY | -0.14153 | -0.0033 | -1.41048 | 0.163103 | 0.548271 | -4.93592 |
| VERHAAK_GLIOBLASTOMA_NEURAL | -0.06097 | -0.03299 | -1.41043 | 0.16312 | 0.548271 | -4.93599 |
| WANG_MLL_TARGETS | 0.049986 | -0.0841 | 1.410151 | 0.163201 | 0.548271 | -4.93634 |
| REACTOME_NS1_MEDIATED_EFFECTS_ON_HOST_PATHWAYS | -0.11362 | -0.02132 | -1.40996 | 0.163258 | 0.548271 | -4.93659 |
| BIOCARTA_CFTR_PATHWAY | -0.07892 | -0.07713 | -1.40978 | 0.163309 | 0.548271 | -4.93681 |
| REACTOME_HYDROLYSIS_OF_LPC | 0.129754 | -0.01502 | 1.409589 | 0.163366 | 0.548271 | -4.93706 |
| GARGALOVIC_RESPONSE_TO_OXIDIZED_PHOSPHOLIPIDS_RED_DN | -0.10864 | -0.07447 | -1.40904 | 0.163526 | 0.548271 | -4.93775 |
| BIOCARTA_STATHMIN_PATHWAY | -0.10201 | -0.06104 | -1.40856 | 0.16367 | 0.548271 | -4.93837 |
| FLORIO_NEOCORTEX_BASAL_RADIAL_GLIA_UP | 0.066425 | 0.013778 | 1.408517 | 0.163682 | 0.548271 | -4.93842 |
| HANN_RESISTANCE_TO_BCL2_INHIBITOR_DN | 0.048428 | -0.13749 | 1.408494 | 0.163689 | 0.548271 | -4.93845 |
| MURAKAMI_UV_RESPONSE_1HR_DN | 0.094552 | -0.01454 | 1.407711 | 0.163919 | 0.548298 | -4.93944 |
| WP_MIRNA_REGULATION_OF_PROSTATE_CANCER_SIGNALING_PATHWAYS | 0.07429 | -0.05965 | 1.407608 | 0.16395 | 0.548298 | -4.93958 |
| KEGG_PYRIMIDINE_METABOLISM | -0.07968 | -0.03848 | -1.40759 | 0.163955 | 0.548298 | -4.9396 |
| WP_ARYL_HYDROCARBON_RECEPTOR_PATHWAY_WP2873 | 0.05331 | -0.12837 | 1.406582 | 0.164253 | 0.549005 | -4.94088 |
| STEIN_ESRRA_TARGETS_DN | -0.0903 | -0.15109 | -1.40583 | 0.164474 | 0.549328 | -4.94183 |
| ONKEN_UVEAL_MELANOMA_DN | -0.09103 | -0.08654 | -1.40548 | 0.164578 | 0.549328 | -4.94227 |
| REACTOME_NR1H2_NR1H3_REGULATE_GENE_EXPRESSION_LINKED_TO_GLUCONEOGENESIS | 0.093814 | -0.46027 | 1.404961 | 0.164732 | 0.549328 | -4.94294 |
| SHIN_B_CELL_LYMPHOMA_CLUSTER_7 | -0.04831 | -0.23479 | -1.40482 | 0.164774 | 0.549328 | -4.94311 |
| WP_SEROTONIN_RECEPTOR_467_AND_NR3C_SIGNALING | -0.10075 | 0.005702 | -1.4048 | 0.164781 | 0.549328 | -4.94315 |
| SU_SALIVARY_GLAND | 0.124746 | 0.037538 | 1.404294 | 0.16493 | 0.549537 | -4.94378 |
| SERVITJA_ISLET_HNF1A_TARGETS_UP | 0.063607 | -0.05661 | 1.403749 | 0.165092 | 0.549545 | -4.94447 |
| REACTOME_TRANSLESION_SYNTHESIS_BY_Y_FAMILY_DNA_POLYMERASES_BYPASSES_LESIONS_ON_DNA_TEMPLATE | -0.09728 | 0.00127 | -1.4037 | 0.165105 | 0.549545 | -4.94453 |
| STEGER_ADIPOGENESIS_UP | 0.116881 | -0.02783 | 1.402872 | 0.165352 | 0.549719 | -4.94559 |
| REACTOME_PROPIONYL_COA_CATABOLISM | -0.1744 | -0.02432 | -1.40269 | 0.165407 | 0.549719 | -4.94582 |
| FLECHNER_BIOPSY_KIDNEY_TRANSPLANT_REJECTED_VS_OK_DN | -0.08076 | -0.06051 | -1.40265 | 0.165417 | 0.549719 | -4.94586 |
| REACTOME_LGI_ADAM_INTERACTIONS | 0.112323 | 0.024665 | 1.401328 | 0.165811 | 0.550742 | -4.94754 |
| ELVIDGE_HIF1A_AND_HIF2A_TARGETS_DN | 0.086446 | -0.06551 | 1.400554 | 0.166042 | 0.55089 | -4.94852 |
| IKEDA_MIR1_TARGETS_DN | -0.12037 | -0.00805 | -1.40055 | 0.166044 | 0.55089 | -4.94853 |
| REACTOME_ACETYLCHOLINE_INHIBITS_CONTRACTION_OF_OUTER_HAIR_CELLS | 0.125633 | 0.006834 | 1.399902 | 0.166236 | 0.55089 | -4.94935 |
| ZHU_CMV_8_HR_UP | 0.060206 | -0.26299 | 1.399835 | 0.166256 | 0.55089 | -4.94943 |
| OUYANG_PROSTATE_CANCER_PROGRESSION_UP | 0.103589 | -0.10029 | 1.399566 | 0.166336 | 0.55089 | -4.94977 |
| RUNNE_GENDER_EFFECT_UP | -0.14181 | -0.01545 | -1.39944 | 0.166375 | 0.55089 | -4.94993 |
| WP_OXYSTEROLS_DERIVED_FROM_CHOLESTEROL | 0.056319 | -0.02193 | 1.398212 | 0.16674 | 0.551242 | -4.95148 |
| REACTOME_SIGNAL_REGULATORY_PROTEIN_FAMILY_INTERACTIONS | 0.100593 | -0.00546 | 1.397408 | 0.166981 | 0.551242 | -4.9525 |
| HELLER_SILENCED_BY_METHYLATION_UP | 0.039251 | -0.29719 | 1.397338 | 0.167002 | 0.551242 | -4.95259 |
| REACTOME_CELL_JUNCTION_ORGANIZATION | 0.058476 | -0.01303 | 1.397286 | 0.167018 | 0.551242 | -4.95265 |
| REACTOME_REGULATION_OF_PTEN_GENE_TRANSCRIPTION | -0.07507 | -0.09532 | -1.39689 | 0.167137 | 0.551242 | -4.95315 |
| SAKAI_TUMOR_INFILTRATING_MONOCYTES_DN | -0.11765 | -0.03929 | -1.39675 | 0.167178 | 0.551242 | -4.95333 |
| REACTOME_DNA_DAMAGE_BYPASS | -0.09564 | -0.0028 | -1.39657 | 0.167231 | 0.551242 | -4.95355 |
| REACTOME_ACYL_CHAIN_REMODELING_OF_DAG_AND_TAG | 0.099051 | -0.11726 | 1.396554 | 0.167237 | 0.551242 | -4.95357 |
| BENNETT_SYSTEMIC_LUPUS_ERYTHEMATOSUS | 0.076567 | -0.2753 | 1.396436 | 0.167272 | 0.551242 | -4.95372 |
| CAIRO_HEPATOBLASTOMA_UP | -0.08531 | -0.12797 | -1.39618 | 0.167348 | 0.551242 | -4.95404 |
| ZWANG_CLASS_1_TRANSIENTLY_INDUCED_BY_EGF | 0.056533 | -0.09978 | 1.395524 | 0.167546 | 0.551609 | -4.95487 |
| BIOCARTA_IGF1R_PATHWAY | -0.08269 | -0.10124 | -1.39521 | 0.167638 | 0.551629 | -4.95526 |
| LAMB_CCND1_TARGETS | 0.054022 | -0.32449 | 1.394462 | 0.167865 | 0.552039 | -4.95621 |
| BIOCARTA_SHH_PATHWAY | -0.08676 | -0.10267 | -1.39422 | 0.167936 | 0.552039 | -4.95651 |
| CREIGHTON_ENDOCRINE_THERAPY_RESISTANCE_4 | -0.05695 | -0.0879 | -1.39372 | 0.168088 | 0.55208 | -4.95715 |
| SARTIPY_NORMAL_AT_INSULIN_RESISTANCE_UP | 0.122732 | -0.01373 | 1.393512 | 0.16815 | 0.55208 | -4.95741 |
| REACTOME_SEMA3A_PLEXIN_REPULSION_SIGNALING_BY_INHIBITING_INTEGRIN_ADHESION | 0.109171 | -0.00336 | 1.393316 | 0.168209 | 0.55208 | -4.95766 |
| REACTOME_TRANSLESION_SYNTHESIS_BY_POLK | -0.12776 | -0.00447 | -1.39288 | 0.16834 | 0.552224 | -4.9582 |
| ROSS_LEUKEMIA_WITH_MLL_FUSIONS | -0.07593 | -0.09597 | -1.39249 | 0.168459 | 0.552252 | -4.9587 |
| PID_IL4_2PATHWAY | 0.07221 | -0.08717 | 1.39217 | 0.168555 | 0.552252 | -4.9591 |
| ALONSO_METASTASIS_UP | -0.10906 | -0.06043 | -1.39144 | 0.168775 | 0.552252 | -4.96002 |
| MIKKELSEN_NPC_LCP_WITH_H3K4ME3 | 0.047609 | -0.22023 | 1.391286 | 0.168821 | 0.552252 | -4.96021 |
| WANG_ADIPOGENIC_GENES_REPRESSED_BY_SIRT1 | 0.093564 | -0.10199 | 1.391234 | 0.168837 | 0.552252 | -4.96027 |
| PID_ATF2_PATHWAY | 0.077962 | -0.04424 | 1.391127 | 0.168869 | 0.552252 | -4.96041 |
| REACTOME_SYNTHESIS_OF_IP3_AND_IP4_IN_THE_CYTOSOL | -0.06334 | -0.10937 | -1.39083 | 0.168959 | 0.552263 | -4.96078 |
| REACTOME_SYNTHESIS_OF_PIPS_AT_THE_PLASMA_MEMBRANE | -0.06984 | -0.07536 | -1.39002 | 0.169202 | 0.552476 | -4.96179 |
| REACTOME_PROCESSING_OF_SMDT1 | -0.13014 | -0.12869 | -1.38992 | 0.169233 | 0.552476 | -4.96192 |
| WAESCH_ANAPHASE_PROMOTING_COMPLEX | -0.12661 | -0.0152 | -1.38956 | 0.169341 | 0.552476 | -4.96237 |
| PID_NEPHRIN_NEPH1_PATHWAY | -0.09097 | -0.02167 | -1.38938 | 0.169398 | 0.552476 | -4.96261 |
| LI_DCP2_BOUND_MRNA | -0.10763 | -0.07083 | -1.38917 | 0.169461 | 0.552476 | -4.96287 |
| DAVIES_MULTIPLE_MYELOMA_VS_MGUS_UP | 0.069582 | -0.31081 | 1.388716 | 0.169598 | 0.552476 | -4.96344 |
| CASORELLI_ACUTE_PROMYELOCYTIC_LEUKEMIA_DN | -0.08174 | -0.09386 | -1.38856 | 0.169644 | 0.552476 | -4.96363 |
| TSUNODA_CISPLATIN_RESISTANCE_UP | 0.110984 | -0.06888 | 1.387997 | 0.169816 | 0.552476 | -4.96434 |
| GROSS_HYPOXIA_VIA_ELK3_DN | 0.086842 | -0.01247 | 1.387667 | 0.169916 | 0.552476 | -4.96475 |
| PID_ERBB_NETWORK_PATHWAY | 0.104885 | 0.010623 | 1.387625 | 0.169929 | 0.552476 | -4.96481 |
| WP_VITAMIN_DSENSITIVE_CALCIUM_SIGNALING_IN_DEPRESSION | 0.044859 | -0.08929 | 1.38695 | 0.170134 | 0.552476 | -4.96565 |
| PID_ERBB1_RECEPTOR_PROXIMAL_PATHWAY | -0.09742 | -0.02431 | -1.38691 | 0.170145 | 0.552476 | -4.9657 |
| WP_GLYCEROLIPIDS_AND_GLYCEROPHOSPHOLIPIDS | 0.070896 | -0.1224 | 1.386887 | 0.170153 | 0.552476 | -4.96573 |
| RICKMAN_TUMOR_DIFFERENTIATED_WELL_VS_POORLY_DN | 0.055835 | -0.0581 | 1.385757 | 0.170496 | 0.553093 | -4.96715 |
| WP_FTO_OBESITY_VARIANT_MECHANISM | 0.114707 | -0.00048 | 1.385427 | 0.170596 | 0.553093 | -4.96756 |
| REACTOME_TERMINATION_OF_O_GLYCAN_BIOSYNTHESIS | 0.082298 | -0.08723 | 1.384541 | 0.170866 | 0.553093 | -4.96867 |
| VERNELL_RETINOBLASTOMA_PATHWAY_DN | 0.116485 | -0.0063 | 1.384384 | 0.170914 | 0.553093 | -4.96887 |
| WP_CORI_CYCLE | 0.079822 | -0.16715 | 1.384372 | 0.170918 | 0.553093 | -4.96888 |
| SASAI_TARGETS_OF_CXCR6_AND_PTCH1_DN | 0.111352 | -0.00631 | 1.384371 | 0.170918 | 0.553093 | -4.96888 |
| LOPEZ_MBD_TARGETS_IMPRINTED_AND_X_LINKED | -0.09671 | 9.36E-05 | -1.38399 | 0.171033 | 0.553093 | -4.96935 |
| REACTOME_VEGFR2_MEDIATED_CELL_PROLIFERATION | -0.09977 | -0.04228 | -1.38398 | 0.171038 | 0.553093 | -4.96938 |
| KEGG_LEUKOCYTE_TRANSENDOTHELIAL_MIGRATION | 0.048639 | -0.05333 | 1.38316 | 0.171287 | 0.553617 | -4.9704 |
| FINETTI_BREAST_CANCERS_KINOME_BLUE | -0.06834 | 0.005367 | -1.38274 | 0.171417 | 0.553754 | -4.97093 |
| REACTOME_TRANSCRIPTIONAL_REGULATION_BY_RUNX1 | -0.06869 | -0.15872 | -1.38183 | 0.171692 | 0.554314 | -4.97205 |
| BONOME_OVARIAN_CANCER_POOR_SURVIVAL_UP | -0.11368 | -0.00463 | -1.3816 | 0.171764 | 0.554314 | -4.97235 |
| WP_TYPE_2_PAPILLARY_RENAL_CELL_CARCINOMA | 0.096417 | -0.00907 | 1.3794 | 0.172438 | 0.555641 | -4.97509 |
| CAIRO_LIVER_DEVELOPMENT_UP | -0.07605 | -0.04932 | -1.37915 | 0.172514 | 0.555641 | -4.9754 |
| REACTOME_MRNA_SPLICING_MINOR_PATHWAY | -0.10103 | -0.12553 | -1.37909 | 0.172534 | 0.555641 | -4.97548 |
| REACTOME_P75_NTR_RECEPTOR_MEDIATED_SIGNALLING | 0.054633 | -0.06967 | 1.379071 | 0.172539 | 0.555641 | -4.9755 |
| WATANABE_COLON_CANCER_MSI_VS_MSS_UP | -0.05699 | -0.07122 | -1.37883 | 0.172612 | 0.555641 | -4.9758 |
| RIZ_ERYTHROID_DIFFERENTIATION_CCNE1 | -0.06328 | -0.1548 | -1.37851 | 0.172711 | 0.555679 | -4.9762 |
| REACTOME_PLATELET_HOMEOSTASIS | -0.04314 | -0.00735 | -1.37813 | 0.172827 | 0.555767 | -4.97667 |
| HUMMERICH_BENIGN_SKIN_TUMOR_DN | 0.082608 | -0.13781 | 1.377645 | 0.172977 | 0.555767 | -4.97728 |
| REACTOME_METABOLISM_OF_AMINO_ACIDS_AND_DERIVATIVES | -0.0634 | -0.10648 | -1.37743 | 0.173044 | 0.555767 | -4.97755 |
| HEIDENBLAD_AMPLIFIED_IN_PANCREATIC_CANCER | -0.05399 | -0.33469 | -1.37722 | 0.173108 | 0.555767 | -4.97781 |
| CHESLER_BRAIN_HIGHEST_EXPRESSION | 0.061986 | -0.02784 | 1.376827 | 0.173228 | 0.555767 | -4.9783 |
| WP_ACETYLCHOLINE_SYNTHESIS | 0.111474 | 0.016615 | 1.376703 | 0.173266 | 0.555767 | -4.97845 |
| LIN_NPAS4_TARGETS_DN | 0.060844 | -0.01847 | 1.376257 | 0.173404 | 0.555767 | -4.97901 |
| REACTOME_REGULATION_OF_INNATE_IMMUNE_RESPONSES_TO_CYTOSOLIC_DNA | 0.11473 | 0.00117 | 1.375994 | 0.173485 | 0.555767 | -4.97934 |
| GRANDVAUX_IRF3_TARGETS_DN | 0.078473 | -0.05187 | 1.375866 | 0.173524 | 0.555767 | -4.97949 |
| NIKOLSKY_BREAST_CANCER_7Q21_Q22_AMPLICON | -0.05185 | -0.01698 | -1.37528 | 0.173705 | 0.555896 | -4.98022 |
| LANDEMAINE_LUNG_METASTASIS | 0.059278 | -0.08921 | 1.375169 | 0.173739 | 0.555896 | -4.98036 |
| WP_CANONICAL_AND_NONCANONICAL_NOTCH_SIGNALING | -0.06063 | -0.21657 | -1.37469 | 0.173887 | 0.556074 | -4.98096 |
| KEGG_N_GLYCAN_BIOSYNTHESIS | -0.10315 | -0.01938 | -1.37442 | 0.17397 | 0.556074 | -4.98129 |
| MIKKELSEN_ES_HCP_WITH_H3_UNMETHYLATED | 0.093599 | -0.04005 | 1.373986 | 0.174105 | 0.556117 | -4.98183 |
| BIOCARTA_ERYTH_PATHWAY | 0.080721 | -0.12045 | 1.372859 | 0.174453 | 0.556117 | -4.98323 |
| REACTOME_LDL_CLEARANCE | 0.097133 | 0.000503 | 1.372449 | 0.17458 | 0.556117 | -4.98374 |
| ELVIDGE_HIF1A_AND_HIF2A_TARGETS_UP | -0.0944 | 0.009045 | -1.37225 | 0.174643 | 0.556117 | -4.98399 |
| WP_OSTEOBLAST_SIGNALING | 0.09192 | -0.00814 | 1.372203 | 0.174656 | 0.556117 | -4.98405 |
| REACTOME_DEGRADATION_OF_BETA_CATENIN_BY_THE_DESTRUCTION_COMPLEX | -0.08107 | -0.17228 | -1.37175 | 0.174797 | 0.556117 | -4.98461 |
| REACTOME_NUCLEAR_IMPORT_OF_REV_PROTEIN | -0.11471 | -0.00257 | -1.37173 | 0.174804 | 0.556117 | -4.98464 |
| SONG_TARGETS_OF_IE86_CMV_PROTEIN | -0.08855 | -0.0375 | -1.3717 | 0.174812 | 0.556117 | -4.98467 |
| SEITZ_NEOPLASTIC_TRANSFORMATION_BY_8P_DELETION_DN | 0.050777 | -0.20187 | 1.371426 | 0.174897 | 0.556117 | -4.98501 |
| ZHONG_SECRETOME_OF_LUNG_CANCER_AND_ENDOTHELIUM | 0.053055 | -0.29645 | 1.370362 | 0.175227 | 0.556117 | -4.98633 |
| VANASSE_BCL2_TARGETS_UP | -0.05802 | -0.2467 | -1.37008 | 0.175314 | 0.556117 | -4.98668 |
| WP_UREA_CYCLE_AND_RELATED_DISEASES | -0.11005 | 0.008269 | -1.36978 | 0.175407 | 0.556117 | -4.98705 |
| REACTOME_RHOBTB3_ATPASE_CYCLE | -0.10171 | -0.00433 | -1.36953 | 0.175485 | 0.556117 | -4.98736 |
| REACTOME_PHOSPHORYLATION_OF_EMI1 | -0.12962 | -0.02403 | -1.36941 | 0.175523 | 0.556117 | -4.98751 |
| COLIN_PILOCYTIC_ASTROCYTOMA_VS_GLIOBLASTOMA_DN | 0.107797 | -0.03983 | 1.369317 | 0.175552 | 0.556117 | -4.98762 |
| HUPER_BREAST_BASAL_VS_LUMINAL_DN | 0.046265 | -0.36693 | 1.369308 | 0.175555 | 0.556117 | -4.98763 |
| REACTOME_RESOLUTION_OF_AP_SITES_VIA_THE_MULTIPLE_NUCLEOTIDE_PATCH_REPLACEMENT_PATHWAY | -0.13154 | -0.01178 | -1.36916 | 0.175602 | 0.556117 | -4.98782 |
| WP_CELL_MIGRATION_AND_INVASION_THROUGH_P75NTR | 0.069062 | -0.06438 | 1.369128 | 0.175611 | 0.556117 | -4.98786 |
| CROONQUIST_IL6_DEPRIVATION_UP | 0.085244 | -0.07639 | 1.369022 | 0.175644 | 0.556117 | -4.98799 |
| BYSTRYKH_HEMATOPOIESIS_STEM_CELL_AND_BRAIN_QTL_CIS | -0.10904 | -0.08702 | -1.36835 | 0.175854 | 0.556506 | -4.98882 |
| BIOCARTA_VDR_PATHWAY | -0.11676 | -0.01279 | -1.36772 | 0.176049 | 0.556693 | -4.9896 |
| HONRADO_BREAST_CANCER_BRCA1_VS_BRCA2 | 0.066908 | -0.05582 | 1.367596 | 0.176088 | 0.556693 | -4.98975 |
| MITSIADES_RESPONSE_TO_APLIDIN_DN | -0.08947 | -0.07766 | -1.36706 | 0.176253 | 0.55694 | -4.99041 |
| REACTOME_INTRAFLAGELLAR_TRANSPORT | -0.0757 | 0.000993 | -1.36605 | 0.17657 | 0.55741 | -4.99166 |
| BIOCARTA_SM_PATHWAY | -0.13167 | 0.011813 | -1.36539 | 0.176777 | 0.55741 | -4.99248 |
| REACTOME_TNF_RECEPTOR_SUPERFAMILY_TNFSF_MEMBERS_MEDIATING_NON_CANONICAL_NF_KB_PATHWAY | 0.067223 | -0.33929 | 1.36526 | 0.176817 | 0.55741 | -4.99264 |
| MARTINELLI_IMMATURE_NEUTROPHIL_UP | 0.07374 | -0.25857 | 1.365181 | 0.176842 | 0.55741 | -4.99274 |
| PANGAS_TUMOR_SUPPRESSION_BY_SMAD1_AND_SMAD5_UP | 0.056425 | -0.05302 | 1.365005 | 0.176897 | 0.55741 | -4.99296 |
| PID_INTEGRIN3_PATHWAY | 0.086903 | -0.0077 | 1.364892 | 0.176932 | 0.55741 | -4.9931 |
| CASTELLANO_HRAS_AND_NRAS_TARGETS_DN | -0.11989 | -0.0078 | -1.36459 | 0.177027 | 0.55741 | -4.99347 |
| VANTVEER_BREAST_CANCER_METASTASIS_UP | -0.07571 | -0.00751 | -1.36404 | 0.177199 | 0.55741 | -4.99415 |
| REACTOME_RAS_PROCESSING | -0.09723 | -0.04322 | -1.36345 | 0.177382 | 0.55741 | -4.99487 |
| PATIL_LIVER_CANCER | -0.05989 | -0.16854 | -1.36315 | 0.177478 | 0.55741 | -4.99525 |
| REACTOME_GOLGI_CISTERNAE_PERICENTRIOLAR_STACK_REORGANIZATION | -0.13148 | -0.01462 | -1.36296 | 0.177537 | 0.55741 | -4.99548 |
| BOYLAN_MULTIPLE_MYELOMA_C_D_DN | 0.049418 | -0.1277 | 1.362876 | 0.177564 | 0.55741 | -4.99558 |
| TURJANSKI_MAPK14_TARGETS | 0.091314 | -0.00385 | 1.362677 | 0.177626 | 0.55741 | -4.99583 |
| SHEPARD_BMYB_TARGETS | 0.06182 | -0.11199 | 1.362669 | 0.177629 | 0.55741 | -4.99584 |
| ZHAN_MULTIPLE_MYELOMA_CD2_DN | -0.06903 | -0.08628 | -1.36126 | 0.178072 | 0.558525 | -4.99758 |
| REACTOME_TRAF6_MEDIATED_IRF7_ACTIVATION_IN_TLR7_8_OR_9_SIGNALING | 0.106889 | -0.06421 | 1.36087 | 0.178194 | 0.558633 | -4.99806 |
| REACTOME_UREA_CYCLE | -0.09867 | -0.09306 | -1.36016 | 0.178416 | 0.558641 | -4.99893 |
| REACTOME_ACTIVATION_OF_ATR_IN_RESPONSE_TO_REPLICATION_STRESS | -0.07675 | -0.03956 | -1.35986 | 0.178512 | 0.558641 | -4.9993 |
| REACTOME_INTRINSIC_PATHWAY_FOR_APOPTOSIS | -0.07282 | -0.05452 | -1.35975 | 0.178545 | 0.558641 | -4.99943 |
| PELLICCIOTTA_HDAC_IN_ANTIGEN_PRESENTATION_UP | -0.10592 | -0.09265 | -1.35888 | 0.178819 | 0.558641 | -5.0005 |
| ANDERSEN_CHOLANGIOCARCINOMA_CLASS1 | 0.052144 | -0.14703 | 1.35887 | 0.178824 | 0.558641 | -5.00052 |
| WP_STEROL_REGULATORY_ELEMENTBINDING_PROTEINS_SREBP_SIGNALING | -0.08611 | -0.05322 | -1.35837 | 0.17898 | 0.558641 | -5.00113 |
| BIOCARTA_BCELLSURVIVAL_PATHWAY | -0.09541 | 0.012223 | -1.3582 | 0.179035 | 0.558641 | -5.00134 |
| SASAKI_ADULT_T_CELL_LEUKEMIA | -0.07994 | -0.10754 | -1.35814 | 0.179055 | 0.558641 | -5.00142 |
| REACTOME_TRP_CHANNELS | 0.078756 | -0.02726 | 1.358126 | 0.179058 | 0.558641 | -5.00143 |
| TIEN_INTESTINE_PROBIOTICS_2HR_DN | -0.08403 | -0.03891 | -1.35791 | 0.179127 | 0.558641 | -5.0017 |
| REACTOME_HEDGEHOG_ON_STATE | -0.06435 | -0.18666 | -1.3578 | 0.179162 | 0.558641 | -5.00183 |
| MISSIAGLIA_REGULATED_BY_METHYLATION_DN | -0.08083 | -0.13555 | -1.35709 | 0.179385 | 0.558738 | -5.0027 |
| CHOI_ATL_STAGE_PREDICTOR | -0.13173 | -0.01548 | -1.35702 | 0.179409 | 0.558738 | -5.00279 |
| REACTOME_NUCLEAR_ENVELOPE_BREAKDOWN | -0.1134 | -0.02966 | -1.35649 | 0.179574 | 0.558738 | -5.00344 |
| SANSOM_APC_MYC_TARGETS | -0.05443 | -0.0831 | -1.35597 | 0.179739 | 0.558738 | -5.00408 |
| BIOCARTA_CTBP1_PATHWAY | -0.08993 | 0.016371 | -1.35541 | 0.179916 | 0.558738 | -5.00476 |
| PUJANA_BRCA1_PCC_NETWORK | -0.0758 | -0.13995 | -1.35514 | 0.180002 | 0.558738 | -5.00509 |
| BOYLAN_MULTIPLE_MYELOMA_C_D_UP | -0.04042 | -0.1034 | -1.35514 | 0.180003 | 0.558738 | -5.0051 |
| RUAN_RESPONSE_TO_TNF_TROGLITAZONE_DN | 0.067288 | -0.05385 | 1.355089 | 0.180019 | 0.558738 | -5.00516 |
| KANG_IMMORTALIZED_BY_TERT_DN | 0.04065 | -0.07044 | 1.354855 | 0.180093 | 0.558738 | -5.00545 |
| BIOCARTA_ACETAMINOPHEN_PATHWAY | 0.108541 | -0.02159 | 1.354726 | 0.180134 | 0.558738 | -5.00561 |
| MOOTHA_GLYCOGEN_METABOLISM | -0.07542 | -0.09474 | -1.35446 | 0.180219 | 0.558738 | -5.00593 |
| KONG_E2F3_TARGETS | -0.08173 | -0.09312 | -1.35437 | 0.180247 | 0.558738 | -5.00604 |
| DACOSTA_UV_RESPONSE_VIA_ERCC3_TTD_DN | -0.07483 | -0.0523 | -1.35393 | 0.180385 | 0.558896 | -5.00658 |
| BRUECKNER_TARGETS_OF_MIRLET7A3_DN | 0.083155 | -0.01842 | 1.353359 | 0.180568 | 0.559092 | -5.00728 |
| FIGUEROA_AML_METHYLATION_CLUSTER_2_UP | -0.05329 | -0.10194 | -1.35318 | 0.180624 | 0.559092 | -5.0075 |
| BYSTROEM_CORRELATED_WITH_IL5_UP | 0.072657 | -0.12578 | 1.352706 | 0.180776 | 0.559288 | -5.00808 |
| BIOCARTA_FBW7_PATHWAY | -0.10638 | -0.01162 | -1.35223 | 0.180927 | 0.559427 | -5.00866 |
| BONOME_OVARIAN_CANCER_SURVIVAL_OPTIMAL_DEBULKING | -0.05157 | -0.15466 | -1.35201 | 0.180996 | 0.559427 | -5.00893 |
| HOLLERN_EMT_BREAST_TUMOR_UP | 0.071871 | -0.03855 | 1.351535 | 0.181149 | 0.559502 | -5.00951 |
| VECCHI_GASTRIC_CANCER_ADVANCED_VS_EARLY_DN | 0.036376 | -0.10994 | 1.350871 | 0.18136 | 0.559502 | -5.01033 |
| IVANOVA_HEMATOPOIESIS_STEM_CELL_SHORT_TERM | -0.06747 | -0.08329 | -1.35017 | 0.181584 | 0.559502 | -5.01118 |
| CHOW_RASSF1_TARGETS_DN | -0.09201 | -0.08389 | -1.3499 | 0.181671 | 0.559502 | -5.01152 |
| ZWANG_CLASS_2_TRANSIENTLY_INDUCED_BY_EGF | 0.057963 | -0.47363 | 1.349603 | 0.181764 | 0.559502 | -5.01188 |
| WP_MICRORNA_FOR_TARGETING_CANCER_GROWTH_AND_VASCULARIZATION_IN_GLIOBLASTOMA | 0.144761 | 0.000842 | 1.349517 | 0.181792 | 0.559502 | -5.01198 |
| WP_OSTEOBLAST_DIFFERENTIATION_AND_RELATED_DISEASES | -0.03811 | -0.09432 | -1.34944 | 0.181817 | 0.559502 | -5.01208 |
| SA_PTEN_PATHWAY | -0.07808 | -0.10766 | -1.34914 | 0.181912 | 0.559502 | -5.01244 |
| REACTOME_GABA_B_RECEPTOR_ACTIVATION | 0.042696 | -0.1508 | 1.349025 | 0.181949 | 0.559502 | -5.01258 |
| REACTOME_MITOTIC_G1_PHASE_AND_G1_S_TRANSITION | -0.07519 | -0.1243 | -1.34852 | 0.18211 | 0.559502 | -5.0132 |
| REACTOME_CONJUGATION_OF_BENZOATE_WITH_GLYCINE | 0.147688 | 0.022702 | 1.348338 | 0.182169 | 0.559502 | -5.01342 |
| MORI_LARGE_PRE_BII_LYMPHOCYTE_DN | 0.064372 | -0.34319 | 1.34832 | 0.182175 | 0.559502 | -5.01344 |
| REACTOME_GABA_RECEPTOR_ACTIVATION | 0.057646 | -0.10636 | 1.348157 | 0.182227 | 0.559502 | -5.01364 |
| MMS_MOUSE_LYMPH_HIGH_4HRS_UP | -0.11859 | -0.01296 | -1.34808 | 0.182251 | 0.559502 | -5.01374 |
| WP_GLIOBLASTOMA_SIGNALING_PATHWAYS | -0.07111 | -0.03415 | -1.34773 | 0.182362 | 0.559572 | -5.01416 |
| LEE_LIVER_CANCER_MYC_TGFA_DN | 0.045032 | -0.13858 | 1.347176 | 0.182541 | 0.559768 | -5.01484 |
| WP_BREAST_CANCER_PATHWAY | -0.03556 | -0.13139 | -1.34699 | 0.182602 | 0.559768 | -5.01507 |
| REACTOME_HIV_ELONGATION_ARREST_AND_RECOVERY | -0.10873 | -0.14475 | -1.34659 | 0.182727 | 0.559833 | -5.01555 |
| JAATINEN_HEMATOPOIETIC_STEM_CELL_UP | -0.06649 | -0.05507 | -1.34634 | 0.182808 | 0.559833 | -5.01585 |
| OUELLET_CULTURED_OVARIAN_CANCER_INVASIVE_VS_LMP_DN | 0.064331 | 0.002986 | 1.345879 | 0.182957 | 0.559833 | -5.01642 |
| SCHLOSSER_MYC_TARGETS_REPRESSED_BY_SERUM | -0.11735 | -0.02363 | -1.34582 | 0.182975 | 0.559833 | -5.01649 |
| REACTOME_VITAMIN_B5_PANTOTHENATE_METABOLISM | -0.08059 | -0.05071 | -1.34518 | 0.18318 | 0.559962 | -5.01727 |
| BIOCARTA_LDL_PATHWAY | 0.132509 | -0.00354 | 1.345142 | 0.183193 | 0.559962 | -5.01732 |
| BOYAULT_LIVER_CANCER_SUBCLASS_G6_UP | -0.06185 | -0.00291 | -1.34424 | 0.183482 | 0.560158 | -5.01841 |
| REACTOME_ACTIVATION_OF_SMO | -0.08372 | 0.012608 | -1.34405 | 0.183543 | 0.560158 | -5.01864 |
| CHEOK_RESPONSE_TO_MERCAPTOPURINE_UP | -0.11987 | -0.07631 | -1.3439 | 0.183591 | 0.560158 | -5.01882 |
| REACTOME_SYNTHESIS_OF_ACTIVE_UBIQUITIN_ROLES_OF_E1_AND_E2_ENZYMES | -0.10129 | 0.004789 | -1.3431 | 0.183851 | 0.560158 | -5.01981 |
| LASTOWSKA_NEUROBLASTOMA_COPY_NUMBER_UP | -0.0588 | -0.08427 | -1.3426 | 0.184012 | 0.560158 | -5.02042 |
| SIG_IL4RECEPTOR_IN_B_LYPHOCYTES | -0.08306 | -0.06174 | -1.34193 | 0.184227 | 0.560158 | -5.02122 |
| WP_MICRORNAS_IN_CARDIOMYOCYTE_HYPERTROPHY | -0.04997 | -0.13029 | -1.34184 | 0.184257 | 0.560158 | -5.02134 |
| REACTOME_BASE_EXCISION_REPAIR_AP_SITE_FORMATION | -0.08917 | -0.04715 | -1.3407 | 0.184624 | 0.560158 | -5.02272 |
| COULOUARN_TEMPORAL_TGFB1_SIGNATURE_DN | 0.054234 | -0.05748 | 1.340656 | 0.184639 | 0.560158 | -5.02277 |
| RAMJAUN_APOPTOSIS_BY_TGFB1_VIA_MAPK1_UP | -0.09344 | -0.34986 | -1.34014 | 0.184805 | 0.560158 | -5.0234 |
| REACTOME_SYNAPTIC_ADHESION_LIKE_MOLECULES | -0.06001 | -0.21116 | -1.34002 | 0.184843 | 0.560158 | -5.02354 |
| EBAUER_TARGETS_OF_PAX3_FOXO1_FUSION_DN | 0.047213 | -0.02298 | 1.339997 | 0.184852 | 0.560158 | -5.02357 |
| BAELDE_DIABETIC_NEPHROPATHY_UP | 0.05513 | -0.15672 | 1.339693 | 0.18495 | 0.560158 | -5.02394 |
| OUYANG_PROSTATE_CANCER_MARKERS | 0.073588 | -0.05983 | 1.339574 | 0.184988 | 0.560158 | -5.02409 |
| REACTOME_NEGATIVE_EPIGENETIC_REGULATION_OF_RRNA_EXPRESSION | -0.0749 | -0.16228 | -1.33955 | 0.184995 | 0.560158 | -5.02411 |
| WP_TYPE_I_INTERFERON_INDUCTION_AND_SIGNALING_DURING_SARSCOV2_INFECTION | 0.093031 | -0.05824 | 1.339187 | 0.185114 | 0.560158 | -5.02456 |
| STEIN_ESRRA_TARGETS | -0.07762 | -0.09553 | -1.33913 | 0.185132 | 0.560158 | -5.02463 |
| KEGG_BIOSYNTHESIS_OF_UNSATURATED_FATTY_ACIDS | -0.09861 | -0.04143 | -1.33908 | 0.185147 | 0.560158 | -5.02468 |
| REACTOME_INSULIN_PROCESSING | -0.07463 | -0.00985 | -1.33867 | 0.185282 | 0.560158 | -5.02519 |
| VALK_AML_CLUSTER_3 | 0.087811 | -0.00883 | 1.338546 | 0.185321 | 0.560158 | -5.02533 |
| KEGG_ONE_CARBON_POOL_BY_FOLATE | -0.0897 | -0.17126 | -1.33842 | 0.185362 | 0.560158 | -5.02549 |
| REACTOME_DOWNSTREAM_SIGNAL_TRANSDUCTION | -0.09685 | -0.02846 | -1.33831 | 0.185398 | 0.560158 | -5.02562 |
| REACTOME_INTRA_GOLGI_AND_RETROGRADE_GOLGI_TO_ER_TRAFFIC | -0.07035 | -0.06204 | -1.33812 | 0.185461 | 0.560158 | -5.02585 |
| WP_GLUTATHIONE_METABOLISM | 0.082783 | -0.05782 | 1.337979 | 0.185505 | 0.560158 | -5.02602 |
| WEI_MYCN_TARGETS_WITH_E_BOX | -0.08116 | -0.06754 | -1.33798 | 0.185505 | 0.560158 | -5.02602 |
| ZHANG_GATA6_TARGETS_UP | 0.089833 | -0.00276 | 1.337535 | 0.185649 | 0.560158 | -5.02656 |
| WP_MRNA_PROTEIN_AND_METABOLITE_INDUCATION_PATHWAY_BY_CYCLOSPORIN_A | 0.116847 | -0.00167 | 1.336701 | 0.18592 | 0.560158 | -5.02757 |
| REACTOME_NGF_INDEPENDANT_TRKA_ACTIVATION | 0.124184 | -0.01004 | 1.336573 | 0.185961 | 0.560158 | -5.02772 |
| BIOCARTA_RAC1_PATHWAY | 0.083893 | -0.00282 | 1.336553 | 0.185968 | 0.560158 | -5.02775 |
| CHEN_ETV5_TARGETS_TESTIS | -0.07404 | 0.005397 | -1.33641 | 0.186013 | 0.560158 | -5.02792 |
| OSADA_ASCL1_TARGETS_DN | 0.080367 | -0.04674 | 1.336258 | 0.186064 | 0.560158 | -5.0281 |
| LINDSTEDT_DENDRITIC_CELL_MATURATION_D | 0.065692 | -0.2657 | 1.336034 | 0.186137 | 0.560158 | -5.02838 |
| REACTOME_TELOMERE_MAINTENANCE | -0.08716 | -0.07904 | -1.33578 | 0.18622 | 0.560158 | -5.02869 |
| WEBER_METHYLATED_ICP_IN_SPERM_UP | 0.120384 | -0.10744 | 1.335685 | 0.18625 | 0.560158 | -5.0288 |
| PHONG_TNF_RESPONSE_NOT_VIA_P38 | 0.056538 | -0.16335 | 1.335298 | 0.186376 | 0.560273 | -5.02927 |
| PYEON_HPV_POSITIVE_TUMORS_UP | -0.05914 | -0.07395 | -1.33484 | 0.186525 | 0.560456 | -5.02982 |
| PID_THROMBIN_PAR4_PATHWAY | 0.091003 | -0.00196 | 1.334344 | 0.186687 | 0.560677 | -5.03042 |
| YANG_BREAST_CANCER_ESR1_UP | 0.059789 | -0.07454 | 1.333332 | 0.187017 | 0.561403 | -5.03164 |
| FAELT_B_CLL_WITH_VH_REARRANGEMENTS_DN | -0.11358 | -0.05873 | -1.3324 | 0.187321 | 0.561937 | -5.03277 |
| KRIGE_RESPONSE_TO_TOSEDOSTAT_24HR_DN | -0.06496 | -0.10124 | -1.33206 | 0.187431 | 0.561937 | -5.03318 |
| WP_CYSTEINE_AND_METHIONINE_CATABOLISM | -0.09424 | -0.00668 | -1.33198 | 0.18746 | 0.561937 | -5.03328 |
| WANG_LMO4_TARGETS_UP | -0.06932 | -0.06799 | -1.33147 | 0.187626 | 0.561997 | -5.0339 |
| REACTOME_SELECTIVE_AUTOPHAGY | -0.08019 | -0.10066 | -1.33137 | 0.187656 | 0.561997 | -5.03401 |
| REACTOME_NRCAM_INTERACTIONS | 0.112029 | 0.025825 | 1.330964 | 0.187791 | 0.562135 | -5.0345 |
| BONOME_OVARIAN_CANCER_SURVIVAL_SUBOPTIMAL_DEBULKING | -0.05907 | -0.08369 | -1.3299 | 0.188139 | 0.562715 | -5.03578 |
| KONG_E2F1_TARGETS | 0.094929 | 0.00467 | 1.32977 | 0.188182 | 0.562715 | -5.03594 |
| WP_NANOMATERIAL_INDUCED_APOPTOSIS | -0.09292 | 0.004093 | -1.32956 | 0.188249 | 0.562715 | -5.03619 |
| KEGG_REGULATION_OF_ACTIN_CYTOSKELETON | 0.043713 | -0.03818 | 1.328151 | 0.188713 | 0.563797 | -5.03789 |
| REACTOME_VITAMIN_C_ASCORBATE_METABOLISM | 0.11159 | -0.01225 | 1.327219 | 0.189019 | 0.563797 | -5.03901 |
| MIKKELSEN_NPC_HCP_WITH_H3K27ME3 | 0.08522 | -0.03066 | 1.326338 | 0.189309 | 0.563797 | -5.04007 |
| REACTOME_DOWNSTREAM_SIGNALING_OF_ACTIVATED_FGFR2 | -0.06168 | -0.0247 | -1.3263 | 0.189321 | 0.563797 | -5.04011 |
| HOLLEMAN_PREDNISOLONE_RESISTANCE_ALL_DN | 0.06422 | -0.73056 | 1.325983 | 0.189426 | 0.563797 | -5.0405 |
| ZHENG_FOXP3_TARGETS_IN_THYMUS_DN | 0.08389 | -0.01011 | 1.32589 | 0.189457 | 0.563797 | -5.04061 |
| HOSHIDA_LIVER_CANCER_SUBCLASS_S2 | -0.08838 | -0.09682 | -1.32562 | 0.189545 | 0.563797 | -5.04093 |
| TSAI_RESPONSE_TO_IONIZING_RADIATION | 0.066265 | -0.10463 | 1.325415 | 0.189613 | 0.563797 | -5.04118 |
| REACTOME_CLEC7A_DECTIN_1_INDUCES_NFAT_ACTIVATION | -0.12844 | -0.00231 | -1.32535 | 0.189634 | 0.563797 | -5.04125 |
| REACTOME_PCNA_DEPENDENT_LONG_PATCH_BASE_EXCISION_REPAIR | -0.12858 | -0.00847 | -1.32462 | 0.189874 | 0.563797 | -5.04213 |
| PRAMOONJAGO_SOX4_TARGETS_UP | 0.091829 | -0.13508 | 1.324551 | 0.189898 | 0.563797 | -5.04222 |
| REACTOME_DISEASES_OF_BASE_EXCISION_REPAIR | -0.13042 | -0.01134 | -1.32444 | 0.189936 | 0.563797 | -5.04235 |
| BRACHAT_RESPONSE_TO_CAMPTOTHECIN_DN | -0.08467 | -0.16986 | -1.32419 | 0.190018 | 0.563797 | -5.04265 |
| WAKABAYASHI_ADIPOGENESIS_PPARG_BOUND_8D | -0.0612 | -0.07879 | -1.32411 | 0.190043 | 0.563797 | -5.04274 |
| REACTOME_ASYMMETRIC_LOCALIZATION_OF_PCP_PROTEINS | -0.07748 | -0.23259 | -1.32404 | 0.190068 | 0.563797 | -5.04283 |
| FU_INTERACT_WITH_ALKBH8 | -0.14948 | -0.00812 | -1.32371 | 0.190176 | 0.563797 | -5.04322 |
| REACTOME_NECTIN_NECL_TRANS_HETERODIMERIZATION | 0.103638 | 0.005641 | 1.323711 | 0.190176 | 0.563797 | -5.04322 |
| WP_MIRNAS_INVOLVEMENT_IN_THE_IMMUNE_RESPONSE_IN_SEPSIS | 0.06499 | -0.24952 | 1.323615 | 0.190208 | 0.563797 | -5.04334 |
| WP_CLASSICAL_PATHWAY_OF_STEROIDOGENESIS_WITH_GLUCOCORTICOID_AND_MINERALOCORTICOID_METABOLISM | 0.080706 | -0.25123 | 1.323351 | 0.190295 | 0.563797 | -5.04366 |
| BENPORATH_CYCLING_GENES | -0.06328 | -0.12468 | -1.32232 | 0.190635 | 0.564027 | -5.04489 |
| MURAKAMI_UV_RESPONSE_1HR_UP | 0.066146 | -0.06543 | 1.322148 | 0.190693 | 0.564027 | -5.0451 |
| DELASERNA_MYOD_TARGETS_UP | -0.04062 | -0.08334 | -1.32153 | 0.190898 | 0.564027 | -5.04584 |
| REACTOME_RECRUITMENT_OF_NUMA_TO_MITOTIC_CENTROSOMES | -0.05624 | -0.119 | -1.32136 | 0.190954 | 0.564027 | -5.04604 |
| ACEVEDO_LIVER_TUMOR_VS_NORMAL_ADJACENT_TISSUE_UP | -0.09964 | -0.15803 | -1.32122 | 0.191 | 0.564027 | -5.04621 |
| BURTON_ADIPOGENESIS_1 | 0.09713 | -0.18132 | 1.321204 | 0.191005 | 0.564027 | -5.04623 |
| REACTOME_ACTIVATION_OF_RAC1_DOWNSTREAM_OF_NMDARS | -0.12639 | -0.00216 | -1.32106 | 0.191054 | 0.564027 | -5.04641 |
| WILCOX_RESPONSE_TO_PROGESTERONE_DN | 0.059617 | -0.11361 | 1.320874 | 0.191115 | 0.564027 | -5.04662 |
| SENGUPTA_NASOPHARYNGEAL_CARCINOMA_DN | 0.047296 | -0.11839 | 1.320708 | 0.19117 | 0.564027 | -5.04682 |
| WP_GENES_ASSOCIATED_WITH_THE_DEVELOPMENT_OF_RHEUMATOID_ARTHRITIS | 0.073981 | -0.27618 | 1.319615 | 0.191533 | 0.56439 | -5.04813 |
| REACTOME_TP53_REGULATES_METABOLIC_GENES | -0.08686 | -0.06305 | -1.31954 | 0.191556 | 0.56439 | -5.04822 |
| BARRIER_CANCER_RELAPSE_NORMAL_SAMPLE_UP | -0.07792 | -0.04879 | -1.31923 | 0.191662 | 0.56439 | -5.0486 |
| LEE_LIVER_CANCER_SURVIVAL_DN | -0.07468 | -0.20615 | -1.31905 | 0.19172 | 0.56439 | -5.04881 |
| REACTOME_CYTOCHROME_P450_ARRANGED_BY_SUBSTRATE_TYPE | 0.0684 | -0.07894 | 1.318815 | 0.191799 | 0.56439 | -5.04909 |
| REACTOME_INITIAL_TRIGGERING_OF_COMPLEMENT | 0.03774 | -0.44432 | 1.318735 | 0.191825 | 0.56439 | -5.04918 |
| YAO_TEMPORAL_RESPONSE_TO_PROGESTERONE_CLUSTER_8 | 0.081287 | 0.007416 | 1.317288 | 0.192307 | 0.5655 | -5.05091 |
| LEE_CALORIE_RESTRICTION_MUSCLE_UP | 0.061478 | -0.20349 | 1.317068 | 0.19238 | 0.5655 | -5.05117 |
| REACTOME_RAP1_SIGNALLING | -0.07749 | -0.04338 | -1.31585 | 0.192785 | 0.566429 | -5.05262 |
| KEGG_PANTOTHENATE_AND_COA_BIOSYNTHESIS | -0.0911 | -0.05527 | -1.31557 | 0.192879 | 0.566442 | -5.05296 |
| REACTOME_SIGNALING_BY_FGFR4 | -0.05628 | -0.02089 | -1.31514 | 0.193023 | 0.566604 | -5.05347 |
| KAUFFMANN_MELANOMA_RELAPSE_UP | -0.07939 | -0.06407 | -1.31485 | 0.19312 | 0.566626 | -5.05382 |
| PODAR_RESPONSE_TO_ADAPHOSTIN_DN | 0.093057 | -0.04883 | 1.314422 | 0.193264 | 0.566789 | -5.05433 |
| CHEBOTAEV_GR_TARGETS_DN | -0.04857 | -0.11992 | -1.3139 | 0.193439 | 0.567042 | -5.05496 |
| REACTOME_SYNTHESIS_OF_IP2_IP_AND_INS_IN_THE_CYTOSOL | -0.07316 | -0.06951 | -1.31258 | 0.19388 | 0.567656 | -5.05652 |
| REACTOME_PI_METABOLISM | -0.06552 | -0.06073 | -1.31226 | 0.193988 | 0.567656 | -5.05691 |
| WP_PATHOGENESIS_OF_SARSCOV2_MEDIATED_BY_NSP9NSP10_COMPLEX | 0.061636 | -0.3583 | 1.312214 | 0.194003 | 0.567656 | -5.05696 |
| FONTAINE_THYROID_TUMOR_UNCERTAIN_MALIGNANCY_UP | 0.053217 | -0.0283 | 1.312207 | 0.194006 | 0.567656 | -5.05697 |
| REACTOME_SEMAPHORIN_INTERACTIONS | 0.07207 | -0.04935 | 1.31189 | 0.194112 | 0.567706 | -5.05735 |
| REACTOME_DEFECTIVE_CFTR_CAUSES_CYSTIC_FIBROSIS | -0.07792 | -0.28534 | -1.31107 | 0.194386 | 0.568246 | -5.05832 |
| WP_PLURIPOTENT_STEM_CELL_DIFFERENTIATION_PATHWAY | 0.068622 | -0.0063 | 1.310072 | 0.194723 | 0.5686 | -5.05951 |
| PID_SYNDECAN_3_PATHWAY | 0.069476 | -0.04968 | 1.309929 | 0.194771 | 0.5686 | -5.05968 |
| WANG_IMMORTALIZED_BY_HOXA9_AND_MEIS1_UP | 0.05131 | -0.11831 | 1.309917 | 0.194775 | 0.5686 | -5.05969 |
| FONTAINE_FOLLICULAR_THYROID_ADENOMA_DN | 0.05439 | -0.08061 | 1.30872 | 0.195178 | 0.569515 | -5.06111 |
| KRISHNAN_FURIN_TARGETS_DN | -0.1061 | -0.00891 | -1.30801 | 0.195416 | 0.569565 | -5.06195 |
| JAERVINEN_AMPLIFIED_IN_LARYNGEAL_CANCER | -0.08552 | -0.13091 | -1.30796 | 0.195436 | 0.569565 | -5.06202 |
| REACTOME_FGFRL1_MODULATION_OF_FGFR1_SIGNALING | 0.091592 | 0.008503 | 1.307872 | 0.195464 | 0.569565 | -5.06212 |
| BHAT_ESR1_TARGETS_VIA_AKT1_UP | 0.044565 | -0.09226 | 1.30698 | 0.195765 | 0.570182 | -5.06318 |
| TORCHIA_TARGETS_OF_EWSR1_FLI1_FUSION_TOP20_DN | -0.09312 | -0.01129 | -1.30671 | 0.195855 | 0.570185 | -5.06349 |
| REACTOME_IL_6_TYPE_CYTOKINE_RECEPTOR_LIGAND_INTERACTIONS | 0.072047 | 0.003621 | 1.306422 | 0.195953 | 0.570209 | -5.06384 |
| WNT_SIGNALING | -0.04287 | -0.04713 | -1.30554 | 0.196251 | 0.570644 | -5.06488 |
| WAKASUGI_HAVE_ZNF143_BINDING_SITES | -0.06314 | -0.19145 | -1.30545 | 0.196282 | 0.570644 | -5.06499 |
| MORI_LARGE_PRE_BII_LYMPHOCYTE_UP | -0.07934 | -0.10512 | -1.30485 | 0.196484 | 0.570796 | -5.06569 |
| WP_SYNAPTIC_VESICLE_PATHWAY | 0.047614 | -0.08359 | 1.304765 | 0.196514 | 0.570796 | -5.0658 |
| LEE_TARGETS_OF_PTCH1_AND_SUFU_DN | 0.047484 | -0.06326 | 1.303464 | 0.196955 | 0.570839 | -5.06734 |
| NIKOLSKY_BREAST_CANCER_21Q22_AMPLICON | -0.05837 | -0.21091 | -1.30321 | 0.197039 | 0.570839 | -5.06763 |
| REACTOME_DISEASES_ASSOCIATED_WITH_O_GLYCOSYLATION_OF_PROTEINS | 0.051659 | -0.11157 | 1.302934 | 0.197135 | 0.570839 | -5.06796 |
| FEVR_CTNNB1_TARGETS_UP | 0.034727 | -0.18489 | 1.302911 | 0.197142 | 0.570839 | -5.06799 |
| REACTOME_PROLACTIN_RECEPTOR_SIGNALING | -0.05962 | -0.00145 | -1.30278 | 0.197186 | 0.570839 | -5.06814 |
| REACTOME_SELENOAMINO_ACID_METABOLISM | -0.09113 | -0.13699 | -1.30271 | 0.19721 | 0.570839 | -5.06823 |
| REACTOME_APC_CDC20_MEDIATED_DEGRADATION_OF_NEK2A | -0.09753 | -0.01 | -1.3025 | 0.197282 | 0.570839 | -5.06848 |
| REACTOME_HYALURONAN_METABOLISM | 0.085662 | 0.000948 | 1.301976 | 0.19746 | 0.570839 | -5.0691 |
| WP_COMPLEMENT_AND_COAGULATION_CASCADES | 0.041473 | -0.28004 | 1.301821 | 0.197513 | 0.570839 | -5.06928 |
| KEGG_DNA_REPLICATION | -0.10888 | -0.03499 | -1.30182 | 0.197514 | 0.570839 | -5.06928 |
| MYLLYKANGAS_AMPLIFICATION_HOT_SPOT_25 | -0.08617 | -0.01783 | -1.30181 | 0.197515 | 0.570839 | -5.06929 |
| WP_PKCGAMMA_CALCIUM_SIGNALING_PATHWAY_IN_ATAXIA | 0.074922 | 0.015649 | 1.301359 | 0.19767 | 0.571027 | -5.06983 |
| WP_STING_PATHWAY_IN_KAWASAKILIKE_DISEASE_AND_COVID19 | 0.106635 | -0.04905 | 1.300556 | 0.197943 | 0.571522 | -5.07077 |
| BIOCARTA_KREB_PATHWAY | -0.14595 | 0.024097 | -1.30024 | 0.19805 | 0.571522 | -5.07114 |
| REACTOME_HEDGEHOG_LIGAND_BIOGENESIS | -0.07677 | -0.22617 | -1.30006 | 0.198111 | 0.571522 | -5.07135 |
| SARTIPY_BLUNTED_BY_INSULIN_RESISTANCE_UP | 0.108466 | -0.06146 | 1.299589 | 0.198273 | 0.57173 | -5.07191 |
| FARDIN_HYPOXIA_9 | -0.11947 | 0.002761 | -1.29885 | 0.198523 | 0.57201 | -5.07278 |
| REACTOME_SIGNALING_BY_FGFR1_IN_DISEASE | -0.05651 | -0.02333 | -1.29878 | 0.19855 | 0.57201 | -5.07287 |
| MIKKELSEN_ES_HCP_WITH_H3K27ME3 | 0.091112 | -0.00434 | 1.297815 | 0.198878 | 0.572481 | -5.074 |
| WP_TRANSCRIPTION_COFACTORS_SKI_AND_SKIL_PROTEIN_PARTNERS | -0.09405 | -0.00249 | -1.29777 | 0.198893 | 0.572481 | -5.07405 |
| YAO_TEMPORAL_RESPONSE_TO_PROGESTERONE_CLUSTER_3 | -0.10561 | -0.07165 | -1.29688 | 0.199198 | 0.572704 | -5.07511 |
| STEIN_ESRRA_TARGETS_RESPONSIVE_TO_ESTROGEN_DN | -0.05972 | -0.17083 | -1.29677 | 0.199235 | 0.572704 | -5.07523 |
| WP_METABOLISM_OF_SPINGOLIPIDS_IN_ER_AND_GOLGI_APPARATUS | 0.057406 | -0.04056 | 1.296755 | 0.199241 | 0.572704 | -5.07525 |
| REACTOME_REGULATION_OF_EXPRESSION_OF_SLITS_AND_ROBOS | -0.08293 | -0.16486 | -1.2964 | 0.199363 | 0.572798 | -5.07567 |
| JACKSON_DNMT1_TARGETS_UP | 0.0634 | -0.13863 | 1.295413 | 0.1997 | 0.573248 | -5.07683 |
| REACTOME_G_PROTEIN_MEDIATED_EVENTS | -0.05471 | -0.04759 | -1.29526 | 0.199754 | 0.573248 | -5.07702 |
| CUI_GLUCOSE_DEPRIVATION | 0.052512 | -0.17165 | 1.295151 | 0.19979 | 0.573248 | -5.07714 |
| REACTOME_RHOT1_GTPASE_CYCLE | -0.10933 | -0.14693 | -1.29407 | 0.200162 | 0.573907 | -5.07841 |
| WP_SPHINGOLIPID_PATHWAY | 0.059603 | -0.03119 | 1.293752 | 0.20027 | 0.573907 | -5.07878 |
| SCHAEFFER_PROSTATE_DEVELOPMENT_AND_CANCER_BOX5_UP | -0.13395 | -0.06996 | -1.29356 | 0.200336 | 0.573907 | -5.079 |
| NOUSHMEHR_GBM_SILENCED_BY_METHYLATION | 0.063346 | -0.0854 | 1.293431 | 0.200381 | 0.573907 | -5.07916 |
| SHIN_B_CELL_LYMPHOMA_CLUSTER_1 | 0.082135 | -0.11941 | 1.292999 | 0.200529 | 0.574074 | -5.07966 |
| REACTOME_TRANSPORT_OF_THE_SLBP_DEPENDANT_MATURE_MRNA | -0.10663 | -0.00745 | -1.29231 | 0.200768 | 0.574097 | -5.08048 |
| REACTOME_PROTEIN_UBIQUITINATION | -0.08265 | -0.02637 | -1.29219 | 0.200808 | 0.574097 | -5.08062 |
| WALLACE_PROSTATE_CANCER_RACE_DN | -0.03922 | -0.11656 | -1.29211 | 0.200834 | 0.574097 | -5.08071 |
| FLORIO_HUMAN_NEOCORTEX | 0.096408 | 0.021341 | 1.291927 | 0.200898 | 0.574097 | -5.08092 |
| KEGG_RETINOL_METABOLISM | 0.070316 | -0.08382 | 1.291655 | 0.200992 | 0.574107 | -5.08124 |
| REACTOME_CGMP_EFFECTS | -0.08738 | 0.01126 | -1.29123 | 0.201138 | 0.574267 | -5.08174 |
| WP_ADIPOGENESIS | 0.052506 | -0.1106 | 1.290384 | 0.20143 | 0.574598 | -5.08273 |
| MIKI_COEXPRESSED_WITH_CYP19A1 | 0.105072 | 0.016477 | 1.29037 | 0.201434 | 0.574598 | -5.08275 |
| SPIELMAN_LYMPHOBLAST_EUROPEAN_VS_ASIAN_2FC_UP | 0.069162 | -0.01492 | 1.289668 | 0.201677 | 0.575031 | -5.08357 |
| WP_1Q211_COPY_NUMBER_VARIATION_SYNDROME | -0.05777 | -0.08597 | -1.28919 | 0.20184 | 0.575079 | -5.08412 |
| REACTOME_MRNA_DECAY_BY_3_TO_5_EXORIBONUCLEASE | -0.11368 | -0.21058 | -1.28881 | 0.201974 | 0.575079 | -5.08458 |
| REACTOME_PHASE_1_INACTIVATION_OF_FAST_NA_CHANNELS | -0.13119 | 0.011532 | -1.28864 | 0.202033 | 0.575079 | -5.08478 |
| WP_P53_TRANSCRIPTIONAL_GENE_NETWORK | 0.059272 | -0.05077 | 1.288367 | 0.202126 | 0.575079 | -5.08509 |
| SOTIRIOU_BREAST_CANCER_GRADE_1_VS_3_DN | -0.08712 | -0.03456 | -1.28831 | 0.202145 | 0.575079 | -5.08515 |
| YU_MYC_TARGETS_UP | -0.11188 | -0.01487 | -1.28794 | 0.202276 | 0.575114 | -5.0856 |
| REACTOME_AKT_PHOSPHORYLATES_TARGETS_IN_THE_NUCLEUS | -0.07693 | -0.07903 | -1.28758 | 0.202399 | 0.575114 | -5.08601 |
| REACTOME_MTORC1_MEDIATED_SIGNALLING | -0.10033 | -0.0089 | -1.28749 | 0.202428 | 0.575114 | -5.08611 |
| RUTELLA_RESPONSE_TO_HGF_VS_CSF2RB_AND_IL4_UP | 0.071554 | -0.15786 | 1.287038 | 0.202587 | 0.575191 | -5.08664 |
| BIOCARTA_AHSP_PATHWAY | 0.088721 | 0.004967 | 1.286894 | 0.202636 | 0.575191 | -5.08681 |
| WP_THYROXINE_THYROID_HORMONE_PRODUCTION | 0.130787 | -0.1231 | 1.285787 | 0.20302 | 0.575538 | -5.0881 |
| NABA_ECM_GLYCOPROTEINS | 0.058368 | -0.08565 | 1.285715 | 0.203045 | 0.575538 | -5.08819 |
| WANG_CLIM2_TARGETS_DN | -0.06728 | -0.11107 | -1.28492 | 0.203321 | 0.575538 | -5.08912 |
| WP_MAMMARY_GLAND_DEVELOPMENT_PATHWAY_PREGNANCY_AND_LACTATION_STAGE_3_OF_4 | 0.060791 | -0.02345 | 1.284866 | 0.20334 | 0.575538 | -5.08918 |
| DIAZ_CHRONIC_MYELOGENOUS_LEUKEMIA_UP | -0.09654 | -0.06813 | -1.2848 | 0.203364 | 0.575538 | -5.08926 |
| HOLLEMAN_DAUNORUBICIN_B_ALL_DN | -0.12643 | -0.0806 | -1.28449 | 0.203469 | 0.575538 | -5.08961 |
| REACTOME_CELLULAR_RESPONSE_TO_CHEMICAL_STRESS | -0.07689 | -0.15246 | -1.28426 | 0.20355 | 0.575538 | -5.08988 |
| REACTOME_PROCESSING_OF_CAPPED_INTRONLESS_PRE_MRNA | -0.10356 | -0.0463 | -1.28409 | 0.203611 | 0.575538 | -5.09009 |
| MATZUK_SPERMATOZOA | 0.033199 | -0.11719 | 1.283983 | 0.203647 | 0.575538 | -5.09021 |
| WEST_ADRENOCORTICAL_CARCINOMA_VS_ADENOMA_UP | 0.057456 | -0.00023 | 1.283936 | 0.203663 | 0.575538 | -5.09026 |
| RHEIN_ALL_GLUCOCORTICOID_THERAPY_DN | -0.09038 | -0.11162 | -1.28336 | 0.203864 | 0.57585 | -5.09094 |
| DAZARD_UV_RESPONSE_CLUSTER_G2 | 0.065013 | -0.34743 | 1.282657 | 0.204108 | 0.576196 | -5.09175 |
| GRYDER_PAX3FOXO1_TOP_ENHANCERS | -0.07432 | -0.05136 | -1.28249 | 0.204168 | 0.576196 | -5.09195 |
| IWANAGA_E2F1_TARGETS_INDUCED_BY_SERUM | -0.10364 | -0.002 | -1.28083 | 0.204747 | 0.576668 | -5.09388 |
| PUJANA_BREAST_CANCER_WITH_BRCA1_MUTATED_UP | -0.11254 | -0.01712 | -1.28074 | 0.204779 | 0.576668 | -5.09399 |
| GOLUB_ALL_VS_AML_UP | -0.12831 | 0.000868 | -1.28046 | 0.204877 | 0.576668 | -5.09432 |
| WP_SPHINGOLIPID_METABOLISM_INTEGRATED_PATHWAY | 0.060561 | -0.03362 | 1.280099 | 0.205001 | 0.576668 | -5.09473 |
| SIG_PIP3_SIGNALING_IN_B_LYMPHOCYTES | -0.05643 | -0.23719 | -1.28005 | 0.205017 | 0.576668 | -5.09478 |
| WP_TYPE_II_INTERFERON_SIGNALING_IFNG | 0.060482 | -0.35348 | 1.280028 | 0.205026 | 0.576668 | -5.09481 |
| REACTOME_TBC_RABGAPS | -0.09824 | -0.00836 | -1.27993 | 0.20506 | 0.576668 | -5.09492 |
| BENPORATH_PROLIFERATION | -0.09385 | -0.02292 | -1.27986 | 0.205086 | 0.576668 | -5.09501 |
| BIOCARTA_GABA_PATHWAY | -0.08121 | -0.13898 | -1.27885 | 0.205439 | 0.576668 | -5.09618 |
| PARK_TRETINOIN_RESPONSE_AND_RARA_PLZF_FUSION | 0.084972 | -0.18189 | 1.278376 | 0.205604 | 0.576668 | -5.09673 |
| REACTOME_METABOLISM_OF_STEROIDS | -0.04044 | -0.05862 | -1.27831 | 0.205628 | 0.576668 | -5.09681 |
| VANOEVELEN_MYOGENESIS_SIN3A_TARGETS | -0.05097 | -0.19468 | -1.27813 | 0.20569 | 0.576668 | -5.09701 |
| DE_YY1_TARGETS_DN | -0.08326 | -0.03635 | -1.27812 | 0.205694 | 0.576668 | -5.09703 |
| ROZANOV_MMP14_TARGETS_SUBSET | 0.104597 | -0.00824 | 1.278105 | 0.205699 | 0.576668 | -5.09705 |
| DARWICHE_SKIN_TUMOR_PROMOTER_DN | -0.04031 | -0.06831 | -1.27762 | 0.205868 | 0.576668 | -5.0976 |
| WILLIAMS_ESR2_TARGETS_UP | -0.08117 | -0.09979 | -1.27751 | 0.205907 | 0.576668 | -5.09773 |
| REACTOME_HIV_INFECTION | -0.08132 | -0.14349 | -1.27748 | 0.205918 | 0.576668 | -5.09777 |
| TONG_INTERACT_WITH_PTTG1 | -0.06597 | -0.17977 | -1.27709 | 0.206054 | 0.576668 | -5.09822 |
| REACTOME_BUDDING_AND_MATURATION_OF_HIV_VIRION | -0.10355 | -0.05941 | -1.2769 | 0.206122 | 0.576668 | -5.09845 |
| TAVOR_CEBPA_TARGETS_UP | 0.069721 | -0.161 | 1.276827 | 0.206147 | 0.576668 | -5.09853 |
| BROWNE_HCMV_INFECTION_10HR_UP | 0.045613 | -0.16743 | 1.275824 | 0.206499 | 0.577158 | -5.09969 |
| BIOCARTA_ERBB3_PATHWAY | 0.154343 | 0.016209 | 1.275205 | 0.206717 | 0.577158 | -5.10041 |
| REACTOME_DEGRADATION_OF_AXIN | -0.07749 | -0.24657 | -1.27513 | 0.206744 | 0.577158 | -5.1005 |
| GUTIERREZ_WALDENSTROEMS_MACROGLOBULINEMIA_1_DN | 0.128059 | 0.006423 | 1.275075 | 0.206763 | 0.577158 | -5.10056 |
| CHIARADONNA_NEOPLASTIC_TRANSFORMATION_CDC25_UP | 0.066216 | -0.06003 | 1.274995 | 0.206791 | 0.577158 | -5.10065 |
| REACTOME_GROWTH_HORMONE_RECEPTOR_SIGNALING | 0.077525 | -0.00557 | 1.274781 | 0.206866 | 0.577158 | -5.1009 |
| ENK_UV_RESPONSE_KERATINOCYTE_DN | -0.09325 | -0.05607 | -1.27441 | 0.206995 | 0.577264 | -5.10132 |
| MCBRYAN_TERMINAL_END_BUD_DN | 0.125291 | -0.00811 | 1.273766 | 0.207224 | 0.577649 | -5.10207 |
| YAGI_AML_SURVIVAL | -0.06542 | -0.06699 | -1.27336 | 0.207366 | 0.577793 | -5.10254 |
| ONDER_CDH1_TARGETS_1_DN | 0.058111 | -0.0407 | 1.272749 | 0.207582 | 0.577934 | -5.10325 |
| VANTVEER_BREAST_CANCER_METASTASIS_DN | -0.08153 | -0.04357 | -1.27244 | 0.207689 | 0.577934 | -5.1036 |
| WP_ULTRACONSERVED_REGION_339_MODULATION_OF_TUMOR_SUPPRESSOR_MICRORNAS_IN_CANCER | 0.146386 | 0.0375 | 1.272445 | 0.207689 | 0.577934 | -5.1036 |
| CERIBELLI_PROMOTERS_INACTIVE_AND_BOUND_BY_NFY | 0.064038 | -0.07201 | 1.272072 | 0.207821 | 0.577991 | -5.10403 |
| REACTOME_ACTIVATION_OF_TRKA_RECEPTORS | 0.112237 | -0.00835 | 1.271841 | 0.207902 | 0.577991 | -5.1043 |
| LINSLEY_MIR16_TARGETS | -0.05633 | -0.17043 | -1.27154 | 0.208008 | 0.577991 | -5.10464 |
| FRASOR_RESPONSE_TO_SERM_OR_FULVESTRANT_DN | -0.09916 | -0.00592 | -1.27122 | 0.208124 | 0.577991 | -5.10502 |
| MORI_EMU_MYC_LYMPHOMA_BY_ONSET_TIME_UP | -0.07085 | -0.08151 | -1.27096 | 0.208213 | 0.577991 | -5.10531 |
| WEBER_METHYLATED_LCP_IN_SPERM_DN | 0.140817 | 0.017035 | 1.270657 | 0.208321 | 0.577991 | -5.10567 |
| REACTOME_DEFECTIVE_RIPK1_MEDIATED_REGULATED_NECROSIS | -0.12617 | 0.035577 | -1.27059 | 0.208346 | 0.577991 | -5.10575 |
| KEGG_HUNTINGTONS_DISEASE | -0.06808 | -0.10841 | -1.26983 | 0.208612 | 0.578139 | -5.10661 |
| STEIN_ESRRA_TARGETS_UP | -0.07388 | -0.07078 | -1.26975 | 0.208642 | 0.578139 | -5.10671 |
| WP_15Q112_COPY_NUMBER_VARIATION_SYNDROME | -0.06452 | -0.39759 | -1.26967 | 0.208671 | 0.578139 | -5.10681 |
| TERAO_AOX4_TARGETS_SKIN_UP | 0.04368 | -0.33381 | 1.269173 | 0.208847 | 0.578153 | -5.10738 |
| MAGRANGEAS_MULTIPLE_MYELOMA_IGLL_VS_IGLK_DN | 0.054513 | -0.30695 | 1.26914 | 0.208858 | 0.578153 | -5.10741 |
| MEISSNER_NPC_HCP_WITH_H3K27ME3 | 0.082896 | -0.0234 | 1.268482 | 0.209092 | 0.578509 | -5.10817 |
| BOSCO_TH1_CYTOTOXIC_MODULE | 0.057927 | -0.04637 | 1.268265 | 0.209169 | 0.578509 | -5.10842 |
| PID_HIV_NEF_PATHWAY | -0.07699 | -0.24326 | -1.2668 | 0.20969 | 0.579568 | -5.11011 |
| ZEILSTRA_CD44_TARGETS_DN | 0.061363 | -0.43828 | 1.266673 | 0.209734 | 0.579568 | -5.11026 |
| MEBARKI_HCC_PROGENITOR_WNT_DN | 0.044203 | -0.09456 | 1.265405 | 0.210185 | 0.580559 | -5.11171 |
| WP_MYD88_DISTINCT_INPUTOUTPUT_PATHWAY | 0.103215 | -0.00421 | 1.264796 | 0.210402 | 0.580559 | -5.11241 |
| REACTOME_REGULATION_OF_HMOX1_EXPRESSION_AND_ACTIVITY | -0.07492 | -0.2688 | -1.26465 | 0.210453 | 0.580559 | -5.11258 |
| BIOCARTA_IL10_PATHWAY | 0.07798 | -0.33452 | 1.264238 | 0.210601 | 0.580559 | -5.11306 |
| REACTOME_MITOCHONDRIAL_TRNA_AMINOACYLATION | -0.10571 | -0.10509 | -1.26413 | 0.210639 | 0.580559 | -5.11318 |
| REACTOME_SIGNALING_BY_PDGFRA_TRANSMEMBRANE_JUXTAMEMBRANE_AND_KINASE_DOMAIN_MUTANTS | -0.11233 | -0.05384 | -1.26413 | 0.210639 | 0.580559 | -5.11318 |
| WP_SPHINGOLIPID_METABOLISM_OVERVIEW | 0.060586 | -0.03733 | 1.26376 | 0.210771 | 0.580576 | -5.11361 |
| REACTOME_CLASS_I_MHC_MEDIATED_ANTIGEN_PROCESSING_PRESENTATION | -0.06039 | -0.17626 | -1.2636 | 0.210828 | 0.580576 | -5.11379 |
| SARRIO_EPITHELIAL_MESENCHYMAL_TRANSITION_DN | 0.067991 | -0.09413 | 1.263141 | 0.210992 | 0.580776 | -5.11431 |
| MIKKELSEN_ES_LCP_WITH_H3K27ME3 | 0.114735 | -0.00075 | 1.262339 | 0.211279 | 0.580796 | -5.11524 |
| WP_PHOTODYNAMIC_THERAPYINDUCED_NFE2L2_NRF2_SURVIVAL_SIGNALING | 0.068464 | -0.10682 | 1.262209 | 0.211325 | 0.580796 | -5.11538 |
| LOPES_METHYLATED_IN_COLON_CANCER_DN | -0.05361 | -0.0295 | -1.26198 | 0.211407 | 0.580796 | -5.11565 |
| HOEGERKORP_CD44_TARGETS_DIRECT_DN | 0.085105 | -0.06399 | 1.261683 | 0.211513 | 0.580796 | -5.11599 |
| REACTOME_INTERLEUKIN_RECEPTOR_SHC_SIGNALING | 0.057784 | -0.04098 | 1.261507 | 0.211576 | 0.580796 | -5.11619 |
| KEGG_WNT_SIGNALING_PATHWAY | -0.03983 | -0.09036 | -1.26135 | 0.211634 | 0.580796 | -5.11637 |
| CUI_TCF21_TARGETS_2_DN | -0.07092 | -0.06886 | -1.2612 | 0.211685 | 0.580796 | -5.11654 |
| REACTOME_CHAPERONE_MEDIATED_AUTOPHAGY | 0.09891 | 0.005703 | 1.261079 | 0.211729 | 0.580796 | -5.11668 |
| DODD_NASOPHARYNGEAL_CARCINOMA_UP | 0.033125 | -0.08262 | 1.260605 | 0.211899 | 0.580833 | -5.11722 |
| REACTOME_NUCLEAR_RECEPTOR_TRANSCRIPTION_PATHWAY | 0.054731 | -0.07812 | 1.260386 | 0.211978 | 0.580833 | -5.11747 |
| HAN_JNK_SINGALING_UP | 0.067259 | -0.10485 | 1.260167 | 0.212056 | 0.580833 | -5.11773 |
| PARK_HSC_AND_MULTIPOTENT_PROGENITORS | -0.09636 | -0.0501 | -1.26002 | 0.212108 | 0.580833 | -5.11789 |
| REACTOME_NONSENSE_MEDIATED_DECAY_NMD | -0.09383 | -0.11291 | -1.25941 | 0.212328 | 0.581186 | -5.11859 |
| REACTOME_NEGATIVE_REGULATION_OF_TCF_DEPENDENT_SIGNALING_BY_WNT_LIGAND_ANTAGONISTS | -0.07717 | -0.0523 | -1.25911 | 0.212436 | 0.58123 | -5.11894 |
| CAMPS_COLON_CANCER_COPY_NUMBER_UP | 0.03701 | -0.1659 | 1.25863 | 0.212608 | 0.58145 | -5.11948 |
| LEE_CALORIE_RESTRICTION_NEOCORTEX_DN | -0.0512 | -0.04407 | -1.25767 | 0.212954 | 0.582113 | -5.12059 |
| IIZUKA_LIVER_CANCER_EARLY_RECURRENCE | 0.105438 | -0.321 | 1.257447 | 0.213033 | 0.582113 | -5.12084 |
| LIN_NPAS4_TARGETS_UP | -0.05625 | -0.16705 | -1.25653 | 0.213363 | 0.582158 | -5.12189 |
| CHICAS_RB1_TARGETS_GROWING | -0.06242 | -0.04527 | -1.25648 | 0.213382 | 0.582158 | -5.12195 |
| DER_IFN_ALPHA_RESPONSE_DN | -0.16728 | -0.04593 | -1.25601 | 0.213551 | 0.582158 | -5.12248 |
| GARGALOVIC_RESPONSE_TO_OXIDIZED_PHOSPHOLIPIDS_TAN_DN | -0.11541 | -0.02344 | -1.25596 | 0.213568 | 0.582158 | -5.12253 |
| YIH_RESPONSE_TO_ARSENITE_C4 | 0.070145 | -0.17799 | 1.255901 | 0.213589 | 0.582158 | -5.1226 |
| REACTOME_DEGRADATION_OF_GLI1_BY_THE_PROTEASOME | -0.07661 | -0.23957 | -1.25588 | 0.213598 | 0.582158 | -5.12263 |
| YEMELYANOV_GR_TARGETS_DN | 0.091336 | -0.07121 | 1.254966 | 0.213926 | 0.582803 | -5.12367 |
| REACTOME_COPI_MEDIATED_ANTEROGRADE_TRANSPORT | -0.07912 | -0.02208 | -1.25435 | 0.214148 | 0.583157 | -5.12437 |
| BIOCARTA_CALCINEURIN_PATHWAY | -0.09967 | -0.0493 | -1.25401 | 0.21427 | 0.583239 | -5.12475 |
| REACTOME_ESTROGEN_DEPENDENT_GENE_EXPRESSION | -0.08496 | -0.021 | -1.25311 | 0.214598 | 0.583882 | -5.12579 |
| REACTOME_PREVENTION_OF_PHAGOSOMAL_LYSOSOMAL_FUSION | 0.131538 | 0.004278 | 1.252517 | 0.214811 | 0.584128 | -5.12646 |
| ELVIDGE_HYPOXIA_BY_DMOG_UP | 0.083396 | -0.05656 | 1.252139 | 0.214948 | 0.584128 | -5.12689 |
| PAPASPYRIDONOS_UNSTABLE_ATEROSCLEROTIC_PLAQUE_DN | -0.10385 | -0.10323 | -1.25169 | 0.215109 | 0.584128 | -5.1274 |
| BIOCARTA_HBX_PATHWAY | -0.10476 | -0.10767 | -1.25164 | 0.215128 | 0.584128 | -5.12746 |
| RICKMAN_HEAD_AND_NECK_CANCER_C | 0.054015 | -0.14771 | 1.25159 | 0.215147 | 0.584128 | -5.12752 |
| HASLINGER_B_CLL_WITH_6Q21_DELETION | -0.10838 | -0.06684 | -1.25032 | 0.215608 | 0.585057 | -5.12896 |
| NIKOLSKY_BREAST_CANCER_1Q32_AMPLICON | 0.088094 | -0.00269 | 1.250002 | 0.215723 | 0.585057 | -5.12932 |
| PID_RET_PATHWAY | -0.07234 | -0.07722 | -1.24945 | 0.215921 | 0.585057 | -5.12994 |
| REACTOME_ER_QUALITY_CONTROL_COMPARTMENT_ERQC | -0.0885 | -0.21433 | -1.24918 | 0.216022 | 0.585057 | -5.13026 |
| WP_PI3KAKT_SIGNALING_PATHWAY | 0.030395 | -0.05443 | 1.249136 | 0.216037 | 0.585057 | -5.1303 |
| WP_AIRWAY_SMOOTH_MUSCLE_CELL_CONTRACTION | -0.09264 | 0.004867 | -1.24913 | 0.216041 | 0.585057 | -5.13032 |
| PID_LKB1_PATHWAY | 0.058928 | -0.07809 | 1.247915 | 0.216481 | 0.585795 | -5.13169 |
| BURTON_ADIPOGENESIS_PEAK_AT_8HR | 0.080446 | -0.02675 | 1.247528 | 0.216622 | 0.585795 | -5.13213 |
| FARMER_BREAST_CANCER_CLUSTER_5 | -0.09767 | -0.06414 | -1.24734 | 0.21669 | 0.585795 | -5.13234 |
| REACTOME_PI3K_AKT_ACTIVATION | -0.10936 | -0.00142 | -1.24698 | 0.21682 | 0.585795 | -5.13275 |
| PID_RHODOPSIN_PATHWAY | 0.077303 | -0.09285 | 1.246564 | 0.216973 | 0.585795 | -5.13322 |
| REACTOME_RHOG_GTPASE_CYCLE | 0.049102 | -0.03933 | 1.246295 | 0.217071 | 0.585795 | -5.13353 |
| BOQUEST_STEM_CELL_CULTURED_VS_FRESH_DN | 0.084265 | -0.06305 | 1.246227 | 0.217096 | 0.585795 | -5.1336 |
| REACTOME_SUMOYLATION_OF_RNA_BINDING_PROTEINS | -0.07676 | -0.11882 | -1.24608 | 0.217149 | 0.585795 | -5.13377 |
| STEARMAN_LUNG_CANCER_EARLY_VS_LATE_UP | -0.08281 | -0.07862 | -1.24596 | 0.217192 | 0.585795 | -5.1339 |
| KAMMINGA_EZH2_TARGETS | -0.11962 | -0.0245 | -1.24585 | 0.217234 | 0.585795 | -5.13403 |
| REACTOME_MAPK6_MAPK4_SIGNALING | -0.06634 | -0.20128 | -1.24559 | 0.217327 | 0.585799 | -5.13432 |
| YANG_BCL3_TARGETS_DN | 0.073882 | -0.00981 | 1.244864 | 0.217594 | 0.58619 | -5.13515 |
| HALMOS_CEBPA_TARGETS_UP | 0.074325 | -0.00322 | 1.244476 | 0.217735 | 0.58619 | -5.13559 |
| REACTOME_TRANSPORT_OF_MATURE_TRANSCRIPT_TO_CYTOPLASM | -0.08778 | -0.07948 | -1.24444 | 0.217749 | 0.58619 | -5.13563 |
| BIOCARTA_CIRCADIAN_PATHWAY | 0.114281 | -0.00591 | 1.244172 | 0.217846 | 0.586204 | -5.13593 |
| RODRIGUES_NTN1_AND_DCC_TARGETS | 0.068621 | -0.0624 | 1.243039 | 0.218261 | 0.587032 | -5.13721 |
| REACTOME_RRNA_PROCESSING | -0.08213 | -0.13754 | -1.24283 | 0.218338 | 0.587032 | -5.13745 |
| FUJII_YBX1_TARGETS_DN | -0.05467 | -0.17183 | -1.24245 | 0.218477 | 0.587157 | -5.13788 |
| REACTOME_ATF6_ATF6_ALPHA_ACTIVATES_CHAPERONES | -0.12727 | ####### | -1.24204 | 0.218626 | 0.587199 | -5.13834 |
| SANDERSON_PPARA_TARGETS | -0.09879 | -0.07245 | -1.2419 | 0.218677 | 0.587199 | -5.1385 |
| REACTOME_LIGAND_RECEPTOR_INTERACTIONS | -0.10932 | -0.01859 | -1.24066 | 0.219135 | 0.58818 | -5.1399 |
| KUNINGER_IGF1_VS_PDGFB_TARGETS_DN | 0.058232 | -0.04493 | 1.240286 | 0.21927 | 0.588295 | -5.14032 |
| LEE_LIVER_CANCER_DENA_DN | 0.04723 | -0.05723 | 1.237732 | 0.22021 | 0.590568 | -5.1432 |
| YANG_BREAST_CANCER_ESR1_LASER_UP | -0.06102 | -0.03557 | -1.23712 | 0.220435 | 0.590736 | -5.14389 |
| SETLUR_PROSTATE_CANCER_TMPRSS2_ERG_FUSION_UP | -0.06322 | -0.06022 | -1.23706 | 0.220458 | 0.590736 | -5.14396 |
| REACTOME_INTERFERON_GAMMA_SIGNALING | 0.033777 | -0.47473 | 1.236491 | 0.220668 | 0.591048 | -5.14459 |
| WP_REGULATION_OF_ACTIN_CYTOSKELETON | 0.036753 | -0.03874 | 1.235691 | 0.220963 | 0.591427 | -5.14549 |
| WP_OSTEOCLAST_SIGNALING | 0.084918 | -0.05597 | 1.235468 | 0.221046 | 0.591427 | -5.14574 |
| KEGG_SNARE_INTERACTIONS_IN_VESICULAR_TRANSPORT | -0.08583 | -0.01016 | -1.23535 | 0.221088 | 0.591427 | -5.14587 |
| MENSE_HYPOXIA_UP | 0.078279 | -0.05106 | 1.234864 | 0.221269 | 0.591662 | -5.14642 |
| REACTOME_CYCLIN_A_CDK2_ASSOCIATED_EVENTS_AT_S_PHASE_ENTRY | -0.07176 | -0.1866 | -1.23413 | 0.221541 | 0.591664 | -5.14725 |
| KEGG_DRUG_METABOLISM_CYTOCHROME_P450 | 0.048976 | -0.11121 | 1.23362 | 0.221729 | 0.591664 | -5.14782 |
| WANG_BARRETTS_ESOPHAGUS_AND_ESOPHAGUS_CANCER_UP | 0.043392 | -0.32862 | 1.233343 | 0.221832 | 0.591664 | -5.14813 |
| ELVIDGE_HYPOXIA_UP | 0.072336 | -0.07985 | 1.233061 | 0.221936 | 0.591664 | -5.14845 |
| REACTOME_BETA_OXIDATION_OF_OCTANOYL_COA_TO_HEXANOYL_COA | -0.16543 | -0.01072 | -1.23298 | 0.221967 | 0.591664 | -5.14854 |
| REACTOME_SYNTHESIS_OF_PE | -0.09188 | 0.009195 | -1.23295 | 0.221977 | 0.591664 | -5.14857 |
| BAKER_HEMATOPOIESIS_STAT3_TARGETS | 0.100617 | -0.00188 | 1.232939 | 0.221982 | 0.591664 | -5.14858 |
| HAHTOLA_MYCOSIS_FUNGOIDES_CD4_DN | -0.10645 | -0.02683 | -1.23285 | 0.222014 | 0.591664 | -5.14868 |
| KEGG_OLFACTORY_TRANSDUCTION | 0.070664 | -0.25241 | 1.232507 | 0.222142 | 0.591758 | -5.14907 |
| MEISSNER_ES_ICP_WITH_H3K4ME3_AND_H3K27ME3 | 0.095905 | 0.009527 | 1.232226 | 0.222246 | 0.591788 | -5.14938 |
| HOLLEMAN_ASPARAGINASE_RESISTANCE_B_ALL_DN | 0.090097 | 0.012296 | 1.231455 | 0.222532 | 0.592198 | -5.15025 |
| REACTOME_REGULATION_OF_PTEN_MRNA_TRANSLATION | -0.0882 | -0.08417 | -1.23103 | 0.22269 | 0.592198 | -5.15072 |
| HATADA_METHYLATED_IN_LUNG_CANCER_DN | 0.078364 | -0.07312 | 1.230603 | 0.222849 | 0.592198 | -5.1512 |
| BIOCARTA_TUBBY_PATHWAY | 0.130195 | -0.00463 | 1.230594 | 0.222852 | 0.592198 | -5.15121 |
| SENGUPTA_NASOPHARYNGEAL_CARCINOMA_WITH_LMP1_UP | -0.04954 | -0.04131 | -1.23034 | 0.222945 | 0.592198 | -5.15149 |
| REACTOME_INNATE_IMMUNE_SYSTEM | 0.045857 | -0.16736 | 1.230309 | 0.222958 | 0.592198 | -5.15153 |
| MATZUK_PREOVULATORY_FOLLICLE | -0.08931 | -0.00524 | -1.22968 | 0.223192 | 0.59238 | -5.15223 |
| REACTOME_TP53_REGULATES_TRANSCRIPTION_OF_DNA_REPAIR_GENES | -0.05802 | -0.27176 | -1.22962 | 0.223213 | 0.59238 | -5.1523 |
| MARSON_FOXP3_TARGETS_DN | 0.084395 | -0.06279 | 1.227583 | 0.223973 | 0.593568 | -5.15458 |
| REACTOME_INTERLEUKIN_17_SIGNALING | 0.055302 | -0.00891 | 1.227173 | 0.224126 | 0.593568 | -5.15504 |
| REACTOME_GLUCAGON_SIGNALING_IN_METABOLIC_REGULATION | -0.04429 | -0.10596 | -1.22709 | 0.224157 | 0.593568 | -5.15513 |
| BIOCARTA_LAIR_PATHWAY | 0.062568 | -0.27407 | 1.226981 | 0.224198 | 0.593568 | -5.15525 |
| WP_CELLS_AND_MOLECULES_INVOLVED_IN_LOCAL_ACUTE_INFLAMMATORY_RESPONSE | 0.062568 | -0.27407 | 1.226981 | 0.224198 | 0.593568 | -5.15525 |
| REACTOME_CREB_PHOSPHORYLATION | -0.08698 | -0.09315 | -1.22692 | 0.22422 | 0.593568 | -5.15532 |
| KEGG_SPHINGOLIPID_METABOLISM | 0.043416 | -0.21412 | 1.225695 | 0.224679 | 0.594204 | -5.15669 |
| REACTOME_NRAGE_SIGNALS_DEATH_THROUGH_JNK | 0.038812 | -0.08003 | 1.225562 | 0.224728 | 0.594204 | -5.15683 |
| PICCALUGA_ANGIOIMMUNOBLASTIC_LYMPHOMA_DN | 0.093105 | -0.01468 | 1.225529 | 0.224741 | 0.594204 | -5.15687 |
| LEE_CALORIE_RESTRICTION_MUSCLE_DN | -0.07426 | -0.02583 | -1.22436 | 0.225178 | 0.59491 | -5.15817 |
| REACTOME_MISMATCH_REPAIR | -0.09724 | -0.01034 | -1.22411 | 0.22527 | 0.59491 | -5.15845 |
| LIU_TARGETS_OF_VMYB_VS_CMYB_DN | 0.062072 | -0.02355 | 1.223974 | 0.225323 | 0.59491 | -5.1586 |
| REACTOME_INTERACTIONS_OF_VPR_WITH_HOST_CELLULAR_PROTEINS | -0.10396 | 0.001592 | -1.22362 | 0.225457 | 0.59491 | -5.159 |
| RUTELLA_RESPONSE_TO_CSF2RB_AND_IL4_DN | 0.064386 | -0.17383 | 1.223568 | 0.225475 | 0.59491 | -5.15906 |
| ZHAN_MULTIPLE_MYELOMA_HP_DN | -0.08441 | -0.09758 | -1.22332 | 0.225569 | 0.594912 | -5.15934 |
| REACTOME_OPIOID_SIGNALLING | -0.04556 | -0.03918 | -1.22256 | 0.225854 | 0.595351 | -5.16018 |
| YAGI_AML_WITH_T_9_11_TRANSLOCATION | -0.06592 | -0.06646 | -1.22231 | 0.225947 | 0.595351 | -5.16046 |
| BURTON_ADIPOGENESIS_11 | -0.10184 | -0.02371 | -1.22209 | 0.22603 | 0.595351 | -5.1607 |
| COULOUARN_TEMPORAL_TGFB1_SIGNATURE_UP | 0.051683 | -0.11843 | 1.221567 | 0.226226 | 0.595351 | -5.16128 |
| GESERICK_TERT_TARGETS_DN | 0.101418 | -0.21959 | 1.221263 | 0.226341 | 0.595351 | -5.16162 |
| IWANAGA_CARCINOGENESIS_BY_KRAS_PTEN_DN | -0.04283 | -0.05939 | -1.22101 | 0.226437 | 0.595351 | -5.16191 |
| REACTOME_AUTOPHAGY | -0.07298 | -0.07069 | -1.22095 | 0.22646 | 0.595351 | -5.16197 |
| EPPERT_LSC_R | -0.07432 | -0.14017 | -1.2208 | 0.226514 | 0.595351 | -5.16213 |
| REACTOME_ECM_PROTEOGLYCANS | 0.070213 | -0.02703 | 1.220633 | 0.226578 | 0.595351 | -5.16232 |
| WP_NUCLEAR_RECEPTORS | 0.053749 | -0.111 | 1.219977 | 0.226824 | 0.59564 | -5.16305 |
| OUILLETTE_CLL_13Q14_DELETION_DN | -0.03476 | -0.17351 | -1.21984 | 0.226875 | 0.59564 | -5.1632 |
| MYLLYKANGAS_AMPLIFICATION_HOT_SPOT_18 | -0.14522 | -0.0326 | -1.21938 | 0.227048 | 0.595848 | -5.16371 |
| KEGG_RENIN_ANGIOTENSIN_SYSTEM | 0.075691 | -0.04458 | 1.218412 | 0.227414 | 0.596564 | -5.16479 |
| PID_RXR_VDR_PATHWAY | 0.036253 | -0.33234 | 1.21799 | 0.227574 | 0.596736 | -5.16525 |
| WP_TRANSLATION_INHIBITORS_IN_CHRONICALLY_ACTIVATED_PDGFRA_CELLS | -0.07172 | -0.05872 | -1.21769 | 0.227687 | 0.596789 | -5.16559 |
| LUI_THYROID_CANCER_CLUSTER_4 | 0.028833 | -0.74971 | 1.217291 | 0.227838 | 0.596936 | -5.16603 |
| WP_SLEEP_REGULATION | 0.053421 | 0.006332 | 1.216255 | 0.228229 | 0.597686 | -5.16717 |
| REACTOME_CS_DS_DEGRADATION | 0.078545 | -0.01496 | 1.216037 | 0.228312 | 0.597686 | -5.16742 |
| REACTOME_RAF_INDEPENDENT_MAPK1_3_ACTIVATION | 0.075947 | -0.11409 | 1.2147 | 0.228818 | 0.598738 | -5.1689 |
| WP_MITOCHONDRIAL_GENE_EXPRESSION | -0.08507 | -0.00523 | -1.21448 | 0.228902 | 0.598738 | -5.16914 |
| REACTOME_POSITIVE_EPIGENETIC_REGULATION_OF_RRNA_EXPRESSION | -0.07895 | -0.11404 | -1.2129 | 0.229503 | 0.600063 | -5.17089 |
| STEINER_ERYTHROCYTE_MEMBRANE_GENES | 0.070558 | -0.00753 | 1.21264 | 0.229599 | 0.60007 | -5.17117 |
| BIOCARTA_PTEN_PATHWAY | -0.07639 | -0.10297 | -1.21181 | 0.229915 | 0.600361 | -5.17209 |
| MAHADEVAN_GIST_MORPHOLOGICAL_SWITCH | 0.091637 | -0.01743 | 1.211609 | 0.229992 | 0.600361 | -5.17231 |
| REACTOME_TRANSCRIPTIONAL_REGULATION_BY_TP53 | -0.05506 | -0.14211 | -1.2116 | 0.229994 | 0.600361 | -5.17232 |
| BIOCARTA_CTL_PATHWAY | 0.083129 | -0.33151 | 1.210934 | 0.230248 | 0.600377 | -5.17305 |
| XU_AKT1_TARGETS_6HR | -0.0781 | -0.07533 | -1.21072 | 0.230329 | 0.600377 | -5.17329 |
| FLECHNER_BIOPSY_KIDNEY_TRANSPLANT_REJECTED_VS_OK_UP | 0.068654 | -0.24734 | 1.21051 | 0.23041 | 0.600377 | -5.17352 |
| KANG_FLUOROURACIL_RESISTANCE_UP | -0.09006 | -0.03855 | -1.21043 | 0.230442 | 0.600377 | -5.17361 |
| REACTOME_PHASE_I_FUNCTIONALIZATION_OF_COMPOUNDS | 0.044941 | -0.06997 | 1.209722 | 0.23071 | 0.600377 | -5.17439 |
| BIOCARTA_PPARG_PATHWAY | -0.13097 | -0.03095 | -1.20932 | 0.230865 | 0.600377 | -5.17484 |
| INGRAM_SHH_TARGETS_UP | 0.040809 | -0.09945 | 1.209057 | 0.230964 | 0.600377 | -5.17512 |
| REACTOME_SIGNALING_BY_HEDGEHOG | -0.04846 | -0.13596 | -1.20865 | 0.23112 | 0.600377 | -5.17557 |
| PARK_TRETINOIN_RESPONSE | 0.1119 | 0.008309 | 1.208621 | 0.23113 | 0.600377 | -5.1756 |
| REACTOME_NOTCH_HLH_TRANSCRIPTION_PATHWAY | -0.06142 | -0.11839 | -1.20836 | 0.231232 | 0.600377 | -5.17589 |
| DUNNE_TARGETS_OF_AML1_MTG8_FUSION_DN | 0.093254 | -0.01362 | 1.208282 | 0.23126 | 0.600377 | -5.17597 |
| WP_GENES_TARGETED_BY_MIRNAS_IN_ADIPOCYTES | -0.07103 | -0.07981 | -1.20827 | 0.231263 | 0.600377 | -5.17598 |
| BANDRES_RESPONSE_TO_CARMUSTIN_WITHOUT_MGMT_24HR_UP | -0.07513 | -0.00421 | -1.20822 | 0.231283 | 0.600377 | -5.17604 |
| MCGOWAN_RSP6_TARGETS_DN | -0.14121 | -0.15878 | -1.20812 | 0.231321 | 0.600377 | -5.17615 |
| HOFFMANN_LARGE_TO_SMALL_PRE_BII_LYMPHOCYTE_UP | -0.05915 | -0.1472 | -1.20734 | 0.231619 | 0.600411 | -5.17701 |
| KEGG_ALZHEIMERS_DISEASE | -0.06071 | -0.1367 | -1.2072 | 0.231672 | 0.600411 | -5.17716 |
| BENPORATH_MYC_MAX_TARGETS | -0.07096 | -0.14157 | -1.20666 | 0.231879 | 0.600411 | -5.17776 |
| WU_HBX_TARGETS_2_UP | 0.062728 | -0.19814 | 1.206292 | 0.232021 | 0.600411 | -5.17816 |
| REACTOME_TRANSCRIPTIONAL_REGULATION_OF_WHITE_ADIPOCYTE_DIFFERENTIATION | -0.06202 | -0.11163 | -1.20621 | 0.232051 | 0.600411 | -5.17825 |
| PID_HIF1A_PATHWAY | 0.096125 | 0.004895 | 1.206096 | 0.232096 | 0.600411 | -5.17838 |
| AKL_HTLV1_INFECTION_UP | -0.09405 | -0.14831 | -1.20606 | 0.23211 | 0.600411 | -5.17842 |
| KONDO_COLON_CANCER_HCP_WITH_H3K27ME1 | 0.053947 | -0.0047 | 1.205173 | 0.232449 | 0.600411 | -5.17939 |
| SCHLOSSER_SERUM_RESPONSE_DN | -0.08027 | -0.07766 | -1.2051 | 0.232478 | 0.600411 | -5.17947 |
| REACTOME_INTERLEUKIN_27_SIGNALING | 0.092773 | -0.17458 | 1.205093 | 0.23248 | 0.600411 | -5.17948 |
| HOFFMANN_SMALL_PRE_BII_TO_IMMATURE_B_LYMPHOCYTE_UP | 0.041786 | -0.34082 | 1.205069 | 0.232489 | 0.600411 | -5.17951 |
| REACTOME_CDT1_ASSOCIATION_WITH_THE_CDC6_ORC_ORIGIN_COMPLEX | -0.0727 | -0.23097 | -1.20497 | 0.232526 | 0.600411 | -5.17961 |
| AGUIRRE_PANCREATIC_CANCER_COPY_NUMBER_UP | -0.04244 | -0.19386 | -1.20477 | 0.232604 | 0.600411 | -5.17983 |
| DELACROIX_RAR_TARGETS_DN | 0.063986 | -0.07854 | 1.204607 | 0.232666 | 0.600411 | -5.18001 |
| ZHAN_MULTIPLE_MYELOMA_PR_DN | -0.06506 | -0.06642 | -1.20439 | 0.232749 | 0.600411 | -5.18025 |
| NUYTTEN_NIPP1_TARGETS_UP | -0.06154 | -0.06663 | -1.20391 | 0.232935 | 0.600466 | -5.18078 |
| LINDGREN_BLADDER_CANCER_CLUSTER_2B | 0.058602 | -0.15731 | 1.203564 | 0.233067 | 0.600466 | -5.18116 |
| REACTOME_PD_1_SIGNALING | 0.037209 | -0.58435 | 1.203304 | 0.233167 | 0.600466 | -5.18144 |
| YAMASHITA_LIVER_CANCER_WITH_EPCAM_UP | -0.08821 | -0.01592 | -1.20268 | 0.233406 | 0.600466 | -5.18212 |
| IVANOVA_HEMATOPOIESIS_STEM_CELL_LONG_TERM | -0.04146 | -0.08463 | -1.20266 | 0.233413 | 0.600466 | -5.18214 |
| ACEVEDO_LIVER_CANCER_UP | -0.09312 | -0.14612 | -1.20261 | 0.233435 | 0.600466 | -5.18221 |
| REACTOME_DOWNSTREAM_SIGNALING_EVENTS_OF_B_CELL_RECEPTOR_BCR | -0.07837 | -0.19288 | -1.20225 | 0.23357 | 0.600466 | -5.18259 |
| HUTTMANN_B_CLL_POOR_SURVIVAL_UP | 0.042214 | -0.13069 | 1.202037 | 0.233654 | 0.600466 | -5.18283 |
| REACTOME_RECEPTOR_TYPE_TYROSINE_PROTEIN_PHOSPHATASES | -0.06579 | -0.06027 | -1.20202 | 0.23366 | 0.600466 | -5.18285 |
| WP_OVERLAP_BETWEEN_SIGNAL_TRANSDUCTION_PATHWAYS_CONTRIBUTING_TO_LMNA_LAMINOPATHIES | 0.062435 | -0.02242 | 1.201818 | 0.233738 | 0.600466 | -5.18307 |
| REACTOME_ACYL_CHAIN_REMODELLING_OF_PE | 0.054644 | -0.04127 | 1.201347 | 0.233919 | 0.600466 | -5.18358 |
| REACTOME_ORC1_REMOVAL_FROM_CHROMATIN | -0.07411 | -0.20035 | -1.20127 | 0.233948 | 0.600466 | -5.18367 |
| SENESE_HDAC2_TARGETS_DN | 0.0574 | -0.04792 | 1.200989 | 0.234057 | 0.600466 | -5.18398 |
| WP_JOUBERT_SYNDROME | -0.05716 | -0.02669 | -1.20016 | 0.234377 | 0.600466 | -5.18488 |
| WP_METHIONINE_METABOLISM_LEADING_TO_SULFUR_AMINO_ACIDS_AND_RELATED_DISORDERS | -0.08701 | 0.001405 | -1.20006 | 0.234415 | 0.600466 | -5.18499 |
| GRYDER_PAX3FOXO1_ENHANCERS_KO_DOWN | -0.06347 | -0.0535 | -1.19971 | 0.234549 | 0.600466 | -5.18537 |
| TSAI_RESPONSE_TO_RADIATION_THERAPY | 0.087766 | -0.16154 | 1.199527 | 0.234621 | 0.600466 | -5.18557 |
| PID_S1P_S1P4_PATHWAY | -0.09709 | 0.004727 | -1.19945 | 0.234651 | 0.600466 | -5.18566 |
| RIZ_ERYTHROID_DIFFERENTIATION_APOBEC2 | 0.069612 | -0.02084 | 1.199442 | 0.234654 | 0.600466 | -5.18567 |
| REACTOME_NOSTRIN_MEDIATED_ENOS_TRAFFICKING | -0.13521 | 0.005781 | -1.19943 | 0.234657 | 0.600466 | -5.18568 |
| LIU_VMYB_TARGETS_UP | -0.06413 | -0.05823 | -1.19877 | 0.234913 | 0.600879 | -5.1864 |
| REACTOME_SIGNALING_BY_RNF43_MUTANTS | -0.09298 | -0.10245 | -1.19774 | 0.235311 | 0.601656 | -5.18752 |
| LANDIS_ERBB2_BREAST_PRENEOPLASTIC_UP | 0.063567 | -0.00071 | 1.1973 | 0.235482 | 0.601778 | -5.18801 |
| WP_DNA_REPLICATION | -0.08288 | -0.01838 | -1.19713 | 0.235548 | 0.601778 | -5.18819 |
| FORTSCHEGGER_PHF8_TARGETS_UP | -0.05613 | -0.03986 | -1.19678 | 0.235682 | 0.601879 | -5.18857 |
| BIOCARTA_MITR_PATHWAY | 0.091702 | -0.01928 | 1.196243 | 0.235891 | 0.60208 | -5.18916 |
| CAFFAREL_RESPONSE_TO_THC_24HR_3_UP | -0.13382 | -0.00728 | -1.19609 | 0.23595 | 0.60208 | -5.18933 |
| WAKABAYASHI_ADIPOGENESIS_PPARG_BOUND_36HR | -0.08283 | -0.0063 | -1.19565 | 0.236119 | 0.602269 | -5.1898 |
| REACTOME_EUKARYOTIC_TRANSLATION_INITIATION | -0.09152 | -0.12098 | -1.19472 | 0.236483 | 0.602758 | -5.19082 |
| REACTOME_LOSS_OF_FUNCTION_OF_MECP2_IN_RETT_SYNDROME | -0.07982 | -0.11565 | -1.19408 | 0.236731 | 0.602758 | -5.19152 |
| GINESTIER_BREAST_CANCER_20Q13_AMPLIFICATION_UP | -0.07302 | -0.03404 | -1.19405 | 0.23674 | 0.602758 | -5.19154 |
| OSWALD_HEMATOPOIETIC_STEM_CELL_IN_COLLAGEN_GEL_UP | 0.05605 | -0.08945 | 1.1931 | 0.237111 | 0.602758 | -5.19258 |
| CHEN_LUNG_CANCER_SURVIVAL | 0.070522 | -0.03396 | 1.193093 | 0.237114 | 0.602758 | -5.19259 |
| REACTOME_VASOPRESSIN_LIKE_RECEPTORS | 0.11684 | 0.022803 | 1.192948 | 0.23717 | 0.602758 | -5.19274 |
| SINGH_NFE2L2_TARGETS | 0.097755 | 0.007705 | 1.192705 | 0.237265 | 0.602758 | -5.19301 |
| TANAKA_METHYLATED_IN_ESOPHAGEAL_CARCINOMA | 0.033907 | -0.07689 | 1.192617 | 0.237299 | 0.602758 | -5.1931 |
| PID_INTEGRIN5_PATHWAY | 0.086855 | 0.00596 | 1.192579 | 0.237314 | 0.602758 | -5.19314 |
| BIOCARTA_EFP_PATHWAY | 0.073072 | -0.13034 | 1.19251 | 0.237341 | 0.602758 | -5.19322 |
| KEGG_ARACHIDONIC_ACID_METABOLISM | 0.047291 | -0.09166 | 1.192398 | 0.237384 | 0.602758 | -5.19334 |
| OSADA_ASCL1_TARGETS_UP | 0.040941 | -0.1501 | 1.192235 | 0.237447 | 0.602758 | -5.19352 |
| LIN_MELANOMA_COPY_NUMBER_DN | -0.0872 | -0.08121 | -1.1915 | 0.237732 | 0.603067 | -5.19431 |
| WP_INTRACELLULAR_TRAFFICKING_PROTEINS_INVOLVED_IN_CMT_NEUROPATHY | 0.061684 | 0.001196 | 1.191436 | 0.237759 | 0.603067 | -5.19439 |
| WP_MICRORNA_NETWORK_ASSOCIATED_WITH_CHRONIC_LYMPHOCYTIC_LEUKEMIA | 0.126881 | 0.010561 | 1.190649 | 0.238065 | 0.603514 | -5.19524 |
| VALK_AML_CLUSTER_16 | 0.056 | -0.00575 | 1.190389 | 0.238167 | 0.603514 | -5.19552 |
| ZHAN_V1_LATE_DIFFERENTIATION_GENES_UP | 0.084883 | -0.18446 | 1.190255 | 0.238219 | 0.603514 | -5.19567 |
| TERAMOTO_OPN_TARGETS_CLUSTER_3 | -0.11203 | -0.01564 | -1.18969 | 0.23844 | 0.603832 | -5.19628 |
| REACTOME_INTRA_GOLGI_TRAFFIC | -0.06107 | -0.05617 | -1.18943 | 0.238541 | 0.603848 | -5.19656 |
| KUROZUMI_RESPONSE_TO_ONCOCYTIC_VIRUS_AND_CYCLIC_RGD | 0.065165 | -0.21214 | 1.189004 | 0.238708 | 0.603888 | -5.19702 |
| PID_ATM_PATHWAY | -0.05381 | -0.24994 | -1.1889 | 0.238747 | 0.603888 | -5.19713 |
| YAO_TEMPORAL_RESPONSE_TO_PROGESTERONE_CLUSTER_2 | 0.041121 | -0.15581 | 1.188177 | 0.239031 | 0.604367 | -5.19792 |
| KYNG_ENVIRONMENTAL_STRESS_RESPONSE_NOT_BY_UV_IN_WS | 0.076032 | -0.06804 | 1.187771 | 0.23919 | 0.604529 | -5.19836 |
| CONCANNON_APOPTOSIS_BY_EPOXOMICIN_UP | 0.062241 | -0.09985 | 1.1871 | 0.239453 | 0.604898 | -5.19909 |
| REACTOME_SYNTHESIS_OF_BILE_ACIDS_AND_BILE_SALTS_VIA_27_HYDROXYCHOLESTEROL | 0.076189 | -0.00672 | 1.186912 | 0.239526 | 0.604898 | -5.19929 |
| REACTOME_ESR_MEDIATED_SIGNALING | -0.06366 | -0.02987 | -1.18504 | 0.240262 | 0.606417 | -5.20131 |
| PEDERSEN_METASTASIS_BY_ERBB2_ISOFORM_6 | 0.066619 | -0.01162 | 1.184784 | 0.240361 | 0.606417 | -5.20159 |
| KRIGE_RESPONSE_TO_TOSEDOSTAT_6HR_DN | -0.05726 | -0.1042 | -1.18465 | 0.240413 | 0.606417 | -5.20173 |
| NADERI_BREAST_CANCER_PROGNOSIS_UP | -0.06475 | -0.03061 | -1.1835 | 0.240867 | 0.607321 | -5.20298 |
| NGUYEN_NOTCH1_TARGETS_DN | 0.04928 | -0.30977 | 1.182707 | 0.241178 | 0.607503 | -5.20383 |
| KEGG_BASE_EXCISION_REPAIR | -0.09921 | -0.00173 | -1.18261 | 0.241215 | 0.607503 | -5.20393 |
| FISCHER_G1_S_CELL_CYCLE | -0.05103 | -0.06282 | -1.18258 | 0.241226 | 0.607503 | -5.20396 |
| REACTOME_TRAFFICKING_AND_PROCESSING_OF_ENDOSOMAL_TLR | 0.104153 | -0.04867 | 1.182107 | 0.241414 | 0.607737 | -5.20448 |
| LI_ADIPOGENESIS_BY_ACTIVATED_PPARG | 0.064525 | -0.15301 | 1.181464 | 0.241667 | 0.60796 | -5.20517 |
| WP_GLUCURONIDATION | 0.069536 | -0.08013 | 1.181366 | 0.241706 | 0.60796 | -5.20527 |
| MARKS_HDAC_TARGETS_UP | 0.059035 | -0.0081 | 1.181154 | 0.241789 | 0.60796 | -5.2055 |
| REACTOME_TRANSCRIPTIONAL_REGULATION_BY_MECP2 | 0.03599 | -0.07006 | 1.180693 | 0.241971 | 0.608177 | -5.206 |
| NAKAMURA_ALVEOLAR_EPITHELIUM | -0.10729 | -0.15504 | -1.18013 | 0.242193 | 0.608495 | -5.2066 |
| BIOCARTA_S1P_PATHWAY | -0.10823 | 0.014106 | -1.1789 | 0.242682 | 0.609385 | -5.20793 |
| BRUINS_UVC_RESPONSE_LATE | -0.0542 | -0.08902 | -1.17875 | 0.242739 | 0.609385 | -5.20809 |
| REACTOME_SHC1_EVENTS_IN_EGFR_SIGNALING | 0.067309 | -0.06957 | 1.178442 | 0.242861 | 0.609451 | -5.20842 |
| BIOCARTA_MELANOCYTE_PATHWAY | -0.10178 | -0.11217 | -1.17795 | 0.243056 | 0.609701 | -5.20895 |
| ZHOU_CELL_CYCLE_GENES_IN_IR_RESPONSE_2HR | -0.09047 | -0.00559 | -1.17747 | 0.243244 | 0.609762 | -5.20946 |
| GNATENKO_PLATELET_SIGNATURE | 0.059147 | -0.2563 | 1.177113 | 0.243387 | 0.609762 | -5.20985 |
| REACTOME_ZINC_INFLUX_INTO_CELLS_BY_THE_SLC39_GENE_FAMILY | 0.067316 | -0.29077 | 1.176657 | 0.243568 | 0.609762 | -5.21033 |
| REACTOME_INFLUENZA_INFECTION | -0.08577 | -0.12028 | -1.17642 | 0.243663 | 0.609762 | -5.21059 |
| SMID_BREAST_CANCER_LUMINAL_A_DN | 0.086009 | -0.02045 | 1.17636 | 0.243686 | 0.609762 | -5.21065 |
| WP_GLYCOSYLATION_AND_RELATED_CONGENITAL_DEFECTS | -0.09594 | 0.002114 | -1.17628 | 0.243718 | 0.609762 | -5.21074 |
| GROSS_HYPOXIA_VIA_ELK3_ONLY_DN | -0.07814 | -0.02603 | -1.17619 | 0.243753 | 0.609762 | -5.21084 |
| KIM_MYC_AMPLIFICATION_TARGETS_UP | -0.06975 | -0.06389 | -1.17595 | 0.243847 | 0.609762 | -5.21109 |
| ZHOU_PANCREATIC_EXOCRINE_PROGENITOR | 0.088747 | -0.06864 | 1.174501 | 0.244424 | 0.61018 | -5.21265 |
| BLANCO_MELO_HUMAN_PARAINFLUENZA_VIRUS_3_INFECTION_A594_CELLS_DN | 0.044042 | -0.23935 | 1.174424 | 0.244455 | 0.61018 | -5.21273 |
| WAMUNYOKOLI_OVARIAN_CANCER_LMP_DN | -0.08201 | -0.04581 | -1.17436 | 0.244479 | 0.61018 | -5.21279 |
| WP_NRF2ARE_REGULATION | 0.071996 | -0.00301 | 1.174098 | 0.244584 | 0.61018 | -5.21308 |
| BIOCARTA_CK1_PATHWAY | -0.06417 | -0.06045 | -1.17392 | 0.244653 | 0.61018 | -5.21326 |
| BIOCARTA_ARAP_PATHWAY | 0.095302 | 0.009996 | 1.1739 | 0.244663 | 0.61018 | -5.21329 |
| PID_GMCSF_PATHWAY | 0.079944 | -0.042 | 1.173678 | 0.244751 | 0.61018 | -5.21353 |
| KEGG_FATTY_ACID_METABOLISM | -0.08041 | -0.00684 | -1.17343 | 0.244851 | 0.61018 | -5.21379 |
| PYEON_HPV_POSITIVE_TUMORS_DN | 0.072196 | -0.16976 | 1.173285 | 0.244907 | 0.61018 | -5.21395 |
| REACTOME_OLFACTORY_SIGNALING_PATHWAY | 0.050166 | -0.39423 | 1.172893 | 0.245064 | 0.61018 | -5.21437 |
| REACTOME_NEUTROPHIL_DEGRANULATION | 0.055385 | -0.1689 | 1.172675 | 0.24515 | 0.61018 | -5.2146 |
| HOLLEMAN_ASPARAGINASE_RESISTANCE_ALL_DN | -0.09991 | -0.04556 | -1.17184 | 0.245483 | 0.61018 | -5.21549 |
| KAAB_HEART_ATRIUM_VS_VENTRICLE_DN | -0.0631 | -0.08304 | -1.17184 | 0.245483 | 0.61018 | -5.21549 |
| BIOCARTA_MALATEX_PATHWAY | -0.12524 | 0.009492 | -1.17174 | 0.245524 | 0.61018 | -5.2156 |
| ZHAN_MULTIPLE_MYELOMA_LB_UP | -0.04054 | -0.02294 | -1.17129 | 0.245703 | 0.61018 | -5.21608 |
| WP_RAS_AND_BRADYKININ_PATHWAYS_IN_COVID19 | 0.049929 | -0.21275 | 1.171287 | 0.245704 | 0.61018 | -5.21608 |
| FARDIN_HYPOXIA_11 | 0.09115 | -0.0691 | 1.171015 | 0.245812 | 0.61018 | -5.21637 |
| MIKKELSEN_MEF_ICP_WITH_H3K27ME3 | 0.051023 | -0.20842 | 1.171012 | 0.245814 | 0.61018 | -5.21638 |
| BHATI_G2M_ARREST_BY_2METHOXYESTRADIOL_DN | -0.04947 | -0.15324 | -1.17096 | 0.245836 | 0.61018 | -5.21644 |
| BERTUCCI_MEDULLARY_VS_DUCTAL_BREAST_CANCER_UP | 0.053503 | -0.21085 | 1.170471 | 0.246029 | 0.610316 | -5.21695 |
| MACAEVA_PBMC_RESPONSE_TO_IR | -0.05523 | -0.08008 | -1.17027 | 0.246111 | 0.610316 | -5.21717 |
| REACTOME_RNA_POLYMERASE_II_TRANSCRIPTION_TERMINATION | -0.08196 | -0.09819 | -1.16963 | 0.246365 | 0.610316 | -5.21785 |
| SENESE_HDAC3_TARGETS_DN | -0.04336 | -0.08733 | -1.16959 | 0.24638 | 0.610316 | -5.21789 |
| WEBER_METHYLATED_LCP_IN_SPERM_UP | 0.110322 | -0.00193 | 1.169438 | 0.246442 | 0.610316 | -5.21806 |
| WP_IL17_SIGNALING_PATHWAY | 0.058912 | -0.05917 | 1.169165 | 0.246552 | 0.610316 | -5.21835 |
| CHIARETTI_T_ALL_REFRACTORY_TO_THERAPY | 0.054791 | -0.03094 | 1.169039 | 0.246602 | 0.610316 | -5.21848 |
| GOUYER_TATI_TARGETS_UP | 0.079364 | -0.36768 | 1.168558 | 0.246794 | 0.610316 | -5.21899 |
| TAKEDA_TARGETS_OF_NUP98_HOXA9_FUSION_16D_UP | 0.03615 | -0.17602 | 1.168477 | 0.246827 | 0.610316 | -5.21908 |
| REACTOME_TRIGLYCERIDE_CATABOLISM | -0.0535 | -0.04058 | -1.16842 | 0.24685 | 0.610316 | -5.21914 |
| LIM_MAMMARY_STEM_CELL_DN | -0.04222 | -0.13033 | -1.16801 | 0.247015 | 0.610357 | -5.21958 |
| LEIN_OLIGODENDROCYTE_MARKERS | 0.044541 | -0.11741 | 1.167899 | 0.247058 | 0.610357 | -5.2197 |
| SAKAI_CHRONIC_HEPATITIS_VS_LIVER_CANCER_UP | -0.07077 | -0.26986 | -1.16627 | 0.247712 | 0.61157 | -5.22143 |
| WP_DNA_MISMATCH_REPAIR | -0.09496 | -0.0132 | -1.16606 | 0.247797 | 0.61157 | -5.22166 |
| BORCZUK_MALIGNANT_MESOTHELIOMA_UP | -0.09775 | -0.02484 | -1.16596 | 0.247837 | 0.61157 | -5.22176 |
| RUAN_RESPONSE_TO_TROGLITAZONE_UP | 0.071121 | -0.23168 | 1.165329 | 0.248089 | 0.611612 | -5.22243 |
| REACTOME_ERYTHROPOIETIN_ACTIVATES_STAT5 | 0.103856 | -0.02223 | 1.165065 | 0.248196 | 0.611612 | -5.22271 |
| REACTOME_CYTOSOLIC_TRNA_AMINOACYLATION | -0.09831 | 0.003832 | -1.16478 | 0.248311 | 0.611612 | -5.22302 |
| WP_INHIBITION_OF_EXOSOME_BIOGENESIS_AND_SECRETION_BY_MANUMYCIN_A_IN_CRPC_CELLS | -0.09783 | -0.11265 | -1.16473 | 0.248329 | 0.611612 | -5.22306 |
| MULLIGHAN_NPM1_SIGNATURE_3_UP | -0.04014 | -0.09146 | -1.16472 | 0.248335 | 0.611612 | -5.22308 |
| WANG_BARRETTS_ESOPHAGUS_AND_ESOPHAGUS_CANCER_DN | 0.050109 | -0.15473 | 1.16444 | 0.248447 | 0.611649 | -5.22337 |
| WP_RETINOBLASTOMA_GENE_IN_CANCER | -0.08756 | -0.02842 | -1.16381 | 0.248698 | 0.611938 | -5.22404 |
| REACTOME_MRNA_EDITING_C_TO_U_CONVERSION | 0.094381 | -0.09354 | 1.163671 | 0.248756 | 0.611938 | -5.22419 |
| REACTOME_ACTIVATION_OF_THE_MRNA_UPON_BINDING_OF_THE_CAP_BINDING_COMPLEX_AND_EIFS_AND_SUBSEQUENT_BINDING_TO_43S | -0.08421 | -0.20473 | -1.16337 | 0.248878 | 0.612001 | -5.22451 |
| REACTOME_G2_M_CHECKPOINTS | -0.05914 | -0.18908 | -1.16291 | 0.249064 | 0.612222 | -5.225 |
| KEGG_GLIOMA | -0.05589 | -0.05321 | -1.16207 | 0.249401 | 0.612543 | -5.22589 |
| WP_HIPPO_SIGNALING_REGULATION_PATHWAYS | -0.03461 | -0.02663 | -1.16192 | 0.249461 | 0.612543 | -5.22605 |
| TURASHVILI_BREAST_LOBULAR_CARCINOMA_VS_LOBULAR_NORMAL_UP | -0.05698 | -0.06151 | -1.16153 | 0.249617 | 0.612543 | -5.22646 |
| PUJANA_XPRSS_INT_NETWORK | -0.07479 | -0.15183 | -1.16142 | 0.249662 | 0.612543 | -5.22658 |
| BILANGES_RAPAMYCIN_SENSITIVE_VIA_TSC1_AND_TSC2 | -0.06699 | -0.20718 | -1.16118 | 0.249759 | 0.612543 | -5.22683 |
| REACTOME_SIGNALING_BY_NOTCH1_T_7_9_NOTCH1_M1580_K2555_TRANSLOCATION_MUTANT | -0.09839 | -0.10937 | -1.16112 | 0.249785 | 0.612543 | -5.2269 |
| ZIRN_TRETINOIN_RESPONSE_WT1_UP | -0.08404 | -0.04369 | -1.16091 | 0.249868 | 0.612543 | -5.22712 |
| REACTOME_DEADENYLATION_DEPENDENT_MRNA_DECAY | -0.06309 | -0.28827 | -1.15998 | 0.250246 | 0.612893 | -5.22811 |
| GOBERT_OLIGODENDROCYTE_DIFFERENTIATION_UP | -0.05128 | -0.1125 | -1.15969 | 0.250361 | 0.612893 | -5.22841 |
| REACTOME_METABOLISM_OF_NUCLEOTIDES | -0.05584 | -0.03426 | -1.15968 | 0.250365 | 0.612893 | -5.22842 |
| WP_PATHWAYS_OF_NUCLEIC_ACID_METABOLISM_AND_INNATE_IMMUNE_SENSING | 0.079823 | -0.00098 | 1.159606 | 0.250396 | 0.612893 | -5.2285 |
| BOYLAN_MULTIPLE_MYELOMA_PCA3_UP | 0.055305 | -0.00646 | 1.158441 | 0.250868 | 0.61318 | -5.22973 |
| REACTOME_DOPAMINE_RECEPTORS | 0.115452 | -0.15445 | 1.158368 | 0.250897 | 0.61318 | -5.22981 |
| BIOCARTA_RHODOPSIN_PATHWAY | 0.084324 | -0.2012 | 1.158194 | 0.250968 | 0.61318 | -5.22999 |
| REACTOME_RET_SIGNALING | -0.04487 | -0.02444 | -1.15786 | 0.251102 | 0.61318 | -5.23034 |
| ONO_FOXP3_TARGETS_DN | 0.069351 | -0.02785 | 1.157834 | 0.251114 | 0.61318 | -5.23037 |
| SCHAEFFER_SOX9_TARGETS_IN_PROSTATE_DEVELOPMENT_UP | -0.07164 | 0.001324 | -1.15769 | 0.251173 | 0.61318 | -5.23053 |
| KEGG_SPLICEOSOME | -0.06441 | -0.26627 | -1.15765 | 0.251188 | 0.61318 | -5.23057 |
| ZHANG_RESPONSE_TO_CANTHARIDIN_DN | -0.08529 | -0.09891 | -1.15705 | 0.251432 | 0.61336 | -5.2312 |
| WP_NITRIC_OXIDE_METABOLISM_IN_CYSTIC_FIBROSIS | -0.05988 | -0.29757 | -1.15676 | 0.251551 | 0.61336 | -5.23151 |
| REACTOME_DECTIN_1_MEDIATED_NONCANONICAL_NF_KB_SIGNALING | -0.0729 | -0.21964 | -1.15665 | 0.251592 | 0.61336 | -5.23162 |
| REACTOME_NONHOMOLOGOUS_END_JOINING_NHEJ | -0.05404 | -0.19329 | -1.15652 | 0.251647 | 0.61336 | -5.23176 |
| PIEPOLI_LGI1_TARGETS_DN | -0.06582 | -0.07963 | -1.156 | 0.251858 | 0.613638 | -5.23231 |
| LEE_METASTASIS_AND_ALTERNATIVE_SPLICING_UP | 0.052256 | -0.099 | 1.154621 | 0.252418 | 0.614554 | -5.23376 |
| CHEOK_RESPONSE_TO_MERCAPTOPURINE_AND_LD_MTX_DN | 0.052359 | -0.13746 | 1.1546 | 0.252427 | 0.614554 | -5.23378 |
| BIOCARTA_G2_PATHWAY | 0.058626 | -0.08057 | 1.153984 | 0.252677 | 0.6147 | -5.23443 |
| PID_CDC42_REG_PATHWAY | 0.052121 | -0.06338 | 1.153717 | 0.252786 | 0.6147 | -5.23471 |
| CAFFAREL_RESPONSE_TO_THC_8HR_3_DN | -0.11768 | 0.015214 | -1.15359 | 0.252838 | 0.6147 | -5.23485 |
| REACTOME_REGULATION_OF_MRNA_STABILITY_BY_PROTEINS_THAT_BIND_AU_RICH_ELEMENTS | -0.0759 | -0.21128 | -1.1535 | 0.252873 | 0.6147 | -5.23494 |
| KYNG_DNA_DAMAGE_BY_UV | 0.049755 | -0.01804 | 1.152893 | 0.253122 | 0.61507 | -5.23558 |
| TONKS_TARGETS_OF_RUNX1_RUNX1T1_FUSION_SUSTAINED_IN_GRANULOCYTE_UP | 0.080303 | 0.008334 | 1.15254 | 0.253266 | 0.615184 | -5.23595 |
| BIOCARTA_CDC42RAC_PATHWAY | -0.13153 | -0.0157 | -1.15129 | 0.253775 | 0.616187 | -5.23726 |
| WP_CILIARY_LANDSCAPE | -0.06978 | -0.02048 | -1.14953 | 0.254495 | 0.617639 | -5.23911 |
| BIOCARTA_EEA1_PATHWAY | 0.097643 | -0.00606 | 1.149354 | 0.254567 | 0.617639 | -5.2393 |
| REACTOME_HEME_SIGNALING | -0.06695 | -0.01295 | -1.14904 | 0.254696 | 0.617715 | -5.23963 |
| ELVIDGE_HIF1A_TARGETS_UP | -0.08673 | -0.01392 | -1.14858 | 0.254883 | 0.617934 | -5.24011 |
| HUI_MAPK14_TARGETS_UP | -0.04847 | -0.166 | -1.14781 | 0.255199 | 0.618463 | -5.24091 |
| AGUIRRE_PANCREATIC_CANCER_COPY_NUMBER_DN | -0.0463 | -0.22625 | -1.14724 | 0.255435 | 0.618801 | -5.24152 |
| YU_MYC_TARGETS_DN | 0.036233 | -0.45068 | 1.146551 | 0.255716 | 0.619245 | -5.24223 |
| SWEET_LUNG_CANCER_KRAS_UP | 0.049818 | -0.14575 | 1.145909 | 0.25598 | 0.619648 | -5.24291 |
| JOHANSSON_GLIOMAGENESIS_BY_PDGFB_UP | 0.054303 | -0.20221 | 1.145456 | 0.256166 | 0.619862 | -5.24338 |
| MANNE_COVID19_COMBINED_COHORT_VS_HEALTHY_DONOR_PLATELETS_UP | 0.045374 | -0.00475 | 1.144375 | 0.25661 | 0.620639 | -5.24451 |
| BIOCARTA_RACC_PATHWAY | 0.093849 | -0.05267 | 1.144093 | 0.256726 | 0.620639 | -5.2448 |
| GARGALOVIC_RESPONSE_TO_OXIDIZED_PHOSPHOLIPIDS_GREY_DN | -0.06541 | -0.00661 | -1.14384 | 0.256829 | 0.620639 | -5.24507 |
| CAFFAREL_RESPONSE_TO_THC_24HR_5_DN | -0.08308 | 0.004812 | -1.14373 | 0.256877 | 0.620639 | -5.24519 |
| WOO_LIVER_CANCER_RECURRENCE_UP | 0.066344 | -0.07831 | 1.142324 | 0.257456 | 0.620903 | -5.24665 |
| KEGG_FRUCTOSE_AND_MANNOSE_METABOLISM | 0.06219 | 0.001695 | 1.142262 | 0.257481 | 0.620903 | -5.24671 |
| JI_METASTASIS_REPRESSED_BY_STK11 | 0.05317 | -0.03156 | 1.141687 | 0.257718 | 0.620903 | -5.24731 |
| REACTOME_THROMBOXANE_SIGNALLING_THROUGH_TP_RECEPTOR | 0.05031 | -0.04358 | 1.141167 | 0.257933 | 0.620903 | -5.24786 |
| ZHAN_MULTIPLE_MYELOMA_MS_UP | -0.06762 | -0.0693 | -1.14095 | 0.258024 | 0.620903 | -5.24809 |
| WEST_ADRENOCORTICAL_TUMOR_MARKERS_DN | 0.070937 | -0.10055 | 1.140767 | 0.258099 | 0.620903 | -5.24827 |
| GENTILE_UV_RESPONSE_CLUSTER_D8 | -0.09303 | 0.002473 | -1.14067 | 0.258138 | 0.620903 | -5.24837 |
| BRUINS_UVC_RESPONSE_VIA_TP53_GROUP_C | 0.035696 | -0.10958 | 1.140629 | 0.258156 | 0.620903 | -5.24842 |
| HOFFMANN_PRE_BI_TO_LARGE_PRE_BII_LYMPHOCYTE_DN | 0.038145 | -0.18398 | 1.140543 | 0.258191 | 0.620903 | -5.24851 |
| REACTOME_TRANSCRIPTIONAL_REGULATION_OF_TESTIS_DIFFERENTIATION | 0.096332 | -0.05336 | 1.140456 | 0.258227 | 0.620903 | -5.2486 |
| PID_ERBB2_ERBB3_PATHWAY | -0.0615 | -0.05703 | -1.14027 | 0.258304 | 0.620903 | -5.24879 |
| PID_PLK1_PATHWAY | -0.07879 | -0.01146 | -1.14023 | 0.258321 | 0.620903 | -5.24883 |
| GAVIN_FOXP3_TARGETS_CLUSTER_P3 | 0.034463 | -0.02664 | 1.140163 | 0.258348 | 0.620903 | -5.2489 |
| AMIT_EGF_RESPONSE_60_MCF10A | 0.069879 | -0.18001 | 1.140154 | 0.258352 | 0.620903 | -5.24891 |
| BRUINS_UVC_RESPONSE_MIDDLE | -0.0398 | -0.10977 | -1.13959 | 0.258585 | 0.621228 | -5.2495 |
| REACTOME_MET_ACTIVATES_PTK2_SIGNALING | 0.078351 | -0.12046 | 1.138165 | 0.259175 | 0.622369 | -5.25098 |
| REACTOME_TOLL_LIKE_RECEPTOR_CASCADES | 0.055921 | -0.0718 | 1.137603 | 0.259408 | 0.622369 | -5.25156 |
| RODRIGUES_THYROID_CARCINOMA_POORLY_DIFFERENTIATED_DN | -0.05382 | -0.09573 | -1.13756 | 0.259428 | 0.622369 | -5.25161 |
| PID_ARF6_DOWNSTREAM_PATHWAY | 0.08024 | -0.0713 | 1.137501 | 0.259451 | 0.622369 | -5.25167 |
| REACTOME_INSERTION_OF_TAIL_ANCHORED_PROTEINS_INTO_THE_ENDOPLASMIC_RETICULUM_MEMBRANE | -0.09505 | -0.18215 | -1.13689 | 0.259706 | 0.622516 | -5.25231 |
| LI_WILMS_TUMOR_ANAPLASTIC_UP | -0.08258 | -0.01183 | -1.13603 | 0.26006 | 0.622516 | -5.25319 |
| WP_RELATIONSHIP_BETWEEN_INFLAMMATION_COX2_AND_EGFR | -0.059 | -0.10315 | -1.13596 | 0.260091 | 0.622516 | -5.25327 |
| REACTOME_AMPK_INHIBITS_CHREBP_TRANSCRIPTIONAL_ACTIVATION_ACTIVITY | 0.076199 | -0.12794 | 1.13593 | 0.260103 | 0.622516 | -5.2533 |
| REACTOME_REGULATION_OF_GLYCOLYSIS_BY_FRUCTOSE_2_6_BISPHOSPHATE_METABOLISM | 0.059538 | -0.08484 | 1.135858 | 0.260133 | 0.622516 | -5.25337 |
| RODWELL_AGING_KIDNEY_UP | 0.049068 | -0.26069 | 1.135731 | 0.260185 | 0.622516 | -5.25351 |
| WP_TAMOXIFEN_METABOLISM | 0.066621 | -0.2031 | 1.135704 | 0.260197 | 0.622516 | -5.25353 |
| PUJANA_BRCA_CENTERED_NETWORK | -0.07401 | -0.16752 | -1.13471 | 0.260608 | 0.622887 | -5.25456 |
| KIM_MYCN_AMPLIFICATION_TARGETS_DN | -0.06076 | -0.009 | -1.13466 | 0.260631 | 0.622887 | -5.25462 |
| MILICIC_FAMILIAL_ADENOMATOUS_POLYPOSIS_DN | 0.10487 | -0.00974 | 1.134517 | 0.260691 | 0.622887 | -5.25476 |
| KEGG_LIMONENE_AND_PINENE_DEGRADATION | -0.12667 | 0.003846 | -1.13439 | 0.260743 | 0.622887 | -5.2549 |
| BREDEMEYER_RAG_SIGNALING_VIA_ATM_NOT_VIA_NFKB_DN | -0.07418 | -0.01628 | -1.13355 | 0.261094 | 0.623346 | -5.25577 |
| REACTOME_NOD1_2_SIGNALING_PATHWAY | 0.074759 | -0.00382 | 1.133458 | 0.261132 | 0.623346 | -5.25586 |
| BIOCARTA_TCRA_PATHWAY | 0.04458 | -0.54181 | 1.132292 | 0.261617 | 0.623831 | -5.25707 |
| REACTOME_CELLULAR_RESPONSES_TO_STIMULI | -0.06272 | -0.10797 | -1.13224 | 0.261641 | 0.623831 | -5.25712 |
| KIM_MYC_AMPLIFICATION_TARGETS_DN | -0.03812 | -0.14257 | -1.13184 | 0.261807 | 0.623831 | -5.25754 |
| WP_HEDGEHOG_SIGNALING_PATHWAY_WP4249 | -0.05347 | -0.04476 | -1.13181 | 0.261821 | 0.623831 | -5.25757 |
| WP_SULINDAC_METABOLIC_PATHWAY | -0.11334 | -0.17255 | -1.1317 | 0.261866 | 0.623831 | -5.25768 |
| JIANG_HYPOXIA_VIA_VHL | -0.0927 | 0.001565 | -1.13148 | 0.261957 | 0.623831 | -5.25791 |
| MEISSNER_BRAIN_ICP_WITH_H3K4ME3 | -0.03801 | -0.34301 | -1.13133 | 0.262021 | 0.623831 | -5.25807 |
| MULLIGHAN_NPM1_MUTATED_SIGNATURE_1_UP | -0.03737 | -0.08311 | -1.13098 | 0.262167 | 0.623942 | -5.25843 |
| GRAESSMANN_RESPONSE_TO_MC_AND_SERUM_DEPRIVATION_DN | 0.045042 | -0.0382 | 1.130697 | 0.262284 | 0.623942 | -5.25872 |
| DITTMER_PTHLH_TARGETS_UP | -0.07565 | -0.09958 | -1.13034 | 0.262433 | 0.623942 | -5.25909 |
| EHRLICH_ICF_SYNDROM_UP | 0.100061 | -0.01726 | 1.130174 | 0.262502 | 0.623942 | -5.25926 |
| LEIN_CEREBELLUM_MARKERS | 0.040356 | -0.06262 | 1.130042 | 0.262558 | 0.623942 | -5.25939 |
| MARTENS_TRETINOIN_RESPONSE_DN | -0.05403 | -0.09765 | -1.12954 | 0.262767 | 0.624045 | -5.25991 |
| SERVITJA_LIVER_HNF1A_TARGETS_DN | 0.035458 | -0.14103 | 1.129425 | 0.262816 | 0.624045 | -5.26003 |
| HOLLERN_MICROACINAR_BREAST_TUMOR_UP | 0.039737 | -0.18794 | 1.129235 | 0.262895 | 0.624045 | -5.26022 |
| REACTOME_DEFECTIVE_C1GALT1C1_CAUSES_TNPS | 0.071422 | -0.1623 | 1.128723 | 0.263109 | 0.624217 | -5.26075 |
| REACTOME_ERYTHROPOIETIN_ACTIVATES_PHOSPHOLIPASE_C_GAMMA_PLCG | 0.107657 | -0.02102 | 1.128144 | 0.263352 | 0.624217 | -5.26135 |
| REACTOME_GLUCAGON_LIKE_PEPTIDE_1_GLP1_REGULATES_INSULIN_SECRETION | -0.03772 | -0.06421 | -1.12812 | 0.263361 | 0.624217 | -5.26137 |
| REACTOME_STABILIZATION_OF_P53 | -0.07151 | -0.24031 | -1.12801 | 0.263408 | 0.624217 | -5.26149 |
| PID_IL12_2PATHWAY | 0.055692 | -0.21818 | 1.12771 | 0.263534 | 0.624217 | -5.2618 |
| WAMUNYOKOLI_OVARIAN_CANCER_LMP_UP | -0.04877 | -0.09331 | -1.12766 | 0.263556 | 0.624217 | -5.26185 |
| FARMER_BREAST_CANCER_APOCRINE_VS_BASAL | -0.04947 | -0.05778 | -1.12684 | 0.2639 | 0.624591 | -5.26269 |
| REACTOME_FERTILIZATION | 0.072294 | -0.12518 | 1.126728 | 0.263946 | 0.624591 | -5.26281 |
| PID_INTEGRIN_A4B1_PATHWAY | 0.059834 | -0.05442 | 1.12658 | 0.264008 | 0.624591 | -5.26296 |
| REACTOME_RUNX1_REGULATES_TRANSCRIPTION_OF_GENES_INVOLVED_IN_DIFFERENTIATION_OF_HSCS | -0.06684 | -0.21129 | -1.12619 | 0.264173 | 0.624631 | -5.26336 |
| PID_HNF3A_PATHWAY | 0.050909 | -0.03173 | 1.126072 | 0.264222 | 0.624631 | -5.26348 |
| BIOCARTA_LECTIN_PATHWAY | -0.03541 | -0.53936 | -1.1248 | 0.264755 | 0.625523 | -5.26479 |
| REACTOME_SCF_SKP2_MEDIATED_DEGRADATION_OF_P27_P21 | -0.07185 | -0.22678 | -1.12471 | 0.264796 | 0.625523 | -5.26488 |
| KEGG_TERPENOID_BACKBONE_BIOSYNTHESIS | -0.09097 | 0.003907 | -1.12436 | 0.264943 | 0.62564 | -5.26524 |
| REACTOME_ACYL_CHAIN_REMODELLING_OF_PS | 0.059938 | -0.05519 | 1.123711 | 0.265215 | 0.626049 | -5.26591 |
| REACTOME_CHL1_INTERACTIONS | -0.0886 | -0.01184 | -1.12328 | 0.265397 | 0.626143 | -5.26635 |
| REACTOME_DEFECTIVE_B4GALT1_CAUSES_B4GALT1_CDG_CDG_2D | 0.09751 | -0.0039 | 1.122995 | 0.265517 | 0.626143 | -5.26664 |
| REACTOME_G_ALPHA_S_SIGNALLING_EVENTS | 0.049224 | -0.03453 | 1.122917 | 0.26555 | 0.626143 | -5.26672 |
| SMID_BREAST_CANCER_ERBB2_DN | 0.104582 | 0.017413 | 1.122105 | 0.265892 | 0.626496 | -5.26755 |
| REACTOME_DEUBIQUITINATION | -0.05795 | -0.11663 | -1.1221 | 0.265897 | 0.626496 | -5.26757 |
| BIOCARTA_AKAPCENTROSOME_PATHWAY | -0.05961 | -0.06698 | -1.12169 | 0.266066 | 0.626663 | -5.26798 |
| LEE_DOUBLE_POLAR_THYMOCYTE | 0.052163 | -0.04008 | 1.120968 | 0.266372 | 0.627033 | -5.26872 |
| WEST_ADRENOCORTICAL_TUMOR_UP | -0.07127 | -0.07831 | -1.12059 | 0.266533 | 0.627033 | -5.26911 |
| YAO_TEMPORAL_RESPONSE_TO_PROGESTERONE_CLUSTER_14 | -0.07052 | -0.16282 | -1.12034 | 0.26664 | 0.627033 | -5.26937 |
| BROWNE_HCMV_INFECTION_1HR_UP | 0.046127 | -0.01379 | 1.119781 | 0.266874 | 0.627033 | -5.26993 |
| BIOCARTA_AT1R_PATHWAY | -0.09028 | -0.03492 | -1.11966 | 0.266925 | 0.627033 | -5.27006 |
| REACTOME_REGULATION_OF_SIGNALING_BY_CBL | 0.096393 | 0.014713 | 1.119635 | 0.266936 | 0.627033 | -5.27008 |
| REACTOME_LOSS_OF_MECP2_BINDING_ABILITY_TO_THE_NCOR_SMRT_COMPLEX | -0.10384 | -0.11871 | -1.11956 | 0.266969 | 0.627033 | -5.27016 |
| GEORGES_CELL_CYCLE_MIR192_TARGETS | -0.06882 | -0.04805 | -1.11897 | 0.267216 | 0.627033 | -5.27076 |
| XU_GH1_EXOGENOUS_TARGETS_DN | 0.028399 | -0.14288 | 1.118476 | 0.267427 | 0.627033 | -5.27127 |
| PID_ECADHERIN_NASCENT_AJ_PATHWAY | -0.06935 | -0.17902 | -1.1182 | 0.267543 | 0.627033 | -5.27155 |
| XU_HGF_SIGNALING_NOT_VIA_AKT1_48HR_DN | -0.07163 | -0.01327 | -1.11807 | 0.267599 | 0.627033 | -5.27168 |
| REACTOME_APOPTOTIC_CLEAVAGE_OF_CELLULAR_PROTEINS | 0.036525 | -0.07591 | 1.117422 | 0.267874 | 0.627033 | -5.27235 |
| HOOI_ST7_TARGETS_UP | -0.05039 | -0.04612 | -1.11736 | 0.267901 | 0.627033 | -5.27241 |
| REACTOME_SIGNALING_BY_PTK6 | 0.053089 | -0.02313 | 1.117345 | 0.267907 | 0.627033 | -5.27242 |
| ROSS_AML_WITH_CBFB_MYH11_FUSION | 0.074938 | -0.0523 | 1.117081 | 0.268018 | 0.627033 | -5.27269 |
| BIOCARTA_EGF_PATHWAY | -0.08822 | -0.02943 | -1.11682 | 0.26813 | 0.627033 | -5.27296 |
| REACTOME_GLYCOGEN_METABOLISM | -0.07969 | ####### | -1.11678 | 0.268146 | 0.627033 | -5.273 |
| MALONEY_RESPONSE_TO_17AAG_UP | 0.060116 | -0.11362 | 1.116656 | 0.268199 | 0.627033 | -5.27313 |
| GRAHAM_CML_QUIESCENT_VS_NORMAL_QUIESCENT_DN | 0.029128 | -0.50752 | 1.116367 | 0.268322 | 0.627033 | -5.27342 |
| RAY_TUMORIGENESIS_BY_ERBB2_CDC25A_DN | -0.04598 | -0.09461 | -1.11612 | 0.268427 | 0.627033 | -5.27367 |
| BLANCO_MELO_INFLUENZA_A_INFECTION_A594_CELLS_UP | 0.07386 | -0.02429 | 1.116065 | 0.26845 | 0.627033 | -5.27373 |
| REACTOME_CHOLESTEROL_BIOSYNTHESIS | -0.1037 | 0.005418 | -1.11594 | 0.268503 | 0.627033 | -5.27386 |
| WP_MRNA_PROCESSING | -0.07211 | -0.1744 | -1.11585 | 0.268542 | 0.627033 | -5.27395 |
| CADWELL_ATG16L1_TARGETS_DN | -0.03993 | -0.15402 | -1.11495 | 0.268926 | 0.627033 | -5.27487 |
| BERENJENO_ROCK_SIGNALING_NOT_VIA_RHOA_UP | 0.035333 | -0.40439 | 1.114579 | 0.269082 | 0.627033 | -5.27524 |
| NAKAMURA_LUNG_CANCER | 0.082636 | -0.18584 | 1.114535 | 0.2691 | 0.627033 | -5.27529 |
| WP_CHEMOKINE_SIGNALING_PATHWAY | 0.043369 | -0.06883 | 1.114522 | 0.269106 | 0.627033 | -5.2753 |
| REACTOME_ADORA2B_MEDIATED_ANTI_INFLAMMATORY_CYTOKINES_PRODUCTION | 0.050468 | -0.05295 | 1.114507 | 0.269112 | 0.627033 | -5.27532 |
| WP_AUTOPHAGY | -0.0752 | -0.04406 | -1.1143 | 0.269202 | 0.627033 | -5.27553 |
| MARTINEZ_RESPONSE_TO_TRABECTEDIN_DN | -0.07929 | -0.06534 | -1.11426 | 0.269216 | 0.627033 | -5.27556 |
| REACTOME_CHONDROITIN_SULFATE_BIOSYNTHESIS | 0.075578 | -0.00597 | 1.11412 | 0.269277 | 0.627033 | -5.27571 |
| WU_APOPTOSIS_BY_CDKN1A_VIA_TP53 | -0.08997 | -0.02983 | -1.11337 | 0.269595 | 0.627544 | -5.27647 |
| REACTOME_COPII_MEDIATED_VESICLE_TRANSPORT | -0.06917 | -0.0516 | -1.11218 | 0.270105 | 0.628501 | -5.27769 |
| LUCAS_HNF4A_TARGETS_UP | -0.05297 | -0.13422 | -1.11168 | 0.270318 | 0.628767 | -5.2782 |
| XU_HGF_TARGETS_REPRESSED_BY_AKT1_DN | -0.03262 | -0.06597 | -1.11115 | 0.270543 | 0.62906 | -5.27873 |
| GAUSSMANN_MLL_AF4_FUSION_TARGETS_B_DN | -0.07069 | -0.01068 | -1.11089 | 0.270655 | 0.629091 | -5.279 |
| REACTOME_UB_SPECIFIC_PROCESSING_PROTEASES | -0.0603 | -0.10554 | -1.11052 | 0.270814 | 0.629183 | -5.27937 |
| SOTIRIOU_BREAST_CANCER_GRADE_1_VS_3_UP | -0.07639 | -0.0826 | -1.11033 | 0.270893 | 0.629183 | -5.27956 |
| SEIDEN_MET_SIGNALING | -0.12245 | -0.01509 | -1.10947 | 0.271261 | 0.629632 | -5.28043 |
| BLANCO_MELO_COVID19_SARS_COV_2_INFECTION_A594_CELLS_DN | 0.046537 | -0.17236 | 1.109058 | 0.271439 | 0.629632 | -5.28085 |
| REACTOME_BINDING_OF_TCF_LEF_CTNNB1_TO_TARGET_GENE_PROMOTERS | -0.08091 | 0.002618 | -1.10891 | 0.271501 | 0.629632 | -5.281 |
| PLASARI_TGFB1_SIGNALING_VIA_NFIC_10HR_DN | 0.06015 | -0.02651 | 1.108873 | 0.271518 | 0.629632 | -5.28104 |
| MIZUKAMI_HYPOXIA_UP | 0.085547 | -0.00564 | 1.108727 | 0.271581 | 0.629632 | -5.28119 |
| REACTOME_TRANSPORT_OF_FATTY_ACIDS | 0.096785 | -0.01717 | 1.107941 | 0.271917 | 0.63002 | -5.28199 |
| REACTOME_TRIGLYCERIDE_BIOSYNTHESIS | 0.0684 | -0.06421 | 1.107657 | 0.272039 | 0.63002 | -5.28227 |
| WP_PHOSPHOINOSITIDES_METABOLISM | -0.05735 | -0.13088 | -1.10743 | 0.272135 | 0.63002 | -5.2825 |
| RORIE_TARGETS_OF_EWSR1_FLI1_FUSION_UP | -0.05296 | -0.41176 | -1.10741 | 0.272144 | 0.63002 | -5.28252 |
| WP_HEART_DEVELOPMENT | 0.0575 | -0.04932 | 1.107033 | 0.272307 | 0.630167 | -5.2829 |
| MIZUSHIMA_AUTOPHAGOSOME_FORMATION | -0.08764 | -0.01313 | -1.1066 | 0.272493 | 0.630176 | -5.28334 |
| REACTOME_ER_TO_GOLGI_ANTEROGRADE_TRANSPORT | -0.06623 | -0.02332 | -1.1064 | 0.272579 | 0.630176 | -5.28355 |
| BROWNE_HCMV_INFECTION_8HR_DN | 0.050893 | -0.05716 | 1.106269 | 0.272635 | 0.630176 | -5.28368 |
| MIYAGAWA_TARGETS_OF_EWSR1_ETS_FUSIONS_UP | 0.038339 | -0.06711 | 1.106102 | 0.272706 | 0.630176 | -5.28385 |
| REACTOME_DEX_H_BOX_HELICASES_ACTIVATE_TYPE_I_IFN_AND_INFLAMMATORY_CYTOKINES_PRODUCTION | 0.109091 | -0.10679 | 1.105374 | 0.273019 | 0.630656 | -5.28458 |
| REACTOME_TOLL_LIKE_RECEPTOR_TLR1_TLR2_CASCADE | 0.050663 | -0.07053 | 1.105158 | 0.273112 | 0.630656 | -5.2848 |
| REACTOME_EPIGENETIC_REGULATION_OF_GENE_EXPRESSION | -0.05807 | -0.15902 | -1.10426 | 0.273498 | 0.631317 | -5.28571 |
| REACTOME_TRANSCRIPTION_OF_E2F_TARGETS_UNDER_NEGATIVE_CONTROL_BY_P107_RBL1_AND_P130_RBL2_IN_COMPLEX_WITH_HDAC1 | -0.07231 | -0.00269 | -1.10287 | 0.274099 | 0.632474 | -5.28711 |
| BIOCARTA_P35ALZHEIMERS_PATHWAY | 0.091687 | -0.00636 | 1.102119 | 0.274421 | 0.632914 | -5.28787 |
| KEGG_TYPE_II_DIABETES_MELLITUS | 0.034706 | -0.16958 | 1.10171 | 0.274598 | 0.632914 | -5.28828 |
| GU_PDEF_TARGETS_UP | 0.067959 | -0.017 | 1.101505 | 0.274686 | 0.632914 | -5.28848 |
| WP_DNA_IRDAMAGE_AND_CELLULAR_RESPONSE_VIA_ATR | -0.04923 | -0.1615 | -1.1015 | 0.274687 | 0.632914 | -5.28849 |
| BANDRES_RESPONSE_TO_CARMUSTIN_MGMT_24HR_UP | -0.0889 | -0.01967 | -1.1002 | 0.27525 | 0.633981 | -5.2898 |
| CHOW_RASSF1_TARGETS_UP | -0.09289 | -0.05037 | -1.09972 | 0.275459 | 0.634229 | -5.29028 |
| PID_RAS_PATHWAY | -0.04485 | -0.06446 | -1.09949 | 0.275557 | 0.634229 | -5.29051 |
| BIOCARTA_SKP2E2F_PATHWAY | -0.08197 | -0.0121 | -1.09913 | 0.275714 | 0.634276 | -5.29088 |
| REACTOME_VASOPRESSIN_REGULATES_RENAL_WATER_HOMEOSTASIS_VIA_AQUAPORINS | -0.03501 | -0.08138 | -1.09898 | 0.275777 | 0.634276 | -5.29102 |
| VALK_AML_WITH_CEBPA | 0.05142 | -0.05455 | 1.097643 | 0.276357 | 0.635165 | -5.29237 |
| ZHANG_RESPONSE_TO_CANTHARIDIN_UP | 0.091646 | 0.004892 | 1.097631 | 0.276363 | 0.635165 | -5.29238 |
| KEGG_CITRATE_CYCLE_TCA_CYCLE | -0.09807 | -0.03499 | -1.0972 | 0.276552 | 0.635166 | -5.29282 |
| REACTOME_INTERLEUKIN_6_FAMILY_SIGNALING | 0.069334 | 0.004036 | 1.09717 | 0.276563 | 0.635166 | -5.29284 |
| BOSCO_ALLERGEN_INDUCED_TH2_ASSOCIATED_MODULE | 0.058724 | -0.03036 | 1.096184 | 0.276991 | 0.63592 | -5.29383 |
| MARTIN_VIRAL_GPCR_SIGNALING_DN | 0.031916 | -0.22385 | 1.095661 | 0.277218 | 0.636211 | -5.29435 |
| REACTOME_PYRIMIDINE_CATABOLISM | 0.063573 | -0.0144 | 1.09419 | 0.277858 | 0.637386 | -5.29583 |
| NICK_RESPONSE_TO_PROC_TREATMENT_UP | -0.10919 | -0.00251 | -1.09402 | 0.27793 | 0.637386 | -5.29599 |
| WP_WNT_SIGNALING_IN_KIDNEY_DISEASE | -0.04291 | -0.10143 | -1.09215 | 0.278746 | 0.639014 | -5.29787 |
| REACTOME_AUF1_HNRNP_D0_BINDS_AND_DESTABILIZES_MRNA | -0.06755 | -0.28563 | -1.09139 | 0.279077 | 0.639014 | -5.29862 |
| WP_SARSCOV2_INNATE_IMMUNITY_EVASION_AND_CELLSPECIFIC_IMMUNE_RESPONSE | 0.047424 | -0.16021 | 1.091059 | 0.279223 | 0.639014 | -5.29896 |
| WP_PHYSIOLOGICAL_AND_PATHOLOGICAL_HYPERTROPHY_OF_THE_HEART | -0.05501 | -0.07876 | -1.09087 | 0.279304 | 0.639014 | -5.29914 |
| REACTOME_DNA_DOUBLE_STRAND_BREAK_REPAIR | -0.0483 | -0.08108 | -1.09085 | 0.279312 | 0.639014 | -5.29916 |
| WP_OXIDATIVE_PHOSPHORYLATION | -0.06261 | -0.24352 | -1.09074 | 0.279363 | 0.639014 | -5.29928 |
| WP_SULFATION_BIOTRANSFORMATION_REACTION | 0.063359 | 0.016714 | 1.0906 | 0.279423 | 0.639014 | -5.29941 |
| CHESLER_BRAIN_D6MIT150_QTL_TRANS | 0.0704 | -0.01057 | 1.090353 | 0.279531 | 0.639014 | -5.29966 |
| PURBEY_TARGETS_OF_CTBP1_AND_SATB1_UP | -0.05114 | -0.11693 | -1.09033 | 0.279543 | 0.639014 | -5.29969 |
| SPIELMAN_LYMPHOBLAST_EUROPEAN_VS_ASIAN_DN | -0.08447 | -0.08067 | -1.09001 | 0.279681 | 0.639099 | -5.3 |
| FOROUTAN_PRODRANK_TGFB_EMT_UP | 0.068712 | -0.05958 | 1.08922 | 0.280027 | 0.639659 | -5.30079 |
| BIOCARTA_DICER_PATHWAY | -0.12072 | -0.00539 | -1.08857 | 0.28031 | 0.640076 | -5.30143 |
| BIOCARTA_NO1_PATHWAY | -0.05516 | -0.0459 | -1.0881 | 0.280516 | 0.640223 | -5.3019 |
| WP_BDNFTRKB_SIGNALING | -0.05736 | -0.08152 | -1.08771 | 0.280687 | 0.640223 | -5.30229 |
| HADDAD_B_LYMPHOCYTE_PROGENITOR | -0.04604 | -0.1347 | -1.08767 | 0.280706 | 0.640223 | -5.30233 |
| FIGUEROA_AML_METHYLATION_CLUSTER_3_UP | -0.02916 | -0.08618 | -1.08751 | 0.280777 | 0.640223 | -5.30249 |
| ZHANG_INTERFERON_RESPONSE | 0.085688 | -0.08095 | 1.086681 | 0.281139 | 0.64082 | -5.30332 |
| REACTOME_SIGNALING_BY_INSULIN_RECEPTOR | -0.03998 | -0.08365 | -1.08618 | 0.28136 | 0.641038 | -5.30382 |
| DIERICK_SEROTONIN_FUNCTION_GENES | -0.04878 | -0.45999 | -1.08563 | 0.2816 | 0.641038 | -5.30436 |
| REACTOME_ACTIVATION_OF_NMDA_RECEPTORS_AND_POSTSYNAPTIC_EVENTS | -0.0391 | -0.05244 | -1.08557 | 0.281627 | 0.641038 | -5.30442 |
| BIOCARTA_STRESS_PATHWAY | -0.06657 | -0.23444 | -1.08526 | 0.281761 | 0.641038 | -5.30472 |
| REACTOME_DISEASES_OF_MISMATCH_REPAIR_MMR | -0.10431 | 0.027212 | -1.08518 | 0.281798 | 0.641038 | -5.30481 |
| SA_B_CELL_RECEPTOR_COMPLEXES | 0.075043 | -0.06923 | 1.085087 | 0.281839 | 0.641038 | -5.3049 |
| REACTOME_HSP90_CHAPERONE_CYCLE_FOR_STEROID_HORMONE_RECEPTORS_SHR_IN_THE_PRESENCE_OF_LIGAND | -0.06423 | -0.16184 | -1.08438 | 0.282149 | 0.641435 | -5.3056 |
| REACTOME_CYTOKINE_SIGNALING_IN_IMMUNE_SYSTEM | 0.039457 | -0.17748 | 1.084232 | 0.282215 | 0.641435 | -5.30575 |
| BIOCARTA_ERAD_PATHWAY | -0.08627 | -0.06039 | -1.08387 | 0.282373 | 0.641512 | -5.3061 |
| WP_LINOLEIC_ACID_METABOLISM_AFFECTED_BY_CORONAVIRUS_INFECTION | -0.10253 | 0.012368 | -1.08369 | 0.282456 | 0.641512 | -5.30629 |
| SAKAI_TUMOR_INFILTRATING_MONOCYTES_UP | -0.05525 | -0.09923 | -1.0831 | 0.282713 | 0.641512 | -5.30687 |
| ABBUD_LIF_SIGNALING_2_UP | 0.081031 | 0.007159 | 1.082537 | 0.282962 | 0.641512 | -5.30743 |
| BROWNE_HCMV_INFECTION_1HR_DN | -0.02403 | -0.15549 | -1.08248 | 0.282988 | 0.641512 | -5.30749 |
| GOTTWEIN_TARGETS_OF_KSHV_MIR_K12_11 | -0.07504 | -0.02124 | -1.08231 | 0.283061 | 0.641512 | -5.30765 |
| WP_FACTORS_AND_PATHWAYS_AFFECTING_INSULINLIKE_GROWTH_FACTOR_IGF1AKT_SIGNALING | -0.06165 | -0.03466 | -1.08226 | 0.283083 | 0.641512 | -5.3077 |
| RAO_BOUND_BY_SALL4_ISOFORM_A | -0.02734 | -0.06804 | -1.08212 | 0.283147 | 0.641512 | -5.30784 |
| VALK_AML_CLUSTER_7 | 0.061611 | -0.20038 | 1.081934 | 0.283228 | 0.641512 | -5.30802 |
| BYSTROEM_CORRELATED_WITH_IL5_DN | -0.08025 | -0.02315 | -1.08187 | 0.283257 | 0.641512 | -5.30809 |
| TOMLINS_PROSTATE_CANCER_DN | -0.09264 | -0.01404 | -1.08087 | 0.283698 | 0.642143 | -5.30908 |
| HOELZEL_NF1_TARGETS_UP | 0.037652 | -0.04945 | 1.080778 | 0.283738 | 0.642143 | -5.30917 |
| EPPERT_CE_HSC_LSC | -0.06281 | -0.05377 | -1.08018 | 0.284 | 0.642509 | -5.30976 |
| REACTOME_INFECTION_WITH_MYCOBACTERIUM_TUBERCULOSIS | 0.065685 | -0.19208 | 1.079754 | 0.28419 | 0.64271 | -5.31018 |
| PAL_PRMT5_TARGETS_DN | 0.038877 | -0.32721 | 1.079255 | 0.284411 | 0.642981 | -5.31067 |
| REACTOME_REGULATION_OF_PLK1_ACTIVITY_AT_G2_M_TRANSITION | -0.0556 | -0.12423 | -1.07836 | 0.284809 | 0.643521 | -5.31156 |
| CHEN_PDGF_TARGETS | 0.095165 | -0.01255 | 1.078259 | 0.284852 | 0.643521 | -5.31166 |
| GRAHAM_CML_DIVIDING_VS_NORMAL_QUIESCENT_UP | -0.06301 | -0.04856 | -1.07787 | 0.285026 | 0.643541 | -5.31204 |
| REACTOME_TRANSPORT_TO_THE_GOLGI_AND_SUBSEQUENT_MODIFICATION | -0.0572 | -0.03467 | -1.07747 | 0.285201 | 0.643541 | -5.31243 |
| EGUCHI_CELL_CYCLE_RB1_TARGETS | -0.09179 | -0.02889 | -1.07735 | 0.285254 | 0.643541 | -5.31255 |
| REACTOME_FCERI_MEDIATED_NF_KB_ACTIVATION | -0.06814 | -0.17923 | -1.07733 | 0.285266 | 0.643541 | -5.31258 |
| LANDIS_BREAST_CANCER_PROGRESSION_UP | 0.057296 | -0.04459 | 1.077095 | 0.285368 | 0.643544 | -5.3128 |
| WP_OMEGA3_OMEGA6_FATTY_ACID_SYNTHESIS | -0.0809 | 0.000971 | -1.07685 | 0.285477 | 0.643562 | -5.31305 |
| STEGER_ADIPOGENESIS_DN | 0.096949 | 0.002211 | 1.076542 | 0.285613 | 0.64364 | -5.31335 |
| BENPORATH_ES_CORE_NINE | 0.080067 | -0.00303 | 1.07556 | 0.286049 | 0.644394 | -5.31431 |
| VERRECCHIA_RESPONSE_TO_TGFB1_C4 | 0.082223 | -0.08182 | 1.074991 | 0.286302 | 0.64464 | -5.31488 |
| KAPOSI_LIVER_CANCER_MET_UP | -0.09055 | -0.00266 | -1.07469 | 0.286434 | 0.64464 | -5.31517 |
| WP_AMINO_ACID_METABOLISM | -0.05715 | -0.05795 | -1.07443 | 0.286553 | 0.64464 | -5.31543 |
| REACTOME_ABC_TRANSPORTERS_IN_LIPID_HOMEOSTASIS | -0.0513 | 0.004551 | -1.07427 | 0.28662 | 0.64464 | -5.31558 |
| GENTLES_LEUKEMIC_STEM_CELL_DN | 0.064057 | -0.08349 | 1.074175 | 0.286664 | 0.64464 | -5.31568 |
| REACTOME_RUNX3_REGULATES_IMMUNE_RESPONSE_AND_CELL_MIGRATION | 0.104672 | 0.009498 | 1.073434 | 0.286994 | 0.644709 | -5.31641 |
| BIOCARTA_AKT_PATHWAY | -0.08377 | 0.004617 | -1.0734 | 0.287008 | 0.644709 | -5.31644 |
| KEGG_PROTEASOME | -0.06645 | -0.29874 | -1.07332 | 0.287047 | 0.644709 | -5.31652 |
| REACTOME_TRAIL_SIGNALING | 0.087894 | 0.00137 | 1.073195 | 0.2871 | 0.644709 | -5.31664 |
| TAKAYAMA_BOUND_BY_AR | 0.083791 | -0.02173 | 1.072907 | 0.287229 | 0.64476 | -5.31692 |
| KORKOLA_EMBRYONAL_CARCINOMA | -0.02376 | -0.70125 | -1.07256 | 0.287384 | 0.64476 | -5.31727 |
| REACTOME_RAB_GERANYLGERANYLATION | -0.0578 | -0.02791 | -1.07246 | 0.287427 | 0.64476 | -5.31736 |
| WP_BASE_EXCISION_REPAIR | -0.08955 | -0.00214 | -1.0717 | 0.287768 | 0.645107 | -5.31811 |
| VALK_AML_WITH_FLT3_ITD | -0.05011 | 7.64E-05 | -1.07166 | 0.287784 | 0.645107 | -5.31815 |
| BIOCARTA_NPP1_PATHWAY | 0.079958 | -0.01011 | 1.070277 | 0.288402 | 0.645982 | -5.3195 |
| GOLDRATH_HOMEOSTATIC_PROLIFERATION | -0.07689 | -0.05553 | -1.06931 | 0.288832 | 0.645982 | -5.32045 |
| BURTON_ADIPOGENESIS_10 | -0.0896 | -0.19447 | -1.06904 | 0.288956 | 0.645982 | -5.32072 |
| REACTOME_NADE_MODULATES_DEATH_SIGNALLING | -0.08981 | -0.13649 | -1.06898 | 0.28898 | 0.645982 | -5.32077 |
| REACTOME_ADENYLATE_CYCLASE_INHIBITORY_PATHWAY | -0.0646 | -0.0743 | -1.06869 | 0.289111 | 0.645982 | -5.32106 |
| GEISS_RESPONSE_TO_DSRNA_UP | 0.064261 | -0.18113 | 1.068487 | 0.289202 | 0.645982 | -5.32125 |
| REACTOME_TRAF3_DEPENDENT_IRF_ACTIVATION_PATHWAY | 0.07691 | -0.07289 | 1.068456 | 0.289216 | 0.645982 | -5.32128 |
| BROWNE_HCMV_INFECTION_18HR_UP | -0.05072 | -0.11088 | -1.06835 | 0.289262 | 0.645982 | -5.32139 |
| WP_PDGFRBETA_PATHWAY | -0.09612 | -0.01863 | -1.06829 | 0.28929 | 0.645982 | -5.32145 |
| COATES_MACROPHAGE_M1_VS_M2_DN | 0.045938 | -0.20812 | 1.068188 | 0.289335 | 0.645982 | -5.32155 |
| MOOTHA_ROS | -0.1103 | -0.09818 | -1.06794 | 0.289448 | 0.645982 | -5.32179 |
| BIOCARTA_MHC_PATHWAY | 0.019159 | -0.79043 | 1.0679 | 0.289465 | 0.645982 | -5.32183 |
| MOOTHA_GLUCONEOGENESIS | 0.059973 | -0.09342 | 1.067834 | 0.289494 | 0.645982 | -5.32189 |
| GAUSSMANN_MLL_AF4_FUSION_TARGETS_G_UP | -0.02348 | -0.09055 | -1.06733 | 0.289722 | 0.646195 | -5.32239 |
| MCCABE_HOXC6_TARGETS_CANCER_DN | -0.05902 | -0.34883 | -1.06664 | 0.290027 | 0.646195 | -5.32305 |
| THEILGAARD_NEUTROPHIL_AT_SKIN_WOUND_UP | 0.054677 | -0.2285 | 1.066555 | 0.290067 | 0.646195 | -5.32314 |
| KEGG_MATURITY_ONSET_DIABETES_OF_THE_YOUNG | 0.080834 | -0.04778 | 1.066363 | 0.290153 | 0.646195 | -5.32333 |
| HELLER_HDAC_TARGETS_UP | 0.035433 | -0.19427 | 1.066268 | 0.290196 | 0.646195 | -5.32342 |
| REACTOME_REGULATION_OF_INSULIN_SECRETION | 0.033853 | -0.05218 | 1.066034 | 0.290301 | 0.646195 | -5.32365 |
| RAMASWAMY_METASTASIS_UP | -0.06313 | -0.11195 | -1.06584 | 0.29039 | 0.646195 | -5.32384 |
| DAZARD_RESPONSE_TO_UV_NHEK_UP | 0.041485 | -0.18333 | 1.065809 | 0.290402 | 0.646195 | -5.32387 |
| RAO_BOUND_BY_SALL4 | -0.03497 | -0.068 | -1.06516 | 0.290695 | 0.64624 | -5.32451 |
| HEIDENBLAD_AMPLICON_8Q24_UP | -0.03909 | -0.12322 | -1.06514 | 0.290704 | 0.64624 | -5.32453 |
| BIOCARTA_GHRELIN_PATHWAY | 0.066886 | -0.01813 | 1.065085 | 0.290727 | 0.64624 | -5.32458 |
| REACTOME_MITOTIC_G2_G2_M_PHASES | -0.04908 | -0.1477 | -1.06444 | 0.291017 | 0.64666 | -5.32521 |
| REACTOME_GLUCONEOGENESIS | 0.060041 | -0.06273 | 1.063778 | 0.291315 | 0.647094 | -5.32585 |
| BLUM_RESPONSE_TO_SALIRASIB_DN | -0.06197 | -0.08436 | -1.06349 | 0.291444 | 0.647156 | -5.32613 |
| PLASARI_TGFB1_TARGETS_10HR_DN | -0.0569 | -0.04617 | -1.06246 | 0.291906 | 0.647956 | -5.32713 |
| BLANCO_MELO_MERS_COV_INFECTION_MCR5_CELLS_DN | 0.047507 | -0.23567 | 1.062119 | 0.292061 | 0.647986 | -5.32746 |
| REACTOME_TICAM1_TRAF6_DEPENDENT_INDUCTION_OF_TAK1_COMPLEX | 0.070999 | 0.009948 | 1.061842 | 0.292186 | 0.647986 | -5.32773 |
| LEI_HOXC8_TARGETS_DN | 0.058807 | -0.11394 | 1.061756 | 0.292225 | 0.647986 | -5.32782 |
| WP_SPLICING_FACTOR_NOVA_REGULATED_SYNAPTIC_PROTEINS | 0.041167 | -0.05557 | 1.06136 | 0.292403 | 0.648155 | -5.3282 |
| XU_GH1_AUTOCRINE_TARGETS_DN | -0.05009 | -0.07544 | -1.06104 | 0.292547 | 0.648238 | -5.32851 |
| REACTOME_SIGNALING_BY_CTNNB1_PHOSPHO_SITE_MUTANTS | -0.07543 | 0.007214 | -1.06083 | 0.292644 | 0.648238 | -5.32872 |
| WP_15Q133_COPY_NUMBER_VARIATION_SYNDROME | 0.038889 | -0.30565 | 1.060142 | 0.292952 | 0.648499 | -5.32938 |
| KEGG_GALACTOSE_METABOLISM | 0.05298 | -0.10596 | 1.060113 | 0.292966 | 0.648499 | -5.32941 |
| FUKUSHIMA_TNFSF11_TARGETS | -0.05119 | -0.29262 | -1.05956 | 0.293215 | 0.648807 | -5.32995 |
| REACTOME_TLR3_MEDIATED_TICAM1_DEPENDENT_PROGRAMMED_CELL_DEATH | 0.080245 | -0.11684 | 1.059167 | 0.293393 | 0.648807 | -5.33033 |
| REACTOME_GAP_FILLING_DNA_REPAIR_SYNTHESIS_AND_LIGATION_IN_GG_NER | -0.08899 | -0.00351 | -1.05913 | 0.293411 | 0.648807 | -5.33037 |
| REACTOME_COLLAGEN_FORMATION | 0.064721 | -0.06483 | 1.0585 | 0.293694 | 0.64918 | -5.33098 |
| XU_HGF_TARGETS_INDUCED_BY_AKT1_6HR | 0.070622 | -0.0488 | 1.058303 | 0.293784 | 0.64918 | -5.33117 |
| NIKOLSKY_BREAST_CANCER_1Q21_AMPLICON | -0.04418 | -0.14091 | -1.05798 | 0.293931 | 0.649281 | -5.33148 |
| MEISSNER_BRAIN_HCP_WITH_H3K4ME2 | 0.078284 | -0.04216 | 1.057718 | 0.294048 | 0.649314 | -5.33173 |
| BILANGES_SERUM_AND_RAPAMYCIN_SENSITIVE_GENES | -0.0754 | -0.14951 | -1.05679 | 0.294469 | 0.649976 | -5.33263 |
| WP_16P112_PROXIMAL_DELETION_SYNDROME | -0.04998 | -0.0328 | -1.05661 | 0.294552 | 0.649976 | -5.33281 |
| SMITH_LIVER_CANCER | -0.0617 | -0.1312 | -1.05569 | 0.294968 | 0.650132 | -5.3337 |
| BIOCARTA_ETC_PATHWAY | -0.11566 | -0.00158 | -1.05566 | 0.294981 | 0.650132 | -5.33373 |
| KYNG_DNA_DAMAGE_BY_GAMMA_RADIATION | 0.047559 | -0.0879 | 1.055647 | 0.294987 | 0.650132 | -5.33374 |
| WACKER_HYPOXIA_TARGETS_OF_VHL | 0.096701 | -0.0187 | 1.055549 | 0.295031 | 0.650132 | -5.33383 |
| HOFFMANN_LARGE_TO_SMALL_PRE_BII_LYMPHOCYTE_DN | 0.035432 | -0.12342 | 1.055117 | 0.295227 | 0.650338 | -5.33425 |
| CHEN_NEUROBLASTOMA_COPY_NUMBER_GAINS | -0.04326 | -0.08231 | -1.05306 | 0.296161 | 0.651602 | -5.33623 |
| KEGG_RIBOSOME | -0.07559 | -0.17293 | -1.05247 | 0.296431 | 0.651602 | -5.33681 |
| HOLLMANN_APOPTOSIS_VIA_CD40_UP | -0.06317 | -0.04358 | -1.0522 | 0.296555 | 0.651602 | -5.33707 |
| MARIADASON_REGULATED_BY_HISTONE_ACETYLATION_DN | -0.03807 | -0.10888 | -1.05211 | 0.296593 | 0.651602 | -5.33715 |
| MYLLYKANGAS_AMPLIFICATION_HOT_SPOT_6 | 0.087191 | 0.004773 | 1.051949 | 0.296668 | 0.651602 | -5.33731 |
| REACTOME_METABOLISM_OF_NITRIC_OXIDE_NOS3_ACTIVATION_AND_REGULATION | -0.07789 | -0.2506 | -1.05188 | 0.296698 | 0.651602 | -5.33737 |
| REACTOME_RHO_GTPASES_ACTIVATE_WASPS_AND_WAVES | -0.08015 | -0.0379 | -1.05174 | 0.296763 | 0.651602 | -5.33751 |
| GARGALOVIC_RESPONSE_TO_OXIDIZED_PHOSPHOLIPIDS_SALMON_DN | 0.07397 | -0.01411 | 1.051614 | 0.29682 | 0.651602 | -5.33763 |
| HASINA_NOL7_TARGETS_DN | 0.087539 | -0.01123 | 1.051601 | 0.296826 | 0.651602 | -5.33764 |
| REACTOME_PEROXISOMAL_PROTEIN_IMPORT | -0.04698 | -0.0764 | -1.05149 | 0.296877 | 0.651602 | -5.33775 |
| FOROUTAN_INTEGRATED_TGFB_EMT_UP | 0.073402 | -0.07814 | 1.05136 | 0.296936 | 0.651602 | -5.33787 |
| REACTOME_ACTIVATION_OF_THE_TFAP2_AP_2_FAMILY_OF_TRANSCRIPTION_FACTORS | 0.061411 | 0.006124 | 1.051154 | 0.29703 | 0.651602 | -5.33807 |
| REACTOME_AURKA_ACTIVATION_BY_TPX2 | -0.05162 | -0.14656 | -1.0507 | 0.297237 | 0.651831 | -5.33851 |
| ISHIDA_E2F_TARGETS | -0.08285 | -0.04615 | -1.0502 | 0.297463 | 0.652104 | -5.33899 |
| REACTOME_APC_C_CDC20_MEDIATED_DEGRADATION_OF_CYCLIN_B | -0.08292 | -0.00433 | -1.04979 | 0.297651 | 0.652177 | -5.33938 |
| PUJANA_BREAST_CANCER_LIT_INT_NETWORK | -0.0531 | -0.10102 | -1.04968 | 0.297702 | 0.652177 | -5.33949 |
| STEIN_ESRRA_TARGETS_RESPONSIVE_TO_ESTROGEN_UP | -0.05456 | -0.20829 | -1.04913 | 0.297951 | 0.652498 | -5.34001 |
| WP_EFFECT_OF_PROGERIN_ON_GENES_INVOLVED_IN_HUTCHINSONGILFORD_PROGERIA_SYNDROME | -0.07959 | -0.01179 | -1.04847 | 0.298256 | 0.652941 | -5.34065 |
| KEGG_ENDOMETRIAL_CANCER | -0.04714 | -0.05347 | -1.04766 | 0.298627 | 0.653529 | -5.34143 |
| WP_CELLTYPE_DEPENDENT_SELECTIVITY_OF_CCK2R_SIGNALING | -0.06743 | 0.005037 | -1.0462 | 0.299296 | 0.654419 | -5.34283 |
| AMIT_EGF_RESPONSE_20_MCF10A | 0.072352 | -0.2556 | 1.046177 | 0.299304 | 0.654419 | -5.34285 |
| MATZUK_MATERNAL_EFFECT | 0.075705 | -0.09795 | 1.045999 | 0.299385 | 0.654419 | -5.34302 |
| REACTOME_SIGNALING_BY_WNT_IN_CANCER | -0.04129 | -0.01751 | -1.04587 | 0.299445 | 0.654419 | -5.34315 |
| WP_VITAMIN_B12_DISORDERS | -0.06325 | -0.01273 | -1.04542 | 0.299651 | 0.654423 | -5.34358 |
| MARSHALL_VIRAL_INFECTION_RESPONSE_UP | 0.062498 | -0.0946 | 1.045264 | 0.299723 | 0.654423 | -5.34373 |
| KEGG_LEISHMANIA_INFECTION | 0.02534 | -0.52856 | 1.04497 | 0.299857 | 0.654423 | -5.34401 |
| BILD_CTNNB1_ONCOGENIC_SIGNATURE | -0.05772 | -0.04642 | -1.04497 | 0.299858 | 0.654423 | -5.34401 |
| REACTOME_FLT3_SIGNALING_BY_CBL_MUTANTS | 0.111505 | 0.010004 | 1.044573 | 0.300039 | 0.654594 | -5.34439 |
| CUI_TCF21_TARGETS_2_UP | -0.03072 | -0.1068 | -1.04382 | 0.300386 | 0.654919 | -5.34511 |
| KREPPEL_CD99_TARGETS_DN | -0.09207 | -0.00559 | -1.0438 | 0.300394 | 0.654919 | -5.34513 |
| SIG_PIP3_SIGNALING_IN_CARDIAC_MYOCTES | -0.04563 | -0.08506 | -1.04308 | 0.300727 | 0.655039 | -5.34582 |
| ZHENG_GLIOBLASTOMA_PLASTICITY_DN | 0.050392 | -0.0756 | 1.043071 | 0.300729 | 0.655039 | -5.34582 |
| LU_EZH2_TARGETS_DN | -0.06646 | -0.09339 | -1.04284 | 0.300836 | 0.655039 | -5.34605 |
| GUTIERREZ_MULTIPLE_MYELOMA_UP | -0.08875 | -0.04981 | -1.04271 | 0.300894 | 0.655039 | -5.34617 |
| WP_ACE_INHIBITOR_PATHWAY | 0.061357 | -0.04229 | 1.042471 | 0.301005 | 0.655039 | -5.3464 |
| BIOCARTA_GSK3_PATHWAY | 0.076761 | -0.00731 | 1.04217 | 0.301144 | 0.655039 | -5.34668 |
| REACTOME_TRANSPORT_OF_BILE_SALTS_AND_ORGANIC_ACIDS_METAL_IONS_AND_AMINE_COMPOUNDS | 0.038003 | -0.13553 | 1.042113 | 0.30117 | 0.655039 | -5.34674 |
| MOTAMED_RESPONSE_TO_ANDROGEN_UP | 0.11928 | -0.02925 | 1.041828 | 0.301301 | 0.655101 | -5.34701 |
| REACTOME_SPERM_MOTILITY_AND_TAXES | 0.082712 | -0.10645 | 1.041186 | 0.301596 | 0.655355 | -5.34762 |
| REACTOME_PHASE_II_CONJUGATION_OF_COMPOUNDS | 0.033103 | -0.02684 | 1.041097 | 0.301637 | 0.655355 | -5.34771 |
| TERAMOTO_OPN_TARGETS_CLUSTER_5 | -0.06797 | -0.18408 | -1.04077 | 0.301789 | 0.655355 | -5.34802 |
| SEMENZA_HIF1_TARGETS | 0.070737 | -0.02663 | 1.04068 | 0.30183 | 0.655355 | -5.34811 |
| FARMER_BREAST_CANCER_CLUSTER_6 | -0.0826 | -0.00854 | -1.04031 | 0.301999 | 0.655412 | -5.34846 |
| KAMMINGA_SENESCENCE | -0.04556 | -0.02571 | -1.0396 | 0.302328 | 0.655412 | -5.34914 |
| WP_LEUKOCYTEINTRINSIC_HIPPO_PATHWAY_FUNCTIONS | -0.06105 | -0.02567 | -1.03935 | 0.302441 | 0.655412 | -5.34937 |
| REACTOME_TP53_REGULATES_TRANSCRIPTION_OF_CELL_DEATH_GENES | 0.049653 | -0.04566 | 1.039351 | 0.302443 | 0.655412 | -5.34937 |
| REACTOME_STAT5_ACTIVATION_DOWNSTREAM_OF_FLT3_ITD_MUTANTS | 0.088901 | 0.017746 | 1.039337 | 0.302449 | 0.655412 | -5.34939 |
| REACTOME_TAK1_ACTIVATES_NFKB_BY_PHOSPHORYLATION_AND_ACTIVATION_OF_IKKS_COMPLEX | 0.054979 | -0.15638 | 1.039266 | 0.302481 | 0.655412 | -5.34945 |
| ZHU_SKIL_TARGETS_DN | -0.06268 | 0.006009 | -1.03906 | 0.302577 | 0.655412 | -5.34965 |
| WANG_RESPONSE_TO_BEXAROTENE_UP | -0.04207 | -0.03806 | -1.0381 | 0.30302 | 0.655898 | -5.35056 |
| REACTOME_EUKARYOTIC_TRANSLATION_ELONGATION | -0.07576 | -0.15905 | -1.03797 | 0.303079 | 0.655898 | -5.35068 |
| ZHAN_LATE_DIFFERENTIATION_GENES_UP | -0.0879 | -0.00413 | -1.03783 | 0.303144 | 0.655898 | -5.35082 |
| KEGG_GRAFT_VERSUS_HOST_DISEASE | 0.015587 | -0.86474 | 1.037519 | 0.303288 | 0.655898 | -5.35112 |
| REACTOME_N_GLYCAN_TRIMMING_IN_THE_ER_AND_CALNEXIN_CALRETICULIN_CYCLE | -0.07208 | -0.16977 | -1.03746 | 0.303317 | 0.655898 | -5.35117 |
| MILICIC_FAMILIAL_ADENOMATOUS_POLYPOSIS_UP | 0.065739 | -0.07404 | 1.037018 | 0.30352 | 0.656115 | -5.35159 |
| BUCKANOVICH_T_LYMPHOCYTE_HOMING_ON_TUMOR_UP | -0.07793 | 0.000312 | -1.03675 | 0.303644 | 0.656159 | -5.35185 |
| MARTENS_BOUND_BY_PML_RARA_FUSION | 0.048126 | -0.05895 | 1.036443 | 0.303786 | 0.65623 | -5.35214 |
| WP_WHITE_FAT_CELL_DIFFERENTIATION | 0.064829 | -0.00226 | 1.036234 | 0.303883 | 0.65623 | -5.35234 |
| REACTOME_PHASE_2_PLATEAU_PHASE | 0.094294 | -0.01957 | 1.035988 | 0.303997 | 0.656254 | -5.35257 |
| LEE_RECENT_THYMIC_EMIGRANT | -0.05629 | -0.10229 | -1.03559 | 0.304183 | 0.656432 | -5.35295 |
| REACTOME_PRC2_METHYLATES_HISTONES_AND_DNA | -0.07613 | -0.08127 | -1.03504 | 0.304436 | 0.656757 | -5.35347 |
| REACTOME_SIGNALING_BY_ERBB2_IN_CANCER | -0.0494 | -0.04186 | -1.03465 | 0.304615 | 0.656802 | -5.35383 |
| KANG_DOXORUBICIN_RESISTANCE_UP | -0.08388 | -0.07482 | -1.0345 | 0.304685 | 0.656802 | -5.35398 |
| REACTOME_TRAFFICKING_OF_MYRISTOYLATED_PROTEINS_TO_THE_CILIUM | -0.10916 | -0.00303 | -1.0342 | 0.304826 | 0.656802 | -5.35427 |
| PID_AJDISS_2PATHWAY | 0.044948 | -0.04376 | 1.034104 | 0.30487 | 0.656802 | -5.35436 |
| WP_NRP1TRIGGERED_SIGNALING_PATHWAYS_IN_PANCREATIC_CANCER | 0.055925 | -0.06403 | 1.032659 | 0.305541 | 0.658024 | -5.35572 |
| KEGG_PROTEIN_EXPORT | -0.11549 | -0.00224 | -1.03207 | 0.305816 | 0.658394 | -5.35628 |
| HUANG_DASATINIB_RESISTANCE_DN | -0.0391 | -0.06099 | -1.03183 | 0.305928 | 0.658413 | -5.35651 |
| WP_NSP1_FROM_SARSCOV2_INHIBITS_TRANSLATION_INITIATION_IN_THE_HOST_CELL | -0.10733 | 0.001064 | -1.03156 | 0.30605 | 0.658453 | -5.35676 |
| MEISSNER_BRAIN_HCP_WITH_H3K27ME3 | 0.066608 | -0.04891 | 1.030971 | 0.306326 | 0.658823 | -5.35732 |
| REACTOME_SLC_TRANSPORTER_DISORDERS | -0.03061 | -0.0234 | -1.02933 | 0.307091 | 0.66004 | -5.35887 |
| REACTOME_GLUCURONIDATION | 0.079162 | -0.04586 | 1.029227 | 0.307138 | 0.66004 | -5.35897 |
| KAN_RESPONSE_TO_ARSENIC_TRIOXIDE | 0.052078 | -0.11569 | 1.029088 | 0.307203 | 0.66004 | -5.3591 |
| CAIRO_HEPATOBLASTOMA_POOR_SURVIVAL | -0.05941 | 0.010258 | -1.02841 | 0.307519 | 0.660496 | -5.35974 |
| GAUSSMANN_MLL_AF4_FUSION_TARGETS_D_DN | 0.078074 | -0.01278 | 1.028116 | 0.307657 | 0.660506 | -5.36002 |
| REACTOME_SIGNALING_BY_FGFR3 | -0.04872 | -0.01711 | -1.02796 | 0.307731 | 0.660506 | -5.36016 |
| REACTOME_MITOPHAGY | -0.08204 | -0.2004 | -1.0275 | 0.307944 | 0.660723 | -5.36059 |
| MORI_PLASMA_CELL_DN | 0.070224 | -0.00095 | 1.026778 | 0.308282 | 0.660723 | -5.36128 |
| REACTOME_NUCLEAR_EVENTS_STIMULATED_BY_ALK_SIGNALING_IN_CANCER | 0.087501 | -0.00865 | 1.026729 | 0.308304 | 0.660723 | -5.36132 |
| HU_ANGIOGENESIS_UP | 0.075633 | -0.00678 | 1.026654 | 0.30834 | 0.660723 | -5.36139 |
| SHIRAISHI_PLZF_TARGETS_UP | 0.069074 | -0.07143 | 1.026414 | 0.308452 | 0.660723 | -5.36162 |
| OZANNE_AP1_TARGETS_UP | 0.073792 | -0.05248 | 1.026407 | 0.308455 | 0.660723 | -5.36162 |
| FOROUTAN_TGFB_EMT_UP | 0.066187 | -0.05904 | 1.026019 | 0.308637 | 0.660835 | -5.36199 |
| LINDGREN_BLADDER_CANCER_CLUSTER_3_UP | -0.05484 | -0.10537 | -1.02585 | 0.308715 | 0.660835 | -5.36215 |
| REACTOME_POST_TRANSLATIONAL_PROTEIN_MODIFICATION | -0.03927 | -0.08796 | -1.02543 | 0.308913 | 0.661015 | -5.36255 |
| REACTOME_CELL_DEATH_SIGNALLING_VIA_NRAGE_NRIF_AND_NADE | 0.035311 | -0.0895 | 1.025228 | 0.309007 | 0.661015 | -5.36273 |
| ZHAN_VARIABLE_EARLY_DIFFERENTIATION_GENES_DN | -0.08933 | -0.04057 | -1.02471 | 0.309252 | 0.661316 | -5.36322 |
| BURTON_ADIPOGENESIS_PEAK_AT_2HR | 0.082266 | -0.13275 | 1.024193 | 0.309492 | 0.661607 | -5.3637 |
| MCCOLLUM_GELDANAMYCIN_RESISTANCE_DN | -0.06762 | 0.01278 | -1.02366 | 0.30974 | 0.661683 | -5.3642 |
| REACTOME_ORGANIC_CATION_TRANSPORT | -0.07407 | 0.002028 | -1.02351 | 0.309811 | 0.661683 | -5.36434 |
| PID_IL6_7_PATHWAY | 0.063074 | -0.00307 | 1.023452 | 0.309839 | 0.661683 | -5.3644 |
| FERRANDO_LYL1_NEIGHBORS | 0.076624 | -0.06598 | 1.022757 | 0.310165 | 0.662157 | -5.36505 |
| RAFFEL_VEGFA_TARGETS_UP | -0.06673 | -0.00893 | -1.02243 | 0.310317 | 0.662259 | -5.36536 |
| REACTOME_CROSS_PRESENTATION_OF_SOLUBLE_EXOGENOUS_ANTIGENS_ENDOSOMES | -0.06374 | -0.26427 | -1.02175 | 0.31064 | 0.662546 | -5.366 |
| BIOCARTA_CREB_PATHWAY | -0.0589 | -0.11308 | -1.0213 | 0.31085 | 0.662546 | -5.36642 |
| WAKABAYASHI_ADIPOGENESIS_PPARG_RXRA_BOUND_WITH_H4K20ME1_MARK | -0.05786 | -0.06496 | -1.02129 | 0.310855 | 0.662546 | -5.36643 |
| CERVERA_SDHB_TARGETS_1_DN | 0.034832 | -0.18795 | 1.021261 | 0.310868 | 0.662546 | -5.36645 |
| WP_MOLYBDENUM_COFACTOR_MOCO_BIOSYNTHESIS | -0.097 | -0.00307 | -1.02065 | 0.311154 | 0.662934 | -5.36702 |
| CAFFAREL_RESPONSE_TO_THC_24HR_3_DN | -0.0701 | -0.0018 | -1.02024 | 0.311347 | 0.663046 | -5.36741 |
| COLDREN_GEFITINIB_RESISTANCE_UP | -0.0677 | -0.02942 | -1.0201 | 0.311415 | 0.663046 | -5.36754 |
| REACTOME_SYNTHESIS_OF_LIPOXINS_LX | -0.06348 | -0.24301 | -1.01974 | 0.311584 | 0.663183 | -5.36788 |
| MORI_PLASMA_CELL_UP | -0.08281 | -0.03559 | -1.01898 | 0.31194 | 0.663719 | -5.36858 |
| WANG_NFKB_TARGETS | 0.069407 | 0.02023 | 1.01867 | 0.312087 | 0.663812 | -5.36888 |
| BROWNE_HCMV_INFECTION_16HR_UP | -0.04797 | -0.07031 | -1.01727 | 0.312746 | 0.664451 | -5.37018 |
| PID_RANBP2_PATHWAY | -0.08849 | 0.016611 | -1.01694 | 0.312905 | 0.664451 | -5.37049 |
| REACTOME_FACTORS_INVOLVED_IN_MEGAKARYOCYTE_DEVELOPMENT_AND_PLATELET_PRODUCTION | 0.027837 | -0.04534 | 1.0164 | 0.313159 | 0.664451 | -5.37099 |
| KEGG_PRIMARY_IMMUNODEFICIENCY | 0.052368 | -0.31075 | 1.015931 | 0.31338 | 0.664451 | -5.37143 |
| KEGG_ADHERENS_JUNCTION | -0.05194 | -0.1087 | -1.01559 | 0.313542 | 0.664451 | -5.37175 |
| BLANCO_MELO_INFLUENZA_A_INFECTION_A594_CELLS_DN | 0.03128 | -0.20283 | 1.015489 | 0.313589 | 0.664451 | -5.37184 |
| SASAKI_TARGETS_OF_TP73_AND_TP63 | 0.079497 | -0.10651 | 1.01539 | 0.313636 | 0.664451 | -5.37193 |
| REACTOME_CLASS_C_3_METABOTROPIC_GLUTAMATE_PHEROMONE_RECEPTORS | 0.040094 | -0.41606 | 1.015357 | 0.313652 | 0.664451 | -5.37197 |
| PID_HDAC_CLASSI_PATHWAY | -0.05245 | -0.14478 | -1.01532 | 0.313671 | 0.664451 | -5.372 |
| REACTOME_SIGNALING_BY_SCF_KIT | 0.055469 | -0.02686 | 1.014995 | 0.313823 | 0.664451 | -5.3723 |
| REACTOME_COPI_INDEPENDENT_GOLGI_TO_ER_RETROGRADE_TRAFFIC | -0.06438 | 0.000689 | -1.01495 | 0.313842 | 0.664451 | -5.37234 |
| YAO_TEMPORAL_RESPONSE_TO_PROGESTERONE_CLUSTER_10 | -0.0781 | -0.06276 | -1.01475 | 0.313937 | 0.664451 | -5.37253 |
| BASSO_B_LYMPHOCYTE_NETWORK | -0.07534 | -0.08057 | -1.01451 | 0.314054 | 0.664451 | -5.37276 |
| REACTOME_SIGNALLING_TO_P38_VIA_RIT_AND_RIN | 0.081815 | -0.00954 | 1.013719 | 0.314427 | 0.664451 | -5.37349 |
| KEGG_TASTE_TRANSDUCTION | 0.031124 | -0.42202 | 1.013708 | 0.314432 | 0.664451 | -5.3735 |
| SCHUETZ_BREAST_CANCER_DUCTAL_INVASIVE_DN | 0.029769 | -0.0483 | 1.013635 | 0.314467 | 0.664451 | -5.37357 |
| BLANCO_MELO_COVID19_SARS_COV_2_INFECTION_A594_ACE2_EXPRESSING_CELLS_RUXOLITINIB_DN | 0.045127 | -0.04268 | 1.013498 | 0.314531 | 0.664451 | -5.37369 |
| REACTOME_THE_NLRP3_INFLAMMASOME | 0.082527 | 0.005227 | 1.013236 | 0.314656 | 0.664451 | -5.37394 |
| ROVERSI_GLIOMA_LOH_REGIONS | 0.04599 | 0.004659 | 1.013164 | 0.31469 | 0.664451 | -5.374 |
| WU_HBX_TARGETS_1_UP | 0.086336 | -0.06683 | 1.012929 | 0.314801 | 0.664451 | -5.37422 |
| BROWNE_HCMV_INFECTION_48HR_UP | -0.03652 | -0.13205 | -1.01281 | 0.314857 | 0.664451 | -5.37433 |
| KYNG_NORMAL_AGING_DN | -0.07291 | -0.07213 | -1.01238 | 0.315063 | 0.664451 | -5.37474 |
| WP_MEVALONATE_ARM_OF_CHOLESTEROL_BIOSYNTHESIS_PATHWAY | -0.08763 | -0.07268 | -1.01184 | 0.315319 | 0.664451 | -5.37524 |
| WP_VALPROIC_ACID_PATHWAY | 0.056949 | -0.06959 | 1.011789 | 0.315342 | 0.664451 | -5.37528 |
| RODRIGUES_THYROID_CARCINOMA_ANAPLASTIC_UP | -0.05821 | -0.06149 | -1.01177 | 0.315353 | 0.664451 | -5.3753 |
| ELVIDGE_HYPOXIA_DN | -0.06456 | -0.04387 | -1.01171 | 0.315379 | 0.664451 | -5.37535 |
| MILI_PSEUDOPODIA | -0.06291 | -0.10397 | -1.0116 | 0.315432 | 0.664451 | -5.37546 |
| CROONQUIST_IL6_DEPRIVATION_DN | -0.06608 | -0.09477 | -1.01148 | 0.31549 | 0.664451 | -5.37557 |
| REACTOME_FLT3_SIGNALING_THROUGH_SRC_FAMILY_KINASES | 0.112199 | 0.001676 | 1.011405 | 0.315524 | 0.664451 | -5.37564 |
| WP_HEPATOCYTE_GROWTH_FACTOR_RECEPTOR_SIGNALING | -0.07078 | -0.0837 | -1.01127 | 0.315589 | 0.664451 | -5.37576 |
| WP_ENDODERM_DIFFERENTIATION | -0.0304 | -0.08276 | -1.01113 | 0.315657 | 0.664451 | -5.3759 |
| KIM_ALL_DISORDERS_CALB1_CORR_UP | -0.0659 | -0.06975 | -1.01046 | 0.315972 | 0.664451 | -5.37651 |
| CORRE_MULTIPLE_MYELOMA_UP | 0.042664 | -0.01852 | 1.010461 | 0.315973 | 0.664451 | -5.37651 |
| REACTOME_METALLOTHIONEINS_BIND_METALS | 0.086152 | -0.00127 | 1.010336 | 0.316032 | 0.664451 | -5.37663 |
| REACTOME_METHYLATION | -0.0787 | 0.007589 | -1.01006 | 0.316162 | 0.664451 | -5.37688 |
| CHIARADONNA_NEOPLASTIC_TRANSFORMATION_KRAS_DN | 0.0588 | -0.10634 | 1.009812 | 0.316281 | 0.664451 | -5.37711 |
| GARGALOVIC_RESPONSE_TO_OXIDIZED_PHOSPHOLIPIDS_TURQUOISE_DN | -0.05228 | -0.08365 | -1.00944 | 0.316459 | 0.664451 | -5.37746 |
| WP_P38_MAPK_SIGNALING_PATHWAY | -0.06284 | -0.12778 | -1.00934 | 0.316504 | 0.664451 | -5.37755 |
| REACTOME_SIGNALING_BY_RECEPTOR_TYROSINE_KINASES | 0.037813 | -0.04117 | 1.008778 | 0.316773 | 0.664451 | -5.37807 |
| BOYAULT_LIVER_CANCER_SUBCLASS_G3_DN | -0.04379 | -0.07036 | -1.00875 | 0.316785 | 0.664451 | -5.37809 |
| GAUSSMANN_MLL_AF4_FUSION_TARGETS_E_DN | 0.05654 | -0.19401 | 1.008478 | 0.316916 | 0.664451 | -5.37835 |
| NIKOLSKY_BREAST_CANCER_12Q24_AMPLICON | 0.059362 | -0.21926 | 1.008322 | 0.31699 | 0.664451 | -5.37849 |
| KAAB_FAILED_HEART_ATRIUM_DN | -0.07699 | -0.07765 | -1.00808 | 0.317106 | 0.664451 | -5.37872 |
| WP_HYPOTHETICAL_CRANIOFACIAL_DEVELOPMENT_PATHWAY | -0.06725 | -0.00493 | -1.00798 | 0.317153 | 0.664451 | -5.37881 |
| BIOCARTA_PYK2_PATHWAY | -0.08034 | -0.05958 | -1.00748 | 0.317392 | 0.664451 | -5.37927 |
| THUM_MIR21_TARGETS_HEART_DISEASE_UP | 0.096326 | -0.02207 | 1.007307 | 0.317474 | 0.664451 | -5.37943 |
| NAKAMURA_METASTASIS | 0.052378 | -0.06961 | 1.007291 | 0.317482 | 0.664451 | -5.37945 |
| REACTOME_ION_TRANSPORT_BY_P_TYPE_ATPASES | -0.04183 | -0.02035 | -1.00702 | 0.317612 | 0.664451 | -5.3797 |
| KEGG_NEUROTROPHIN_SIGNALING_PATHWAY | -0.05059 | -0.03312 | -1.00696 | 0.317642 | 0.664451 | -5.37976 |
| BROWNE_HCMV_INFECTION_8HR_UP | 0.044347 | -0.10087 | 1.006826 | 0.317703 | 0.664451 | -5.37987 |
| REACTOME_FGFR3_LIGAND_BINDING_AND_ACTIVATION | 0.072711 | 0.020846 | 1.006702 | 0.317763 | 0.664451 | -5.37999 |
| REACTOME_SRP_DEPENDENT_COTRANSLATIONAL_PROTEIN_TARGETING_TO_MEMBRANE | -0.08139 | -0.12025 | -1.00659 | 0.317817 | 0.664451 | -5.38009 |
| PID_ARF_3PATHWAY | 0.081382 | 0.005517 | 1.006158 | 0.318022 | 0.664459 | -5.38049 |
| REACTOME_SUMOYLATION_OF_CHROMATIN_ORGANIZATION_PROTEINS | -0.06378 | -0.09854 | -1.00609 | 0.318054 | 0.664459 | -5.38055 |
| REACTOME_CYTOSOLIC_IRON_SULFUR_CLUSTER_ASSEMBLY | -0.06853 | -0.00652 | -1.00592 | 0.318134 | 0.664459 | -5.38071 |
| REACTOME_DEGRADATION_OF_DVL | -0.06376 | -0.23887 | -1.00509 | 0.318533 | 0.665061 | -5.38148 |
| REACTOME_SIGNALING_BY_CSF3_G_CSF | 0.07797 | 0.010894 | 1.004882 | 0.318631 | 0.665061 | -5.38167 |
| KEGG_INOSITOL_PHOSPHATE_METABOLISM | -0.04674 | -0.06033 | -1.00434 | 0.318892 | 0.665386 | -5.38217 |
[truncated: 881,613 more chars]
