# Supplementary material for: Integrated analysis of bulk RNA-seq and single-cell RNA-seq reveals the function of pyrocytosis in the pathogenesis of abdominal aortic aneurysm
Source: Aging (Albany NY). 2023 Dec 18;15(24):15287–323. doi: 10.18632/aging.205350 (PMC10781497; doi:10.18632/aging.205350)
Supplement: Supplementary Table 2 [file aging-15-205350-s003.pdf]

SUPPLEMENTARY TABLE

Supplemental Table 2. The gene information of pyrocytosis-related genes.

Genes	Full-names
AIM2	Absent in melanoma 2
BAK1	BRI1-associated receptor kinase 1
BAX	Bcl-2-associated X protein
CASP1	Cysteine-aspartic acid protease-1
CASP3	Cysteine-aspartic acid protease-3
CASP4	Cysteine-aspartic acid protease-4
CASP5	Cysteine-aspartic acid protease-5
CASP6	Cysteine-aspartic acid protease-6
CASP8	Cysteine-aspartic acid protease-8
CASP9	Cysteine-aspartic acid protease-9
CHMP2A	Charged Multivesicular Body Protein 2A
CHMP2B	Charged Multivesicular Body Protein 2B
CHMP3	Charged Multivesicular Body Protein 3
CHMP4A	Charged Multivesicular Body Protein 4A
CHMP4B	Charged Multivesicular Body Protein 4B
CHMP4C	Charged Multivesicular Body Protein 4C
CHMP6	Charged Multivesicular Body Protein 6
CHMP7	Charged Multivesicular Body Protein 7
CYCS	Recombinant Cytochrome C, Somatic
ELANE	Elastase, neutrophil expressed
GPX4	Glutathione peroxidase 4
GSDMA	Gasdermin A
GSDMB	Gasdermin B
GSDMC	Gasdermin C
GSDMD	Gasdermin D
GSDME	Gasdermin E
GZMA	Granzyme A
GZMB	Granzyme B
HMGB1	High-mobility group box-1 protein
IL18	Interleukin 18
IL1A	Interleukin 1 a
IL1B	Interleukin 1 beta
IL6	Interleukin 6
IRF1	Interferon regulatory factor 1
IRF2	Interferon regulatory factor 2
NLRC4	NLR family CARD domain containing 4
NLRP1	NLR family pyrin domain containing 1
NLRP2	NLR family pyrin domain containing 2
NLRP3	NLR family pyrin domain containing 3
NLRP6	NLR family pyrin domain containing 6
NLRP7	NLR family pyrin domain containing 7
NOD1	Nucleotide binding oligomerization domain containing 1
NOD2	Nucleotide binding oligomerization domain containing 2
PJVK	Pejvakin/deafness, autosomal recessive 59

PLCG1	Phospholipase C gamma 1
PRKACA	Protein kinase camp-activated catalytic subunit alpha
PYCARD	PYD and CARD domain containing
SCAF11	SR-related CTD associated factor 11
TIRAP	TIR domain containing adaptor protein
TNF	Tumor necrosis factor
TP53	Tumor Protein P53
TP63	Tumor Protein P63
